# Built-in electric field-boosted attomolar Pb²⁺ sensing on carboxylated black phosphorene

**DOI:** 10.1038/s41467-026-77793-6

**Published:** 2026-09-14

**Authors:** Fan Yang, Xixi Yuan, Jiaqi Zhu, Yuanyuan Pan, Zhongjun Li, Jianqing Li, Yanqi Ge, Leiming Wu, Yuanjiang Xiang, Han Zhang, Jun Yang, Yuwen Qin

**Affiliations:** 1 Institute of Advanced Photonics Technology, School of Information Engineering, Guangdong University of Technology, Guangzhou, China; 2 Key Laboratory of Photonic Technology for Integrated Sensing and Communication (Guangdong University of Technology), Ministry of Education of China, Guangzhou, China; 3 Guangdong Provincial Key Laboratory of Information Photonics Technology, Guangdong University of Technology, Guangzhou, China; 4 School of Computer Science and Technology, Guangdong University of Technology, Guangzhou, China; 5 School of Computer Science and Engineering, Macau University of Science and Technology, Macau, China; 6 Institute of Systems Engineering, Macau University of Science and Technology, Macau, China; 7 State Key Laboratory of Radio Frequency Heterogeneous Integration, College of Physics and Optoelectronic Engineering, Shenzhen University, Shenzhen, China; 8 College of Electrical and Electronic Engineering, Wenzhou University, Wenzhou, China

**Keywords:** Optical materials and structures, Materials for optics

## Abstract

Lead ion (Pb²⁺) contamination poses a severe and persistent threat to environmental safety and human health, demanding ultrasensitive and selective detection strategies. Herein, we fabricate a carboxyl-functionalized black phosphorene-modified surface plasmon resonance sensor (Au+BP-COOH) with built-in electric field enhancement for attomolar-level and highly selective Pb²⁺ detection. The Au/BP-COOH junction generates a strong interfacial built-in electric field, which strengthens near-field electromagnetic coupling and amplifies the SPR optical response, thereby boosting the detection sensitivity. Meanwhile, abundant oxygen-containing sites on BP-COOH enable efficient and preferential interaction with Pb²⁺. Our sensor achieves a detection limit of 10⁻¹⁹ M, 14 orders of magnitude lower than that of bare Au, and exhibits remarkable selectivity against Cd²⁺, Cu²⁺, Zn²⁺, Ni²⁺, Ca²⁺, and Mg²⁺. This label-free and straightforward platform provides a reliable route for ultratrace heavy-metal monitoring in environmental and food-safety applications.

## Abstract (web summary)

Lead ion (Pb²⁺) contamination remains a threat to environmental safety and human health, requiring sensitive detectors. Here, the authors report the realization of carboxyl-functionalized black phosphorene modified surface plasmon resonance sensors with detection limit down to 10⁻¹⁹ M and good selectivity against other metal ions.

## Introduction

Lead contamination remains a persistent environmental and public-health concern. Recent high-level assessments have shown that toxic metal pollution has become a global threat to agricultural sustainability, food safety, and human health^1–3^. Importantly, the risk is not limited to heavily polluted scenarios. Recent evidence indicates that even when heavy-metal concentrations in food formally satisfy existing regulatory thresholds, the corresponding dietary intake may still impose non-negligible health risks^4^. Within this broader context, Pb²⁺ remains particularly concerning because of its persistence across water–soil–food systems, its continuing exposure burden in modern populations, and its well-established toxicity to neurological and developmental health^5–7^. These considerations continue to motivate the development of analytical strategies capable of identifying Pb²⁺ at extremely low concentrations.

A central difficulty in Pb²⁺ monitoring is that environmentally relevant exposure may arise from long-term, low-level, and mixed-source conditions rather than only from highly contaminated samples. Recent work on chemical exposure through drinking water has further emphasized that analytical monitoring must increasingly address low-abundance contaminants in complex exposure pathways^8^. For Pb²⁺, this requirement places particular pressure on sensing platforms to combine high sensitivity with simple operation and reliable interfacial readout. In practice, a desirable Pb²⁺ sensor should not only provide a low limit of detection, but should also maintain a direct and interpretable signal-transduction mechanism on a stable sensing interface.

Substantial progress has been made in Pb²⁺ sensing over the past decade. Fluorescent probes, colorimetric platforms, electrochemical sensors, and photoelectrochemical strategies have all been developed to improve sensitivity through molecular recognition and signal amplification^9–13^. Fluorescence-based methods offer convenient optical readout and have reached low concentration regimes through probe engineering. For example, a single-labeled fluorescent oligonucleotide probe enabled Pb²⁺ detection down to 96 pmol/L, whereas a peptide-based ratiometric fluorescent probe reported a limit of detection of 3.8 nM^9,10^. Electrochemical and photoelectrochemical approaches can be pushed further when coupled with DNAzyme cleavage or amplification cascades. A single-step DNAzyme electrochemical sensor achieved a limit of detection of 1.26 fM, and a homogeneous dual-amplification electrochemical biosensor further reported 16.7 fM Pb²⁺ analysis, while a recent DNAzyme-assisted photoelectrochemical platform operated at 16.67 pmol/L^11–13^. These studies clearly demonstrate that ultra-trace Pb²⁺ analysis is achievable. However, many of these platforms rely on labels, multistep reaction schemes, or amplification modules that are not intrinsic to the sensing interface itself. For a conventional optical platform that reads out surface events directly and in real time, achieving such low concentration detection remains substantially more challenging.

Surface plasmon resonance (SPR) is especially attractive in this regard because it provides a label-free method for converting local refractive-index perturbations at a metal interface into measurable optical signals^14–16^. Since the first demonstration of SPR sensing in the early 1980s^14^, and its subsequent development into a widely used analytical technology^15^, prism-coupled SPR has become a central tool for interfacial analysis in chemistry, biology, and materials science. A recent authoritative primer further emphasized that prism-coupled SPR remains the most broadly used configuration for in situ monitoring of thin films, molecular adsorption, and interfacial interactions^16^. These features are directly relevant to metal-ion detection because they allow the sensing event to be read from the interface itself, without fluorescent tags or electrochemical mediators.

Despite these advantages, bare-Au SPR remains intrinsically challenged for Pb²⁺ sensing. The first challenge is physical: small ions induce only weak interfacial refractive-index perturbations, so the corresponding resonance-angle shift is often limited at low concentration. The second challenge is chemical: if the metal surface lacks favorable binding sites, the interfacial response to Pb²⁺ can remain weak and insufficiently differentiated from that of other ions. Existing Pb²⁺ SPR studies have partially addressed these issues through DNAzyme-assisted recognition, nanoparticle conjugation, or alternative assay configurations^17–19^. For example, a label-free SPR sensor based on DNAzyme–gold nanoparticle conjugates reported an 80 pM detection limit, and an SPR-based assay kit achieved 1 nM detection, whereas a fiber-based SPR configuration operated at approximately 4.8 × 10⁻⁹ M^17–19^. Earlier amplified SPR work based on a Pb²⁺-dependent DNAzyme and a hemin/G-quadruplex label reported 5 fM detection, but this relied on an amplification strategy rather than a simple direct interfacial design^20^. Together, these studies improved SPR performance, but they also illustrate the general difficulty of pushing an SPR platform toward ultra-trace Pb²⁺ detection while preserving structural simplicity. In particular, the concentration regimes accessible to reported Pb²⁺ SPR systems still remain substantially higher than those reached by highly amplified electrochemical routes^11,12^.

One promising way to overcome this limitation is to engineer the plasmonic interface with an ultrathin functional layer. In this strategy, the overlayer is expected to do more than simply decorate the surface. It should simultaneously amplify the effective optical perturbation experienced by the plasmonic field and provide chemically favorable sites for target binding. Two-dimensional materials are appealing in this context because they combine atomic-to-nanoscale thickness, high surface exposure, and tunable interfacial chemistry, and recent analyses of 2D material-based SPR biosensors further underscore the value of such ultrathin interfacial layers for sensitivity enhancement^21,22^. Representative recent studies have also shown that highly active 2D recognition surfaces can enable attomolar-level metal-ion detection when the interfacial chemistry is appropriately designed^23,24^. These advances underscore an important principle: the sensing limit is not determined only by the transducer itself, but also by how efficiently the interface converts analyte binding into an optical or electrical perturbation.

Among the available 2D materials, BP is especially attractive for plasmonic sensing because of its distinctive electronic structure and strong surface sensitivity^25,26^. Since its emergence as a 2D electronic material with unusual optoelectronic properties^25^, BP has been widely recognized as a promising platform for sensing applications^26,27^. At the same time, its practical use depends strongly on interfacial regulation. BP is chemically reactive and prone to degradation under ambient conditions, so protective or functional surface chemistry is often essential for stabilizing the material and making its reactivity analytically useful^28^. In the SPR field, previous studies have already suggested that few-layer BP can improve angular sensitivity by modifying the interfacial optical field and coupling characteristics^22,29^. More recent progress in solution-processable 2D material inks also supports the broader feasibility of constructing functionalized ultrathin 2D interfaces for device applications^30^. Together, these developments make BP a strong candidate for building an SPR interface that is both optically responsive and chemically addressable.

Here, we construct a built-in electric field-enhanced Au + BP-COOH by directly stacking a BP-COOH functional layer onto a prism-coupled Au-film SPR platform. In this design, the BP layer is intended to enhance the angular SPR response, while the oxygen-containing chemical environment of the BP-COOH functional layer provides favorable interfacial sites for Pb²⁺ interaction. Unlike recognition strategies that depend on DNAzymes, labels, or multistep amplification reactions, this structure retains the simplicity of a bare-Au SPR platform while directly engineering the sensing interface itself. Under the same sensing framework, the limit of detection for Pb²⁺ reaches 10⁻¹⁹ M for the proposed built-in electric field-enhanced Au + BP-COOH, whereas bare Au shows a distinguishable Pb²⁺ response from 10⁻⁵ M. This study develops a simple yet effective modification route, which advances SPR sensing for ultratrace Pb²⁺ monitoring.

## Results

### Interfacial structure and Pb²⁺ recognition pathway

**Fig. 1:**
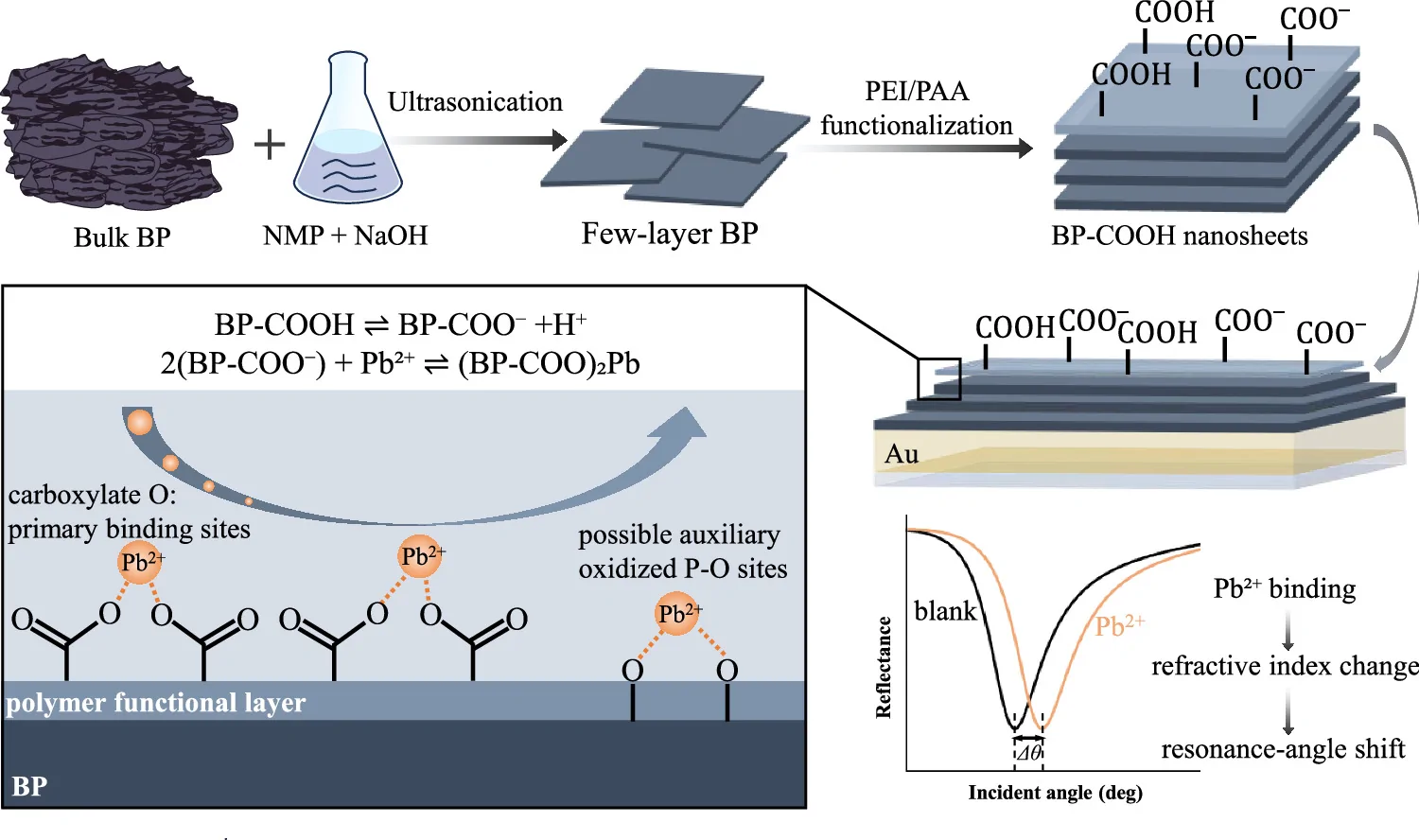
Preparation of carboxyl-functionalized black phosphorus (BP) nanosheets (BP-COOH), construction of built-in electric field-enhanced Au + BP-COOH, and schematic illustration of the proposed Pb²⁺ recognition pathway. Bulk BP was exfoliated in N-methyl-2-pyrrolidone (NMP) + NaOH to obtain few-layer BP, followed by polyethyleneimine (PEI)/poly(acrylic acid) (PAA) functionalization to form BP-COOH nanosheets. The BP-COOH nanosheets were then deposited onto the Au film to construct Au + BP-COOH. The lower schematic shows the proposed interaction of Pb²⁺ with oxygen-containing sites in the BP-COOH functional layer, where Δ*θ* denotes the resonance-angle shift induced by the interfacial refractive-index change.

As illustrated in Fig. 1, an SPR sensor modified with BP-COOH was constructed by depositing a BP-COOH functional layer onto a prism-coupled Au film. The BP-COOH nanosheets were obtained by liquid-phase exfoliation of bulk BP followed by PEI/PAA functionalization, which introduced oxygen-containing surface groups to the nanosheet interface. In the resulting Au + BP-COOH structure, the BP-COOH functional layer serves as the recognition-active interface, while the underlying Au film serves as the plasmonic transduction layer. According to the schematic shown in Fig. 1, carboxylate O sites are expected to be the major interaction sites for Pb²⁺, whereas oxidized P–O sites may provide an auxiliary contribution.

On this basis, the selective interaction mechanism of Pb²⁺ at the BP-COOH interface can be described by the following interfacial reactions:

1 $${{{\rm{BP}}}}-{{{\rm{COOH}}}}\rightleftharpoons {{{\rm{BP}}}}-{{{{\rm{COO}}}}}^{-}+{{{{\rm{H}}}}}^{+}$$

2 $$2\left({{{\rm{BP}}}}-{{{{\rm{COO}}}}}^{-}\right)+{{{{\rm{Pb}}}}}^{2+}\rightleftharpoons {\left({{{\rm{BP}}}}-{{{\rm{COO}}}}\right)}_{2}{{{\rm{Pb}}}}$$

These equations are used here to describe a plausible oxygen-site-mediated interfacial pathway for BP-COOH. A related mechanistic logic has been discussed for Pb(II) uptake on other oxygen-containing two-dimensional surfaces, where hydroxyl-related ion-exchange behavior was considered, the surface process was written in explicit reaction form, and the corresponding XPS results were consistent with strong Pb interaction with O-containing sites^31^. Although the surface chemistry in that system is different from that of BP-COOH, this provides one useful basis for expressing the present Pb²⁺ interaction in reaction form. Under this proposed pathway, Pb²⁺ interaction with the BP-COOH functional layer perturbs the local interfacial refractive index and leads to a resonance-angle shift in the SPR response, as schematically shown in Fig. 1.

**Fig. 2:**
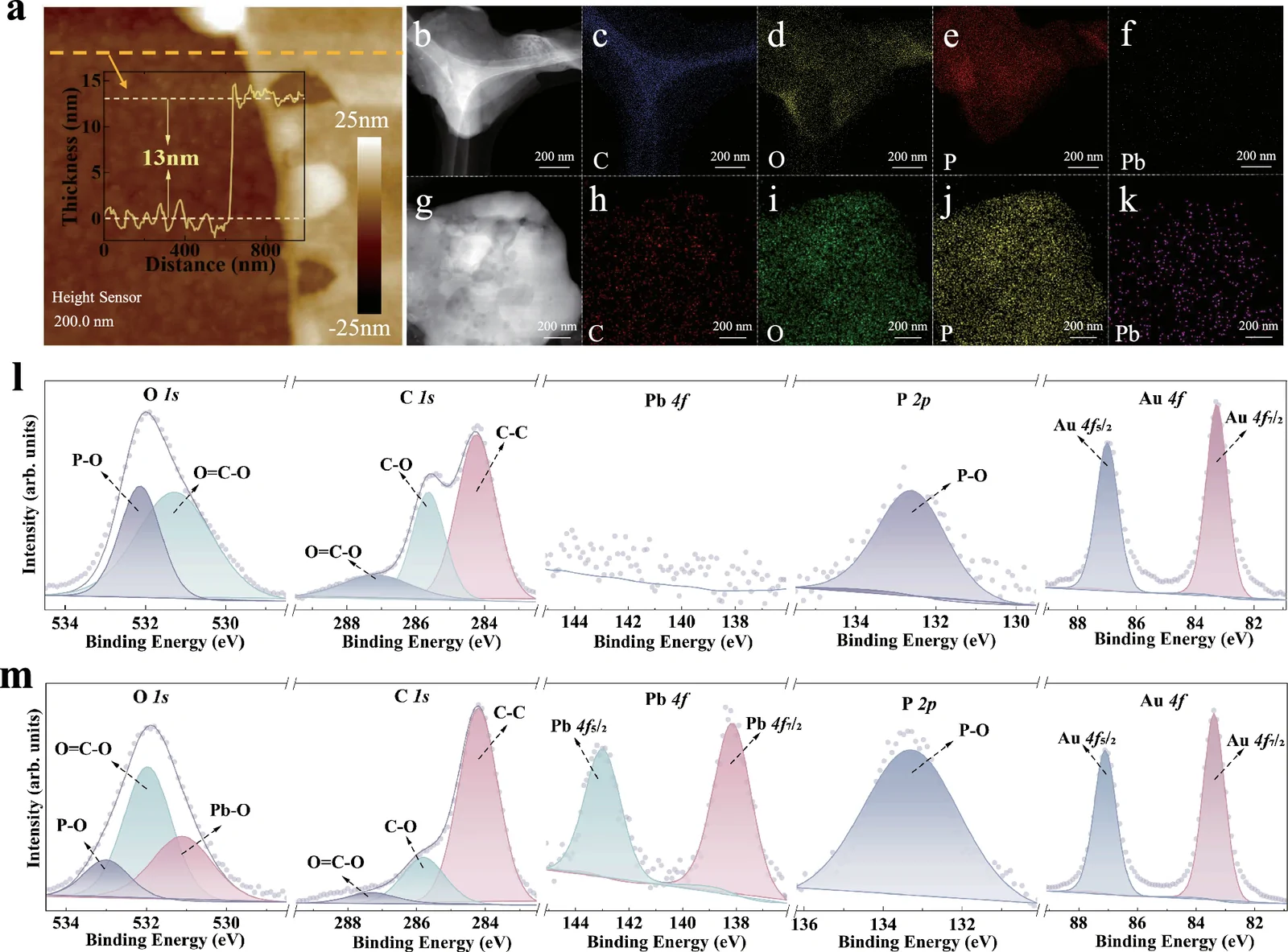
Atomic force microscopy (AFM), scanning transmission electron microscopy (STEM) images, energy-dispersive X-ray spectroscopy (EDS) elemental maps, and X-ray photoelectron spectroscopy (XPS) characterization of the Au + BP-COOH interface before and after Pb²⁺ exposure. **a** AFM image and height profile of the BP-COOH functional layer on the Au film, showing a thickness of approximately 13 nm. **b** STEM image of the BP-COOH functional layer before Pb²⁺ exposure. **c–f** Corresponding EDS elemental maps of C, O, P, and Pb before Pb²⁺ exposure. **g** STEM image of the BP-COOH functional layer after Pb²⁺ exposure. **h–k** Corresponding EDS elemental maps of C, O, P, and Pb after Pb²⁺ exposure. **l** XPS spectra of O 1*s*, C 1*s*, Pb 4*f*, P 2*p*, and Au 4*f* before Pb²⁺ exposure. **m** XPS spectra of O 1*s*, C 1*s*, Pb 4*f*, P 2*p*, and Au 4*f* after Pb²⁺ exposure.

To examine the structural basis and chemical plausibility of this proposed oxygen-site-mediated pathway, the Au + BP-COOH interface was characterized before and after Pb²⁺ exposure by AFM, STEM imaging, EDS elemental mapping, and XPS. As shown in Fig. 2a, AFM confirms that the Au surface is covered by an ultrathin BP-COOH functional layer with a thickness of approximately 13 nm, which was used as the representative structure in the main-text sensing experiments. Additional AFM characterization of ten independently prepared Au + BP-COOH chips is provided in Supplementary Fig. 2. The corresponding height profiles show that the BP-COOH functional layers are mainly maintained in a comparable thickness range of approximately 10–14 nm, indicating that the chips used in the subsequent sensing measurements possess similar BP-COOH coverage. Before Pb²⁺ exposure, the STEM image in Fig. 2b together with the EDS elemental maps in Fig. 2c–f shows the presence of C, O, and P on the nanosheet-covered region, while no obvious Pb signal is detected. After Pb²⁺ exposure, the nanosheet-covered morphology is retained in Fig. 2g, and the corresponding EDS elemental maps in Fig. 2h–k show the coexistence of C, O, P, and Pb, indicating that Pb-containing species are present on the BP-COOH-modified interface.

The XPS results in Fig. 2l, m further support this interfacial evolution. Before reaction, the O 1*s* spectrum contains P–O and O=C–O components, while the C 1*s* spectrum shows C–C, C–O, and O=C–O contributions, consistent with the presence of the BP-COOH functional layer on the Au surface. The P 2*p* region is located in the range of oxidized phosphorus species, and the Au 4*f* doublet from the underlying Au film remains clearly visible. No obvious Pb 4*f* signal is observed before Pb²⁺ exposure. After reaction, the C 1*s* spectrum remains dominated by C–C, C–O, and O=C–O components, indicating that the BP-COOH functional layer is preserved, whereas the O 1*s* spectrum shows an additional Pb–O-related component together with the original P–O and O=C–O signals. At the same time, a distinct Pb 4*f* doublet assigned to Pb 4*f*₅/₂ and Pb 4*f*₇/₂ appears after reaction. The P 2*p* envelope remains mainly associated with oxidized phosphorus species before and after Pb²⁺ exposure. Taken together, the emergence of Pb in the EDS elemental maps, the appearance of Pb 4*f*, and the Pb–O-related component in O 1*s* are consistent with Pb²⁺ interaction with oxygen-containing sites in the BP-COOH functional layer. The structural retention of BP-COOH during storage was further examined by Raman spectroscopy. As shown in Supplementary Fig. 1, Raman spectra recorded from Day 1 to Day 7 retain the Raman features of BP-COOH during this period. Although slight intensity variations are observed, the BP-related Raman response is generally preserved, indicating that the BP-COOH functional material maintains its main structural characteristics during short-term storage.

Overall, Figs. 1 and 2 are used together to clarify the interfacial structure and proposed recognition mechanism of Au + BP-COOH. Figure 1 defines the architecture of the sensing interface and gives a chemically reasonable description of Pb²⁺ interaction at oxygen-containing sites, whereas Fig. 2 provides morphological, elemental, and chemical-state evidence that the BP-COOH functional layer is successfully formed on the Au film and undergoes corresponding interfacial changes after Pb²⁺ exposure. These results establish the structural and mechanistic basis for the subsequent analysis of sensitivity enhancement and Pb²⁺ sensing performance.

### Optical basis of BP-enhanced sensitivity

**Fig. 3:**
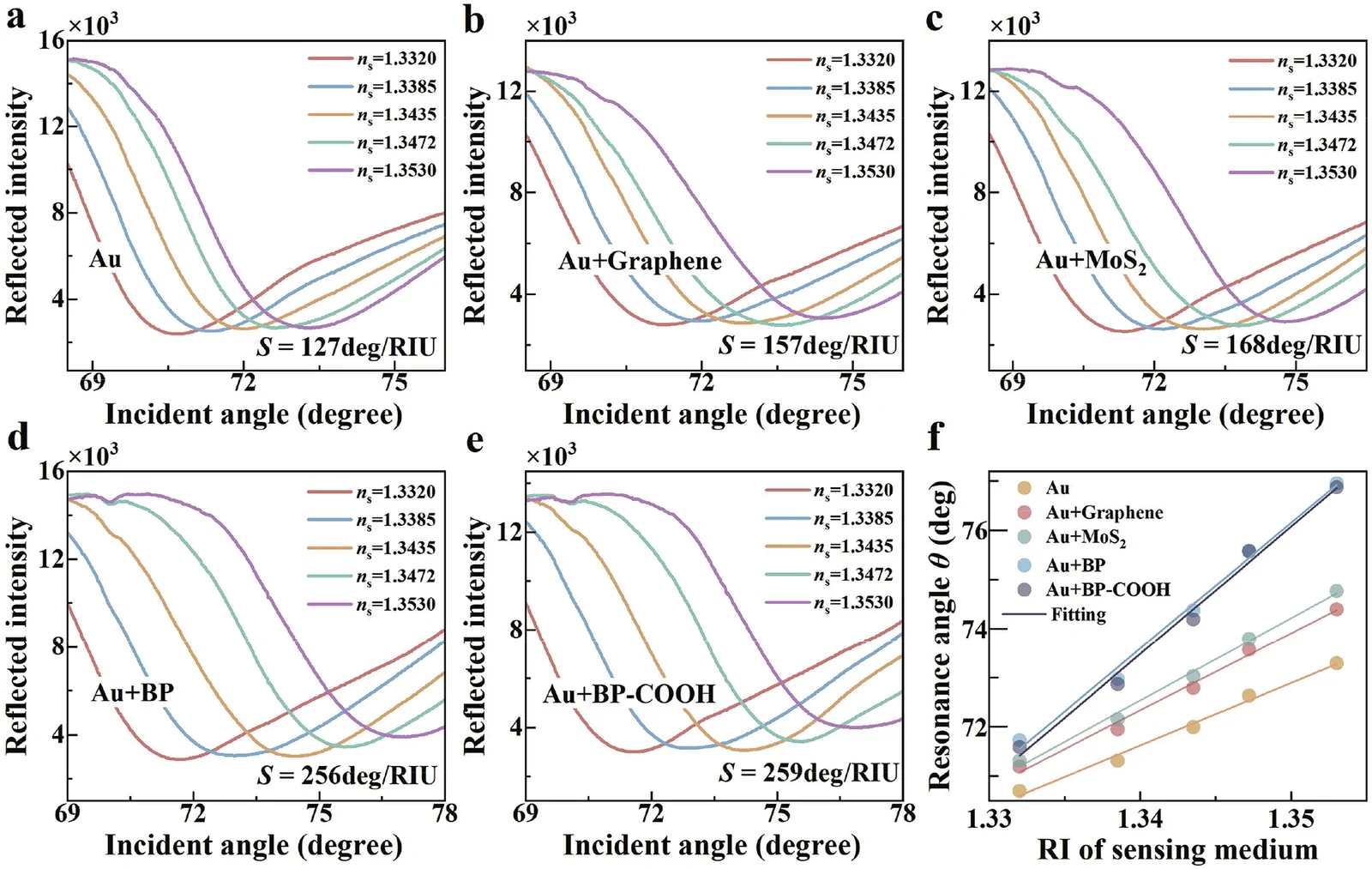
Experimental refractive-index calibration of bare Au, Au + Graphene, Au + MoS₂, Au + BP, and Au + BP-COOH. Angle-scanned reflected-intensity curves of **a** bare Au, **b** Au + Graphene, **c** Au + MoS₂, **d** Au + BP, and **e** Au + BP-COOH measured in sensing media with *n*_s_ = 1.3320, 1.3385, 1.3435, 1.3472, and 1.3530. **f** Resonance angle as a function of the refractive index (RI) of the sensing medium for bare Au, Au + Graphene, Au + MoS₂, Au + BP, and Au + BP-COOH, together with linear fitting curves. *S* denotes angular sensitivity, *θ* denotes the resonance angle, and *n*_s_ denotes the refractive index of the sensing medium.

To clarify the optical basis for the improved sensing performance of Au + BP-COOH, the refractive-index-dependent angular SPR responses of bare Au, Au + Graphene, Au + MoS₂, Au + BP, and Au + BP-COOH were experimentally compared under the same sensing framework. As shown in Fig. 3a–e, the angle-scanned reflected-intensity curves of all five structures shift toward larger incident angles when the refractive index of the sensing medium increases from *n*_s_ = 1.3320 to 1.3530. However, the magnitude of the resonance-angle displacement is different for different interfacial structures.

The corresponding resonance angles are summarized in Fig. 3f. Linear fitting gives angular sensitivities of 127 deg/RIU for bare Au, 157 deg/RIU for Au+Graphene, 168 deg/RIU for Au + MoS₂, 256 deg/RIU for Au + BP, and 259 deg/RIU for Au + BP-COOH. Compared with bare Au, the introduction of two-dimensional overlayers increases the angular response of the Au-film SPR structure to refractive-index variation. Au + Graphene and Au + MoS₂ were used as reference two-dimensional overlayer structures because they also possess high-index confinement and interlayer-coupling-related optical characteristics. However, their angular sensitivities remain lower than those of Au + BP and Au + BP-COOH under the same refractive-index calibration conditions. This comparison indicates that the BP-based overlayer provides a stronger optical response than the other two-dimensional overlayers examined here. In addition, the angular sensitivities of Au + BP and Au + BP-COOH are close to each other, indicating that carboxyl functionalization preserves the BP-related optical response while introducing oxygen-containing interfacial sites for subsequent Pb²⁺ interaction.

The simulated angular SPR response and electric-field distribution of the Au-film structure with and without a BP overlayer are provided in Supplementary Fig. 3. The simulation shows the same enhancement trend after introducing the BP overlayer and provides a theoretical reference for the BP-induced optical response. In the experimental Au + BP-COOH structure, the BP-COOH functional layer contains polymer-functionalized BP nanosheets, and therefore the experimental refractive-index calibration in Fig. 3 is used as the direct basis for evaluating the optical response of the actual sensing interface.

**Fig. 4:**
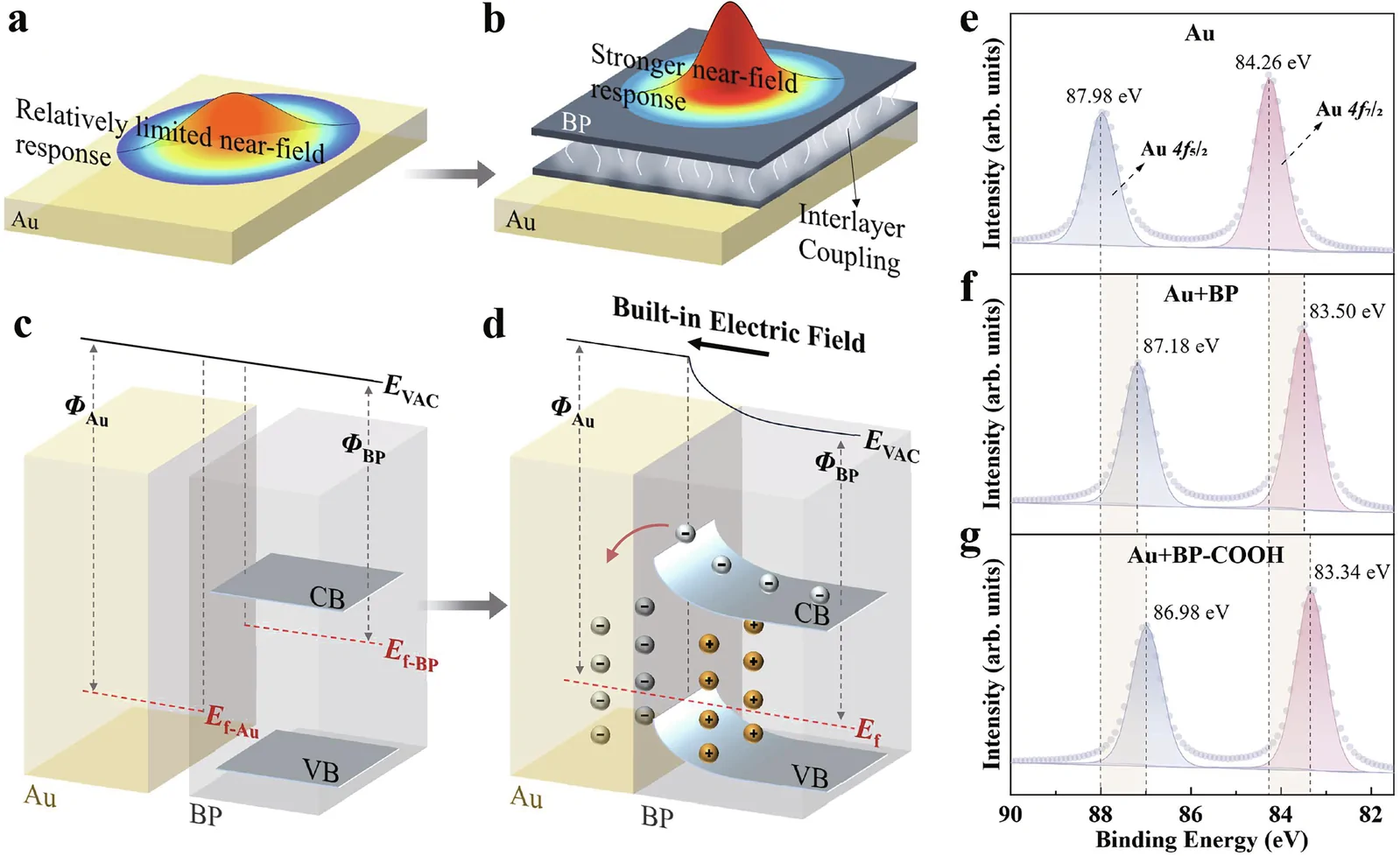
Schematic physical interpretation of BP-enhanced near-field response at the surface plasmon resonance (SPR) interface and XPS characterization of interfacial charge redistribution. **a** Schematic near-field distribution at the Au/sensing-medium interface, showing a relatively limited near-field response. **b** Schematic near-field distribution after introduction of the BP overlayer, showing enhanced field confinement and a stronger near-field response; interlayer coupling within the multilayer BP structure is also illustrated. **c** Schematic energy-level alignment of Au and BP before interfacial charge redistribution, showing the work functions and band positions of the two components. **d** Schematic interfacial charge-transfer process after Au/BP contact, showing the formation of a built-in electric field and the resulting enhancement of local field coupling at the interface. In (**c**, **d**), *Φ*_Au_ and *Φ*_BP_ denote the work functions of Au and BP, respectively; CB and VB denote the conduction band and valence band of BP, respectively; *E*_f-Au_ and *E*_f-BP_ denote the Fermi levels of Au and BP before contact, respectively; *E*_VAC_ denotes the vacuum energy level; and *E*_f_ denotes the equilibrium Fermi level after contact. **e–g** High-resolution Au 4*f* XPS spectra of bare Au, Au + BP, and Au + BP-COOH, respectively. The Au 4*f*₇/₂ and Au 4*f*₅/₂ peaks are located at 84.26 and 87.98 eV for bare Au, 83.50 and 87.18 eV for Au + BP, and 83.34 and 86.98 eV for Au + BP-COOH, respectively.

The physical origin of this enhanced near-field response is schematically summarized in Fig. 4. As illustrated in Fig. 4a, b, the introduction of BP changes the interfacial optical environment of the Au-film SPR structure. BP possesses a relatively high refractive index over a broad spectral range, and its introduction onto the Au surface creates a stronger dielectric contrast at the plasmonic interface. Under this condition, the effective mode volume of the evanescent field is compressed, and the electromagnetic field becomes more strongly localized near the sensing interface. In addition, the multilayer structure of BP introduces interlayer coupling, which further enhances optical confinement and contributes to a stronger near-field response than that of the Au/sensing-medium structure.

A second contribution is illustrated in Fig. 4c, d. UPS measurements give work functions of 4.99 eV for bare Au, 4.30 eV for BP, and 4.26 eV for BP-COOH (Supplementary Fig. 4). Thus, BP and BP-COOH exhibit comparable work functions, both lower than that of Au. The corresponding work-function differences are 0.69 eV for Au/BP and 0.73 eV for Au/BP-COOH. Upon contact, these work-function differences drive electron transfer from BP or BP-COOH toward Au until the Fermi levels reach equilibrium, leading to directional interfacial charge redistribution. The high-resolution Au 4*f* XPS spectra in Fig. 4e–g further support this process. The Au 4*f*₇/₂ and Au 4*f*₅/₂ peaks shift from 84.26 and 87.98 eV for bare Au to 83.50 and 87.18 eV for Au+BP, and to 83.34 and 86.98 eV for Au + BP-COOH. These shifts toward lower binding energies are consistent with increased electron density on the Au side after contact, in agreement with the electron-transfer direction indicated by the measured work-function differences. This interfacial charge redistribution leads to the formation of a built-in electric field at the Au/BP and Au/BP-COOH interfaces, which can further strengthen local electromagnetic coupling near the sensing region. In this sense, the role of BP in improving SPR sensitivity is not limited to adding an ultrathin overlayer, but also involves interfacial regulation of the local field environment. BP also exhibits a high carrier mobility, reported up to approximately 10³ cm²/(V·s) at room temperature^25^, which is favorable for efficient interfacial charge response. Taken together, the high-index confinement effect, multilayer coupling effect, and interfacial charge-transfer effect provide a physically reasonable interpretation for the stronger optical response of Au + BP and Au + BP-COOH observed in Fig. 3.

Taken together, Figs. 3, 4 and Supplementary Figs. 3, 4 show that introducing the BP-based overlayer strengthens the interfacial optical response of the Au-film SPR structure to refractive-index variation. The experimental refractive-index calibration verifies the enhanced angular sensitivity of Au + BP and Au + BP-COOH, while the simulation provides a theoretical reference for the BP-overlayer-induced optical response. The physical interpretation illustrated in Fig. 4 is further supported by the UPS work-function measurements in Supplementary Fig. 4 and the Au *4f* XPS spectra in Fig. 4e–g, which together support electron transfer from BP or BP-COOH toward Au and the resulting interfacial charge redistribution and built-in electric field formation shown in Fig. 4c, d. These results provide the optical and interfacial basis for the subsequent improvement in Pb²⁺ sensing performance observed for Au + BP-COOH.

### Pb²⁺ sensing performance and selectivity of Au + BP-COOH

**Fig. 5:**
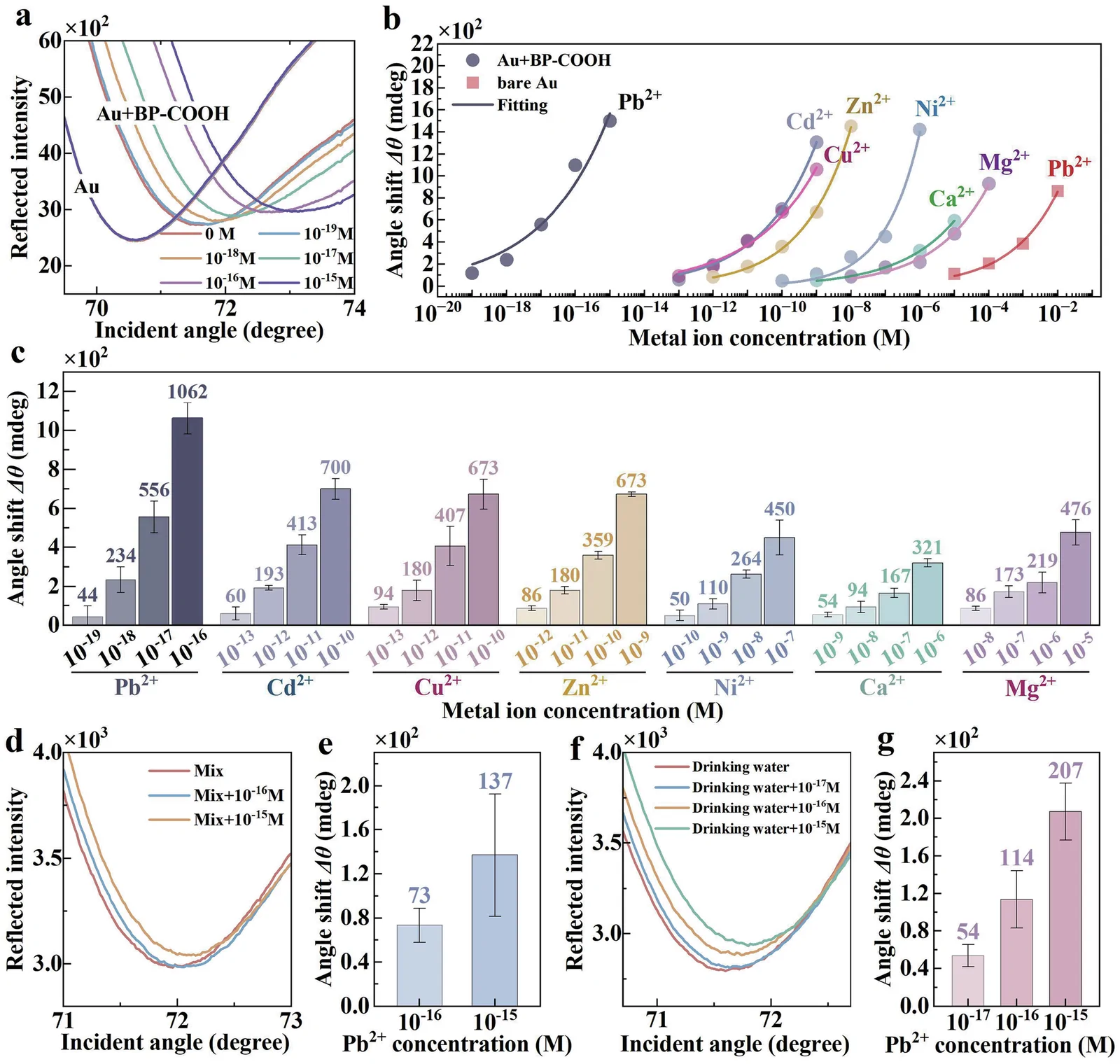
Pb²⁺ sensing response of Au + BP-COOH and comparison with other metal ions in pure water, mixed-ion solution, and drinking water. Mix denotes the mixed-ion solution used as the matrix background. **a** Angle-scanned reflected-intensity curves of Au + BP-COOH measured for Pb²⁺ concentrations of 0, 10⁻¹⁹, 10⁻¹⁸, 10⁻¹⁷, 10⁻¹⁶, and 10⁻¹⁵ M, together with the reflected-intensity curve of bare Au. **b** Resonance-angle shift Δ*θ* as a function of metal-ion concentration for Au + BP-COOH and bare Au. The data points are mean values from the corresponding statistical results in (**c**) and Supplementary Fig. 5, and the solid lines represent nonlinear fitting curves. **c** Resonance-angle shift Δ*θ* values of Au + BP-COOH measured at different concentrations of Pb²⁺, Cd²⁺, Cu²⁺, Zn²⁺, Ni²⁺, Ca²⁺, and Mg²⁺. The Pb²⁺ data are from the ten independently prepared Au + BP-COOH chips shown in Supplementary Figs. 7–16 and statistically summarized in Supplementary Fig. 17, and the data for Cd²⁺, Cu²⁺, Zn²⁺, Ni²⁺, Ca²⁺, and Mg²⁺ are from the three independently prepared Au + BP-COOH chips shown in Supplementary Figs. 26–31. Error bars represent the standard deviation of measurements from these independently prepared chips. **d** Angle-scanned reflected-intensity curves of Au + BP-COOH measured in Mix, Mix + 10⁻¹⁶ M Pb²⁺, and Mix + 10⁻¹⁵ M Pb²⁺. **e** Net resonance-angle shift Δ*θ* as a function of Pb²⁺ concentration in the mixed-ion solution. The statistical data are from Supplementary Fig. 32, and the error bars represent the standard deviation of measurements from three independently prepared Au + BP-COOH chips. **f** Angle-scanned reflected-intensity curves of Au + BP-COOH measured in drinking water, drinking water + 10⁻¹⁷ M Pb²⁺, drinking water + 10⁻¹⁶ M Pb²⁺ and drinking water + 10⁻¹⁵ M Pb²⁺. **g** Net resonance-angle shift Δ*θ* as a function of Pb²⁺ concentration in drinking water. The statistical data are from Supplementary Fig. 33, and the error bars represent the standard deviation of measurements from three independently prepared Au + BP-COOH chips.

The Pb²⁺ sensing performance of Au + BP-COOH is summarized in Fig. 5. As shown in Fig. 5a, when the Pb²⁺ concentration increases from 0 to 10⁻¹⁵ M, the angle-scanned reflected-intensity curves of Au + BP-COOH exhibit concentration-dependent resonance-angle shifts. A distinguishable response is observed at 10⁻¹⁹ M in the representative curve. In contrast, the corresponding bare Au curve remains unresolved within the same low-concentration range, indicating that the BP-COOH functional layer markedly extends the detectable Pb²⁺ concentration regime toward much lower concentrations.

To clarify the contribution of BP and BP-COOH to Pb²⁺ sensing, control measurements were performed on bare Au and Au+BP. The Pb²⁺ sensing results of bare Au and Au + BP are provided in Supplementary Figs. 5 and 6, respectively. Bare Au shows a distinguishable Pb²⁺ response from 10⁻⁵ M, whereas Au+BP shows a distinguishable response from 10⁻⁷ M. This comparison indicates that the BP overlayer improves the Pb²⁺ sensing response relative to bare Au. By further introducing the BP-COOH functional layer, the detectable Pb²⁺ concentration is extended to 10⁻¹⁹ M, indicating that the oxygen-containing interfacial sites of BP-COOH play a key role in the ultralow-concentration Pb²⁺ response.

To examine the statistical detection behavior of Au + BP-COOH at ultralow Pb²⁺ concentrations, time-dependent sensing measurements were performed on ten independently prepared Au + BP-COOH chips. The detection statistics are summarized in Table 1, and the corresponding individual sensing results are provided in Supplementary Figs. 7–16. At 10⁻¹⁹ M Pb²⁺, 1/10, 2/10, 4/10, and 4/10 chips show distinguishable responses at 30, 60, 90, and 120 s, respectively, indicating that this concentration is located in a time-dependent statistical detection regime. In contrast, at 10⁻¹⁸ M and higher Pb²⁺ concentrations, all ten chips show distinguishable responses at each detection time. The statistical resonance-angle shifts obtained from these ten chips are summarized in Supplementary Fig. 17. These results show the time-dependent detectability of Au + BP-COOH at 10⁻¹⁹ M and the consistent detectability from 10⁻¹⁸ to 10⁻¹⁵ M.

**Table 1:** Time-dependent detection statistics of Pb²⁺ obtained from ten independently prepared Au + BP–COOH chips

Pb²⁺ concentration	30 s	60 s	90 s	120 s
10^−19^ M	1/10	2/10	4/10	4/10
10^−18^ M	10/10	10/10	10/10	10/10
10^−17^ M	10/10	10/10	10/10	10/10
10^−16^ M	10/10	10/10	10/10	10/10
10^−15^ M	10/10	10/10	10/10	10/10

A chip was counted as distinguishable when the resonance-angle shift Δ*θ* exceeded 21 mdeg. The statistics were obtained from ten independently prepared Au + BP-COOH chips shown in Supplementary Figs. 7–16.

The concentration dependence of the resonance-angle response is further summarized in Fig. 5b. For Au + BP-COOH, the resonance-angle shift Δ*θ* increases with increasing Pb²⁺ concentration, and the fitted curve extends into the ultralow-concentration regime. Under the same sensing framework, bare Au only begins to show distinguishable Pb²⁺ responses at much higher concentrations. Therefore, relative to bare Au, the BP-COOH functional layer shifts the detectable Pb²⁺ concentration regime toward substantially lower concentrations on the same optical platform.

The selectivity of Au + BP-COOH was further evaluated by comparing its responses to other divalent metal ions. As shown in Fig. 5b, c, the response to Pb²⁺ appears in a substantially lower concentration regime than those of Cd²⁺, Cu²⁺, Zn²⁺, Ni²⁺, Ca²⁺, and Mg²⁺ under identical measurement conditions. The corresponding single-ion sensing results for Cd²⁺, Cu²⁺, Zn²⁺, Ni²⁺, Ca²⁺, and Mg²⁺ are provided in Supplementary Figs. 26–31. The LODs for the other metal ions are 10⁻¹³ M for Cd²⁺, 10⁻¹³ M for Cu²⁺, 10⁻¹² M for Zn²⁺, 10⁻¹⁰ M for Ni²⁺, 10⁻⁹ M for Ca²⁺, and 10⁻⁸ M for Mg²⁺. These results show that Au + BP-COOH exhibits a stronger response to Pb²⁺ than to the other tested divalent ions within the present sensing system.

The preferential response toward Pb²⁺ may arise from the combined effects of hydration-shell stability, ionic size and polarizability, coordination geometry, HSAB interactions, and the accessibility of oxygen-containing sites in BP-COOH. Pb²⁺ is a highly polarizable divalent ion with a stereochemically active *6s*² lone pair, allowing variable coordination numbers and asymmetric geometries; together with its borderline-soft acid character, this coordination flexibility may favor its interaction with surface oxygen-containing groups^31,32^. In comparison, Ca²⁺ and Mg²⁺ are strongly hydrated^33^, whereas the interaction of Cu²⁺, Ni²⁺, and Zn²⁺ with these sites may depend on ion-specific dehydration and reorganization of their more defined hydrated coordination structures^34^. Although Cd²⁺ is less strongly hydrated and can also coordinate with oxygen-containing groups^33,34^, the higher polarizability and *6s*²-lone-pair-associated coordination flexibility of Pb²⁺ may further favor its interfacial interaction^32^. The observed response hierarchy may therefore reflect the combined influence of these ion-dependent factors.

Mixed-ion interference and real-sample measurements were further performed to evaluate the sensing response under more complex matrix conditions. In the mixed-ion experiment, Mix was used as the matrix background, and the net Pb²⁺ response was obtained by subtracting the Mix baseline from the Mix + Pb²⁺ response. As shown in Fig. 5d, e and Supplementary Fig. 32, Pb²⁺ remains distinguishable in the mixed-ion solution at 10⁻¹⁶ M. In the real-sample experiment, drinking water was used as the matrix background, and the net Pb²⁺ response was obtained by subtracting the drinking-water baseline from the drinking water + Pb²⁺ response. As shown in Fig. 5f, g and Supplementary Fig. 33, Pb²⁺ remains distinguishable in drinking water at 10⁻¹⁷ M. The water-quality background of the drinking-water sample is provided in Supplementary Table 1.

The regeneration behavior and storage stability of Au + BP-COOH were also evaluated. The regeneration experiment was performed using 10⁻¹⁸ M Pb²⁺ on the Au + BP-COOH chip shown in Supplementary Fig. 16. As shown in Supplementary Fig. 18, the Pb²⁺ response remains distinguishable after five regeneration cycles. Storage stability was evaluated using 10⁻¹⁵ M Pb²⁺, with the sensing results provided in Supplementary Figs. 19–24 and the corresponding statistical results summarized in Supplementary Fig. 25. The mean Δ*θ* decreases from 1585 mdeg on Day 1 to 557 mdeg on Day 7, corresponding to a 64.9% decrease and retention of 35.1% of the initial response, although the Pb²⁺ response remains distinguishable at all tested time points. Within the broader context of two-dimensional-material-assisted SPR interfaces, this response retention indicates that storage stability remains a limitation of the present Au + BP-COOH interface^22,35^. The decrease may arise from gradual changes in the BP-COOH sensing layer during storage. BP is susceptible to oxidation in the presence of oxygen and adsorbed water, which may alter its surface and optical properties^26,28,36–38^, while changes in nanosheet assembly and the accessibility of oxygen-containing sites may also affect Pb²⁺ interaction and SPR transduction. Oxygen- and moisture-limited storage, optimized polymer passivation, and sealed chip packaging may improve the storage stability of the Au + BP-COOH interface^28,37–39^.

Taken together, Fig. 5 and Table 1 and the supporting results in Supplementary Figs. 5–33 establish the Pb²⁺ sensing performance and selectivity of Au + BP-COOH from five aspects. First, Au + BP-COOH enables ultralow-concentration Pb²⁺ sensing in pure water. Second, the comparison among bare Au, Au + BP, and Au + BP-COOH distinguishes the BP-related optical contribution from the BP-COOH-related Pb²⁺ interaction contribution. Third, the time-dependent statistics of ten independently prepared Au + BP-COOH chips clarify the detection behavior at ultralow Pb²⁺ concentrations. Fourth, the detectable concentration regime for Pb²⁺ remains markedly lower than those of Cd²⁺, Cu²⁺, Zn²⁺, Ni²⁺, Ca²⁺, and Mg²⁺. Fifth, Pb²⁺ can be detected in mixed-ion solution and drinking water after subtracting the corresponding matrix baselines, and Au + BP-COOH retains distinguishable responses after five regeneration cycles and at all tested storage time points, although the storage response decreases with storage time.

### Comparison with representative Pb²⁺ sensing strategies

**Fig. 6:**
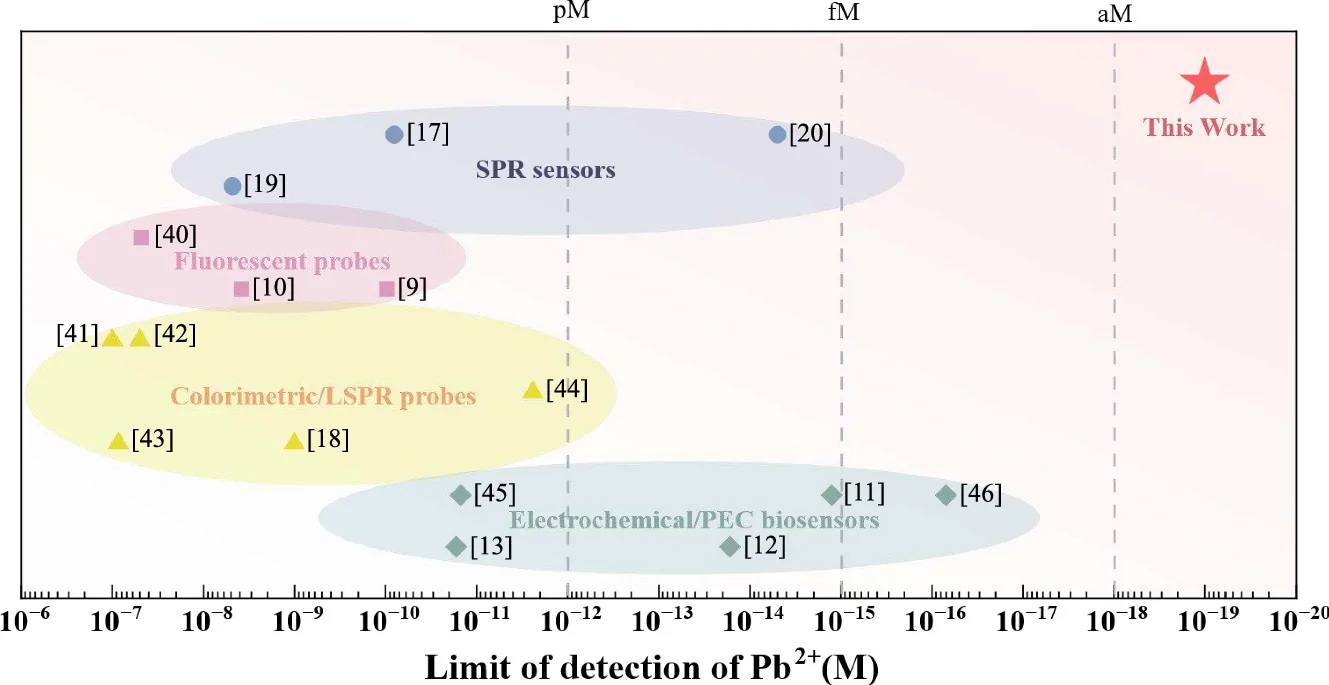
Comparison of the Pb²⁺ limit of detection (LOD) of this work with representative reports from four sensing categories. LOD values from representative SPR sensors, fluorescent probes, colorimetric/localized surface plasmon resonance (LSPR) probes, and electrochemical/photoelectrochemical (PEC) biosensors are plotted on the concentration axis, together with the result of this work. The corresponding references are refs. ^9–13,17–20,40–46^.

To position the present sensing performance in the context of reported Pb²⁺ detection technologies, the LOD of Au + BP-COOH was compared with representative studies from four categories, including SPR sensors, fluorescent probes, colorimetric/LSPR probes, and electrochemical/PEC biosensors, as summarized in Fig. 6. Within the representative SPR studies, previously reported LODs span from 4.8 × 10⁻⁹ to 5.0 × 10⁻¹⁵ M, including 4.8 × 10⁻⁹ M for a fiber-based SPR sensor^19^, 8.0 × 10⁻¹¹ M for a label-free SPR platform based on DNAzyme-gold nanoparticle conjugates^17^, and 5.0 × 10⁻¹⁵ M for an amplified SPR format using a Pb²⁺-dependent DNAzyme and a hemin/G-quadruplex label^20^. Representative fluorescent probes reported LODs of 4.8 × 10⁻⁸, 9.6 × 10⁻¹¹, and 3.8 × 10⁻⁹ M^9,10,40^. Representative colorimetric/LSPR probes reported LODs in the range of 1.0 × 10⁻⁷ to 2.4 × 10⁻^12^ M^18,41–44^. Representative electrochemical and PEC biosensors further reached 1.5 × 10⁻¹¹, 1.67 × 10⁻¹⁴, 1.26 × 10⁻¹⁵, 1.67 × 10⁻¹¹, and 7.0 × 10⁻¹⁷ M^11–13,45,46^.

Against this representative literature background, the LOD of Au + BP-COOH reaches 10⁻¹⁹ M, placing the present work in a distinctly lower concentration regime than the compared SPR, fluorescent, colorimetric/LSPR, and electrochemical/PEC reports shown in Fig. 6. This comparison is notable because the present platform is still based on a prism-coupled Au-film SPR configuration rather than on multistep amplified electrochemical readout. In other words, the performance gain is achieved by direct interfacial engineering of the sensing layer on a traditional SPR platform. Together with the results in Figs. 1–4, this comparison shows that the BP-COOH functional layer enables a substantial extension of the detectable Pb²⁺ concentration regime while retaining the label-free optical readout format of SPR.

## Discussion

In summary, we constructed a surface plasmon resonance sensor modified with carboxyl-functionalized BP (Au + BP-COOH) and demonstrated its ultralow-concentration Pb²⁺ sensing capability on a prism-coupled Au-film SPR platform. The BP-COOH functional layer provides oxygen-containing interfacial sites for Pb²⁺ interaction, while also enhancing the optical response of the SPR interface. This combination is supported by the interfacial characterization, the refractive-index calibration of different Au-based interfaces, and the Pb²⁺ sensing comparison among bare Au, Au + BP, and Au + BP-COOH. As a result, Au + BP-COOH enables distinguishable Pb²⁺ detection down to 10⁻¹⁹ M in pure water, whereas bare Au and Au+BP show distinguishable Pb²⁺ responses from 10⁻⁵ and 10⁻⁷ M, respectively, under the same sensing framework.

In addition to the markedly extended detectable concentration regime, Au + BP-COOH also exhibits a substantially lower response range for Pb²⁺ than for Cd²⁺, Cu²⁺, Zn²⁺, Ni²⁺, Ca²⁺, and Mg²⁺. The time-dependent statistics from ten independently prepared Au + BP-COOH chips further clarify the detection behavior at ultralow Pb²⁺ concentrations, with consistent detectability from 10⁻¹⁸ to 10⁻¹⁵ M and a time-dependent statistical detection regime at 10⁻¹⁹ M. Mixed-ion interference and drinking-water measurements further show distinguishable Pb²⁺ responses in more complex matrix backgrounds. The regeneration and storage-stability tests also show that Au + BP-COOH retains distinguishable Pb²⁺ sensing responses under the tested conditions, although the response decreases during storage. Compared with more stable two-dimensional materials, the storage stability of the present BP-based interface remains comparatively limited, but appropriate storage, passivation, and packaging strategies offer feasible and promising routes for improvement. The preferential response of this system toward Pb²⁺ over the other tested ions may arise from differences in ion–surface interactions, and further investigation into the underlying mechanism represents an important and promising direction for future work. Overall, this work shows that direct interfacial engineering with a BP-COOH functional layer can substantially improve the performance of conventional label-free Au-film SPR for Pb²⁺ detection. These results provide a simple route for extending traditional Au-film SPR toward ultratrace heavy-metal-ion sensing.

## Methods

### Materials

Carboxyl-functionalized BP (BP-COOH) was purchased from Xi’an Ruixi Biological Technology and used as the sensing material. Pb, Cd, Cu, Zn, Ni, Ca, and Mg standard single-element solutions were used for ion sensing experiments. The Pb standard solution was GSB 04-1742-2004 (1000 μg/mL, *c*(HNO₃) = 1.0 mol/L). The Cd, Cu, Zn, Ni, Ca, and Mg standard solutions were GSB 04-1721-2004, GSB 04-1725-2004, GSB 04-1761-2004, GSB 04-1740-2004, GSB 04-1720-2004, and GSB 04-1735-2004, respectively, all with a standard value of 1000 μg/mL in *c*(HNO₃) = 1.0 mol/L medium. Pure water was used for sample preparation and sensing measurements. Unless otherwise specified, all experimental procedures were carried out in a clean laboratory.

### Preparation of BP-COOH

Commercial BP-COOH was obtained from Xi’an Ruixi Biological Technology, and its preparation route is described as follows. BP nanosheets were prepared by liquid-phase exfoliation. Specifically, 5 g NaOH was added into 50 mL anhydrous N-methyl-2-pyrrolidone (NMP) and sonicated in a water bath at 180 W for 30 min to obtain alkalized NMP. Bulk BP crystals were then dispersed into the alkalized NMP solution and ultrasonicated at 500 W in an ice bath using a pulse sequence of 3 s on and 3 s off, while the probe temperature was kept below 40 °C. The sonication was continued for 8–10 h. The resulting dispersion was first centrifuged at 1700 × g at 4 °C for 10 min, and the supernatant was collected. The collected supernatant was then centrifuged at 18,500 × g at 4 °C for 15–20 min, and the precipitate was collected as BP nanosheets.

For carboxyl functionalization, the concentration of the BP nanosheet dispersion was adjusted to 1 mg/mL. Then, 2.5 mg PEI aqueous solution (500 μL) was added, and the mixture was stirred at 500 rpm in a brown bottle under light protection overnight to obtain amino-functionalized BP nanosheets. Subsequently, 2 mL poly(acrylic acid) (PAA, molecular weight 5000, 50%) was adjusted to approximately pH 7.0 by adding a 5 M NaOH aqueous solution stepwise under pH-meter monitoring, and then added dropwise into the above dispersion under magnetic stirring. The mixture was further stirred at room temperature for 6 h, followed by centrifugation, washing with water 2–3 times, and redispersion in deionized water to obtain BP-COOH. A freeze-drying protectant was then added at 20 times the mass of the BP nanosheets. The resulting dispersion was frozen at −80 °C for 24 h and then freeze-dried for 72 h.

### Fabrication of Au + BP-COOH

BP-COOH was stored at −20 °C under light protection before use. For sensing experiments, 0.0001 g BP-COOH was dispersed in 1 mL pure water in the clean laboratory and sonicated in an ice-water bath for 480 s. The dispersion was freshly prepared before use.

The SPR substrate consisted of a 50 nm Au film with a 5 nm Cr adhesion layer. Before use, the Au-coated chip was rinsed once with ethanol, once with pure water, and once again with ethanol. The cleaned Au chip without BP-COOH deposition was used as bare Au. For fabrication of Au + BP-COOH, 0.5 mL of the freshly prepared BP-COOH dispersion was drop-cast onto the Au film and dried naturally at 22 °C in the clean laboratory. The Au + BP control chips were prepared using the same drop-casting and drying procedure, except that the BP dispersion was used instead of the BP-COOH dispersion. The representative Au + BP-COOH structure used in the main-text sensing experiments had a thickness of approximately 13 nm. Additional independently prepared Au + BP-COOH chips were characterized by AFM, as shown in Supplementary Fig. 2. During assembly of the SPR device, cedar wood oil (MACKLIN, C805296) was used as the refractive-index-matching medium between the Au chip and the BK7 prism.

### SPR measurements

SPR measurements were performed on a Dynechem SPR system using 650 nm incident light at 22 °C. The angular scanning range was 65°–80°, and the angular step was 19–21 mdeg. For each measurement, 400 μL of sample solution was introduced into the flow cell at a flow rate of 200 μL/min. The resonance angle was directly read from the angle corresponding to the minimum of the reflected-intensity curve without curve fitting.

For Pb²⁺ time-dependent sensing measurements, the SPR response was recorded at 30, 60, 90, and 120 s. Unless otherwise specified, the resonance-angle shift Δ*θ* used for statistical comparison was extracted at 120 s. For pure-water sensing measurements and single-ion sensing measurements, Δ*θ* was defined relative to the corresponding pure-water baseline. For mixed-ion and drinking-water measurements, the net Δ*θ* was obtained by subtracting the corresponding matrix baseline.

For the time-dependent Pb²⁺ detection statistics, ten independently prepared Au + BP-COOH chips were measured from pure water to increasing Pb²⁺ concentrations. For single-ion selectivity, mixed-ion interference, and drinking-water measurements, three independently prepared Au + BP-COOH chips were used unless otherwise specified. Error bars represent the standard deviation from independently prepared chips.

### Preparation of ion solutions

All ion solutions were prepared in the clean laboratory. The purchased single-element standard solutions were first converted to 10⁻² M stock solutions and then diluted stepwise by a factor of 10 using pure water to obtain the required concentration series. The pH of the test solutions remained at 6.3, and all ions were measured under the same matrix conditions. For each target concentration and each dilution step, a fresh container and a fresh pipette tip were used to minimize carryover between concentrations. Containers and pipette tips were not reused, and the ultralow-concentration solutions were freshly prepared before measurement.

### Definition of distinguishable response and LOD

The resonance-angle shift Δ*θ* was used to evaluate the sensing response. A response was considered distinguishable when Δ*θ* exceeded 21 mdeg. This operational distinguishability threshold was selected according to the angular scanning step of the SPR measurement and the baseline stability of Au + BP-COOH in pure water. Repeated pure-water measurements on the same Au + BP-COOH chip over 50 min showed a maximum resonance-angle drift of 21 mdeg and a standard deviation of 0.00714 deg (Supplementary Fig. 34). The LOD was defined as the lowest concentration at which a distinguishable response was obtained under the corresponding sensing condition.

### Mixed-ion interference and drinking-water measurements

For the mixed-ion interference experiment, a mixed-ion solution was used as the matrix background. The Mix solution contained Cd²⁺, Cu²⁺, Zn²⁺, Ni²⁺, Ca²⁺, and Mg²⁺ at 10⁻¹³, 10⁻¹³, 10⁻¹², 10⁻¹⁰, 10⁻⁹, and 10⁻⁸ M, respectively. The Mix solution was first measured as the matrix baseline. Then, Pb²⁺ was added to the Mix solution to obtain Mix + 10⁻¹⁶ M Pb²⁺ and Mix + 10⁻¹⁵ M Pb²⁺. The net Pb²⁺ response was calculated by subtracting the Mix baseline from the Mix + Pb²⁺ response.

Drinking water was used as the real-sample matrix for Pb²⁺ sensing. The water-quality background of the drinking-water sample is provided in Supplementary Table 1. During measurement, drinking water was first measured as the matrix baseline. Then, Pb²⁺ was added to drinking water to obtain drinking water + 10⁻¹⁷ M Pb²⁺, drinking water + 10⁻¹⁶ M Pb²⁺, and drinking water + 10⁻¹⁵ M Pb²⁺. The net Pb²⁺ response was calculated by subtracting the drinking-water baseline from the drinking water + Pb²⁺ response.

### Storage stability and regeneration measurements

The storage stability of Au + BP-COOH chips was evaluated using 10⁻¹⁵ M Pb²⁺ as a representative concentration. Day 1 corresponds to freshly prepared Au + BP-COOH chips measured immediately after fabrication. Days 2–7 correspond to Au + BP-COOH chips measured after 1, 2, 3, 4, 5, and 6 days of storage, respectively. Before measurement, the fabricated Au + BP-COOH chips were stored for the specified periods at 22 °C and 50% relative humidity under light-protected conditions. For Day 1, the statistical response was obtained from ten independently prepared Au + BP-COOH chips measured at 120 s. For Days 2–7, three independently prepared Au + BP-COOH chips were measured at each storage time point. At each time point, pure water was first measured as the baseline, followed by 10⁻¹⁵ M Pb²⁺. The storage-stability response was defined as the net resonance-angle shift after subtracting the pure-water baseline.

For regeneration measurements, the Au + BP-COOH chip shown in Supplementary Fig. 16 was used, and 10⁻¹⁸ M Pb²⁺ was selected for the regeneration test. After each Pb²⁺ sensing cycle, the Au + BP-COOH chip was treated with 10 mM EDTA solution, followed by rinsing with pure water until the baseline became stable. The regenerated chip was then used for the next Pb²⁺ sensing cycle. Cycle 0 denotes the measurement before regeneration, whereas Cycles 1–5 denote measurements after each regeneration treatment.

### Characterization

AFM measurements were performed on a Bruker Dimension Icon instrument in ScanAsyst mode using a SCANASYST-AIR probe. The probe was made of SiN and had a resonance frequency of 70 kHz, a tip height of 2.5–8 μm, a typical curvature radius of 2 nm, a cantilever length of 115 μm, a cantilever thickness of 0.65 μm, a width of 35 μm, and a spring constant of 0.4 N/m. The scanning frequency was 1 Hz.

Raman spectra of BP-COOH were recorded from Day 1 to Day 7 to evaluate the structural retention of BP-COOH during storage.

UPS measurements were performed using He I radiation with a photon energy of 21.22 eV under a −5 V sample bias, with a pass energy of 2.0 eV and an energy step of 0.020 eV. The Fermi-level reference was calibrated using an Ag standard. The work functions of bare Au, BP, and BP-COOH are provided in Supplementary Fig. 4.

XPS measurements were performed on a ThermoFisher K-Alpha+ system using an Al Kα X-ray source. The pass energy was 100 eV for survey spectra and 50 eV for high-resolution spectra. The chamber pressure was 4.1 × 10⁻⁹ mbar before analysis and 3.9 × 10⁻⁷ mbar during analysis. Charge correction was referenced to C 1*s* at 284.8 eV. Background subtraction was performed using the smart method.

STEM measurements were performed on a JEOL JEM-F200 transmission electron microscope operated at 200 kV. The instrument was used at a high-resolution magnification up to 1.5 million. EDS analysis was performed using a JEOL JED-2300T system.

### Simulation

The optical response of the Au-film structure with and without a BP overlayer was calculated by the transfer matrix method^47^, and the results are shown in Supplementary Fig. 3. The incident wavelength was set to 633 nm. The refractive index of the BK7 prism was taken as 1.5151^29,48^. The thickness of a single BP layer was taken as 0.53 nm, and its refractive index was taken as 3.5 + 0.01i^29,49^. The Au thickness was 50 nm, and its refractive index was calculated by the Drude model as 0.1378 + 3.6195i^50^. The angular sensitivity was defined as the resonance-angle change divided by the refractive-index change.

## Supplementary information

Supplementary Information

Transparent Peer Review File

## Source data

Source Data

## Data Availability

The Source Data underlying the figures of this study are available with the paper. All raw data generated during the current study are available from the corresponding authors upon request. Source data are provided with this paper.

## Code availability

The code used within the study is available from the corresponding author upon request.
