# Supplementary material for: Built-in electric field-boosted attomolar Pb²⁺ sensing on carboxylated black phosphorene
Source: Nat Commun. 2026 Sep 14;17:10772. doi: 10.1038/s41467-026-77793-6 (PMC13661523; doi:10.1038/s41467-026-77793-6)
Supplement: Supplementary file 1 [file 41467_2026_77793_MOESM1_ESM.pdf]

Supporting Information

Built-in electric field-boosted attomolar Pb^{2+} sensing on carboxylated black phosphorene

Fan Yang[1, 2, 3, †], Xixi Yuan[4, †], Jiaqi Zhu[1, 2, 3], Yuanyuan Pan[5], Zhongjun Li[6], Jianqing Li[5], Yanqi Ge[7], Leiming Wu[1, 2, 3, *], Yuanjiang Xiang[8, *], Han Zhang[7, *], Jun Yang[1, 2, 3, *], Yuwen Qin[1, 2, 3]

[1]*Institute of Advanced Photonics Technology, School of Information Engineering, Guangdong University of Technology, Guangzhou, China*
[2]*Key Laboratory of Photonic Technology for Integrated Sensing and Communication (Guangdong University of Technology), Ministry of Education of China, Guangzhou, China*
[3]*Guangdong Provincial Key Laboratory of Information Photonics Technology, Guangdong University of Technology, Guangzhou, 510006, China*
[4]*School of Computer Science and Technology, Guangdong University of Technology, Guangzhou, China*
[5]*School of Computer Science and Engineering, Macau University of Science and Technology, Macau, China*
[6]*Institute of Systems Engineering, Macau University of Science and Technology, Macau, China*
[7]*State Key Laboratory of Radio Frequency Heterogeneous Integration, College of Physics and Optoelectronic Engineering, Shenzhen University, Shenzhen, China*
[8]*College of Electrical and Electronic Engineering, Wenzhou University, Wenzhou, China*
[†]*These authors contributed equally: Fan Yang and Xixi Yuan*
**Corresponding authors: leiming_wu@gdut.edu.cn; xiangyuanjiang@126.com; hzhang@szu.edu.cn; yangj@gdut.edu.cn*

Supplementary Figures

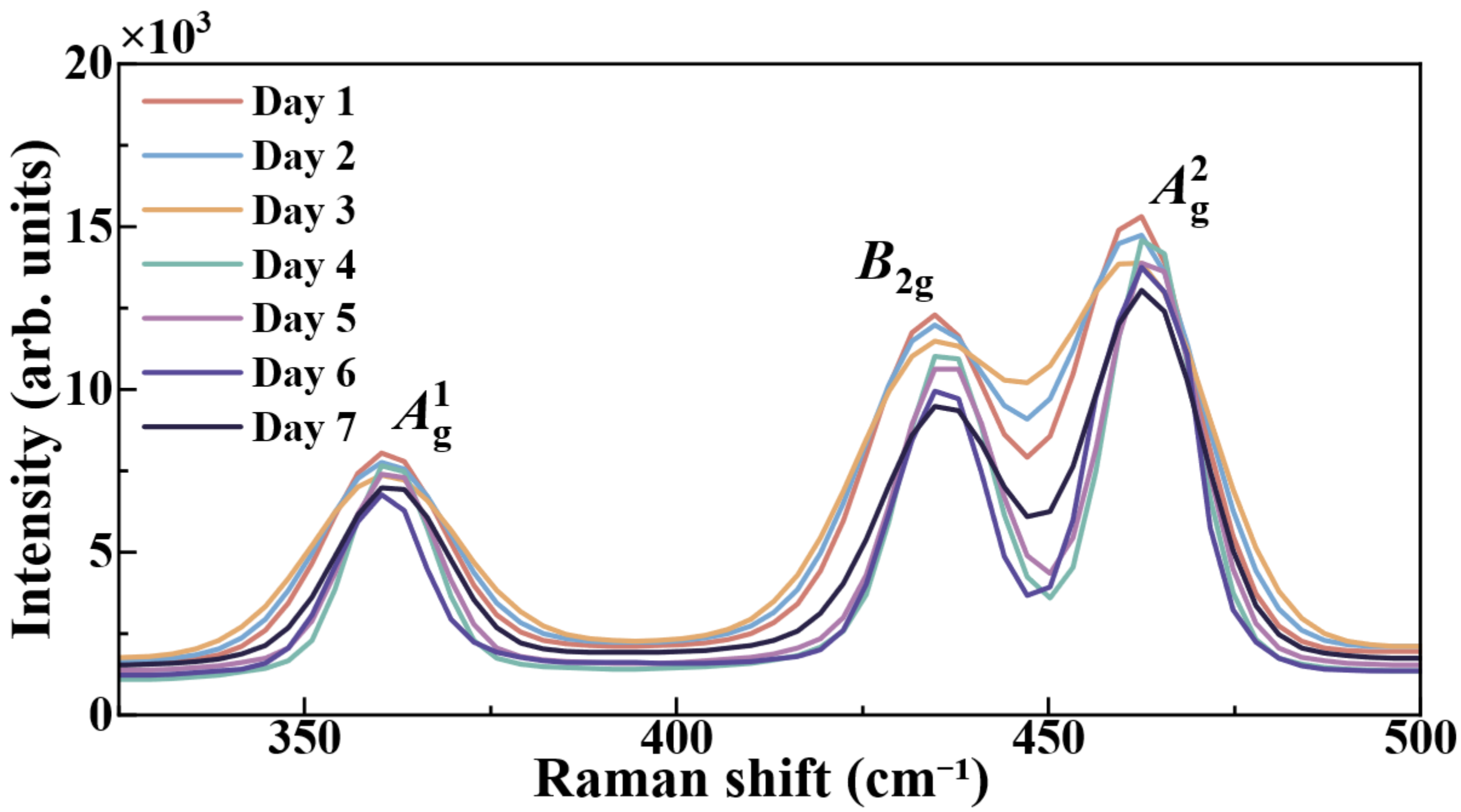

Supplementary Figure 1. Raman spectra of BP-COOH during storage from Day 1 to Day 7. Day 1 corresponds to freshly prepared BP-COOH, and Days 2–7 correspond to BP-COOH stored for 1–6 days, respectively.

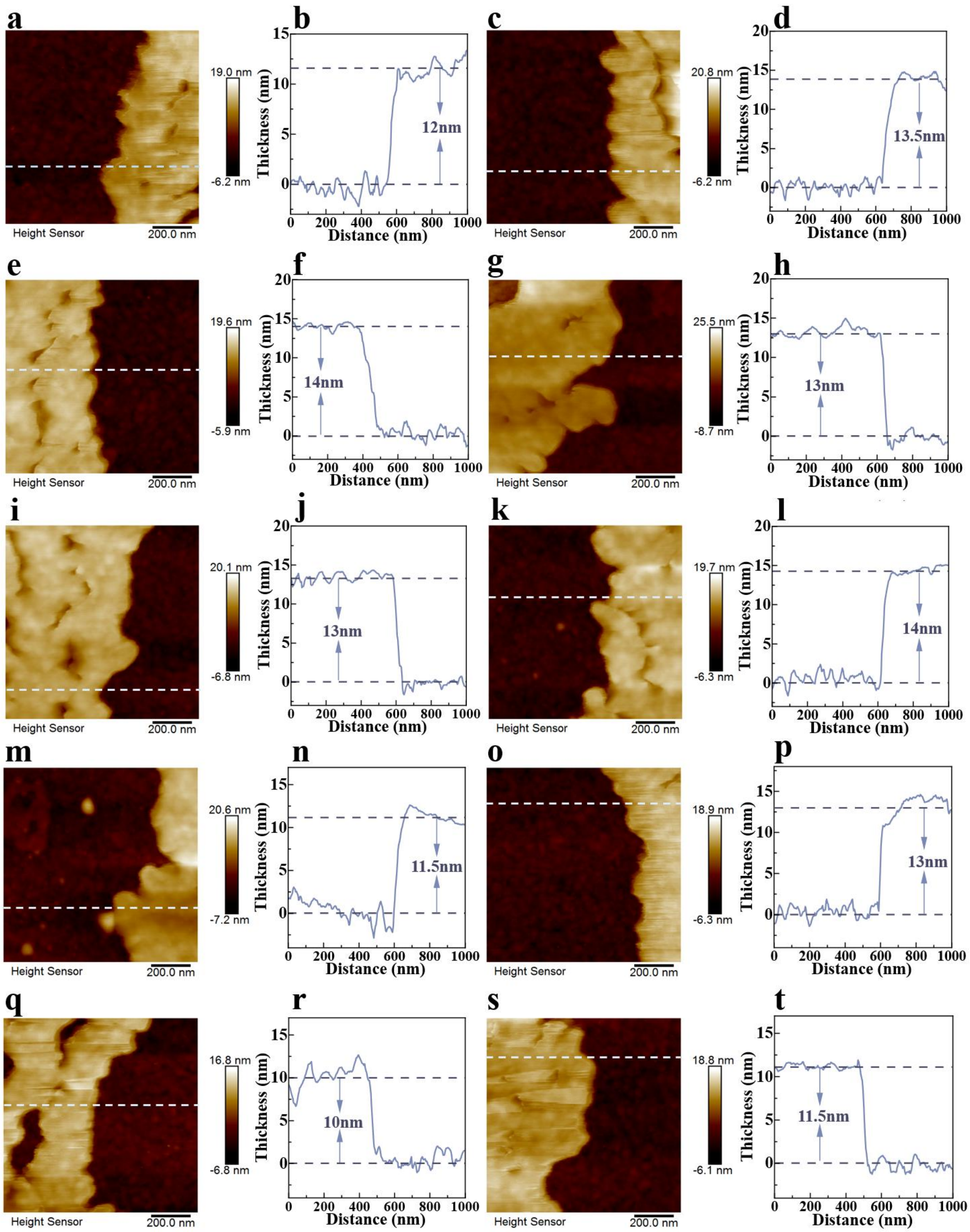

Supplementary Figure 2. AFM characterization of independently prepared Au+BP-COOH chips. (a, c, e, g, i, k, m, o, q, s) AFM images of BP-COOH functional layers on Au films. **(b, d, f, h, j, l, n, p, r, t)** Height profiles extracted from the dashed lines in a, c, e, g, i, k, m, o, q and s, respectively.

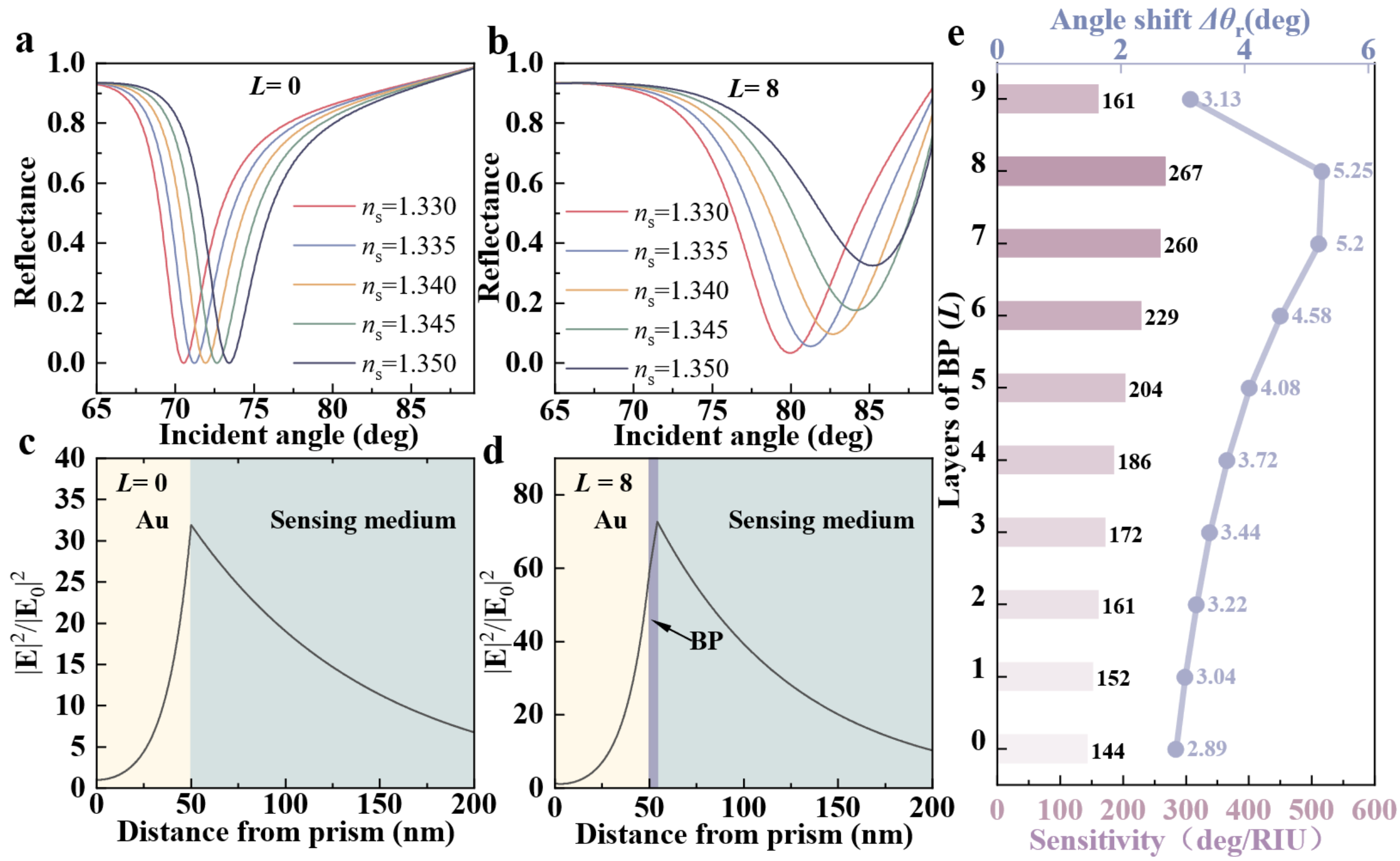

Supplementary Figure 3. Simulated angular SPR response, electric-field distribution, and layer-number-dependent sensitivity of the Au-film SPR structure with and without a BP overlayer. (a) Simulated angle-scanned reflectance curves of bare Au with $L = 0$ at sensing-medium refractive indices n_s = 1.330, 1.335, 1.340, 1.345 and 1.350. **(b)** Simulated angle-scanned reflectance curves of the BP-modified Au structure with $L = 8$ at sensing-medium refractive indices n_s = 1.330, 1.335, 1.340, 1.345 and 1.350. **(c)** Normalized electric-field intensity distribution of bare Au with $L = 0$. **(d)** Normalized electric-field intensity distribution of the BP-modified Au structure with $L = 8$. **(e)** Simulated angular sensitivity and total resonance-angle shift as a function of the number of BP layers.

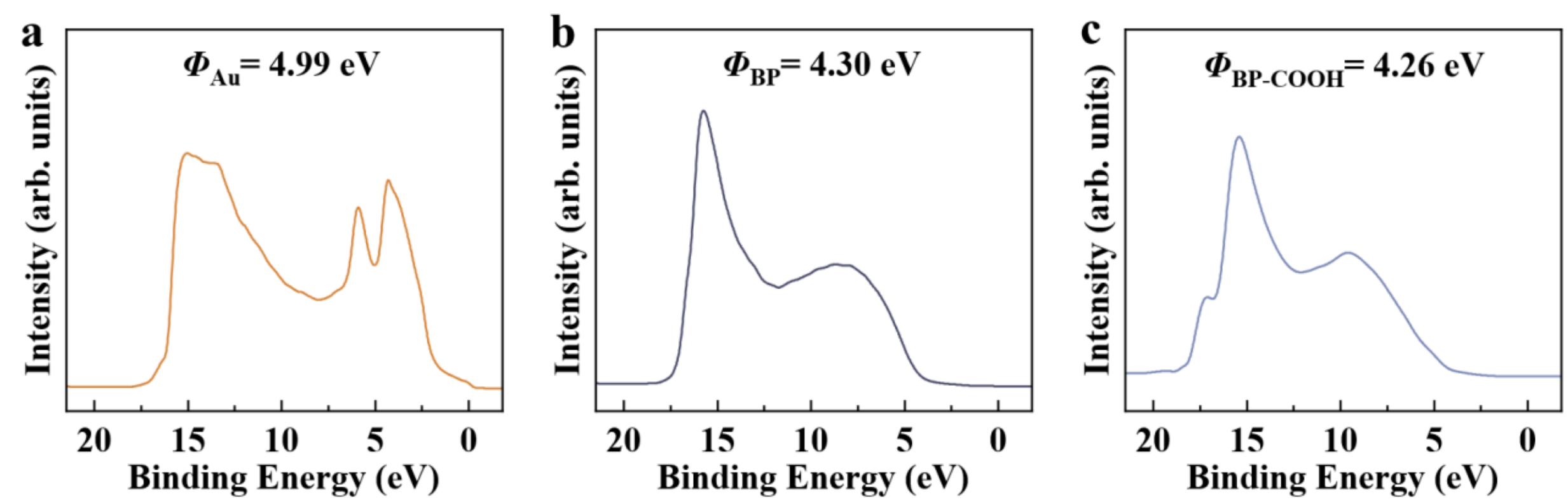

Supplementary Figure 4. UPS work-function spectra of bare Au, BP and BP-COOH. (a–c) UPS spectra of bare Au, BP and BP-COOH, respectively, recorded using He I radiation with a photon energy of 21.22 eV under a −5 V sample bias. The calibrated work functions are 4.99 eV for bare Au, 4.30 eV for BP and 4.26 eV for BP-COOH.

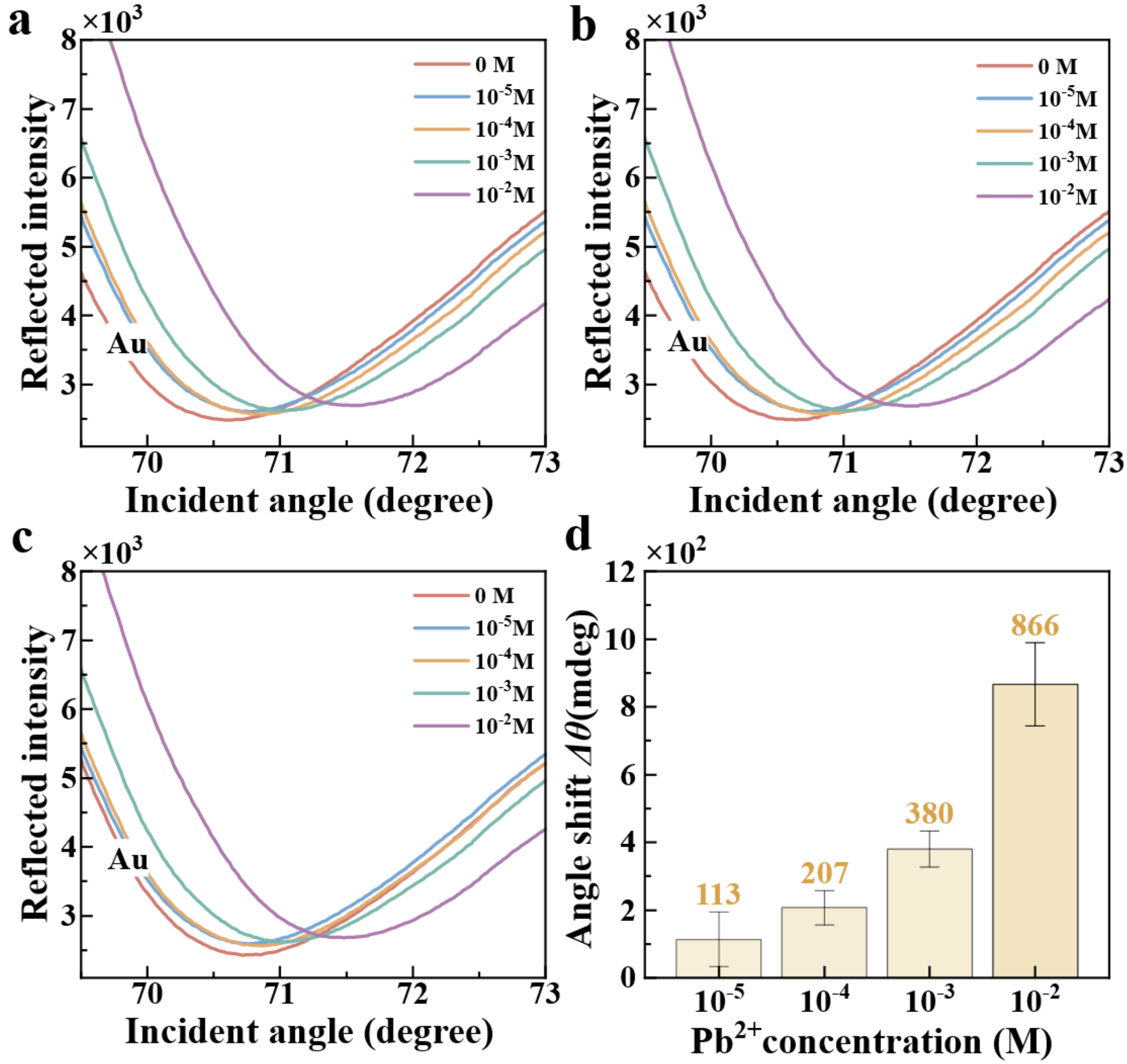

Supplementary Figure 5. Pb^{2+} sensing results obtained from bare Au. (a–c) Angle-scanned reflected-intensity curves of three independently prepared bare Au chips measured for Pb^{2+} concentrations of 0, 10^{-5}, 10^{-4}, 10^{-3} and 10^{-2} M. **(d)** Resonance-angle shift $\Delta\theta$ as a function of Pb^{2+} concentration for bare Au. Bars show mean values, and error bars represent the standard deviation of measurements from three independently prepared bare Au chips.

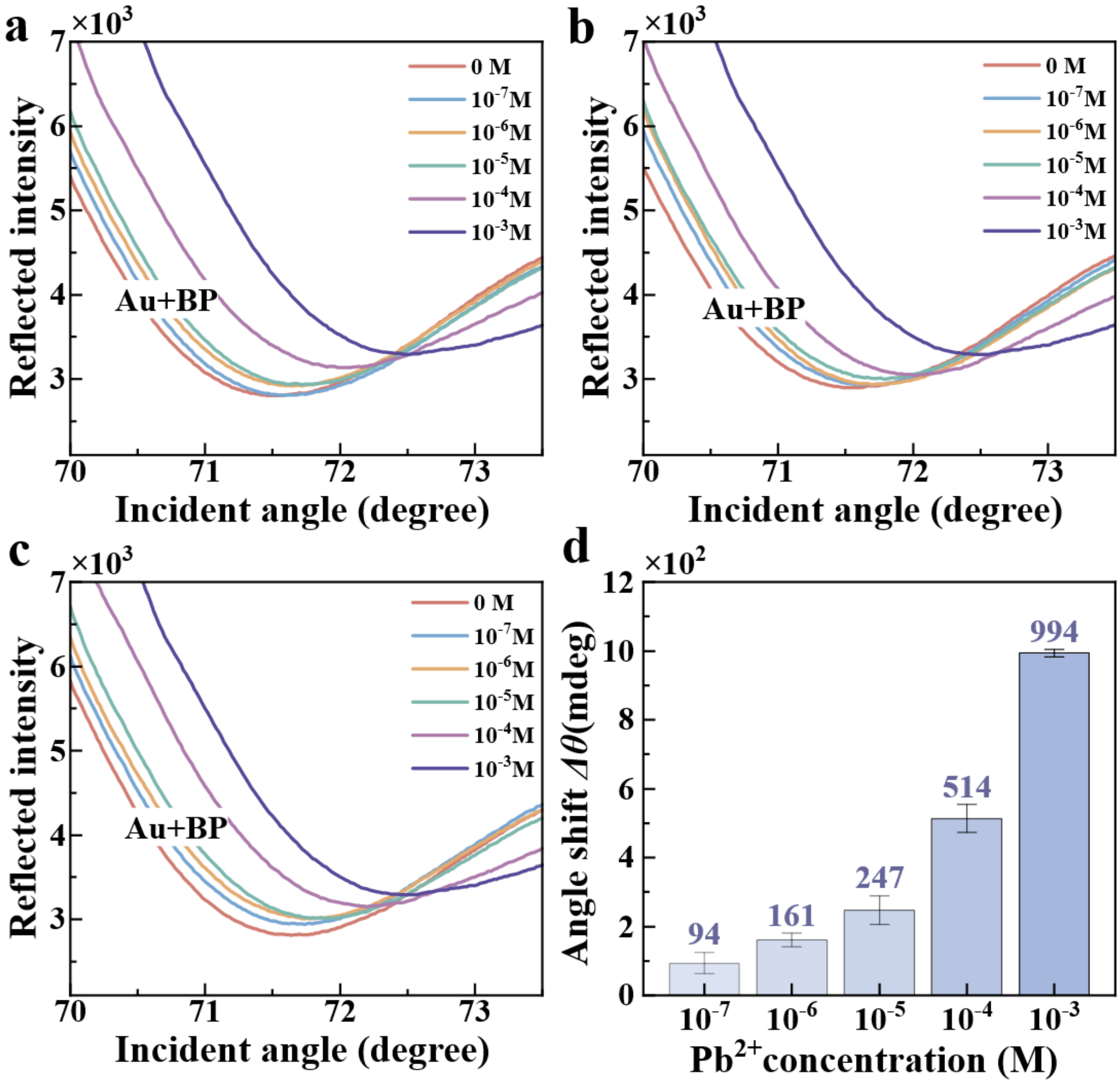

Supplementary Figure 6. Pb^{2+} sensing results obtained from Au+BP. (a–c) Angle-scanned reflected-intensity curves of three independently prepared Au+BP chips measured for Pb^{2+} concentrations of 0, 10^{-7}, 10^{-6}, 10^{-5}, 10^{-4} and 10^{-3} M. **(d)** Resonance-angle shift $\Delta\theta$ as a function of Pb^{2+} concentration for Au+BP. Bars show mean values, and error bars represent the standard deviation of measurements from three independently prepared Au+BP chips.

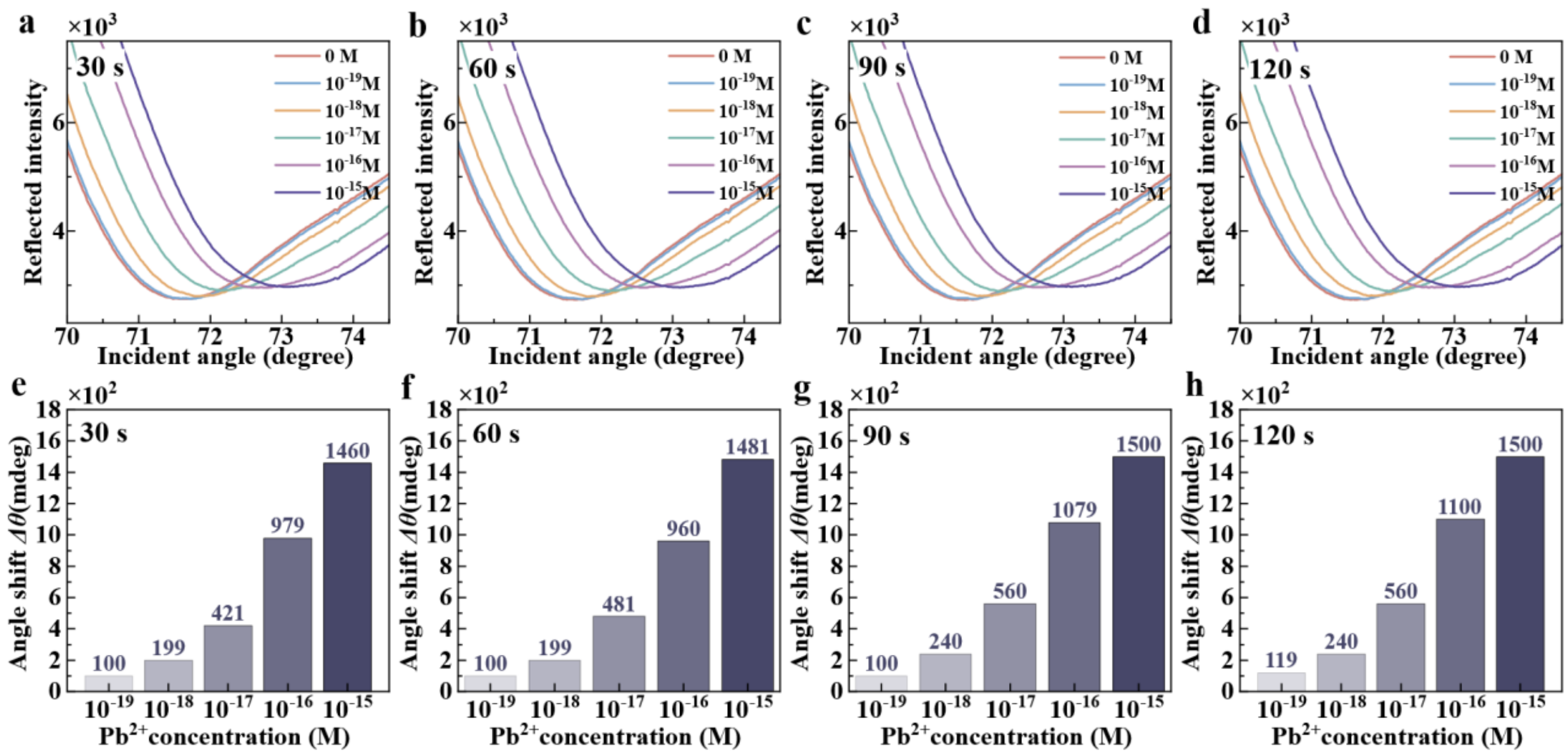

Supplementary Figure 7. Time-dependent Pb^{2+} sensing results obtained from one Au+BP-COOH chip. (a–d) Angle-scanned reflected-intensity curves measured for Pb^{2+} concentrations of 0, 10^{-19}, 10^{-18}, 10^{-17}, 10^{-16} and 10^{-15} M at 30, 60, 90 and 120 s, respectively. **(e–h)** Corresponding resonance-angle shift $\Delta\theta$ values at 30, 60, 90 and 120 s, respectively.

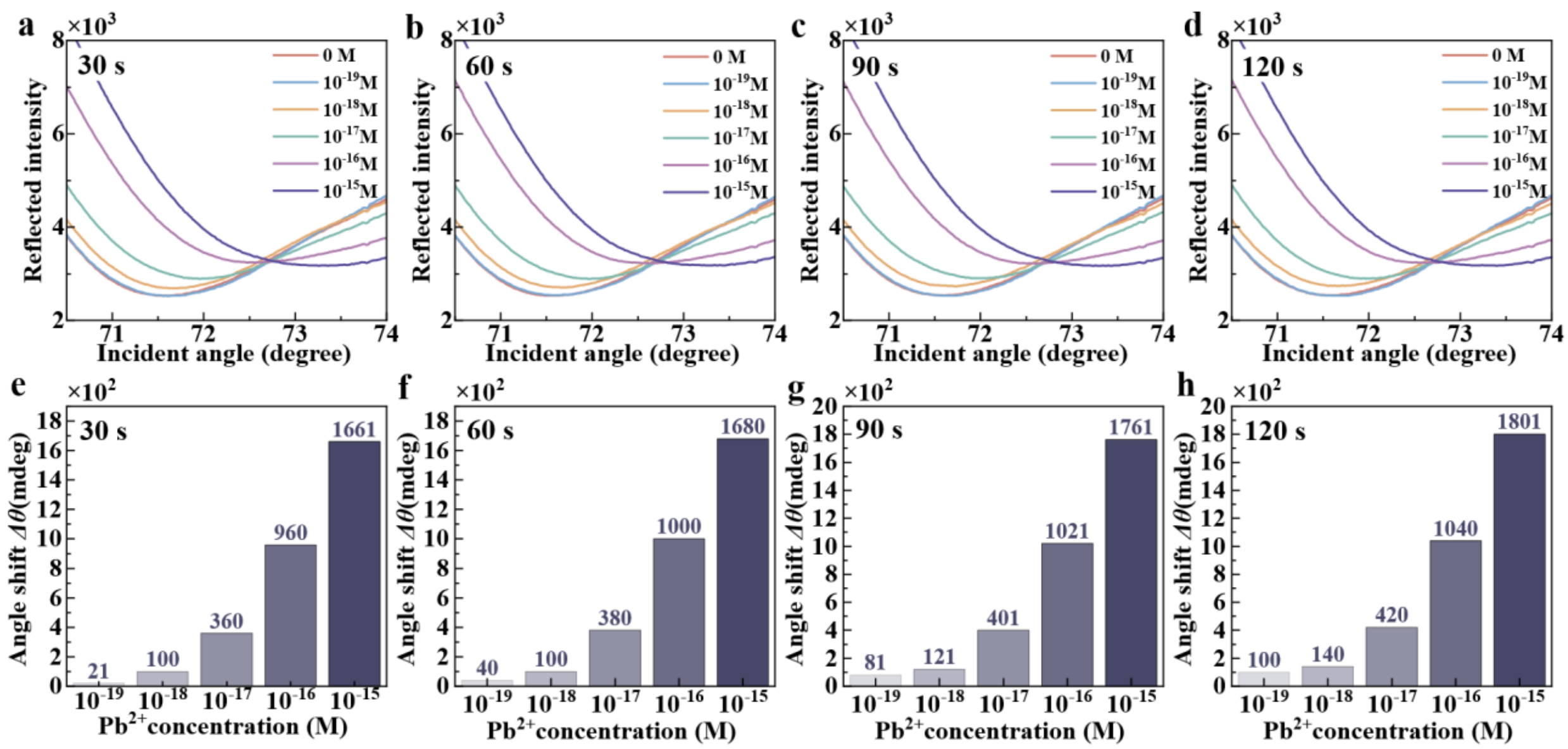

Supplementary Figure 8. Time-dependent Pb^{2+} sensing results obtained from one Au+BP-COOH chip. (a–d) Angle-scanned reflected-intensity curves measured for Pb^{2+} concentrations of 0, 10^{-19}, 10^{-18}, 10^{-17}, 10^{-16} and 10^{-15} M at 30, 60, 90 and 120 s, respectively. **(e–h)** Corresponding resonance-angle shift $\Delta\theta$ values at 30, 60, 90 and 120 s, respectively.

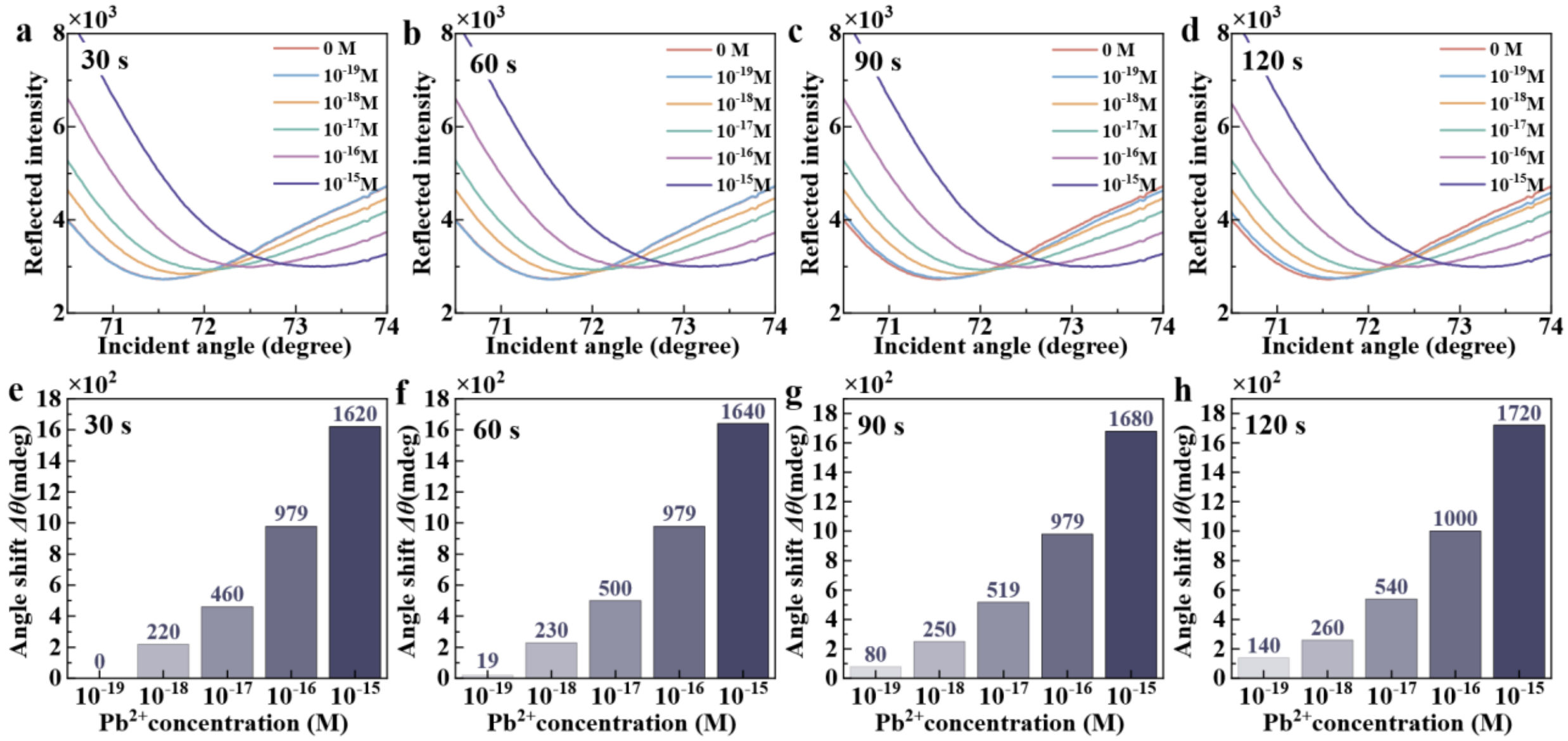

Supplementary Figure 9. Time-dependent Pb^{2+} sensing results obtained from one Au+BP-COOH chip. (a–d) Angle-scanned reflected-intensity curves measured for Pb^{2+} concentrations of 0, 10^{-19}, 10^{-18}, 10^{-17}, 10^{-16} and 10^{-15} M at 30, 60, 90 and 120 s, respectively. **(e–h)** Corresponding resonance-angle shift $\Delta\theta$ values at 30, 60, 90 and 120 s, respectively.

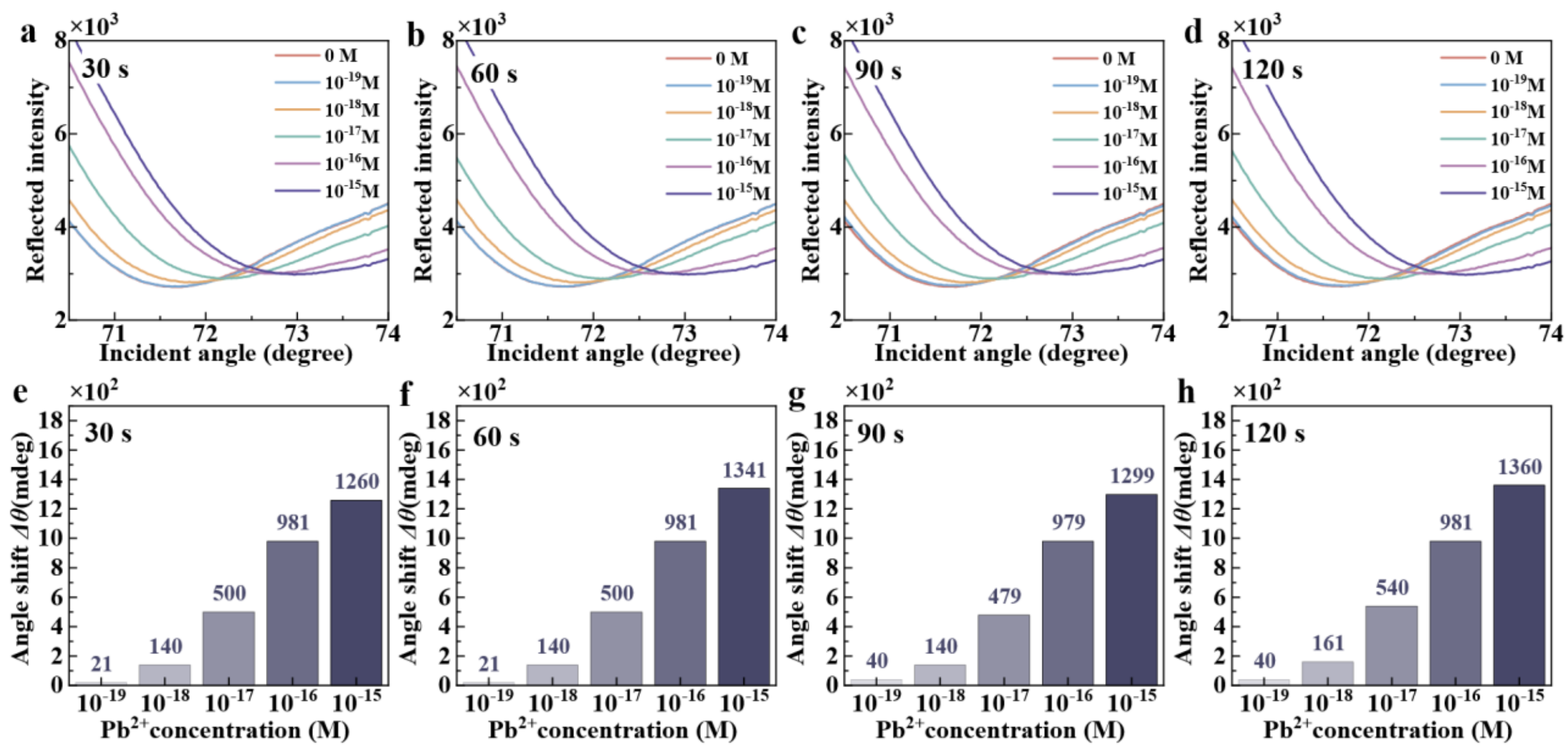

Supplementary Figure 10. Time-dependent Pb^{2+} sensing results obtained from one Au+BP-COOH chip. (a–d) Angle-scanned reflected-intensity curves measured for Pb^{2+} concentrations of 0, 10^{-19}, 10^{-18}, 10^{-17}, 10^{-16} and 10^{-15} M at 30, 60, 90 and 120 s, respectively. **(e–h)** Corresponding resonance-angle shift $\Delta\theta$ values at 30, 60, 90 and 120 s, respectively.

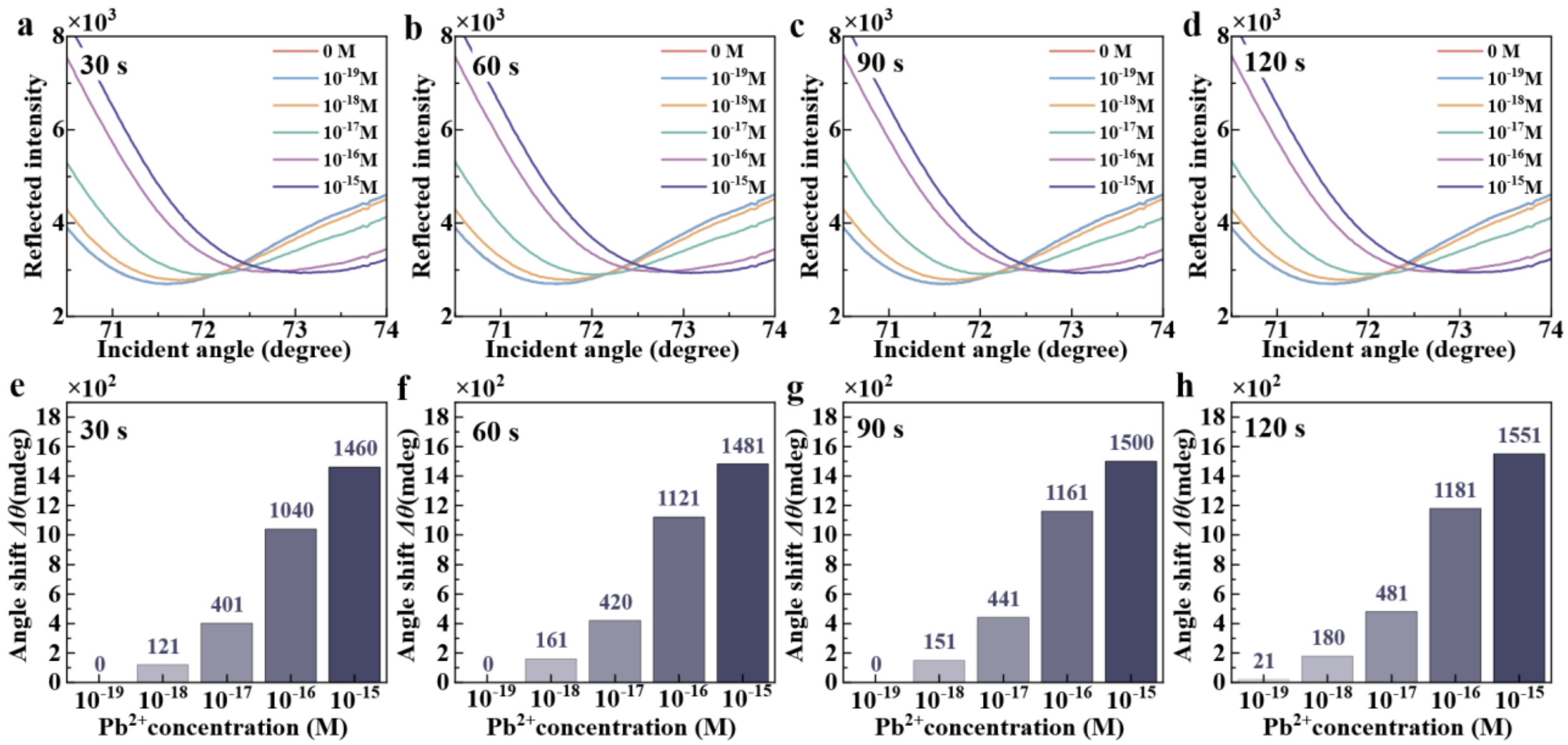

Supplementary Figure 11. Time-dependent Pb^{2+} sensing results obtained from one Au+BP-COOH chip. (a–d) Angle-scanned reflected-intensity curves measured for Pb^{2+} concentrations of 0, 10^{-19}, 10^{-18}, 10^{-17}, 10^{-16} and 10^{-15} M at 30, 60, 90 and 120 s, respectively. **(e–h)** Corresponding resonance-angle shift $\Delta\theta$ values at 30, 60, 90 and 120 s, respectively.

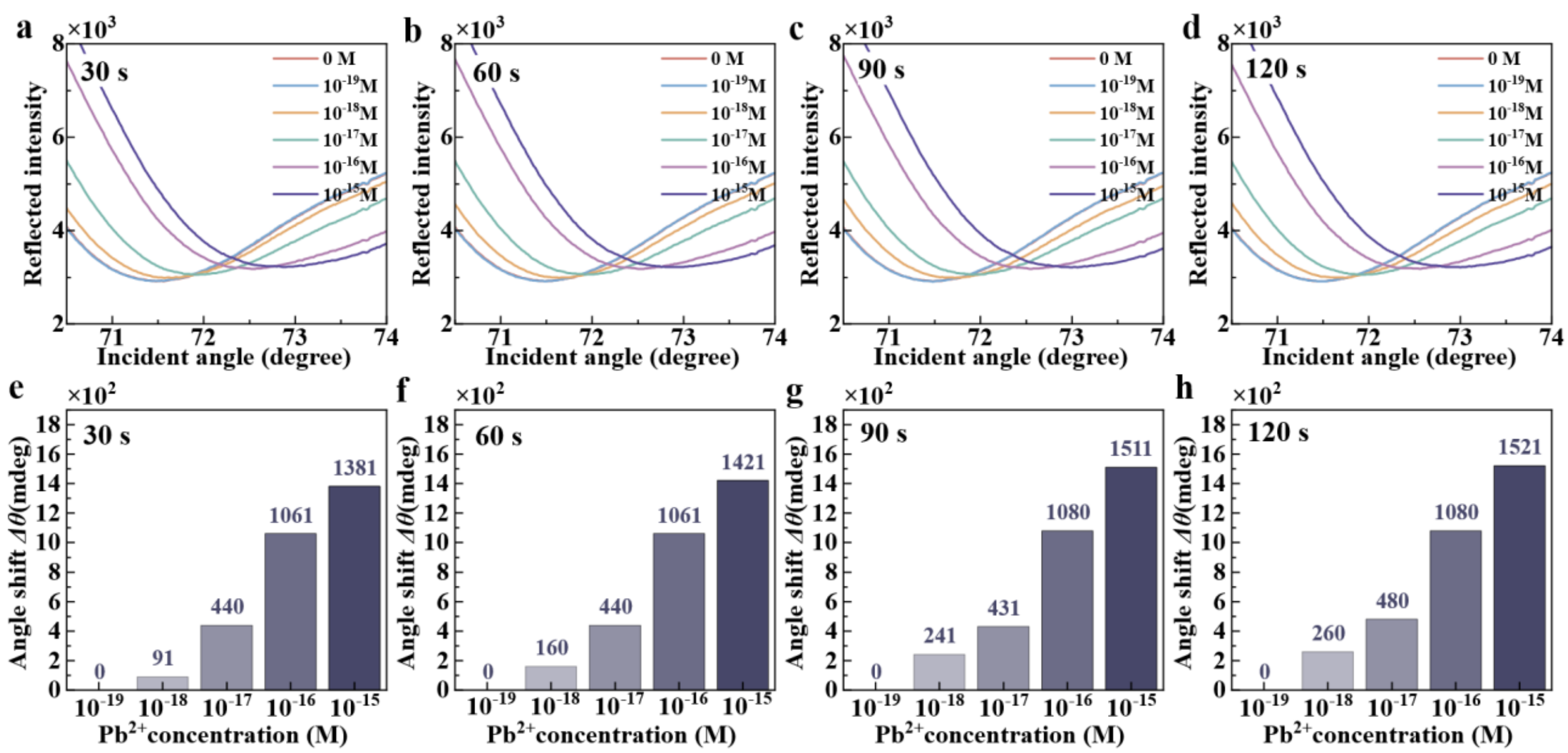

Supplementary Figure 12. Time-dependent Pb^{2+} sensing results obtained from one Au+BP-COOH chip. (a–d) Angle-scanned reflected-intensity curves measured for Pb^{2+} concentrations of 0, 10^{-19}, 10^{-18}, 10^{-17}, 10^{-16} and 10^{-15} M at 30, 60, 90 and 120 s, respectively. **(e–h)** Corresponding resonance-angle shift $\Delta\theta$ values at 30, 60, 90 and 120 s, respectively.

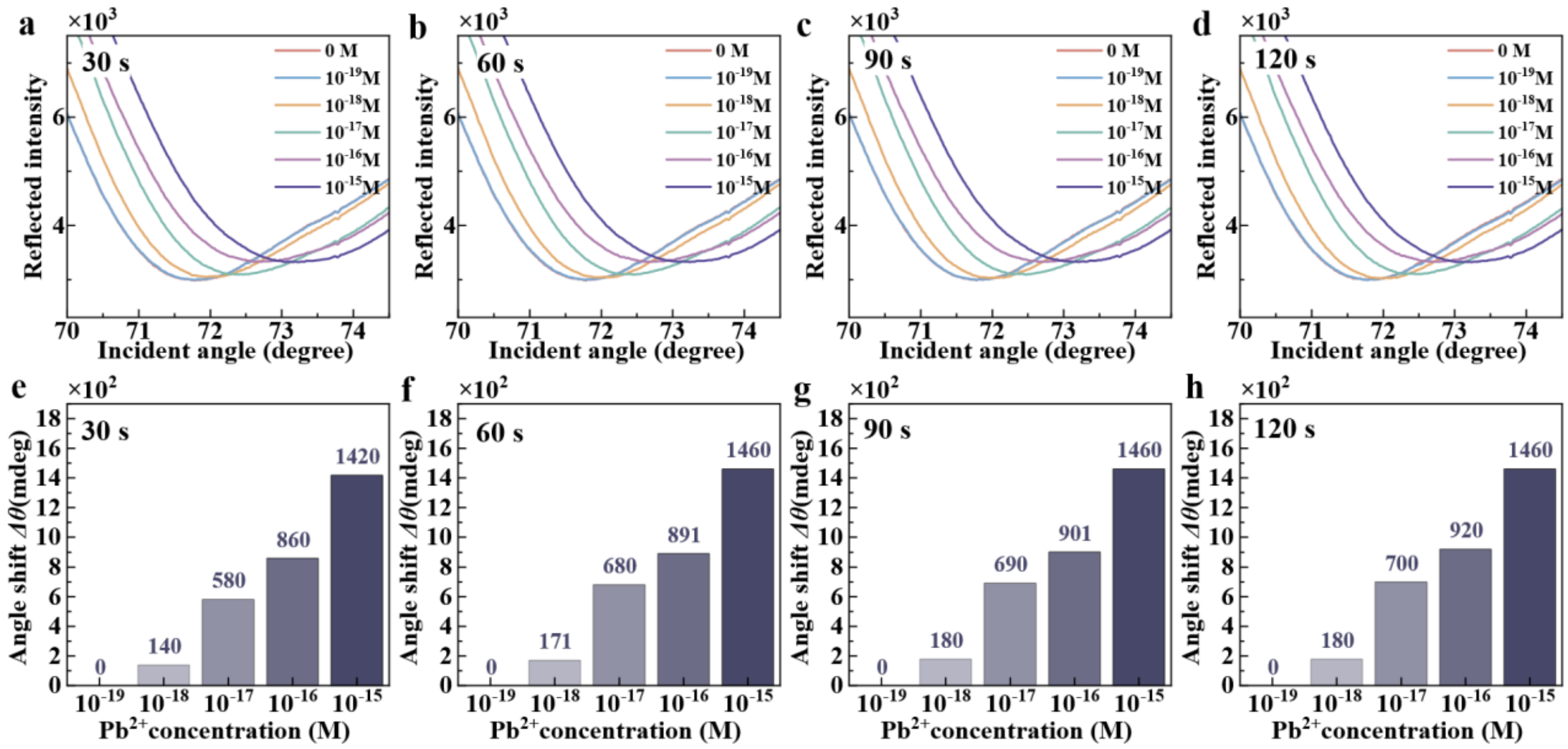

Supplementary Figure 13. Time-dependent Pb^{2+} sensing results obtained from one Au+BP-COOH chip. (a–d) Angle-scanned reflected-intensity curves measured for Pb^{2+} concentrations of 0, 10^{-19}, 10^{-18}, 10^{-17}, 10^{-16} and 10^{-15} M at 30, 60, 90 and 120 s, respectively. **(e–h)** Corresponding resonance-angle shift $\Delta\theta$ values at 30, 60, 90 and 120 s, respectively.

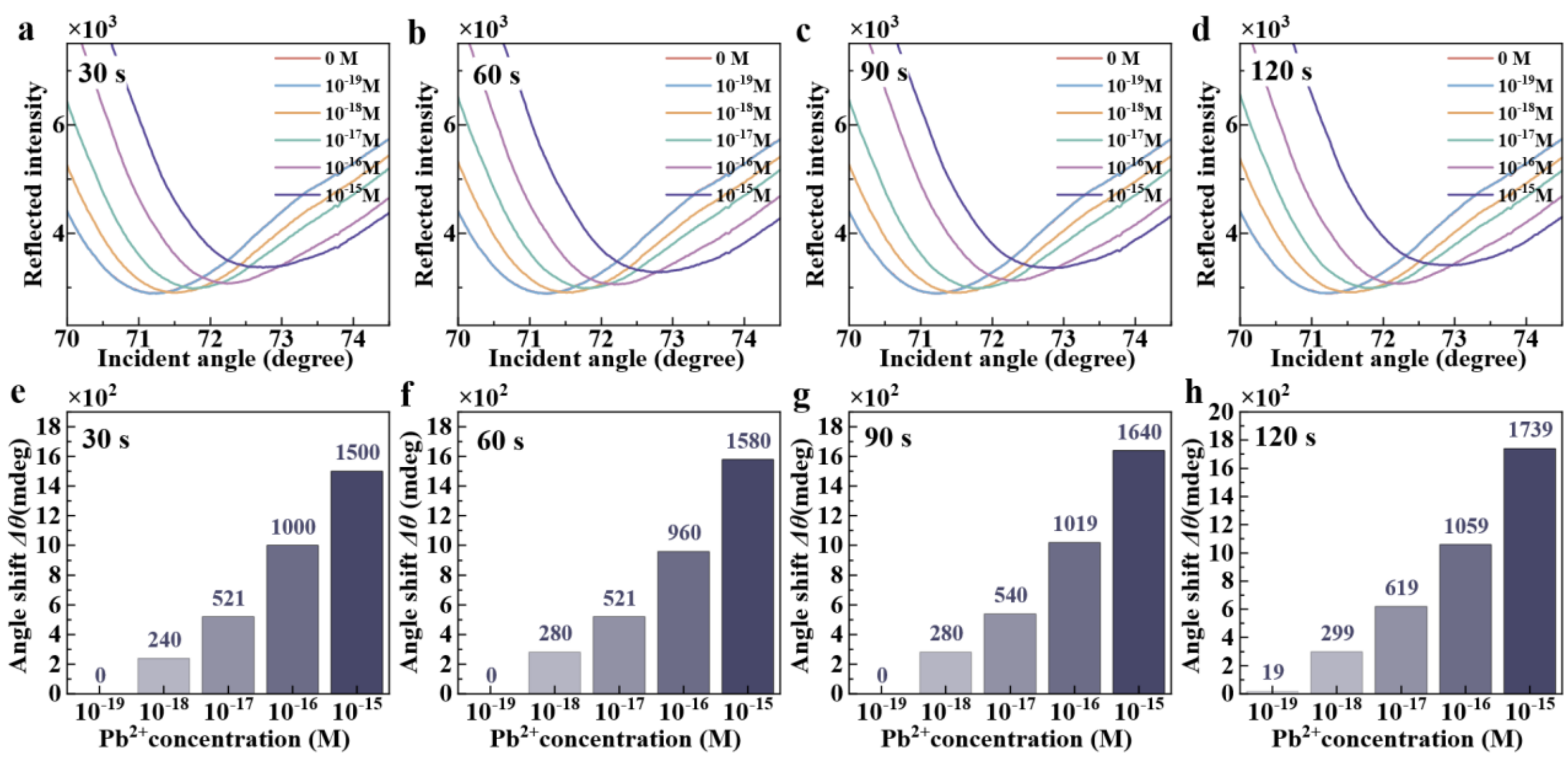

Supplementary Figure 14. Time-dependent Pb^{2+} sensing results obtained from one Au+BP-COOH chip. (a–d) Angle-scanned reflected-intensity curves measured for Pb^{2+} concentrations of 0, 10^{-19}, 10^{-18}, 10^{-17}, 10^{-16} and 10^{-15} M at 30, 60, 90 and 120 s, respectively. **(e–h)** Corresponding resonance-angle shift $\Delta\theta$ values at 30, 60, 90 and 120 s, respectively.

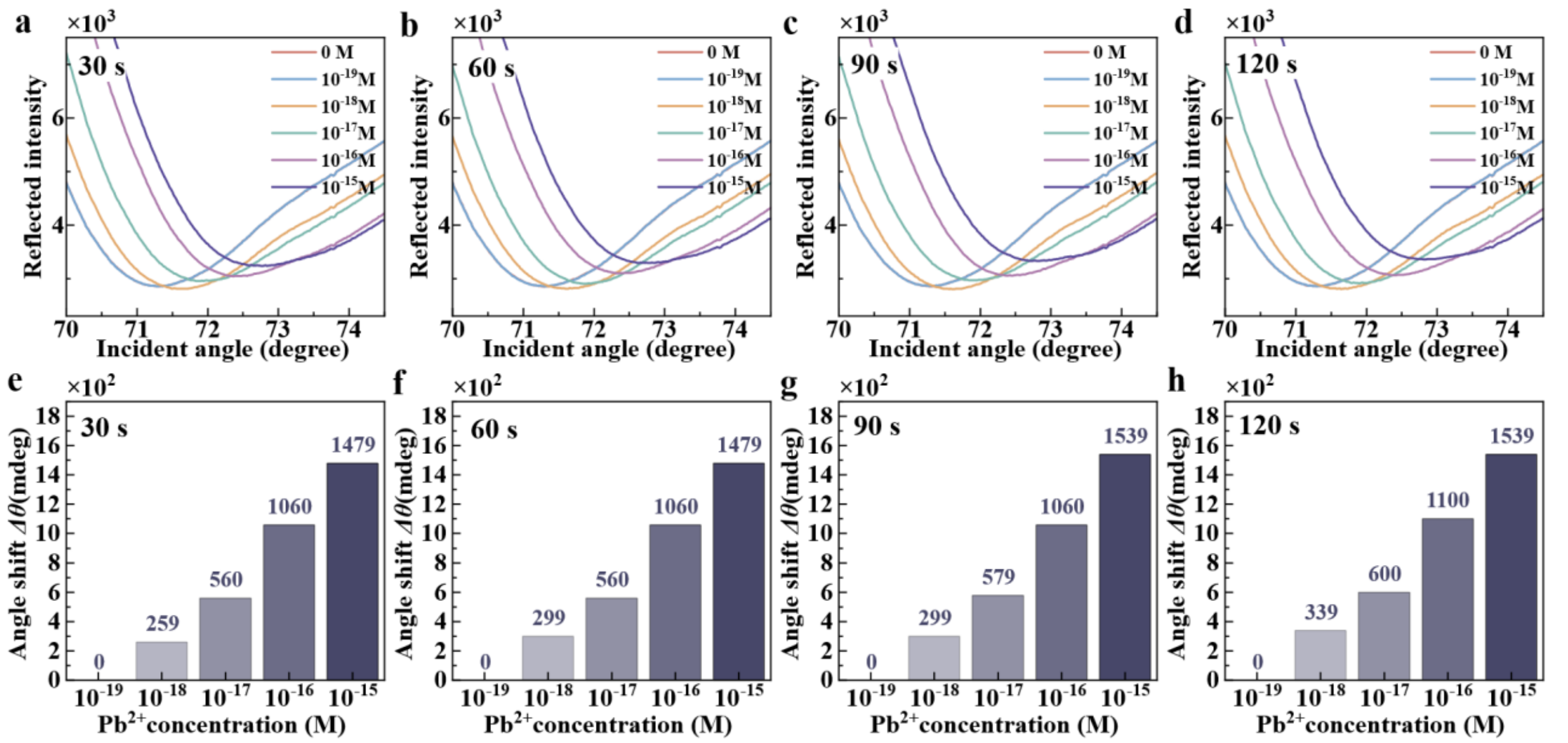

Supplementary Figure 15. Time-dependent Pb^{2+} sensing results obtained from one Au+BP-COOH chip. (a–d) Angle-scanned reflected-intensity curves measured for Pb^{2+} concentrations of 0, 10^{-19}, 10^{-18}, 10^{-17}, 10^{-16} and 10^{-15} M at 30, 60, 90 and 120 s, respectively. **(e–h)** Corresponding resonance-angle shift $\Delta\theta$ values at 30, 60, 90 and 120 s, respectively.

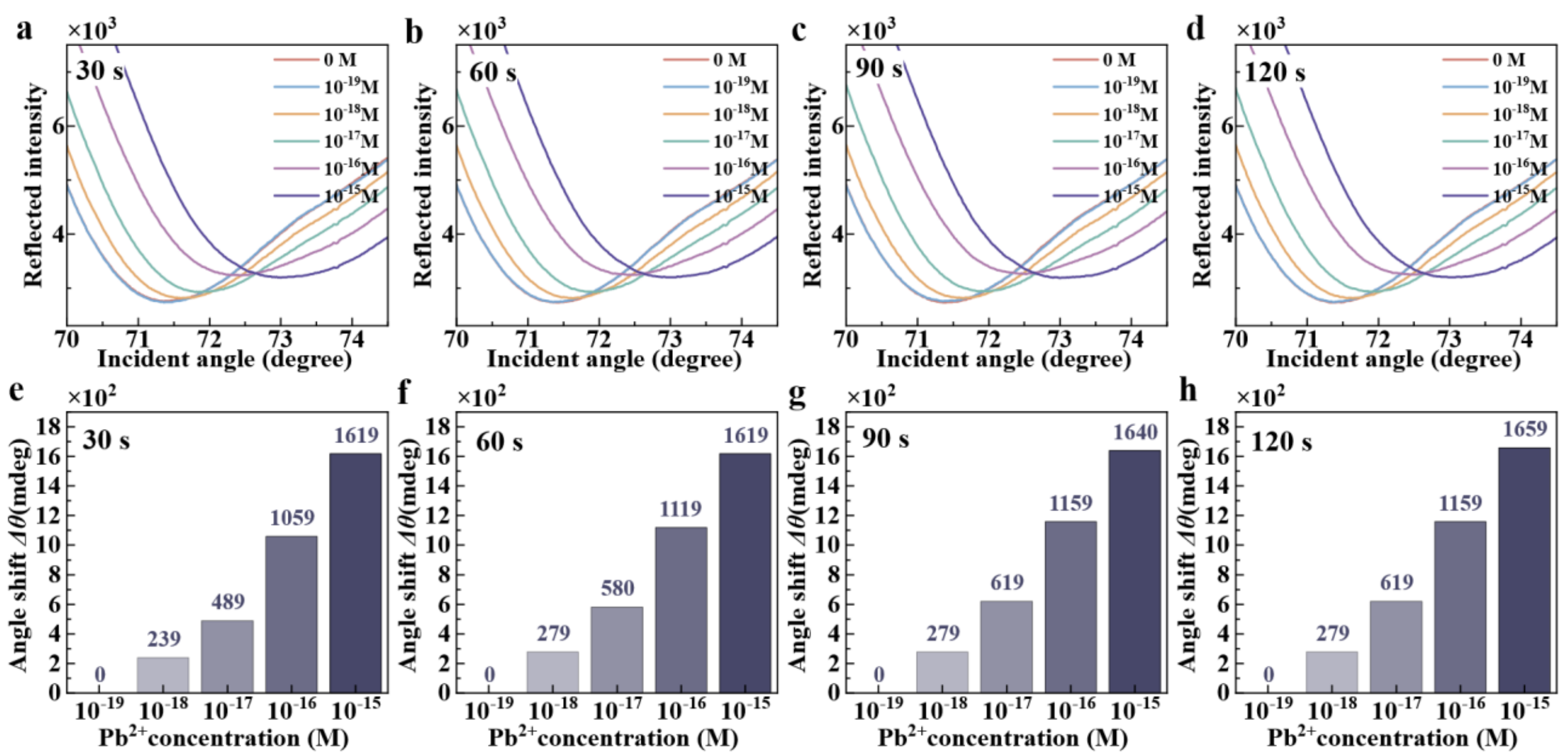

Supplementary Figure 16. Time-dependent Pb^{2+} sensing results obtained from one Au+BP-COOH chip. (a–d) Angle-scanned reflected-intensity curves measured for Pb^{2+} concentrations of 0, 10^{-19}, 10^{-18}, 10^{-17}, 10^{-16} and 10^{-15} M at 30, 60, 90 and 120 s, respectively. **(e–h)** Corresponding resonance-angle shift $\Delta\theta$ values at 30, 60, 90 and 120 s, respectively.

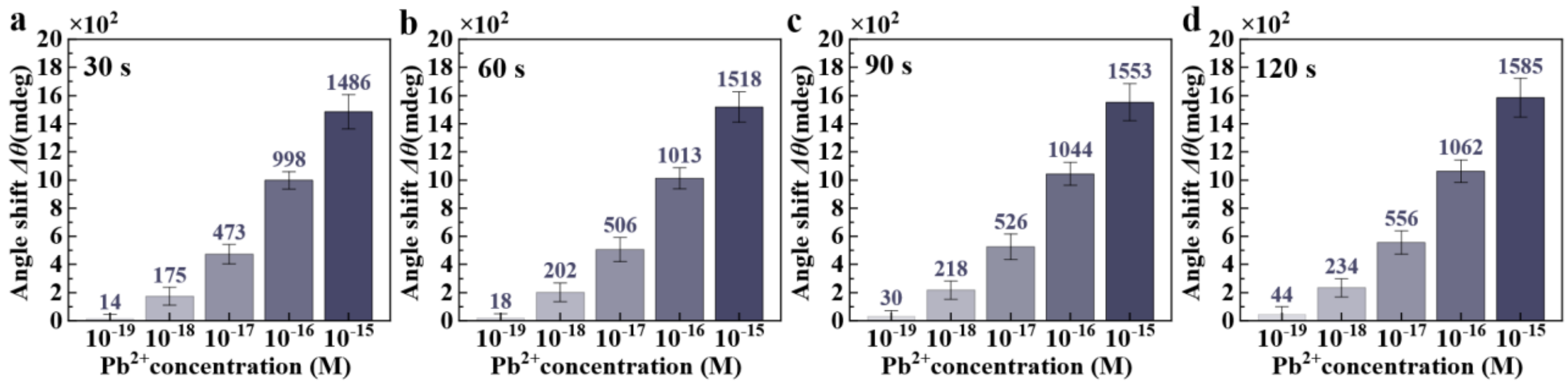

Supplementary Figure 17. Statistical Pb^{2+} sensing results obtained from the ten Au+BP-COOH chips shown in Supplementary Figures 7–16 at different detection times. (a–d) Resonance-angle shift $\Delta\theta$ as a function of Pb^{2+} concentration at 30, 60, 90 and 120 s, respectively. Bars show mean values, and error bars represent the standard deviation of measurements from the ten independently prepared Au+BP-COOH chips shown in Supplementary Figures 7–16.

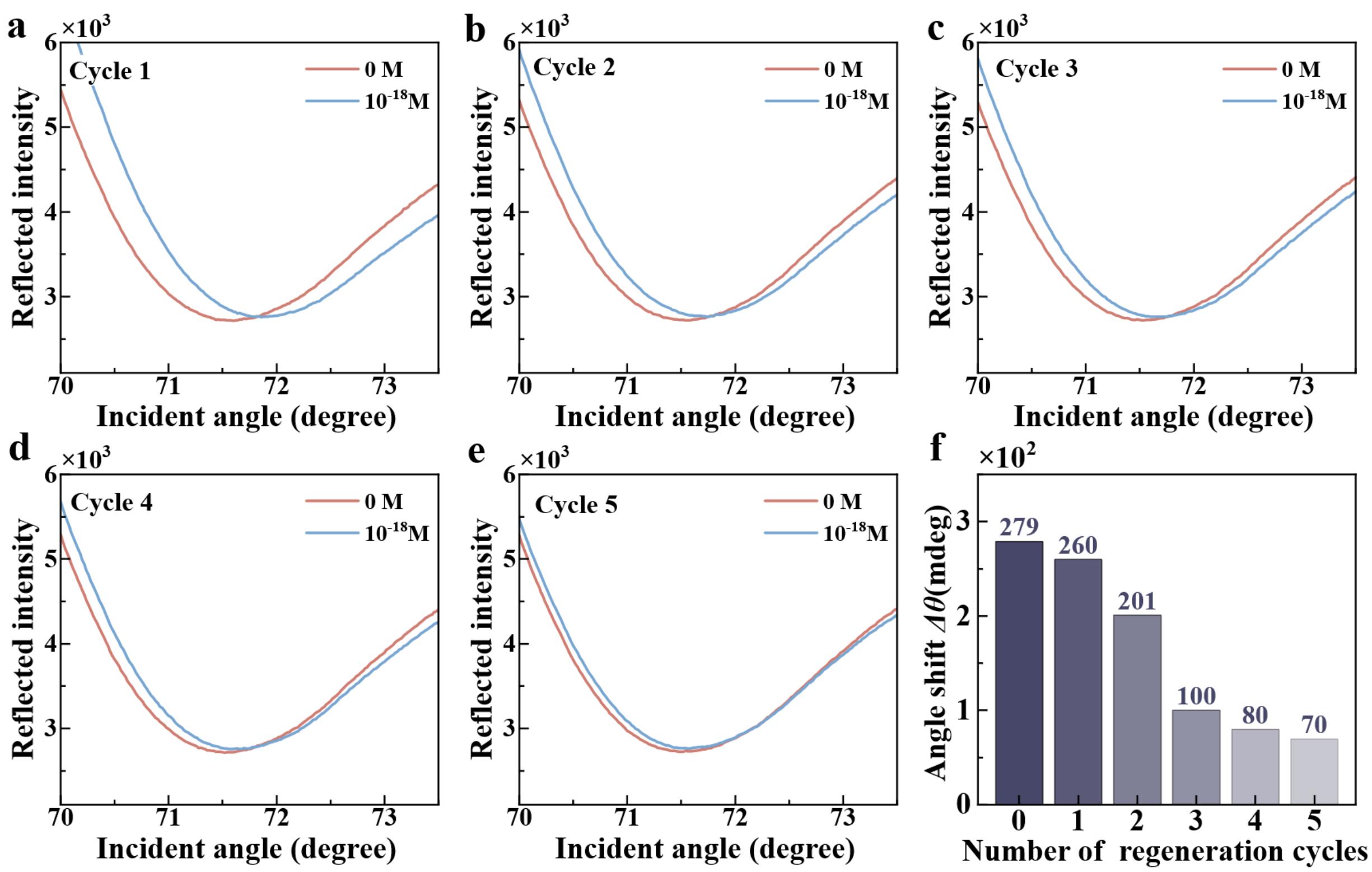

Supplementary Figure 18. Regeneration results obtained from the Au+BP-COOH chip shown in Supplementary Figure 16 using 10^{-18} M Pb^{2+}. (a–e) Angle-scanned reflected-intensity curves recorded for 0 M and 10^{-18} M Pb^{2+} in Cycles 1, 2, 3, 4 and 5, respectively. **(f)** Resonance-angle shift $\Delta\theta$ as a function of the number of regeneration cycles. The value at 0 corresponds to the initial 10^{-18} M Pb^{2+} sensing response before regeneration, and the values at 1–5 correspond to the responses after 1–5 regeneration cycles, respectively.

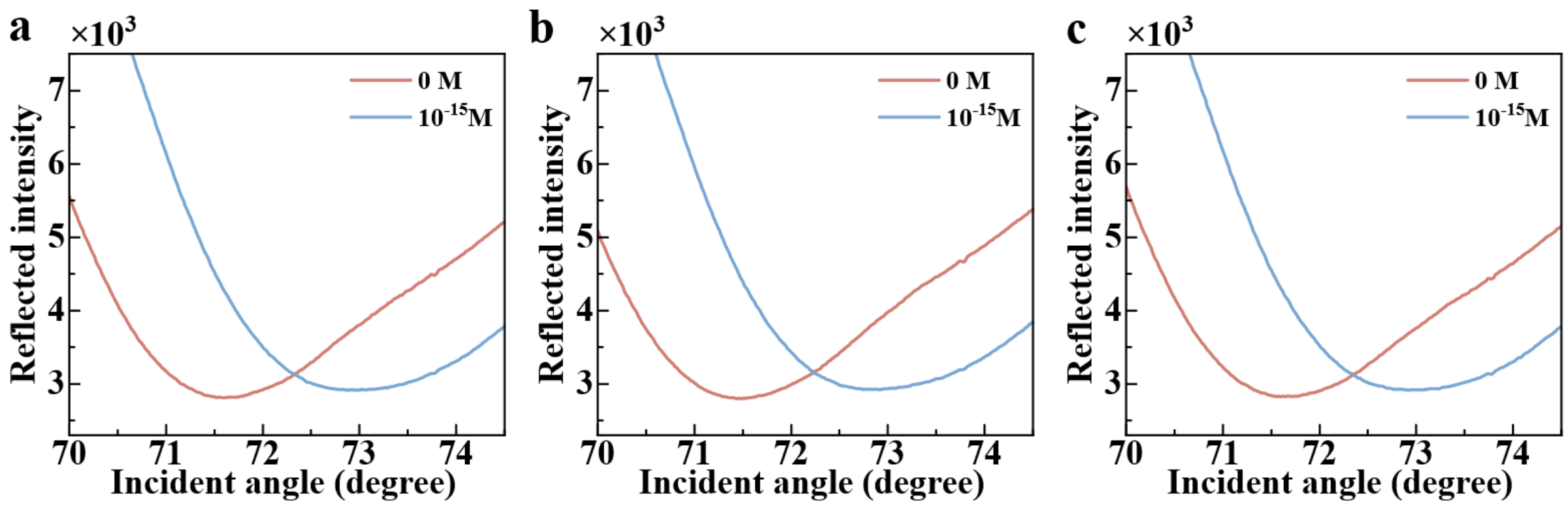

Supplementary Figure 19. Pb^{2+} sensing results obtained from Au+BP-COOH chips at storage time point Day 2. (a–c) Angle-scanned reflected-intensity curves of three independently prepared Au+BP-COOH chips measured for 0 M and 10^{-15} M Pb^{2+}. Day 2 corresponds to chips measured after 1 day of storage.

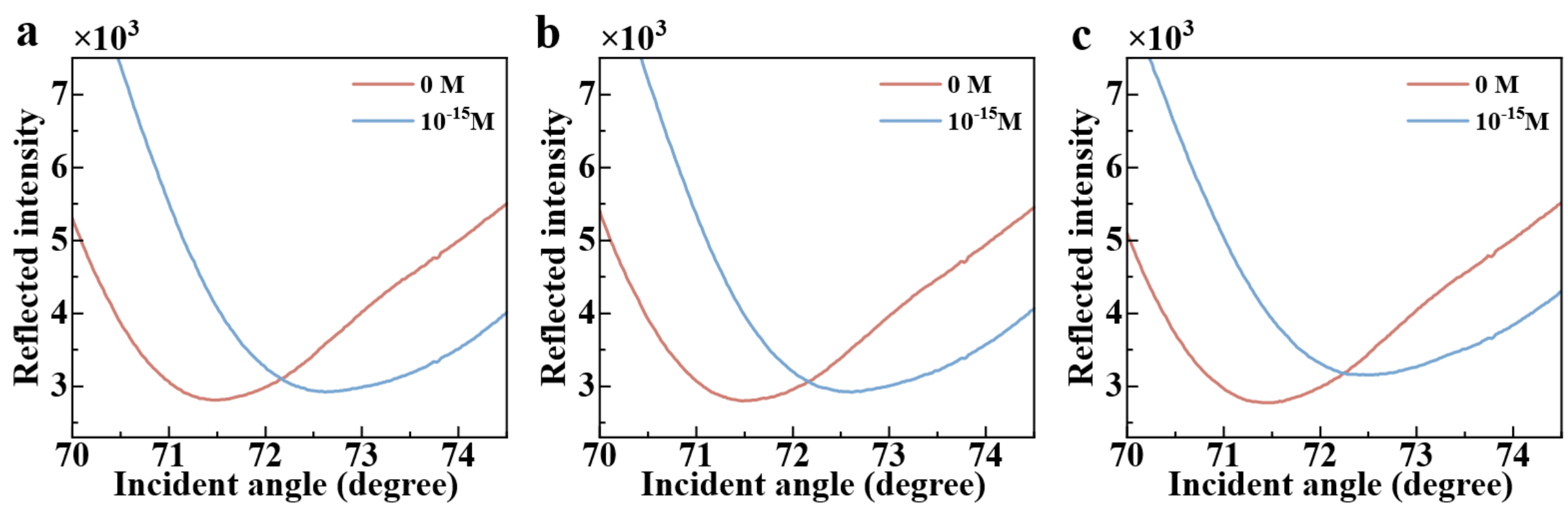

Supplementary Figure 20. Pb^{2+} sensing results obtained from Au+BP-COOH chips at storage time point Day 3. (a–c) Angle-scanned reflected-intensity curves of three independently prepared Au+BP-COOH chips measured for 0 M and 10^{-15} M Pb^{2+}. Day 3 corresponds to chips measured after 2 days of storage.

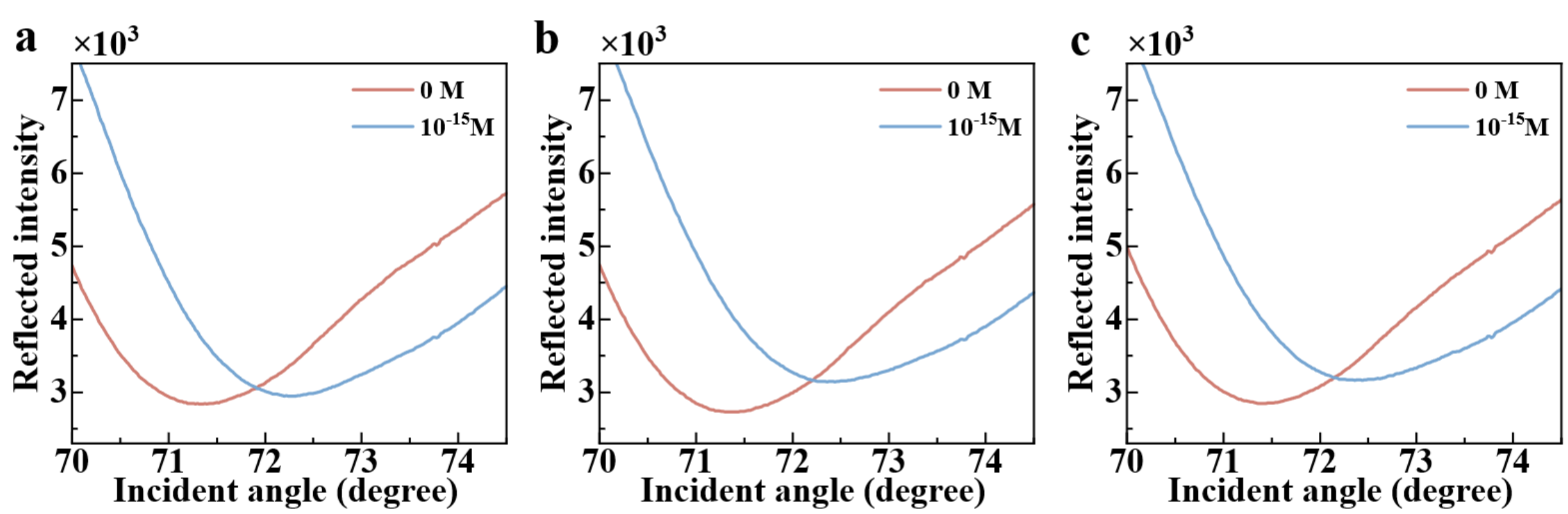

Supplementary Figure 21. Pb^{2+} sensing results obtained from Au+BP-COOH chips at storage time point Day 4. (a–c) Angle-scanned reflected-intensity curves of three independently prepared Au+BP-COOH chips measured for 0 M and 10^{-15} M Pb^{2+}. Day 4 corresponds to chips measured after 3 days of storage.

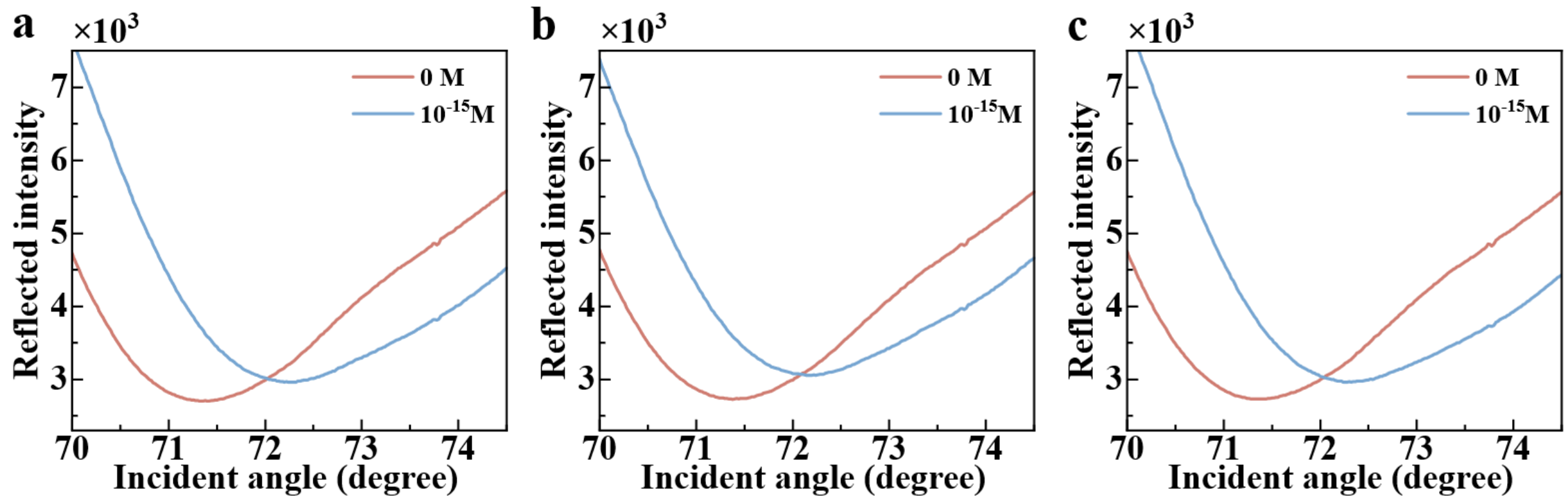

Supplementary Figure 22. **Pb^{2+} sensing results obtained from Au+BP-COOH chips at storage time point Day 5. (a–c)** Angle-scanned reflected-intensity curves of three independently prepared Au+BP-COOH chips measured for 0 M and 10^{-15} M Pb^{2+}. Day 5 corresponds to chips measured after 4 days of storage.

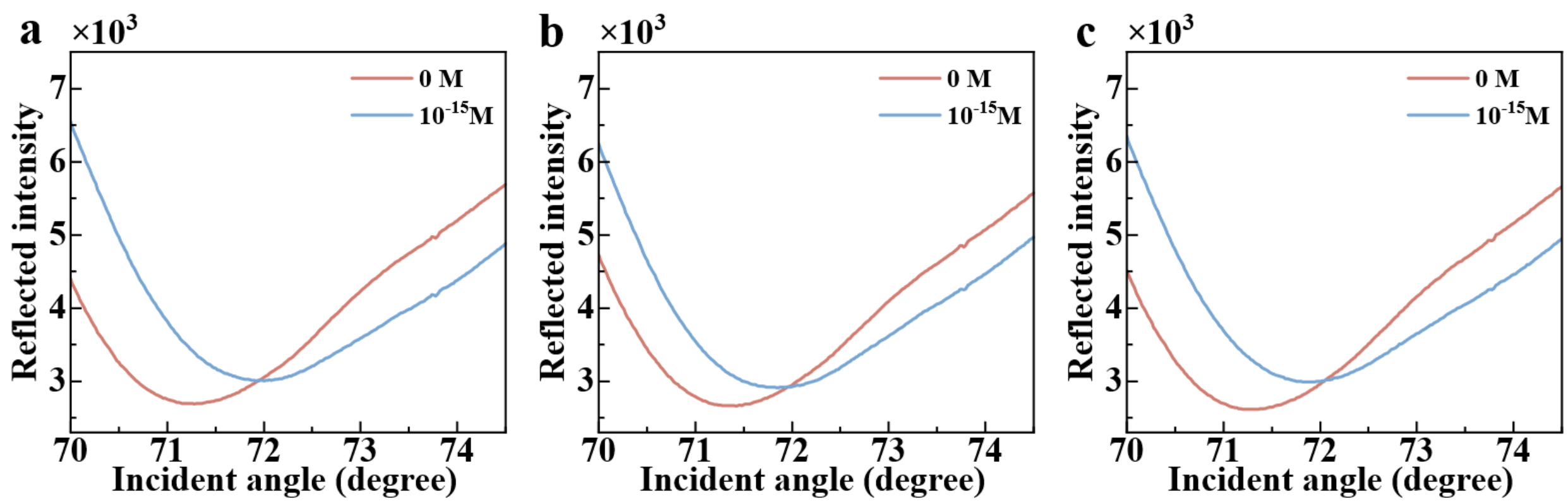

Supplementary Figure 23. Pb^{2+} sensing results obtained from Au+BP-COOH chips at storage time point Day 6. (a–c) Angle-scanned reflected-intensity curves of three independently prepared Au+BP-COOH chips measured for 0 M and 10^{-15} M Pb^{2+}. Day 6 corresponds to chips measured after 5 days of storage.

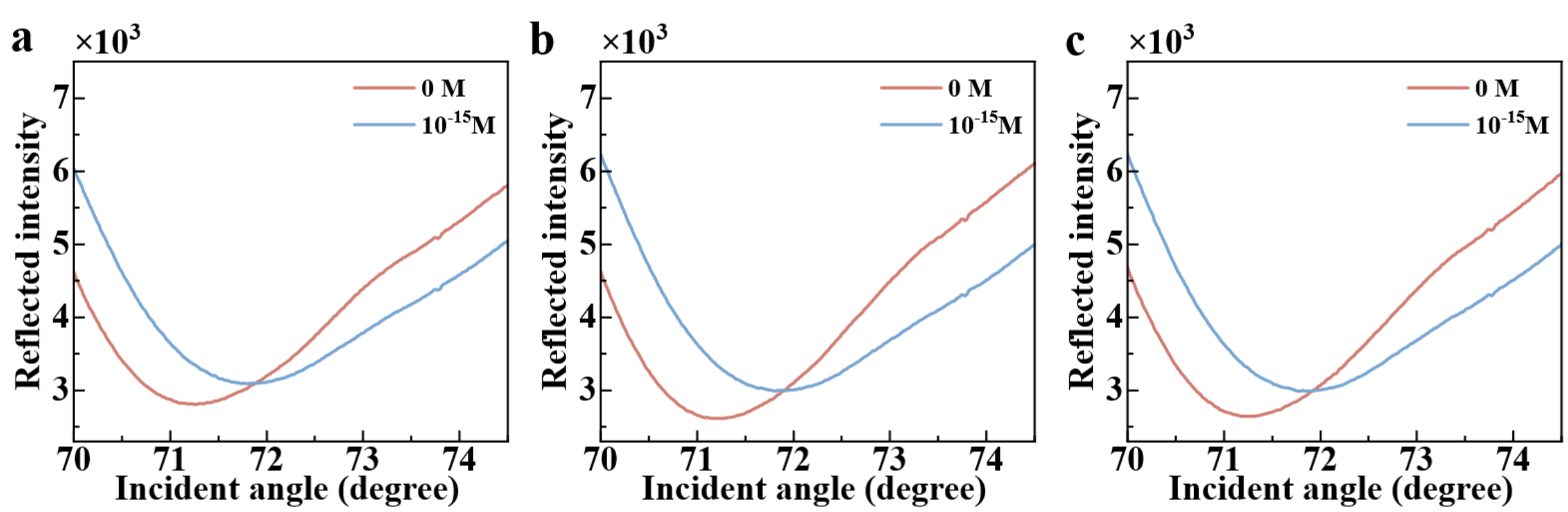

Supplementary Figure 24. Pb^{2+} sensing results obtained from Au+BP-COOH chips at storage time point Day 7. (a–c) Angle-scanned reflected-intensity curves of three independently prepared Au+BP-COOH chips measured for 0 M and 10^{-15} M Pb^{2+}. Day 7 corresponds to chips measured after 6 days of storage.

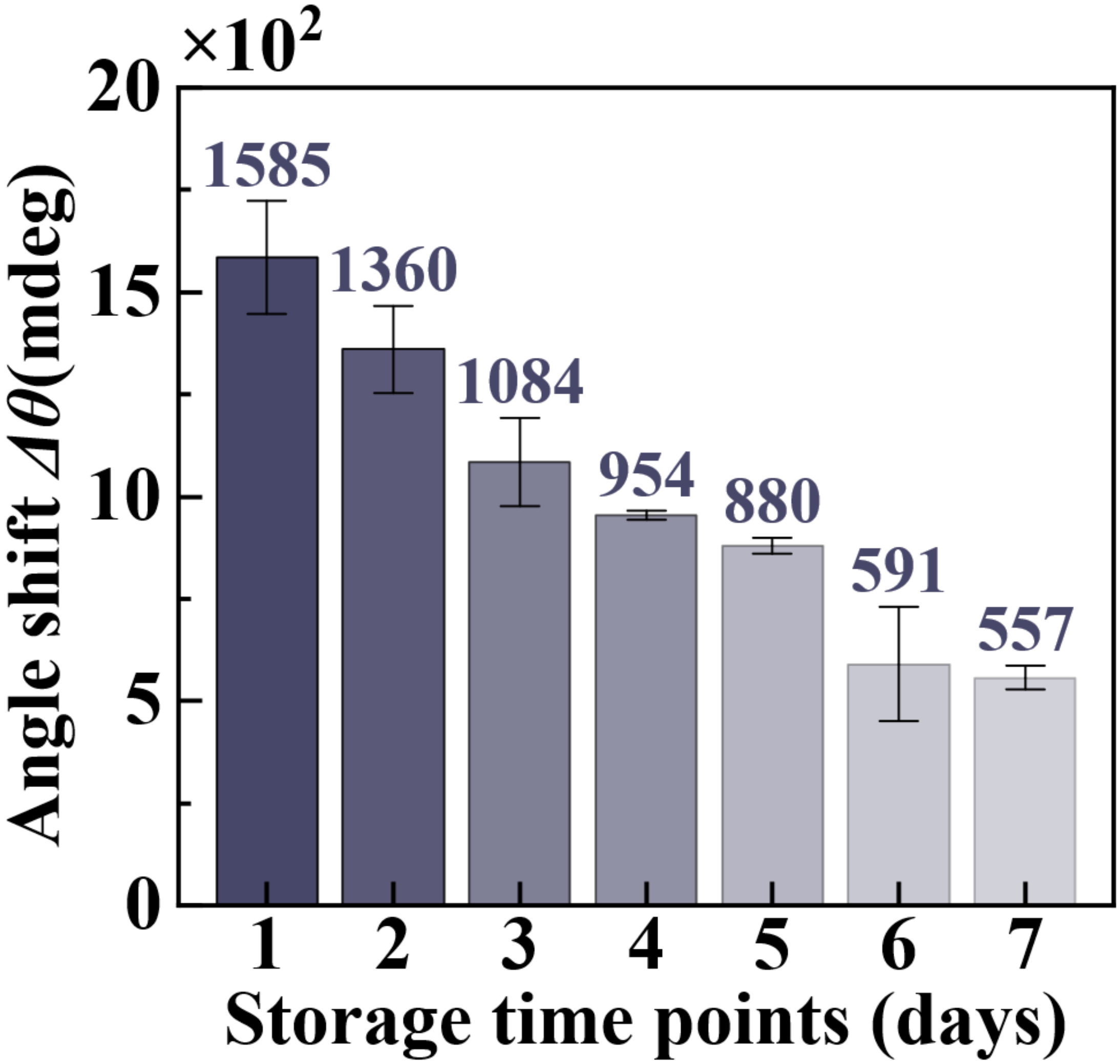

Supplementary Figure 25. Storage-stability statistics of Au+BP-COOH chips measured with 10^{-15} M Pb^{2+} at different storage time points. Resonance-angle shift *Δθ* of Au+BP-COOH chips measured with 10^{-15} M Pb^{2+} from Day 1 to Day 7. Day 1 corresponds to freshly prepared chips measured immediately after fabrication, and Days 2–7 correspond to chips measured after 1, 2, 3, 4, 5 and 6 days of storage, respectively. Bars show mean values, and error bars represent the standard deviation of measurements from ten independently prepared Au+BP-COOH chips on Day 1 and three independently prepared Au+BP-COOH chips at each of Days 2–7. The mean *Δθ* decreases from 1585 mdeg on Day 1 to 557 mdeg on Day 7, corresponding to a 64.9% decrease and retention of 35.1% of the initial response.

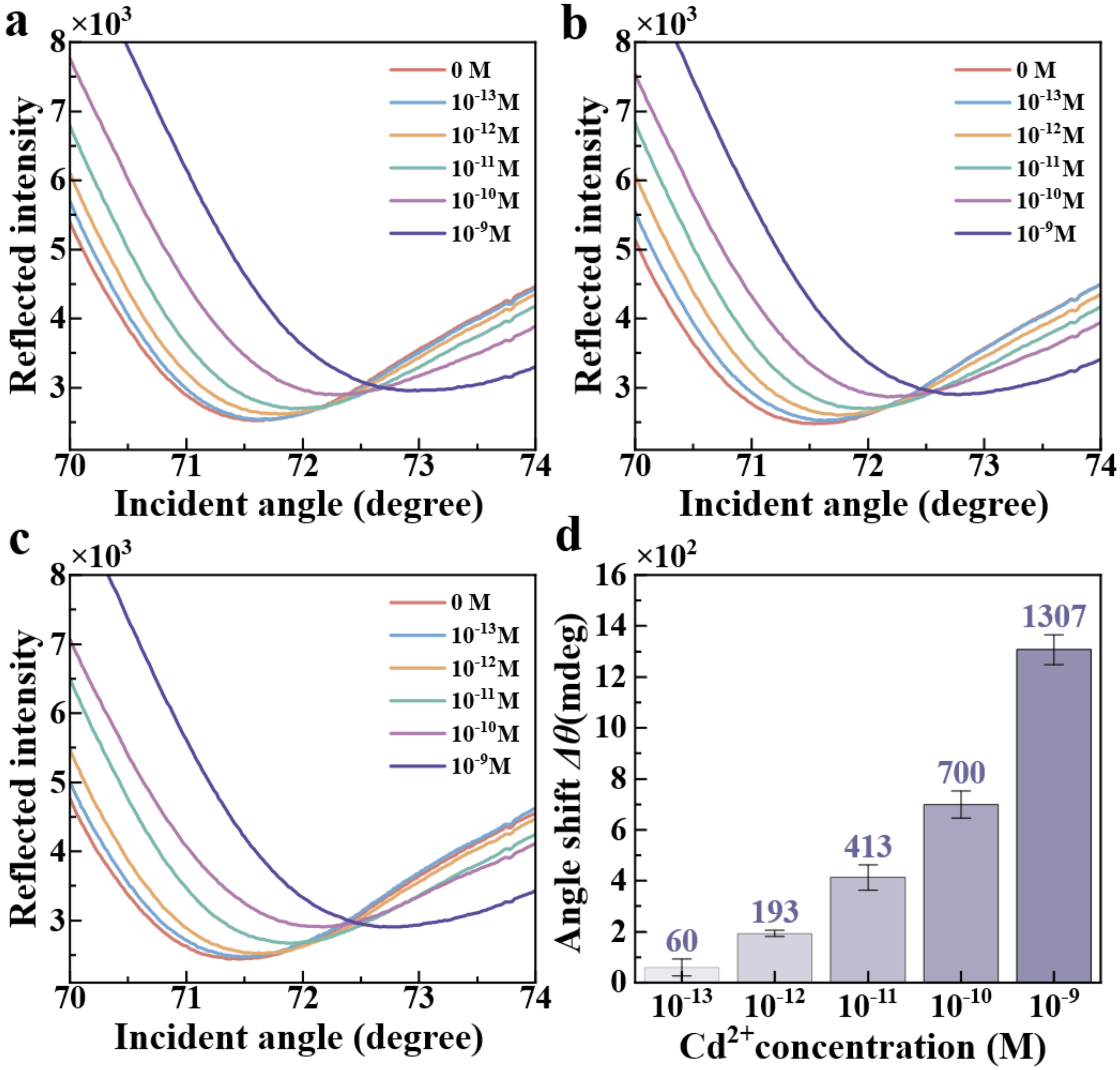

Supplementary Figure 26. Cd^{2+} sensing results obtained from Au+BP-COOH. (a–c) Angle-scanned reflected-intensity curves of three independently prepared Au+BP-COOH chips measured for Cd^{2+} concentrations of 0, 10^{-13}, 10^{-12}, 10^{-11}, 10^{-10} and 10^{-9} M. **(d)** Resonance-angle shift $\Delta\theta$ as a function of Cd^{2+} concentration for Au+BP-COOH. Bars show mean values, and error bars represent the standard deviation of measurements from three independently prepared Au+BP-COOH chips.

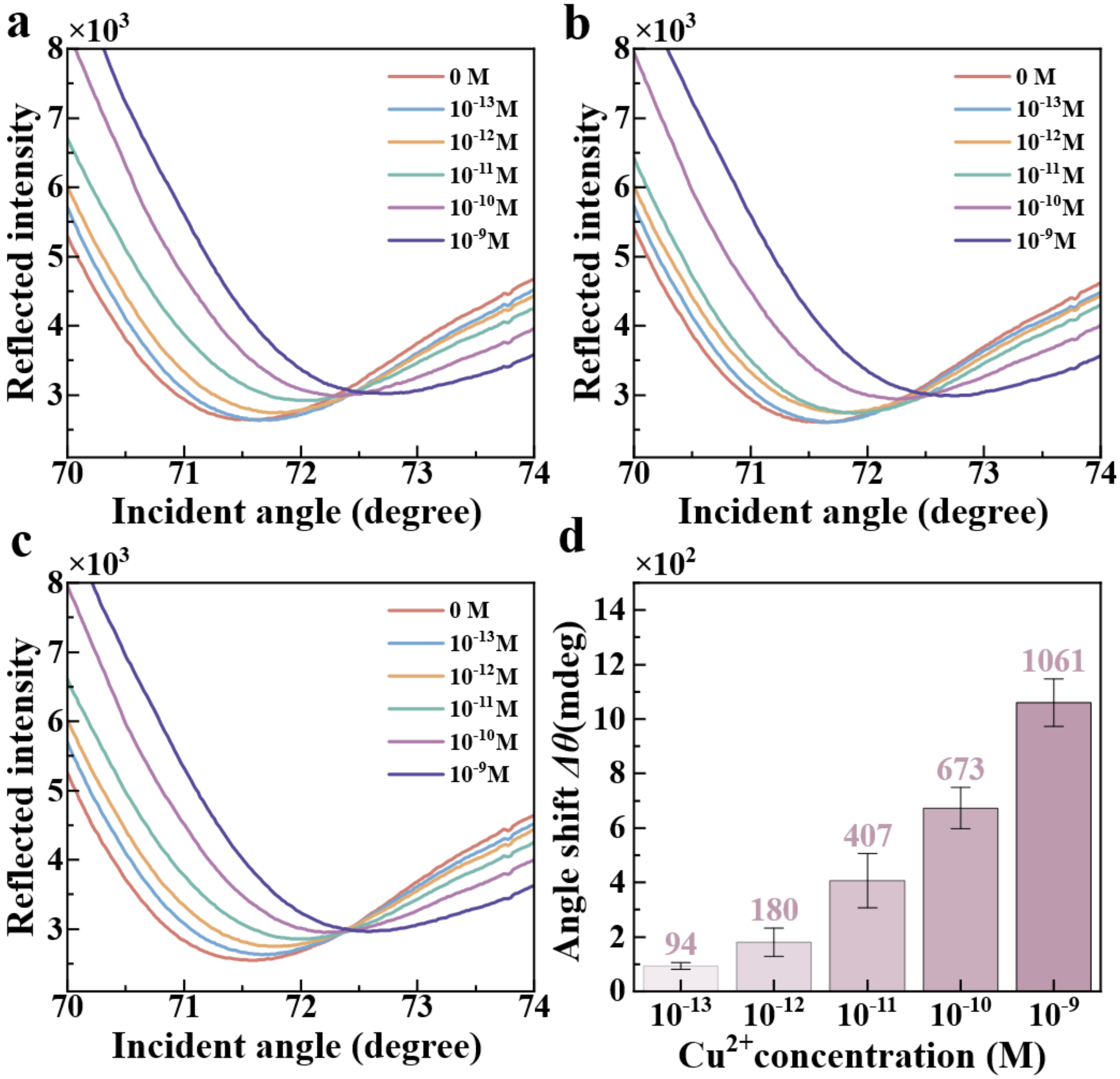

Supplementary Figure 27. Cu^{2+} sensing results obtained from Au+BP-COOH. (a–c) Angle-scanned reflected-intensity curves of three independently prepared Au+BP-COOH chips measured for Cu^{2+} concentrations of 0, 10^{-13}, 10^{-12}, 10^{-11}, 10^{-10} and 10^{-9} M. **(d)** Resonance-angle shift $\Delta\theta$ as a function of Cu^{2+} concentration for Au+BP-COOH. Bars show mean values, and error bars represent the standard deviation of measurements from three independently prepared Au+BP-COOH chips.

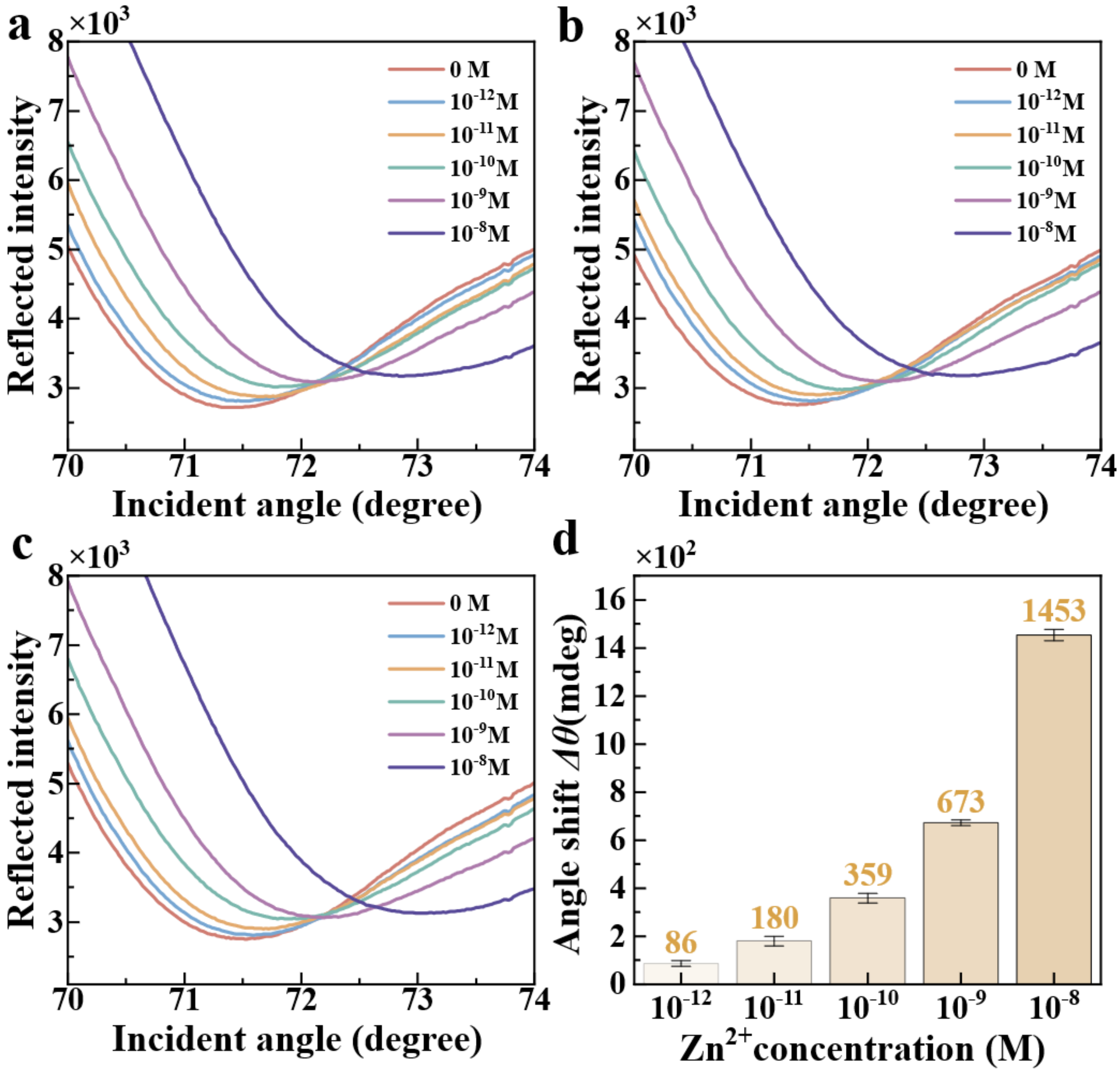

Supplementary Figure 28. Zn^{2+} sensing results obtained from Au+BP-COOH. (a–c) Angle-scanned reflected-intensity curves of three independently prepared Au+BP-COOH chips measured for Zn^{2+} concentrations of 0, 10^{-12}, 10^{-11}, 10^{-10}, 10^{-9} and 10^{-8} M. **(d)** Resonance-angle shift $\Delta\theta$ as a function of Zn^{2+} concentration for Au+BP-COOH. Bars show mean values, and error bars represent the standard deviation of measurements from three independently prepared Au+BP-COOH chips.

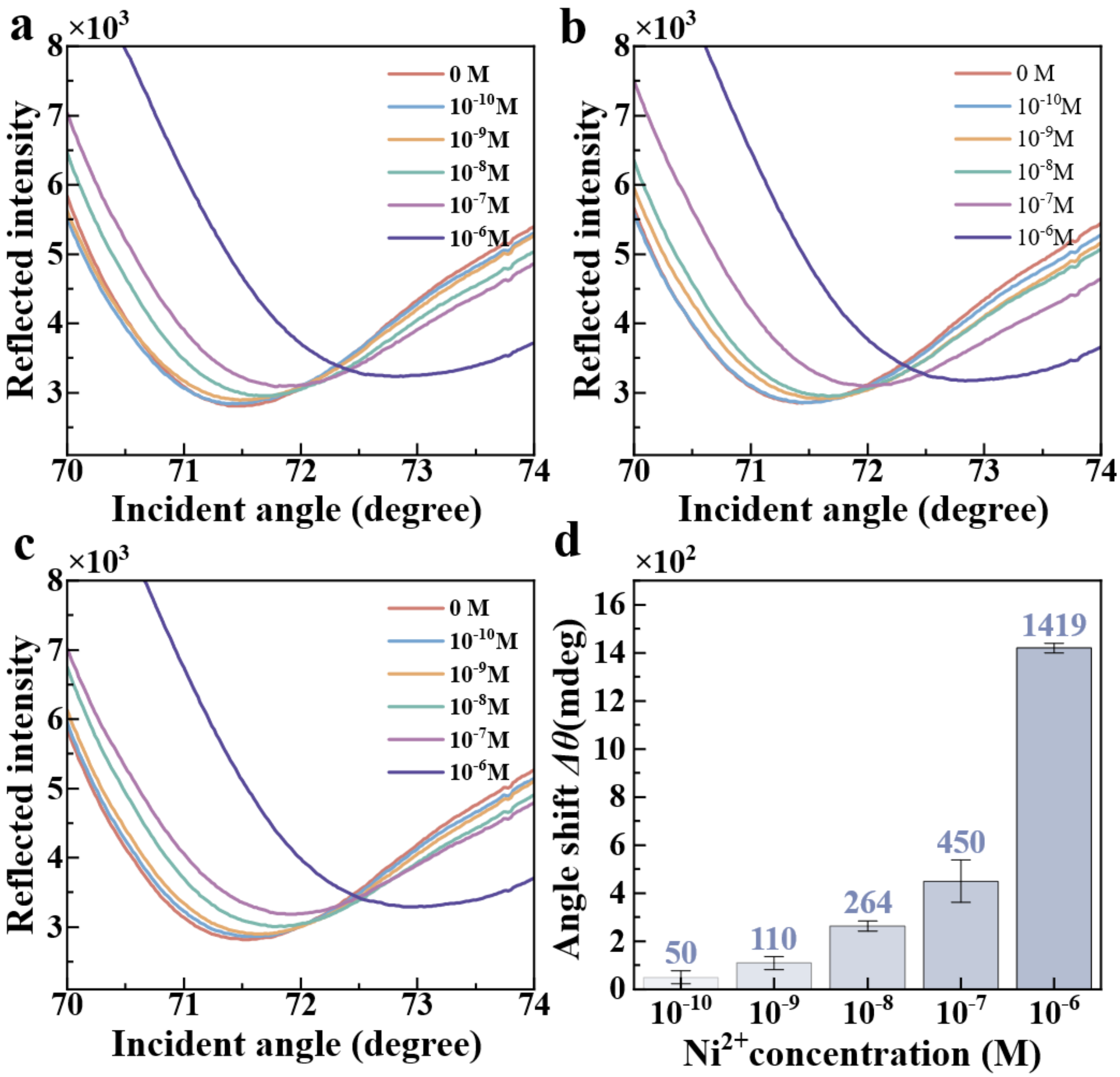

Supplementary Figure 29. Ni^{2+} sensing results obtained from Au+BP-COOH. (a–c) Angle-scanned reflected-intensity curves of three independently prepared Au+BP-COOH chips measured for Ni^{2+} concentrations of 0, 10^{-10}, 10^{-9}, 10^{-8}, 10^{-7} and 10^{-6} M. **(d)** Resonance-angle shift $\Delta\theta$ as a function of Ni^{2+} concentration for Au+BP-COOH. Bars show mean values, and error bars represent the standard deviation of measurements from three independently prepared Au+BP-COOH chips.

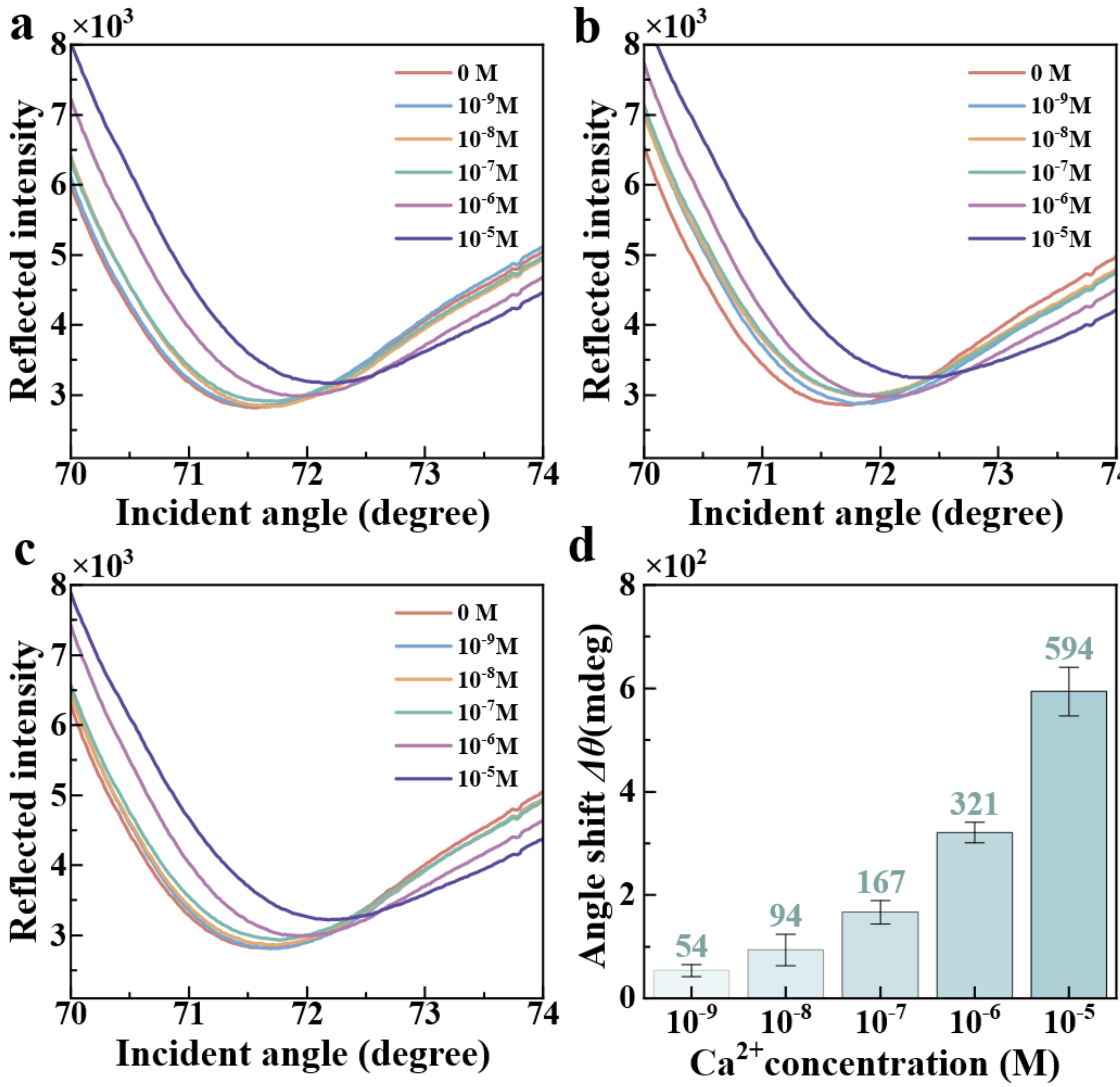

Supplementary Figure 30. Ca^{2+} sensing results obtained from Au+BP-COOH. (a–c) Angle-scanned reflected-intensity curves of three independently prepared Au+BP-COOH chips measured for Ca^{2+} concentrations of 0, 10^{-9}, 10^{-8}, 10^{-7}, 10^{-6} and 10^{-5} M. **(d)** Resonance-angle shift $\Delta\theta$ as a function of Ca^{2+} concentration for Au+BP-COOH. Bars show mean values, and error bars represent the standard deviation of measurements from three independently prepared Au+BP-COOH chips.

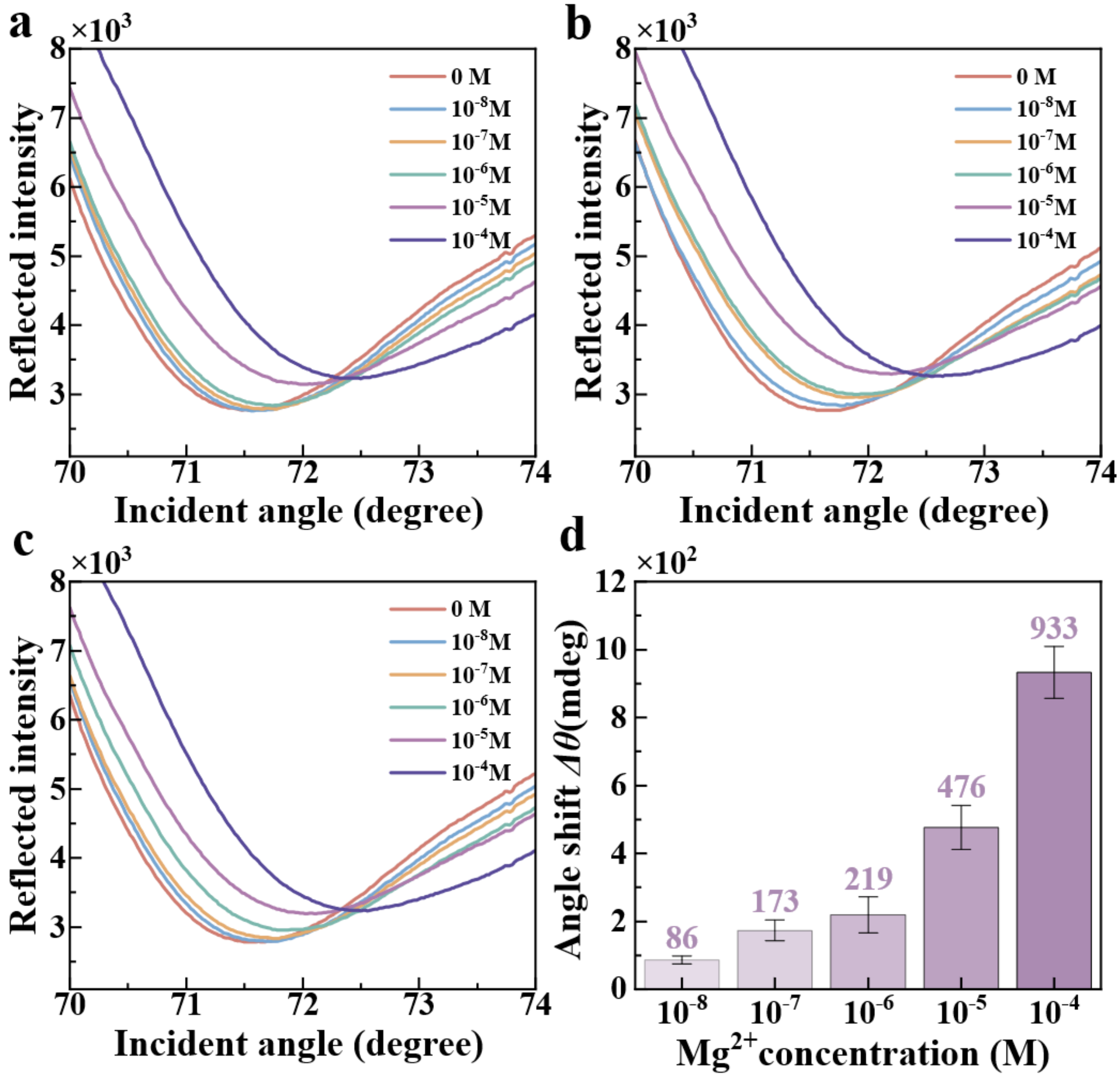

Supplementary Figure 31. Mg^{2+} sensing results obtained from Au+BP-COOH. (a–c) Angle-scanned reflected-intensity curves of three independently prepared Au+BP-COOH chips measured for Mg^{2+} concentrations of 0, 10^{-8}, 10^{-7}, 10^{-6}, 10^{-5} and 10^{-4} M. **(d)** Resonance-angle shift $\Delta\theta$ as a function of Mg^{2+} concentration for Au+BP-COOH. Bars show mean values, and error bars represent the standard deviation of measurements from three independently prepared Au+BP-COOH chips.

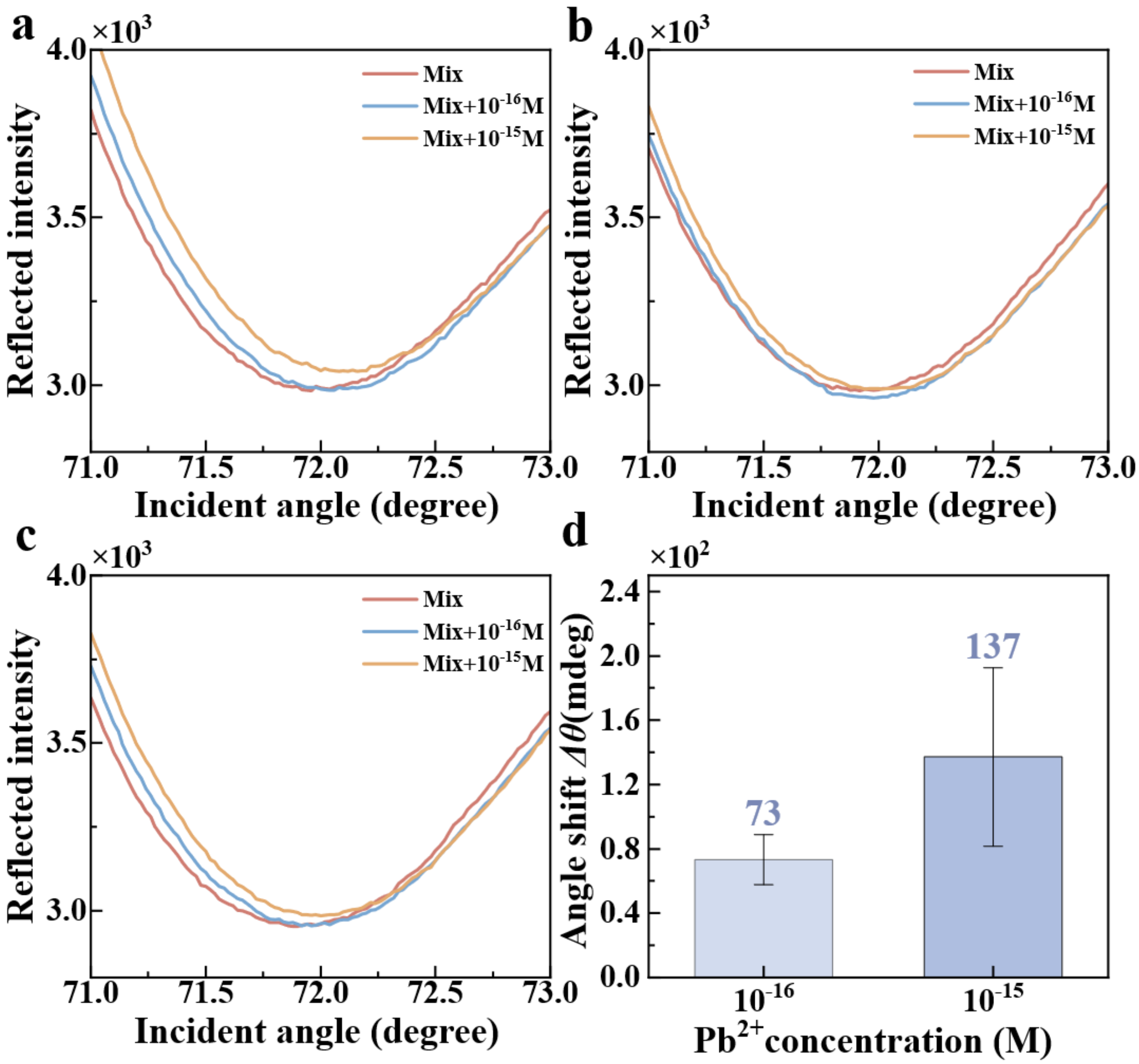

Supplementary Figure 32. Mixed-ion interference sensing results obtained from Au+BP-COOH. (a–c) Angle-scanned reflected-intensity curves of three independently prepared Au+BP-COOH chips measured in Mix, Mix + 10^{-16} M Pb^{2+} and Mix + 10^{-15} M Pb^{2+}. Mix represents the mixed-ion solution containing Cd^{2+}, Cu^{2+}, Zn^{2+}, Ni^{2+}, Ca^{2+} and Mg^{2+}. **(d)** Net resonance-angle shift $\Delta\theta$ as a function of Pb^{2+} concentration in the mixed-ion solution. Bars show mean values, and error bars represent the standard deviation of measurements from three independently prepared Au+BP-COOH chips.

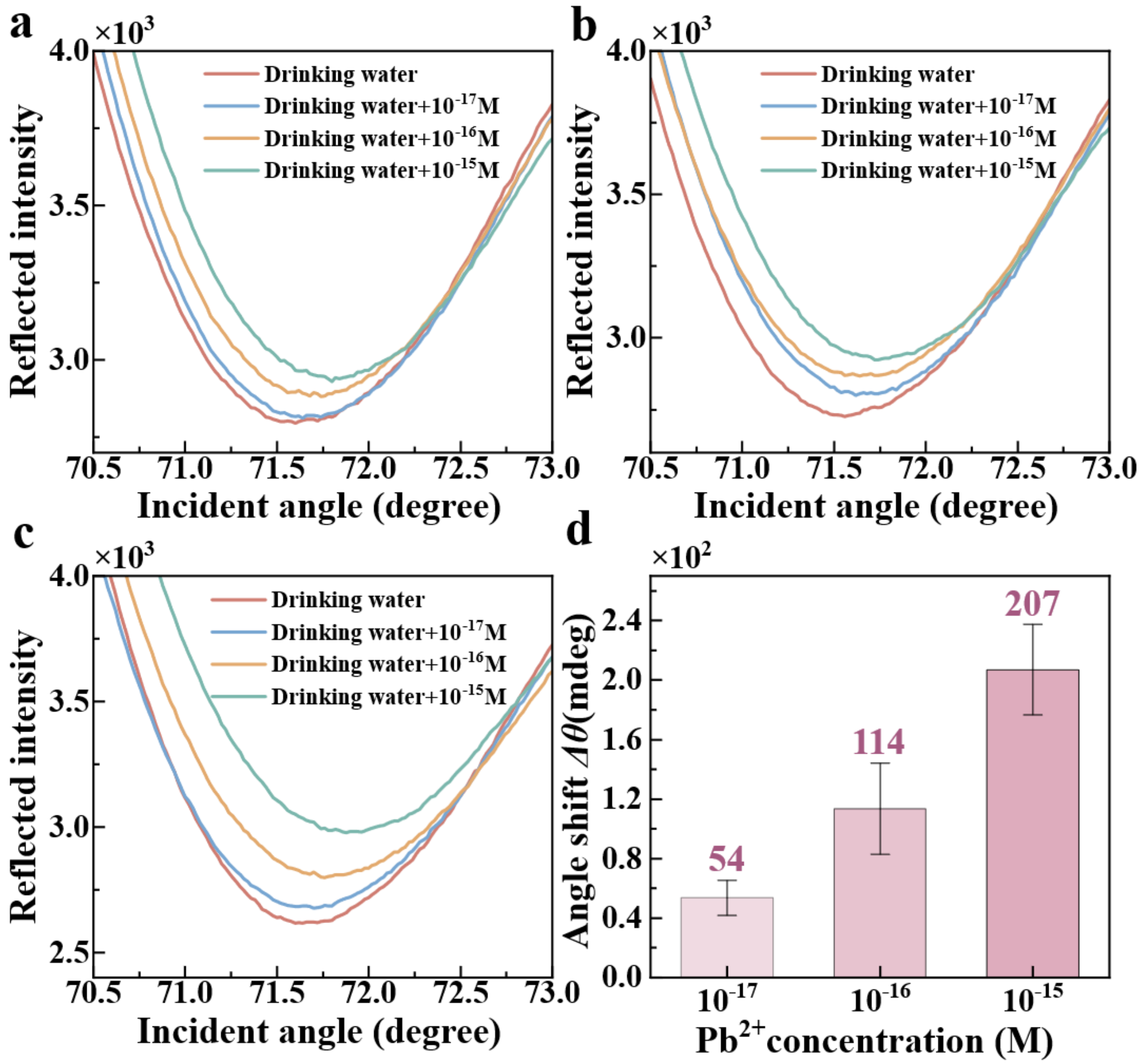

Supplementary Figure 33. Pb^{2+} sensing results obtained from Au+BP-COOH in drinking water. (a–c) Angle-scanned reflected-intensity curves of three independently prepared Au+BP-COOH chips measured in drinking water, drinking water + 10^{-17} M Pb^{2+}, drinking water + 10^{-16} M Pb^{2+} and drinking water + 10^{-15} M Pb^{2+}. **(d)** Net resonance-angle shift $\Delta\theta$ as a function of Pb^{2+} concentration in drinking water. Bars show mean values, and error bars represent the standard deviation of measurements from three independently prepared Au+BP-COOH chips.

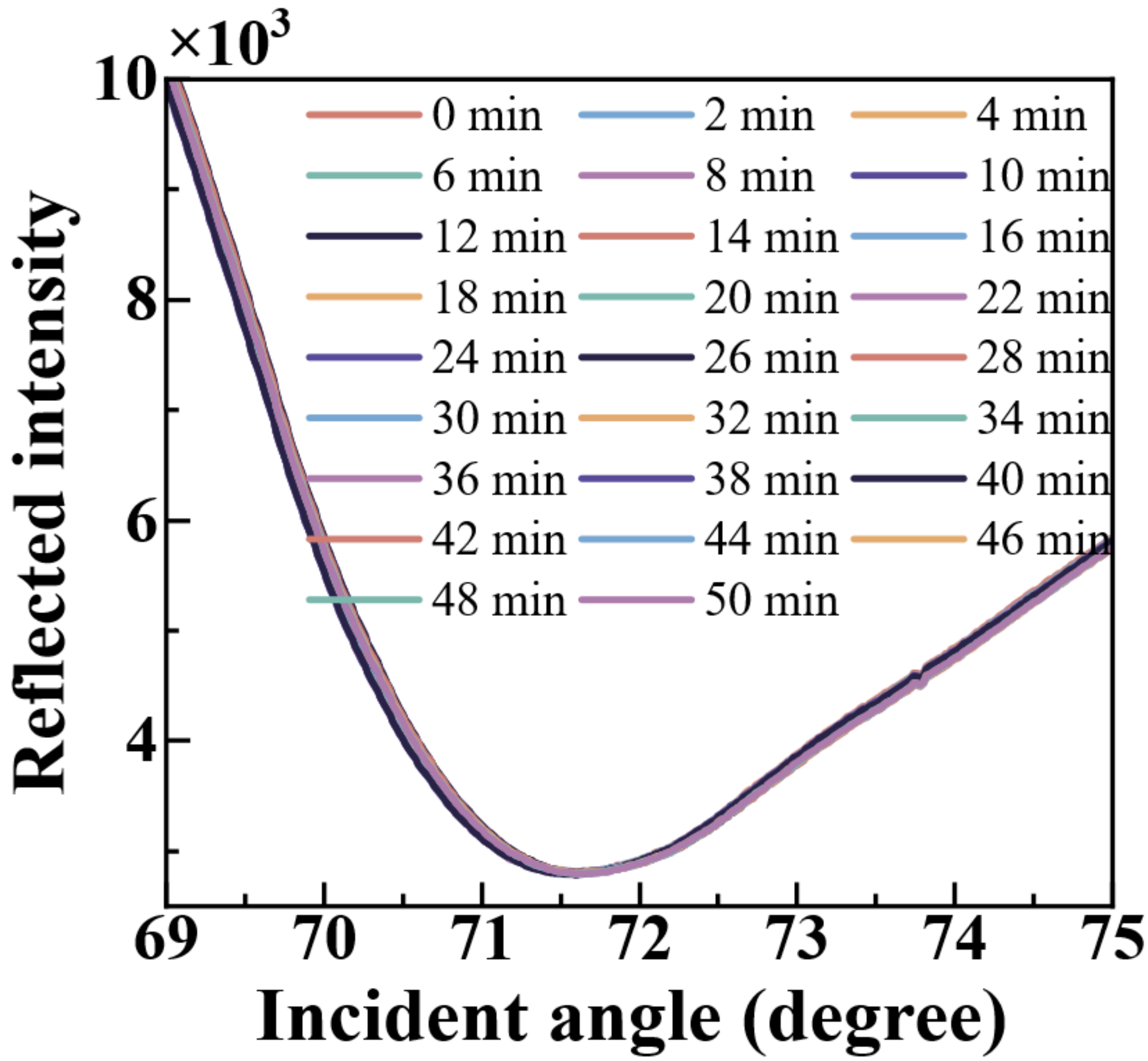

Supplementary Figure 34. Baseline stability of Au+BP-COOH in pure water. Angle-scanned reflected-intensity curves recorded on the same Au+BP-COOH chip in pure water at 2 min intervals over 50 min. The maximum resonance-angle drift during the 50 min measurement was 21 mdeg, and the standard deviation of the resonance angle was 0.00714 deg.

Supplementary Tables

Supplementary Table 1. Water-quality background of the drinking-water sample used for real-sample Pb^{2+} sensing.

Parameter	Unit	Sample	QC duplicate	Mean / reported value	Relative deviation	Acceptance criterion	QC result
pH	—	7.0	7.0	7.0	0.00	±0.1 pH unit	Pass
Conductivity	μS cm^{-1}	70.6	72.4	71.5	0.6%	≤10%	Pass
Total dissolved solids	mg L^{-1}	58	62	60	3.3%	≤10%	Pass
Na^{+}	mg L^{-1}	2.71	2.66	2.69	0.9%	≤10%	Pass
K^{+}	mg L^{-1}	2.90	2.84	2.87	1.0%	≤10%	Pass
Mg^{2+}	mg L^{-1}	1.85	1.80	1.83	1.4%	≤10%	Pass
Ca^{2+}	mg L^{-1}	10.1	9.8	10.0	1.5%	≤10%	Pass
Cl^{-}	mg L^{-1}	2.40	2.45	2.43	1.0%	≤10%	Pass
NO_3^{-}	mg L^{-1}	1.74	1.77	1.76	0.9%	≤10%	Pass
SO_4^{2-}	mg L^{-1}	4.61	4.55	4.58	0.7%	≤10%	Pass
CO_3^{2-}	mg L^{-1}	n.a.	n.a.	n.a.	—	—	—
HCO_3^{-}	mg L^{-1}	36.8	39.3	38.1	3.2%	≤10%	Pass
Hg	μg L^{-1}	0.000	0.000	n.d.	0.0%	≤20%	Pass
Cu	μg L^{-1}	0.213	0.231	0.22	4.1%	≤20%	Pass
Pb	μg L^{-1}	0.000	0.000	n.d.	0.0%	≤20%	Pass
Cd	μg L^{-1}	0.005	0.002	n.d.	0.0%	≤20%	Pass
Zn	μg L^{-1}	0.127	0.120	0.124	2.8%	≤20%	Pass
Ni	μg L^{-1}	0.012	0.012	0.012	0.0%	≤20%	Pass
Total Cr	μg L^{-1}	n.d.	n.d.	n.d.	—	≤10%	Pass

Values are from duplicate external measurements of the drinking-water sample. Relative deviations were provided by the testing laboratory. n.d., not detected; n.a., not applicable. For Cd and total Cr, trace instrumental signals were recorded, but the final reported results were below the reporting/detection threshold and are therefore listed as n.d.
