# Supplementary material for: Built-in electric field-boosted attomolar Pb²⁺ sensing on carboxylated black phosphorene
Source: Nat Commun. 2026 Sep 14;17:10772. doi: 10.1038/s41467-026-77793-6 (PMC13661523; doi:10.1038/s41467-026-77793-6)
Supplement: Supplementary file 2 [file 41467_2026_77793_MOESM2_ESM.pdf]

nature portfolio

Peer Review File

Built-in electric field-boosted attomolar Pb^{2+} sensing on carboxylated black phosphorene

Corresponding Author: Professor Leiming Wu

This file contains all reviewer reports in order by version, followed by all author rebuttals in order by version.

Version 0:

Reviewer comments:

Reviewer #1

(Remarks to the Author)
1. Usually, detection limit is defined as a concentration where a signal exceeds 3 times the noise level, while authors defined this with some ambiguity, simply stating that the response exceeds the instrumental error range. Please clarify how the instrumental error range is quantified and how it was used in the calculation of detection limit.

2. The detection limit of 10^(-19) M is quite impressive but questionable at the same time. At this concentration, the 400 µL analyte solution used in this work contains only approximately 24 Pb2+ ions on average. Considering the actual adsorption rate onto the sensor surface and its contribution to angular changes, this is essentially a result from an extremely small (even countable) number of Pb2+ ions. Such a super-sensitive claim should be verified more carefully. There are no error bars or repeated independent measurements to support the validity of the sensing data. Also, at such extreme dilutions, how can the authors exclude the possibility of Pb2+ contamination from containers or pipette tips during sample preparation and measurement?

3. The authors mention the unstable nature of BP in the Introduction. What is the stability of the prepared BP-COOH under the aqueous sensing conditions used in this work, and over what timescale? Any experimental verification, such as repeated SPR measurements or post-exposure surface characterization, would be necessary to support practical applicability.

4. If this binding is not an enzymatic reaction but rather a chemical coordination, can it be reversible? Recovery and regeneration characteristics of the sensor surface should be examined and discussed.

5. The two configurations are labeled "SPR" and "SPR+BP-COOH" throughout the manuscript, but since SPR refers to the phenomenon itself rather than a specific sample, this is a bit confusing. I would suggest renaming them as "Au" and "Au+BP-COOH" for clarity.

6. How would the sensing performance change if Graphene-COOH or MoS2-COOH were used instead of BP-COOH, given similar thickness and the same functional group? This comparison would help isolate the contribution of the built-in electric field effect from the other two mechanisms (high-index confinement and interlayer coupling), which are not unique to BP.

7. The origin of Pb2+ selectivity is unclear. Interaction with –COOH can happen with other divalent ions as well. A more detailed explanation of why Pb2+ binds preferentially, in terms of ionic radius, HSAB theory, binding constants, or any other solid chemical reasoning, should be presented.

(Remarks on code availability)

Reviewer #2

(Remarks to the Author)
The authors report a carboxyl-functionalized black phosphorene-modified surface plasmon resonance sensor enabled by built-in electric field enhancement, which achieves attomolar-level and highly selective detection of Pb2+. The research topic is timely and of great significance. The integration of interfacial optical enhancement and oxygen-containing

recognition sites possesses distinct scientific appeal. This manuscript is of high academic quality with evident originality, showing promising application prospects in label-free heavy metal ion detection. I thus recommend its publication after appropriate revisions.

1. The use of "selectivity" and "specificity" should be clarified. The manuscript shows a much stronger response toward Pb2+ than toward Cd2+, Cu2+, Zn2+, Ni2+, Ca2+, and Mg2+. The authors should use the terms "selectivity" and "specificity" carefully and consistently.
2. A mixed-ion interference experiment is suggested to further support the selectivity study. The current selectivity data are impressive. To further support the practical selectivity of the SPR+BP-COOH sensor, the authors are encouraged to add a mixed-ion interference experiment, in which Pb2+ is detected in the presence of common interfering ions.
3. More details on ultralow-concentration preparation would be helpful. The authors have described the stepwise dilution procedure. To further support the 10^-19 M detection claim, it would be useful to briefly clarify container pretreatment, water purity, and whether the ultralow-concentration Pb2+ solutions were freshly prepared before measurement.
4. The baseline definition should be clarified. The manuscript defines Δθ relative to pure water. The authors should briefly specify whether a pure-water baseline was measured before each ion-sensing experiment and whether the same baseline procedure was applied to Pb2+ and competing-ion measurements.
5. The LOD comparison in Figure 6 should be described more clearly. I noticed that the LOD values reported in different sensing systems may use different units or concentration expressions. If the authors have converted these values into the same unit for comparison, this should be clearly stated in the figure caption or main text to make the comparison more transparent and rigorous.

(Remarks on code availability)

Reviewer #3

(Remarks to the Author)

The work by Yang et al. reports an interesting approach to ultrasensitive Pb2+ detection by engineering a carboxyl-functionalized black phosphorene layer onto a prism-coupled Au-film SPR platform. this sensor achieves an ultralow detection 27 limit of 10–19 M, 13 orders of magnitude lower than that of bare Au-SPR, and exhibits outstanding selectivity against Cd2+, Cu2+, Zn2+, Ni2+, Ca2+, and Mg2+. The topic is of considerable relevance to the analytical and environmental sensing communities. The paper is generally well structured and the motivation is clearly articulated. However, the manuscript in its present form does not provide sufficient experimental validation and analytical rigor to fully support several of the central claims. The following suggestions/questions should be carefully addressed by the authors.

1. The built-in electric field mechanism is not directly supported. The manuscript mainly relies on an energy-level schematic, but provides no direct evidence to verify the proposed Au/BP interfacial field. The authors should supply a physically rigorous explanation, grounded in SPR optics, for how an interfacial field would enhance angular sensitivity.
2. The selectivity assessment in Fig. 5b and 5c is based exclusively on single-ion measurements and therefore does not provide a rigorous evaluation of selectivity under competitive conditions. The authors should perform competitive adsorption experiments, for example by measuring Pb2+ response in the presence of relevant interfering ions (e.g., Cu2+, Cd2+). In addition, the preferential response toward Pb2+ is currently attributed only to carboxylate coordination, which is insufficient to explain the markedly weaker response to other divalent ions. Stronger chemical evidence, binding analysis, or mechanistic support is therefore needed to substantiate the proposed selectivity mechanism.
3. The oxidative degradation of BP under ambient and aqueous conditions is a well-recognized challenge. Although the authors suggest that PEI/PAA functionalization improves BP stability, no time-dependent stability data are provided. The authors should therefore include systematic stability tests, such as the sensing response of SPR+BP-COOH chips after storage for days to weeks under relevant conditions, together with XPS or Raman characterization to evaluate possible progressive P oxidation.
4. All sensing experiments were conducted in pure water, which is insufficient to support the claim of “ultrarace heavy-metal monitoring in environmental and food-safety applications.” The authors should validate the platform in representative real matrices using spiking-and-recovery tests and report the recovery rates.
5. The LOD is defined as the lowest concentration at which the reflectance curve is distinguishable beyond the instrumental drift range, which does not follow the standard IUPAC criterion of LOD = 3σ/k. The authors should justify why the conventional IUPAC definition was not adopted, explain the basis for using the current criterion instead, and consider whether this choice affects the scientific rigor and comparability of the reported detection limit.
6. The optical simulation is oversimplified and does not match the experiment well. The optical simulation is based on a simplified transfer-matrix model of a bare Au/BP bilayer, in which the polymer-functionalized BP-COOH layer and its actual optical properties are not included, and the simulated optimal thickness is inconsistent with the experimental thickness, which needs explanation.
7. A quantitative inconsistency exists between the reported sensitivity enhancement and LOD improvement. The angular sensitivity increases only from 132 to 281 deg/RIU, whereas the LOD is claimed to improve from 10^{-6} M to 10^{-19} M. The authors should provide explanation for how such a modest optical gain and the BP-COOH affinity could together yield a 13-order-of-magnitude reduction in LOD.
8. The experimental protocol states that the SPR signal is collected 30 s after static sample introduction at 10-19 M in a 400 μL cell. Classical diffusion-limited transport theory (Squires et al., Nature Biotechnology, 2008, DOI: https://www.x-mol.com/paperRedirect/1377170) predicts that analyte arrival at a planar surface under such conditions would require hours to days. The authors should clarify whether a non-classical transport or local preconcentration mechanism contributes to the sensing process and substantiate this possibility through quantitative kinetic analysis, transport modeling, or simulations of interfacial ion enrichment.

(Remarks on code availability)

Version 1:

Reviewer comments:

Reviewer #1

(Remarks to the Author)
The authors have carefully addressed all of my previous comments and significantly improved the manuscript. The additional experiments and discussion substantially strengthen the manuscript and support the main conclusions. I have no further comments and recommend publication in its present form.

Reviewer #2

(Remarks to the Author)
The authors have clearly clarify my concerns.

Reviewer #3

(Remarks to the Author)
The authors made meaningful and significant revisions to address reviewer comments, which has substantially strengthened the manuscript, particularly the addition of independent-chip statistics, the mixed-ion/drinking-water validation, and the refractive-index calibration comparing bare Au, Au+Graphene, Au+MoS2, Au+BP and Au+BP-COOH. Most of my original concerns have been adequately addressed. Three points remain that require further clarification or substantiation before the manuscript is suitable for publication.

1. The built-in electric field is still not fully supported. The revision relies on an energy-level schematic (Fig. 4c, d) without reporting actual work-function values for Au and BP, and no XPS data are provided to verify interfacial charge transfer between Au and BP. Corroborating spectroscopic and work-function measurements should be included as direct empirical support.

2. The discussion on selectivity mechanism remains insufficient (Line 278-293). The HSAB theory can rationalize the superior binding performance of Pb2+ relative to hard hydrated Ca2+ and Mg2+, yet it fails to clarify why the material exhibits far stronger preference for Pb2+ against Cu2+, Cd2+, Zn2+ and Ni2+. A more targeted chemical rationale is required to rationalize the ion selectivity across the full range of tested metal ion species.

3. The Day 1 to Day 7 storage data in Fig. S24 show an overall Δθ decay of ~65%, which seems high for a sensor intended for practical ultratrace monitoring. It is recommended to benchmark the observed decay rate against documented stability metrics of analogous 2D-material-based SPR sensors. The fundamental origin of this signal attenuation is suggested to be explicitly elucidated and propose feasible strategies for improving the long-term storage stability of the sensing chips.

Version 2:

Reviewer comments:

Reviewer #3

(Remarks to the Author)
The authors have satisfactorily answered to my comments and requests. For what concerns me, the manuscript can be accepted.

Open Access This Peer Review File is licensed under a Creative Commons Attribution 4.0 International License, which permits use, sharing, adaptation, distribution and reproduction in any medium or format, as long as you give appropriate credit to the original author(s) and the source, provide a link to the Creative Commons license, and indicate if changes were made.
In cases where reviewers are anonymous, credit should be given to 'Anonymous Referee' and the source.
The images or other third party material in this Peer Review File are included in the article's Creative Commons license, unless indicated otherwise in a credit line to the material. If material is not included in the article's Creative Commons license and your intended use is not permitted by statutory regulation or exceeds the permitted use, you will need to obtain permission directly from the copyright holder.
To view a copy of this license, visit https://creativecommons.org/licenses/by/4.0/

Manuscript ID: NCOMMS-26-039241A

Title: Built-in electric field-boosted attomolar Pb^{2+} sensing on carboxylated black phosphorene

Reviewer 1

Response to Reviewer 1

We sincerely thank Reviewer 1 for the careful evaluation of our manuscript and for the insightful and constructive comments. The reviewer's concerns regarding the LOD definition, the statistical reliability of the 10^{-19} M Pb^{2+} response, possible contamination during ultralow-concentration preparation, BP-COOH stability, regeneration behavior, sample naming, comparison with other two-dimensional overlayers, and the chemical origin of Pb^{2+} selectivity are highly valuable. These comments helped us substantially improve the experimental rigor and clarity of the manuscript. In the revised manuscript, we have clarified the distinguishability criterion and LOD definition, added time-dependent statistics from ten independently prepared Au+BP-COOH chips, introduced error bars based on independent chips, provided additional AFM and Raman characterization, evaluated storage stability and regeneration behavior, renamed the sensing configurations as bare Au and Au+BP-COOH, and strengthened the discussion of the BP-related optical contribution and Pb^{2+} preferential interaction. The detailed responses to each comment are provided below.

Comment 1

Usually, detection limit is defined as a concentration where a signal exceeds 3 times the noise level, while authors defined this with some ambiguity, simply stating that the response exceeds the instrumental error range. Please clarify how the instrumental error range is quantified and how it was used in the calculation of detection limit.

Response:

We thank the reviewer for pointing out the ambiguity in our previous definition of the detection limit. We agree that the basis for the instrumental error range should be clearly quantified. In the revised manuscript, we have clarified that the sensing response was evaluated by the resonance-angle shift $\Delta\theta$, and a response was considered distinguishable when $\Delta\theta$ exceeded 21 mdeg.

This threshold was not arbitrarily selected. It was determined from two experimental factors. First, the angular scanning step of the SPR measurement was 19–21 mdeg. Second, we performed repeated pure-water baseline measurements on the same Au+BP-COOH chip over 50 min. As shown in Fig. S33, the maximum resonance-angle drift during this period was 21 mdeg, and the standard deviation of the resonance angle was 0.00714 deg, corresponding to 7.14 mdeg. Therefore, 3σ is approximately 21.42 mdeg, which is close to both the measured maximum drift and the angular scanning step. Based on this baseline fluctuation and instrumental angular resolution, 21 mdeg was used as the operational distinguishability threshold.

Accordingly, in the revised manuscript, the LOD was defined as the lowest concentration at which the resonance-angle shift $\Delta\theta$ exceeded this 21 mdeg threshold under the corresponding sensing condition. We have revised the Methods section to clarify this criterion and added the corresponding baseline stability experiment in the Supporting Information.

Changes made in the manuscript:

Manuscript, lines 418–424:

"The resonance-angle shift Δθ was used to evaluate the sensing response. A response was considered distinguishable when Δθ exceeded 21 mdeg. This operational distinguishability threshold was selected according to the angular scanning step of the SPR measurement and the baseline stability of Au+BP-COOH in pure water. Repeated pure-water measurements on the same Au+BP-COOH chip over 50 min showed a maximum resonance-angle drift of 21 mdeg and a standard deviation of 0.00714 deg (Fig. S33). The LOD was defined as the lowest concentration at which a distinguishable response was obtained under the corresponding sensing condition."

Supporting Information, lines 158-161:

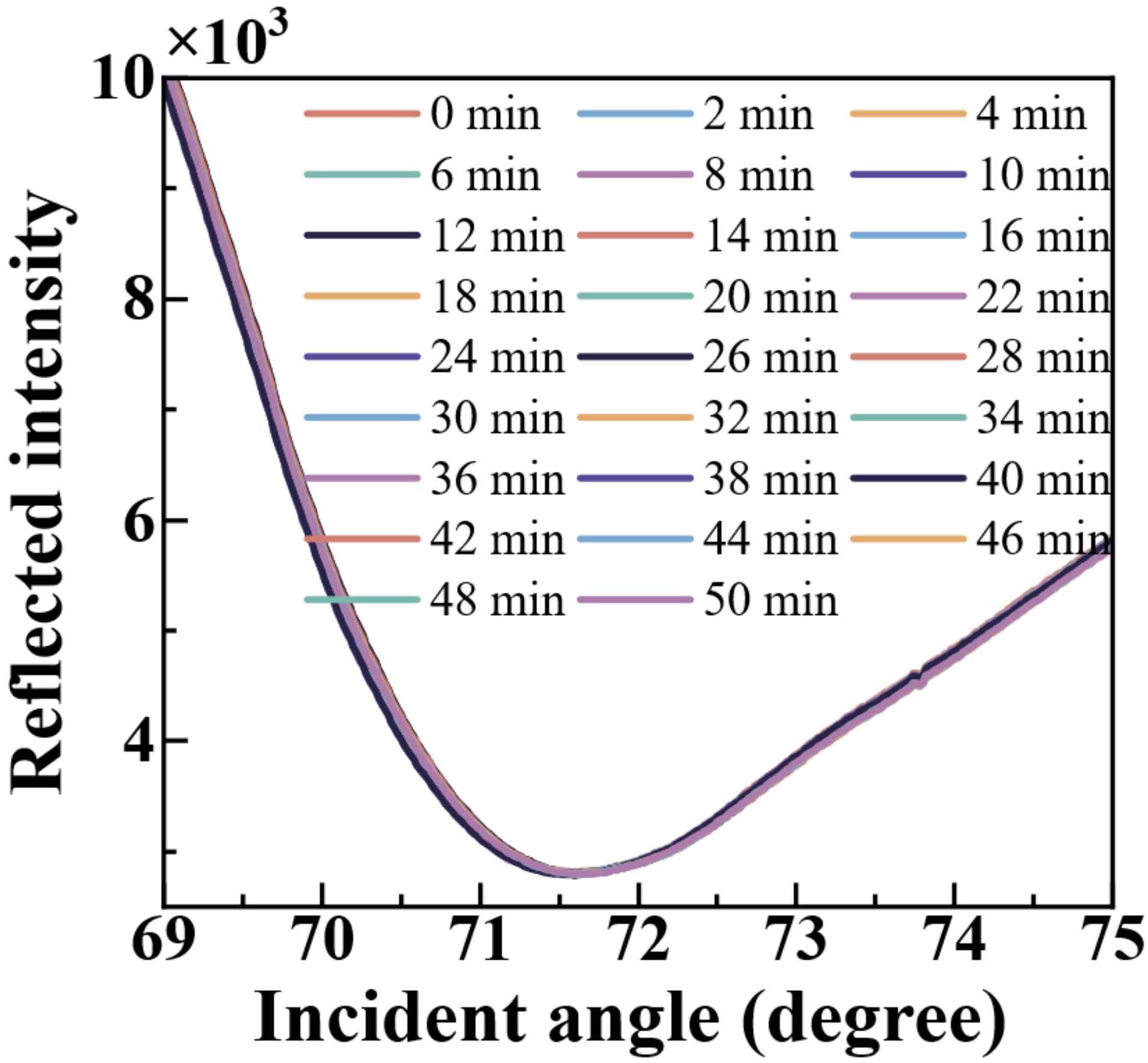

Fig. S33 Baseline stability of Au+BP-COOH in pure water. Angle-scanned reflectance curves recorded on the same Au+BP-COOH chip in pure water at 2 min intervals over 50 min. The maximum resonance-angle drift during the 50 min measurement was 21 mdeg, and the standard deviation of the resonance angle was 0.00714 deg.

Comment 2

The detection limit of 10^{-19} M is quite impressive but questionable at the same time. At this concentration, the 400 μL analyte solution used in this work contains only approximately 24 Pb^{2+} ions on average. Considering the actual adsorption rate onto the sensor surface and its contribution to angular changes, this is essentially a result from an extremely small, even countable, number of

Pb^{2+} ions. Such a super-sensitive claim should be verified more carefully. There are no error bars or repeated independent measurements to support the validity of the sensing data. Also, at such extreme dilutions, how can the authors exclude the possibility of Pb^{2+} contamination from containers or pipette tips during sample preparation and measurement?

Response:

We are very grateful to the reviewer for raising this important concern. We agree that, at 10^{-19} M, the finite number of Pb^{2+} ions in 400 μL solution makes the sensing behavior different from conventional higher-concentration measurements. Therefore, in the revised manuscript, we no longer present the 10^{-19} M response simply as a single deterministic result. Instead, we added time-dependent detection statistics based on ten independently prepared Au+BP-COOH chips to clarify the statistical detection behavior at this ultralow concentration.

Specifically, we measured ten independent Au+BP-COOH chips at 30, 60, 90 and 120 s for Pb^{2+} concentrations from 10^{-19} to 10^{-15} M. The individual sensing results are provided in Figs. S6–S15, and the statistical resonance-angle shifts with error bars are summarized in Fig. S16. The detection statistics are also summarized in Table 1. At 10^{-19} M Pb^{2+}, 1/10, 2/10, 4/10 and 4/10 chips showed distinguishable responses at 30, 60, 90 and 120 s, respectively. This result indicates that 10^{-19} M is located in a time-dependent statistical detection regime, consistent with the reviewer's concern regarding the finite ion number. In contrast, at 10^{-18} M and higher Pb^{2+} concentrations, all ten chips showed distinguishable responses at every detection time. Thus, the revised manuscript now distinguishes the statistical behavior at 10^{-19} M from the consistent detectability observed from 10^{-18} to 10^{-15} M.

We also added error bars to the sensing statistics. For Pb^{2+}, the data in Fig. 5c are from ten independently prepared Au+BP-COOH chips, and the statistical results are shown in Fig. S16. For the other metal ions, the data are from three independently prepared chips, as shown in Figs. S25–S30. These independent-chip statistics directly address the reviewer's concern that the original manuscript lacked repeated independent measurements and error bars.

Regarding possible contamination during ultralow-concentration preparation, we revised the Methods section to describe the dilution procedure more explicitly. All ion solutions were prepared in a clean laboratory. For each target concentration and each dilution step, a fresh container and a fresh pipette tip were used to minimize carryover between concentrations. Containers and pipette tips were not reused, and ultralow-concentration solutions were freshly prepared before measurement. In addition, for each sensing experiment, the response was evaluated relative to the corresponding baseline, and a response was counted as distinguishable only when $\Delta\theta$ exceeded the 21 mdeg threshold established from the baseline stability experiment (Fig. S33). These procedures were added to make the ultralow-concentration measurement and its statistical interpretation clearer.

Changes made in the manuscript:

Manuscript, Table 1, lines 238–240:

"**Table 1.** Time-dependent detection statistics of Pb^{2+} obtained from ten independently prepared Au+BP-COOH chips.

Pb^{2+} concentration	30 s	60 s	90 s	120 s
10^{-19} M	1/10	2/10	4/10	4/10
10^{-18} M	10/10	10/10	10/10	10/10
10^{-17} M	10/10	10/10	10/10	10/10
10^{-16} M	10/10	10/10	10/10	10/10
10^{-15} M	10/10	10/10	10/10	10/10

A chip was counted as distinguishable when the resonance-angle shift Δθ exceeded 21 mdeg. The statistics were obtained from ten independently prepared Au+BP-COOH chips shown in Figs. S6–S15."

Manuscript, Fig. 5, lines 242–252:

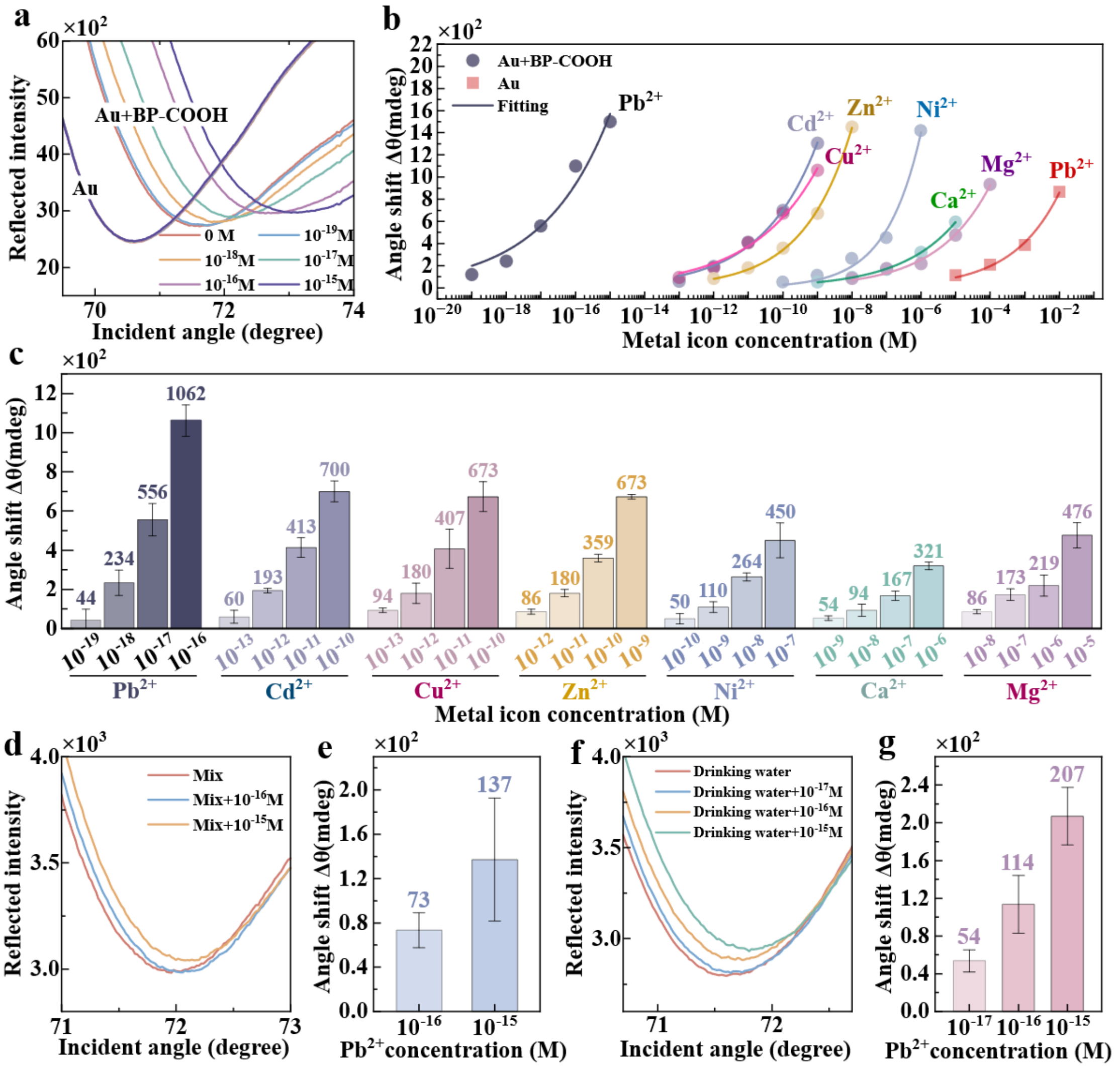

"Fig. 5. Pb^{2+} sensing response of Au+BP-COOH and comparison with other metal ions in pure water, mixed-ion solution, and drinking water. (a) Angle-scanned reflectance curves of Au+BP-COOH measured for Pb^{2+} concentrations of 0, 10^{-19}, 10^{-18}, 10^{-17}, 10^{-16} and 10^{-15} M, together with the reflectance curve of bare Au. **(b)** Resonance-angle shift Δθ as a function of metal-ion concentration for Au+BP-COOH and bare Au. The data points are mean values from the corresponding statistical results in c and Fig. S4, and the solid lines represent nonlinear fitting curves.

(c) Resonance-angle shift Δθ values of Au+BP-COOH measured at different concentrations of Pb^{2+}, Cd^{2+}, Cu^{2+}, Zn^{2+}, Ni^{2+}, Ca^{2+} and Mg^{2+}. The Pb^{2+} data are from the ten independently prepared Au+BP-COOH chips shown in Figs. S6–S15 and statistically summarized in Fig. S16, and the data for Cd^{2+}, Cu^{2+}, Zn^{2+}, Ni^{2+}, Ca^{2+} and Mg^{2+} are from the three independently prepared Au+BP-COOH chips shown in Figs. S25–S30. **(d)** Angle-scanned reflectance curves of Au+BP-COOH measured in Mix, Mix + 10^{-16} M Pb^{2+} and Mix + 10^{-15} M Pb^{2+}. **(e)** Net resonance-angle shift Δθ as a function of Pb^{2+} concentration in the mixed-ion solution. The statistical data are from Fig. S31. **(f)** Angle-scanned reflectance curves of Au+BP-COOH measured in drinking water, drinking water + 10^{-17} M Pb^{2+}, drinking water + 10^{-16} M Pb^{2+} and drinking water + 10^{-15} M Pb^{2+}. **(g)** Net resonance-angle shift Δθ as a function of Pb^{2+} concentration in drinking water. The statistical data are from Fig. S32."

Manuscript, lines 265–272:

"To examine the statistical detection behavior of Au+BP-COOH at ultralow Pb^{2+} concentrations, time-dependent sensing measurements were performed on ten independently prepared Au+BP-COOH chips. The detection statistics are summarized in Table 1, and the corresponding individual sensing results are provided in Figs. S6–S15. At 10^{-19} M Pb^{2+}, 1/10, 2/10, 4/10 and 4/10 chips show distinguishable responses at 30, 60, 90 and 120 s, respectively, indicating that this concentration is located in a time-dependent statistical detection regime. In contrast, at 10^{-18} M and higher Pb^{2+} concentrations, all ten chips show distinguishable responses at each detection time. The statistical resonance-angle shifts obtained from these ten chips are summarized in Fig. S16. These results show the time-dependent detectability of Au+BP-COOH at 10^{-19} M and the consistent detectability from 10^{-18} to 10^{-15} M.**"**

Manuscript, lines 398–410:

"SPR measurements were performed on a Dynechem SPR system using 650 nm incident light at 25 °C. The angular scanning range was 65°–80°, and the angular step was 19–21 mdeg. For each measurement, 400 μL of sample solution was introduced into the flow cell at a flow rate of 200 μL/min. The resonance angle was directly read from the angle corresponding to the minimum reflectance point without curve fitting.

For Pb^{2+} time-dependent sensing measurements, the SPR response was recorded at 30, 60, 90 and 120 s. Unless otherwise specified, the resonance-angle shift Δθ used for statistical comparison was extracted at 120 s. For pure-water sensing measurements and single-ion sensing measurements, Δθ was defined relative to the corresponding pure-water baseline. For mixed-ion and drinking-water measurements, the net Δθ was obtained by subtracting the corresponding matrix baseline.

For the time-dependent Pb^{2+} detection statistics, ten independently prepared Au+BP-COOH chips were measured from pure water to increasing Pb^{2+} concentrations. For single-ion selectivity, mixed-ion interference and drinking-water measurements, three independently prepared Au+BP-COOH chips were used unless otherwise specified. Error bars represent the standard deviation from independently prepared chips.**"**

Manuscript, lines 412–417:

"All ion solutions were prepared in the clean laboratory. The purchased single-element standard solutions were first converted to 10^{-2} M stock solutions and then diluted stepwise by a factor of 10 using pure water to obtain the required concentration series. The pH of the test solutions remained at 6.3, and all ions were measured under the same matrix conditions. For each target concentration and each dilution step, a fresh container and a fresh pipette tip were used to minimize carryover between concentrations. Containers and pipette tips were not reused, and the ultralow-concentration

solutions were freshly prepared before measurement.”

Manuscript, lines 419–424:

“The resonance-angle shift Δθ was used to evaluate the sensing response. A response was considered distinguishable when Δθ exceeded 21 mdeg. This operational distinguishability threshold was selected according to the angular scanning step of the SPR measurement and the baseline stability of Au+BP-COOH in pure water. Repeated pure-water measurements on the same Au+BP-COOH chip over 50 min showed a maximum resonance-angle drift of 21 mdeg and a standard deviation of 0.00714 deg (Fig. S33). The LOD was defined as the lowest concentration at which a distinguishable response was obtained under the corresponding sensing condition.”

Supporting Information, lines 41–80:

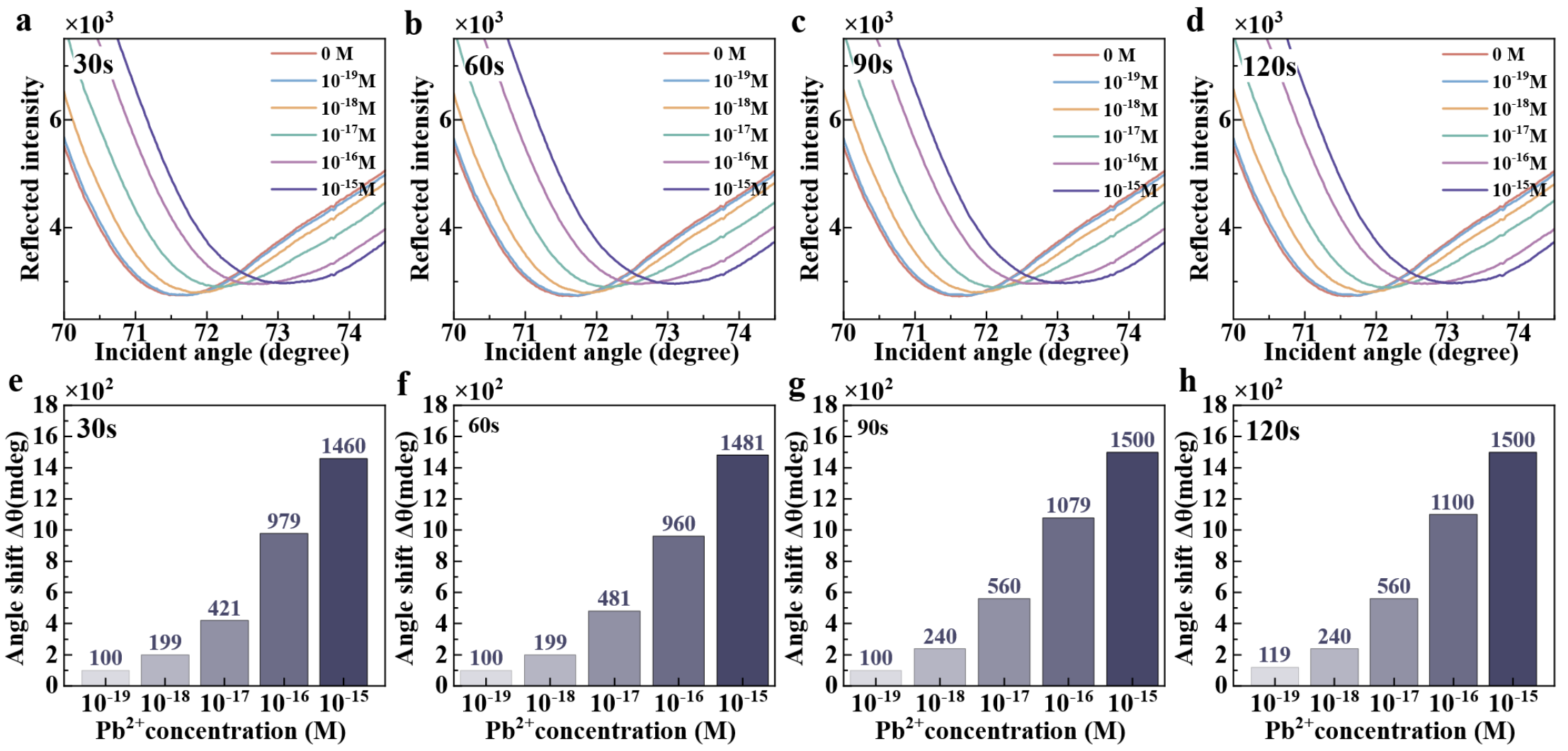

“Fig. S6 Time-dependent Pb^{2+} sensing results obtained from one Au+BP-COOH chip. (a–d) Angle-scanned reflectance curves measured for Pb^{2+} concentrations of 0, 10^{-19}, 10^{-18}, 10^{-17}, 10^{-16} and 10^{-15} M at 30, 60, 90 and 120 s, respectively. (e–h) Corresponding resonance-angle shift Δθ values at 30, 60, 90 and 120 s, respectively.”

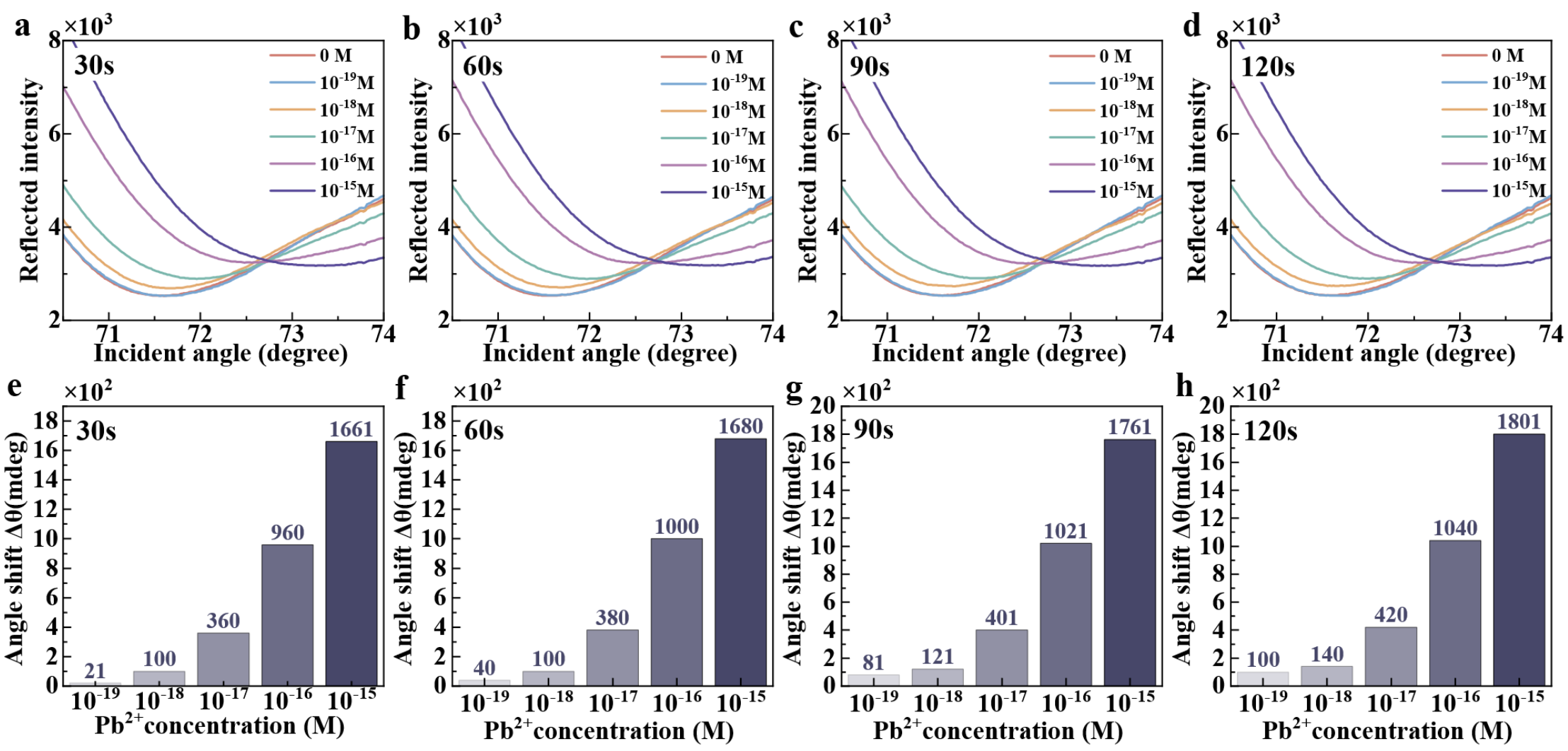

“Fig. S7 Time-dependent Pb^{2+} sensing results obtained from one Au+BP-COOH chip. (a–d) Angle-scanned reflectance curves measured for Pb^{2+} concentrations of 0, 10^{-19}, 10^{-18}, 10^{-17}, 10^{-16} and 10^{-15} M at 30, 60, 90 and 120 s, respectively. (e–h) Corresponding resonance-angle shift Δθ values at 30, 60, 90 and 120 s, respectively.”

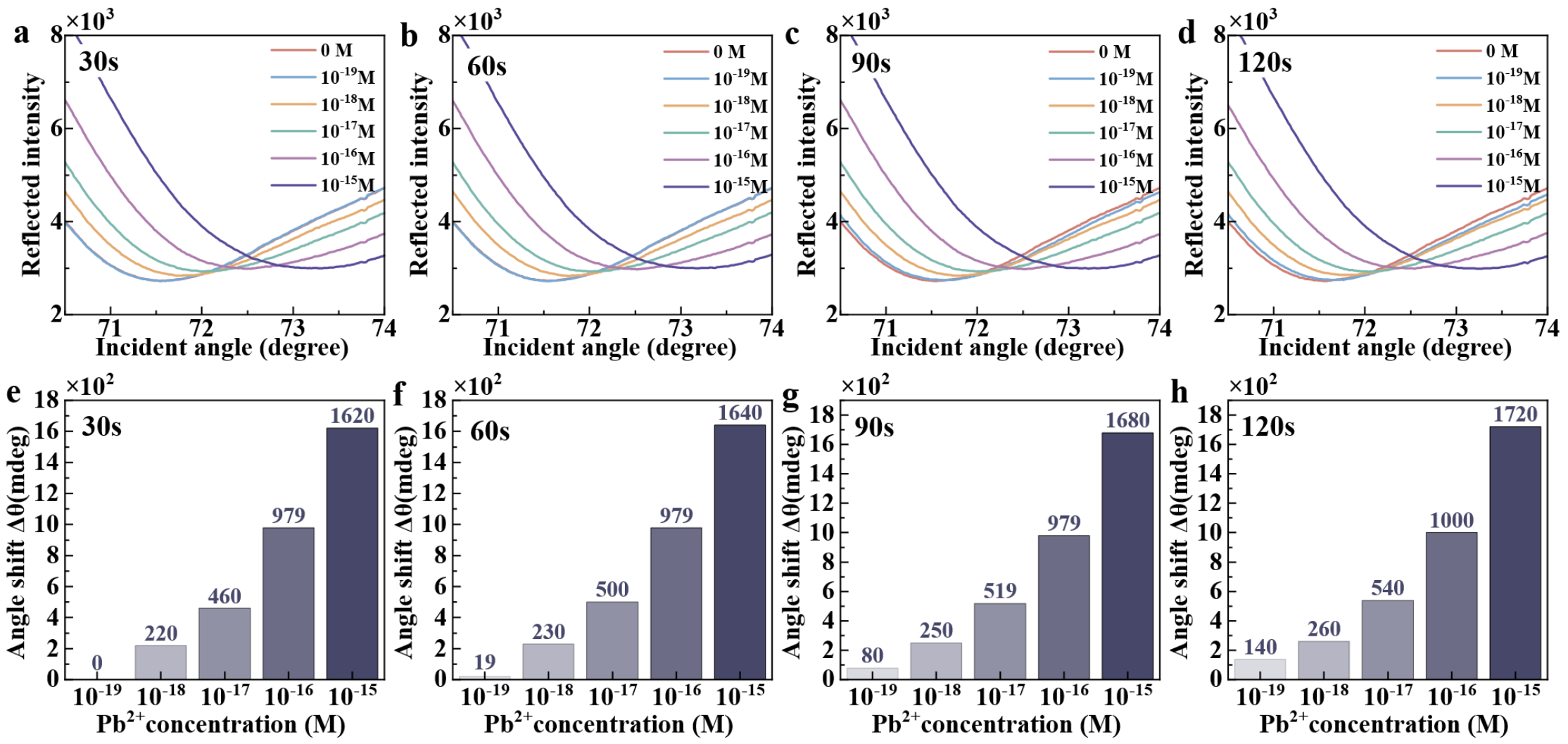

"Fig. S8 Time-dependent Pb^{2+} sensing results obtained from one Au+BP-COOH chip. (a–d) Angle-scanned reflectance curves measured for Pb^{2+} concentrations of 0, 10^{-19}, 10^{-18}, 10^{-17}, 10^{-16} and 10^{-15} M at 30, 60, 90 and 120 s, respectively. **(e–h)** Corresponding resonance-angle shift Δθ values at 30, 60, 90 and 120 s, respectively."

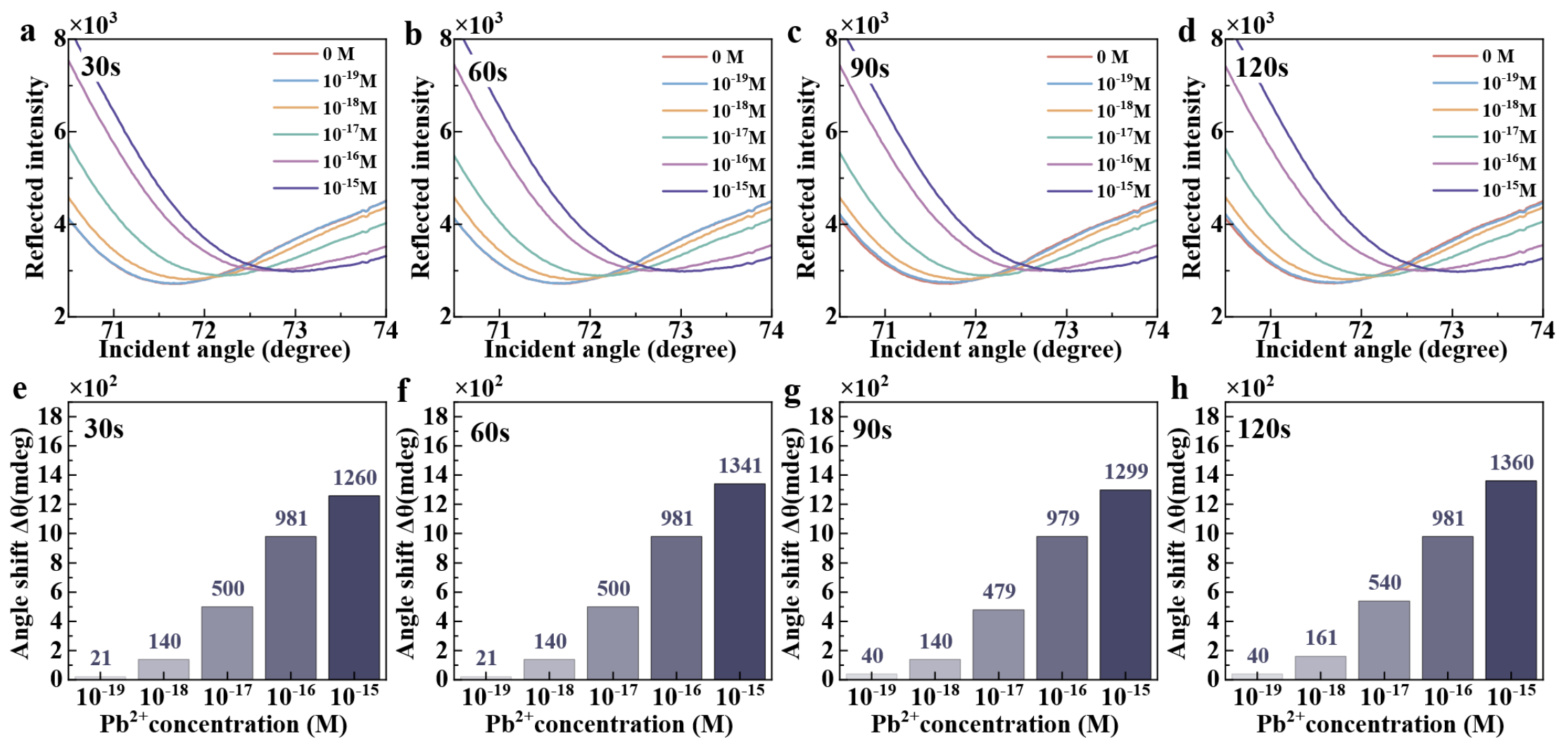

"Fig. S9 Time-dependent Pb^{2+} sensing results obtained from one Au+BP-COOH chip. (a–d) Angle-scanned reflectance curves measured for Pb^{2+} concentrations of 0, 10^{-19}, 10^{-18}, 10^{-17}, 10^{-16} and 10^{-15} M at 30, 60, 90 and 120 s, respectively. **(e–h)** Corresponding resonance-angle shift Δθ values at 30, 60, 90 and 120 s, respectively."

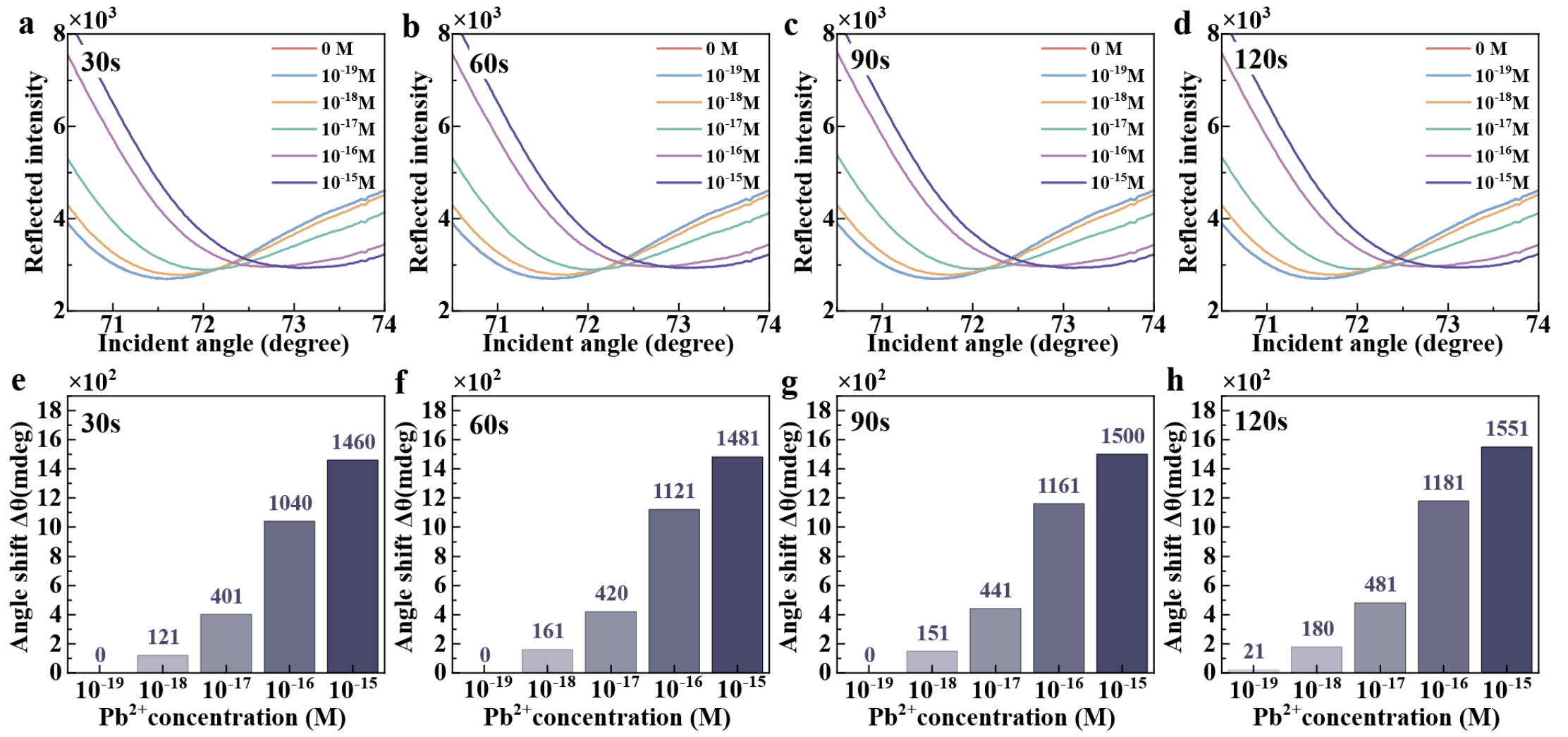

“Fig. S10 Time-dependent Pb^{2+} sensing results obtained from one Au+BP-COOH chip. (a–d) Angle-scanned reflectance curves measured for Pb^{2+} concentrations of 0, 10^{-19}, 10^{-18}, 10^{-17}, 10^{-16} and 10^{-15} M at 30, 60, 90 and 120 s, respectively. **(e–h)** Corresponding resonance-angle shift $\Delta\theta$ values at 30, 60, 90 and 120 s, respectively.”

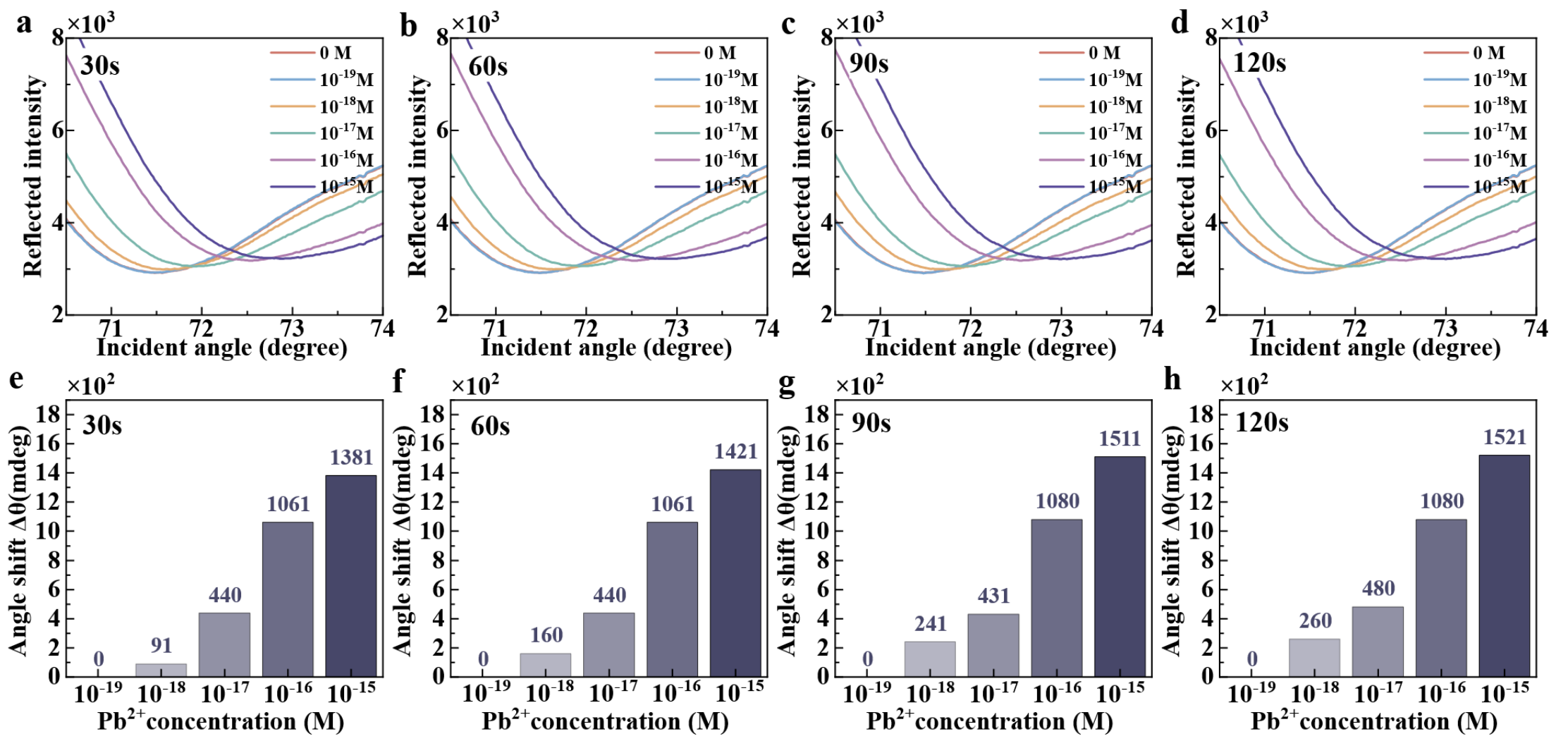

“Fig. S11 Time-dependent Pb^{2+} sensing results obtained from one Au+BP-COOH chip. (a–d) Angle-scanned reflectance curves measured for Pb^{2+} concentrations of 0, 10^{-19}, 10^{-18}, 10^{-17}, 10^{-16} and 10^{-15} M at 30, 60, 90 and 120 s, respectively. **(e–h)** Corresponding resonance-angle shift $\Delta\theta$ values at 30, 60, 90 and 120 s, respectively.”

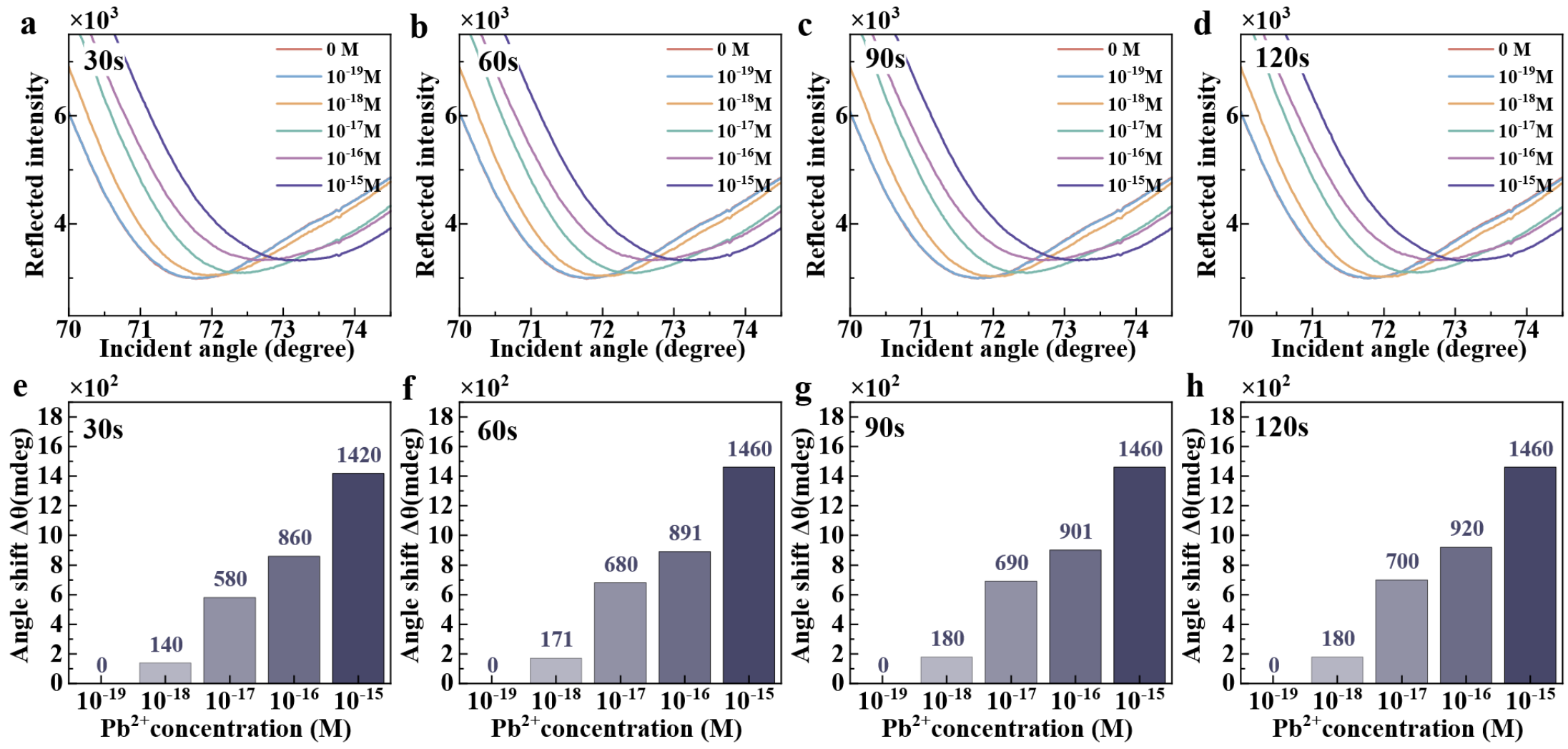

“Fig. S12 Time-dependent Pb^{2+} sensing results obtained from one Au+BP-COOH chip. (a–d) Angle-scanned reflectance curves measured for Pb^{2+} concentrations of 0, 10^{-19}, 10^{-18}, 10^{-17}, 10^{-16} and 10^{-15} M at 30, 60, 90 and 120 s, respectively. **(e–h)** Corresponding resonance-angle shift Δθ values at 30, 60, 90 and 120 s, respectively.”

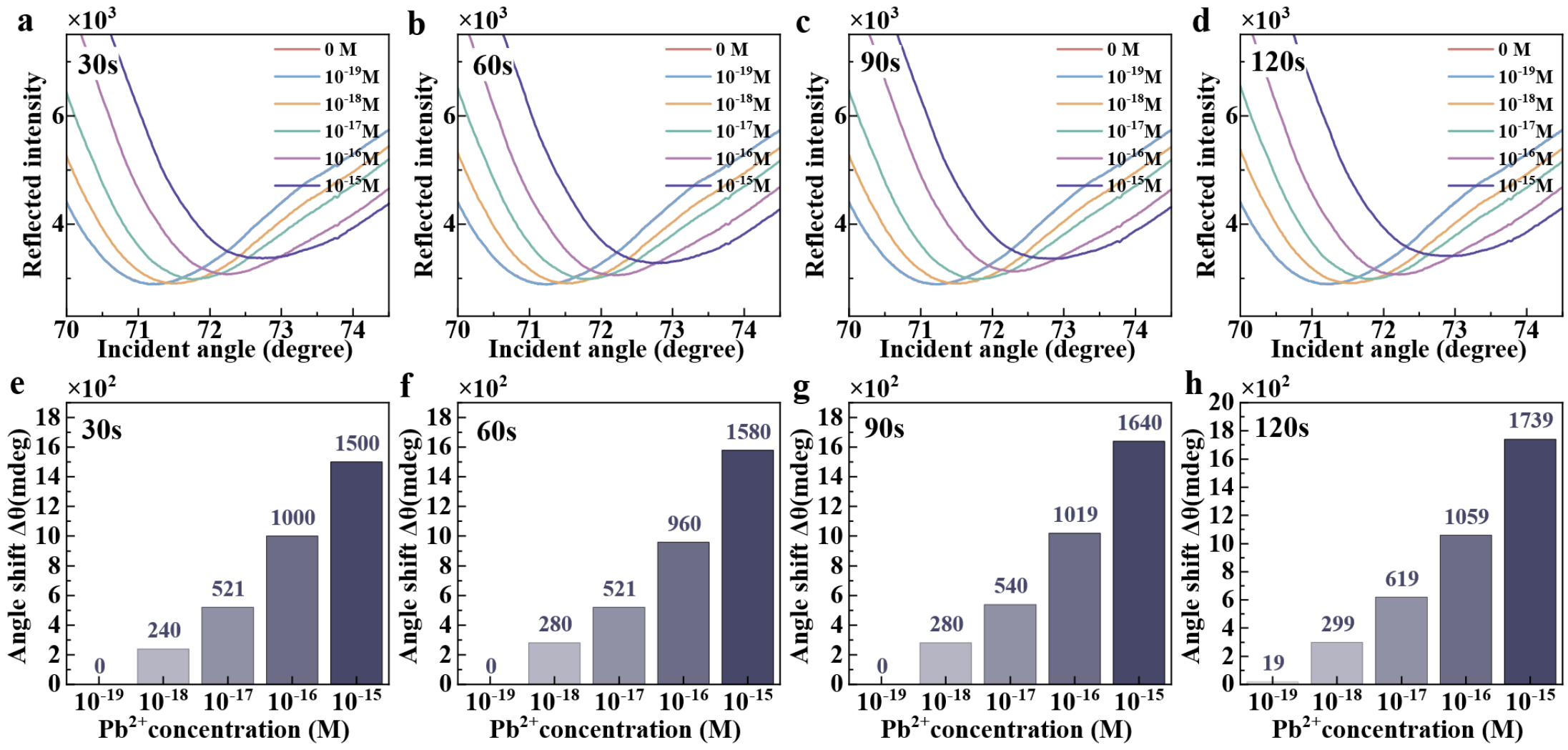

“Fig. S13 Time-dependent Pb^{2+} sensing results obtained from one Au+BP-COOH chip. (a–d) Angle-scanned reflectance curves measured for Pb^{2+} concentrations of 0, 10^{-19}, 10^{-18}, 10^{-17}, 10^{-16} and 10^{-15} M at 30, 60, 90 and 120 s, respectively. **(e–h)** Corresponding resonance-angle shift Δθ values at 30, 60, 90 and 120 s, respectively.”

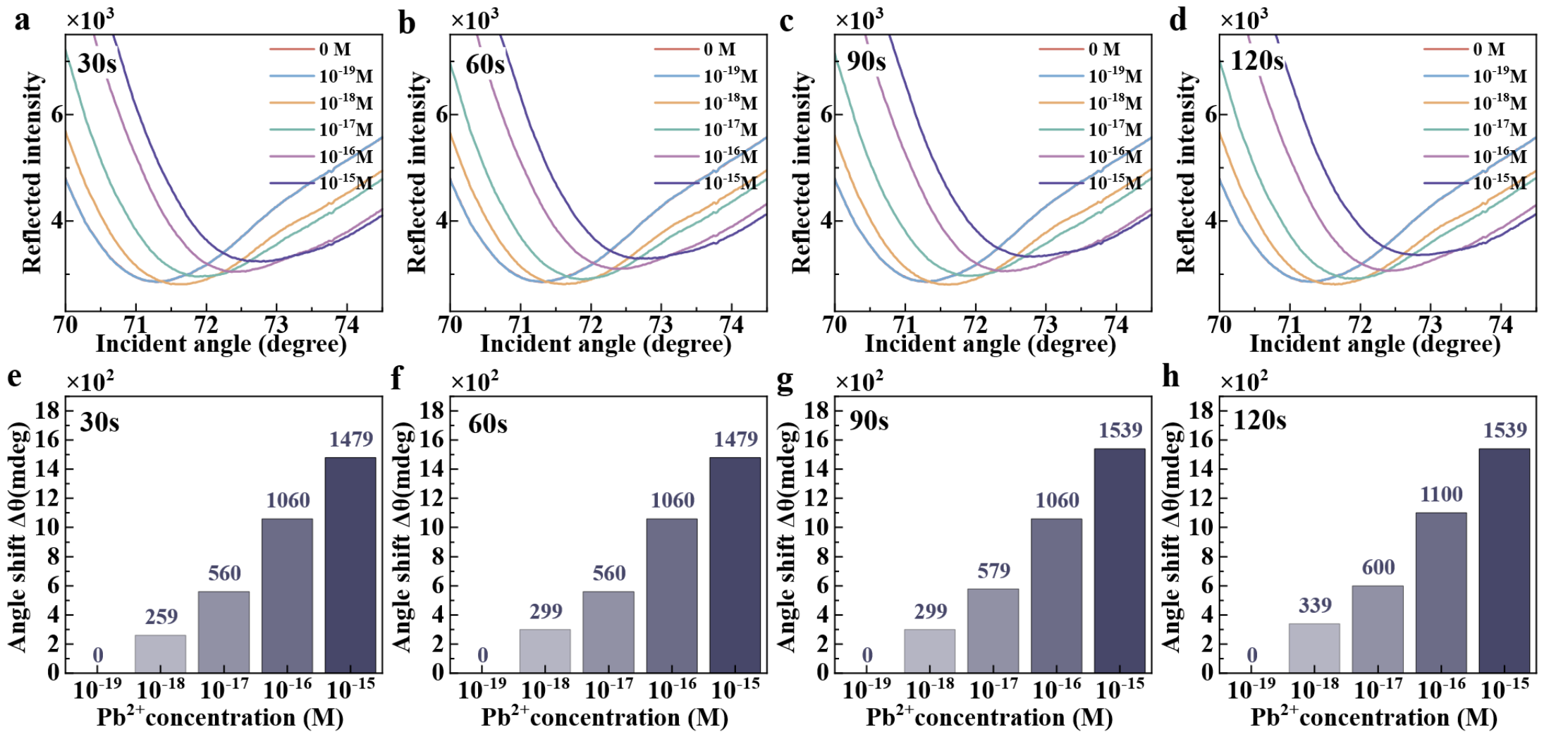

“Fig. S14 Time-dependent Pb^{2+} sensing results obtained from one Au+BP-COOH chip. (a–d) Angle-scanned reflectance curves measured for Pb^{2+} concentrations of 0, 10^{-19}, 10^{-18}, 10^{-17}, 10^{-16} and 10^{-15} M at 30, 60, 90 and 120 s, respectively. **(e–h)** Corresponding resonance-angle shift Δθ values at 30, 60, 90 and 120 s, respectively.”

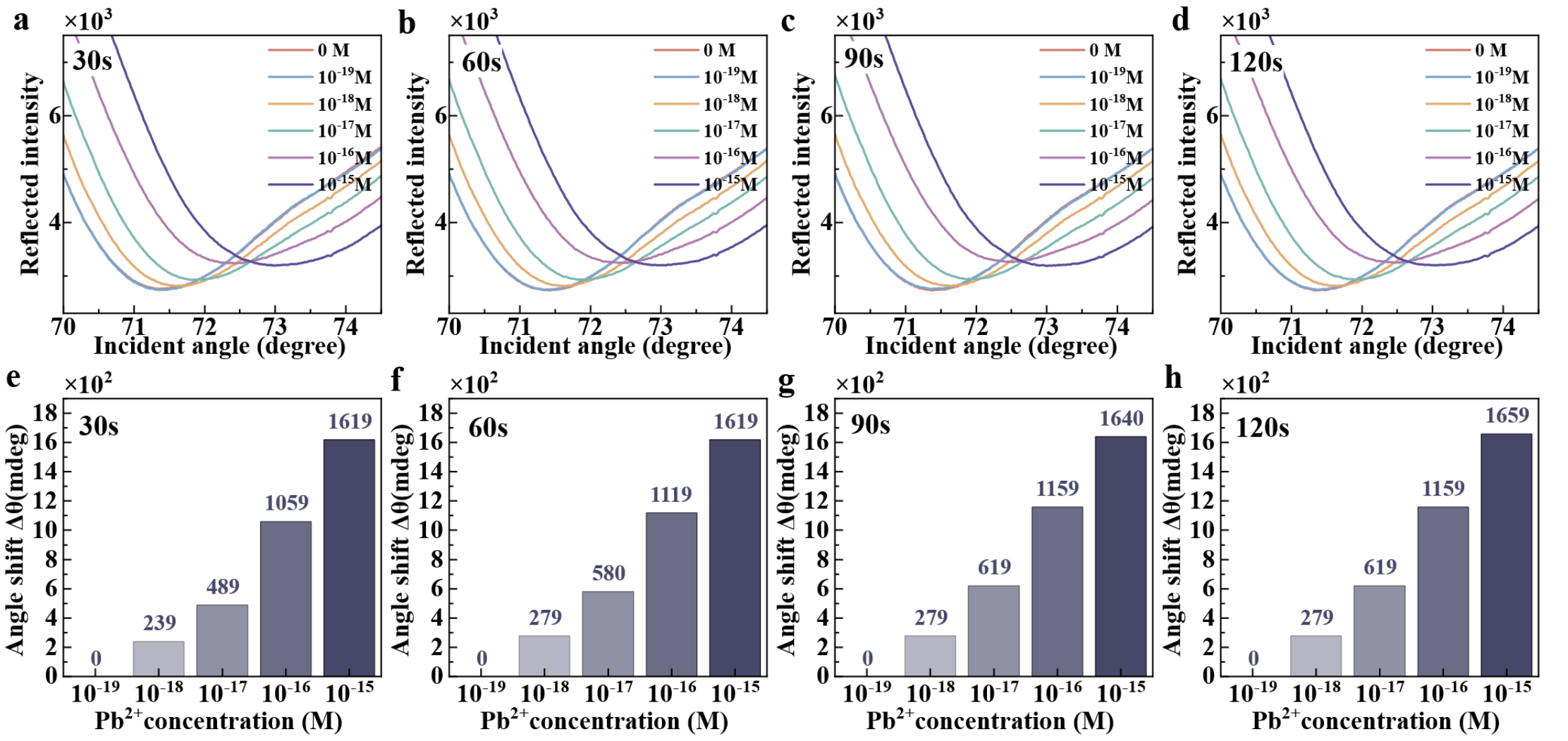

“Fig. S15 Time-dependent Pb^{2+} sensing results obtained from one Au+BP-COOH chip. (a–d) Angle-scanned reflectance curves measured for Pb^{2+} concentrations of 0, 10^{-19}, 10^{-18}, 10^{-17}, 10^{-16} and 10^{-15} M at 30, 60, 90 and 120 s, respectively. **(e–h)** Corresponding resonance-angle shift Δθ values at 30, 60, 90 and 120 s, respectively.”

Supporting Information, lines 81–84:

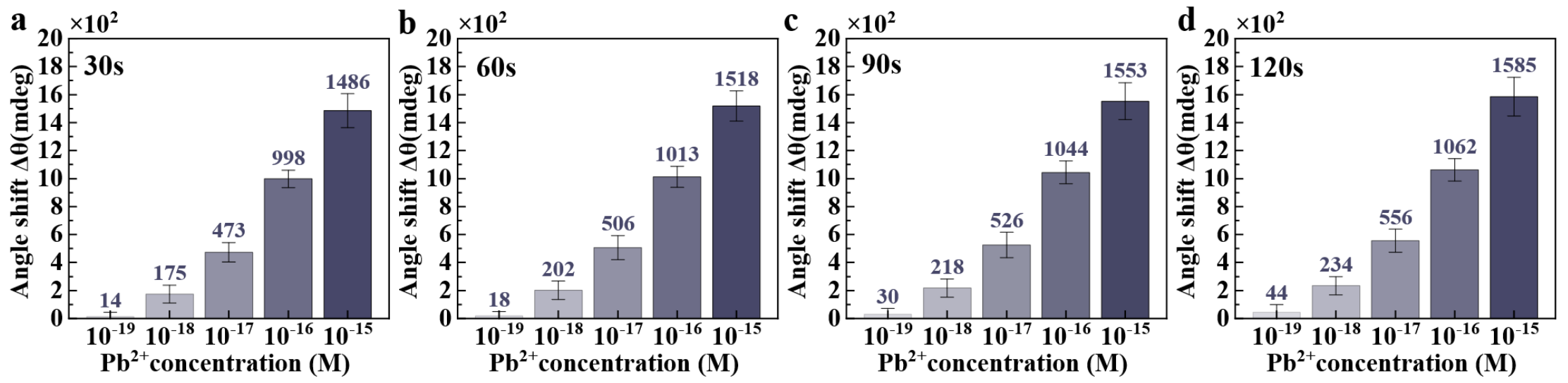

“Fig. S16 Statistical Pb^{2+} sensing results obtained from the ten Au+BP-COOH chips shown in

Figs. S6–S15 at different detection times. (a–d) Resonance-angle shift Δθ as a function of Pb^{2+} concentration at 30, 60, 90 and 120 s, respectively. Error bars are from the ten independently prepared Au+BP-COOH chips shown in Figs. S6–S15.**”**

Supporting Information, lines 158–161:

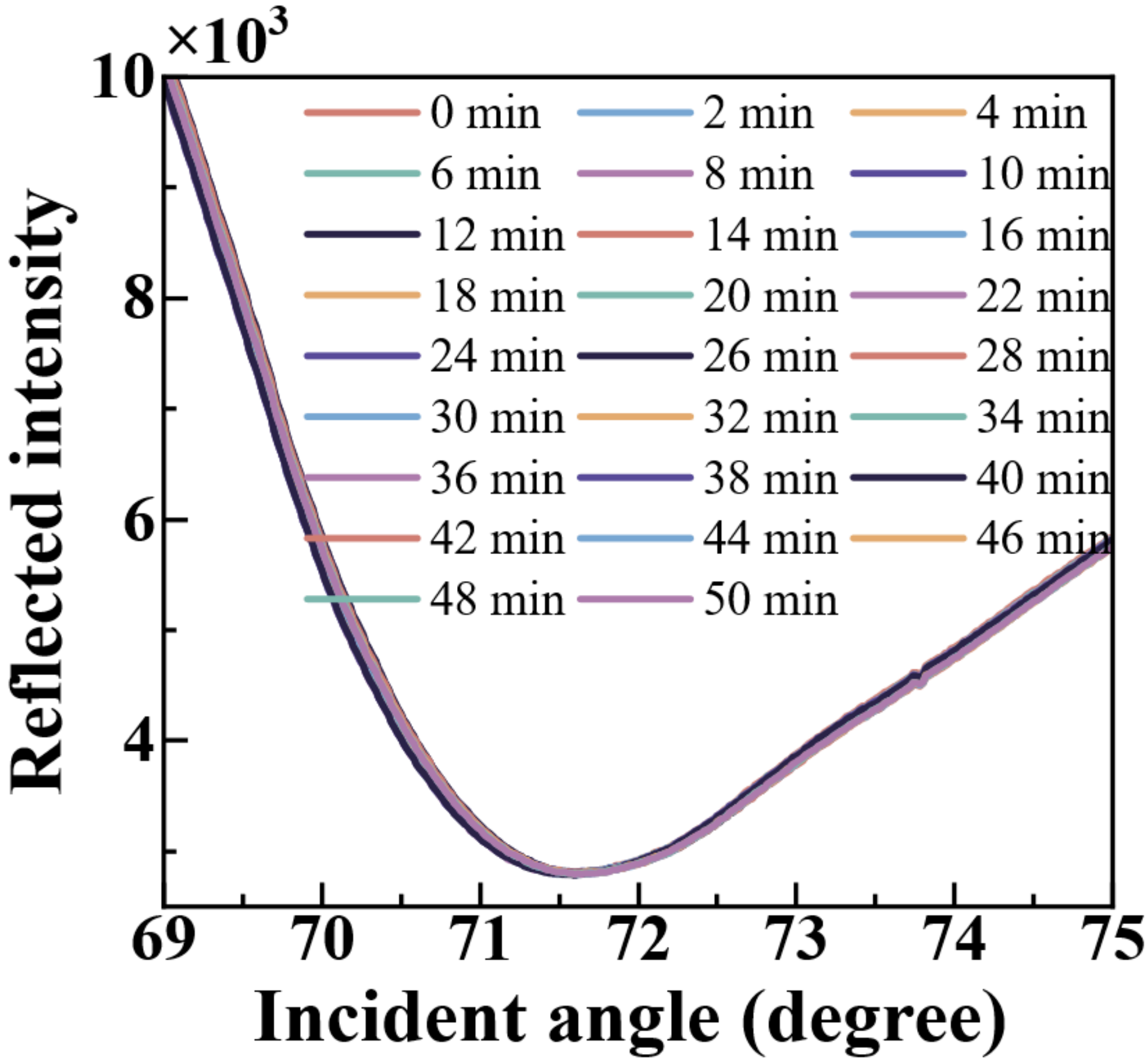

“Fig. S33 Baseline stability of Au+BP-COOH in pure water. Angle-scanned reflectance curves recorded on the same Au+BP-COOH chip in pure water at 2 min intervals over 50 min. The maximum resonance-angle drift during the 50 min measurement was 21 mdeg, and the standard deviation of the resonance angle was 0.00714 deg.**”**

Comment 3

The authors mention the unstable nature of BP in the Introduction. What is the stability of the prepared BP-COOH under the aqueous sensing conditions used in this work, and over what timescale? Any experimental verification, such as repeated SPR measurements or post-exposure surface characterization, would be necessary to support practical applicability.

Response:

We appreciate the reviewer’s important comment on the stability of BP-COOH. We agree that the stability of BP-COOH is essential for evaluating the practical applicability of the sensing interface, especially because BP is known to be sensitive under ambient and aqueous conditions.

In the revised manuscript, we added two types of stability-related experimental evidence. First, we performed Raman measurements of BP-COOH from Day 1 to Day 7 to examine the structural

retention of the BP-COOH functional material during storage. As shown in Fig. S1, the Raman spectra recorded during this period retain the Raman features of BP-COOH, while slight intensity variations are observed. This result indicates that the main structural characteristics of BP-COOH are generally preserved during short-term storage.

Second, we evaluated the sensing stability of Au+BP-COOH chips at different storage time points using 10^{-15} M Pb^{2+} as a representative concentration. This concentration was selected because it gives a clear and reproducible sensing response and is less affected by the finite-ion-number fluctuation observed at 10^{-19} M. The sensing results at different storage time points are provided in Figs. S18–S23, and the statistical results from Day 1 to Day 7 are summarized in Fig. S24. Day 1 corresponds to freshly prepared Au+BP-COOH chips measured immediately after fabrication, while Days 2–7 correspond to chips measured after 1–6 days of storage. The Pb^{2+} response remains distinguishable at all tested storage time points, although the resonance-angle shift gradually decreases with storage time. These results provide direct SPR sensing evidence for the short-term storage stability of Au+BP-COOH under the tested conditions.

We have added these results to the revised manuscript and Supporting Information to clarify the stability of the prepared BP-COOH material and Au+BP-COOH sensing interface over the Day 1–Day 7 timescale.

Changes made in the manuscript:

Manuscript, lines 170–174:

"The structural retention of BP-COOH during storage was further examined by Raman spectroscopy. As shown in Fig. S1, Raman spectra recorded from Day 1 to Day 7 retain the Raman features of BP-COOH during this period. Although slight intensity variations are observed, the BP-related Raman response is generally preserved, indicating that the BP-COOH functional material maintains its main structural characteristics during short-term storage."

Manuscript, lines 301–307:

"The regeneration behavior and storage stability of Au+BP-COOH were also evaluated. The regeneration experiment was performed using 10^{-18} M Pb^{2+} on the Au+BP-COOH chip shown in Fig. S15. As shown in Fig. S17, the Pb^{2+} response remains distinguishable after five regeneration cycles. The storage stability was evaluated using 10^{-15} M Pb^{2+} as a representative concentration. The sensing results obtained at different storage time points are provided in Figs. S18–S23, and the corresponding statistical results from Day 1 to Day 7 are summarized in Fig. S24. The Pb^{2+} response remains distinguishable at all tested storage time points, although the resonance-angle shift gradually decreases with storage time."

Manuscript, lines 437–443:

"The storage stability of Au+BP-COOH chips was evaluated using 10^{-15} M Pb^{2+} as a representative concentration. Day 1 corresponds to freshly prepared Au+BP-COOH chips measured immediately after fabrication. Days 2–7 correspond to Au+BP-COOH chips measured after 1, 2, 3, 4, 5 and 6 days of storage, respectively. For Day 1, the statistical response was obtained from ten independently prepared Au+BP-COOH chips measured at 120 s. For Days 2–7, three independently prepared Au+BP-COOH chips were measured at each storage time point. At each time point, pure water was first measured as the baseline, followed by 10^{-15} M Pb^{2+}. The storage-stability response was defined as the net resonance-angle shift after subtracting the pure-water baseline."

Supporting Information, lines 22–23:

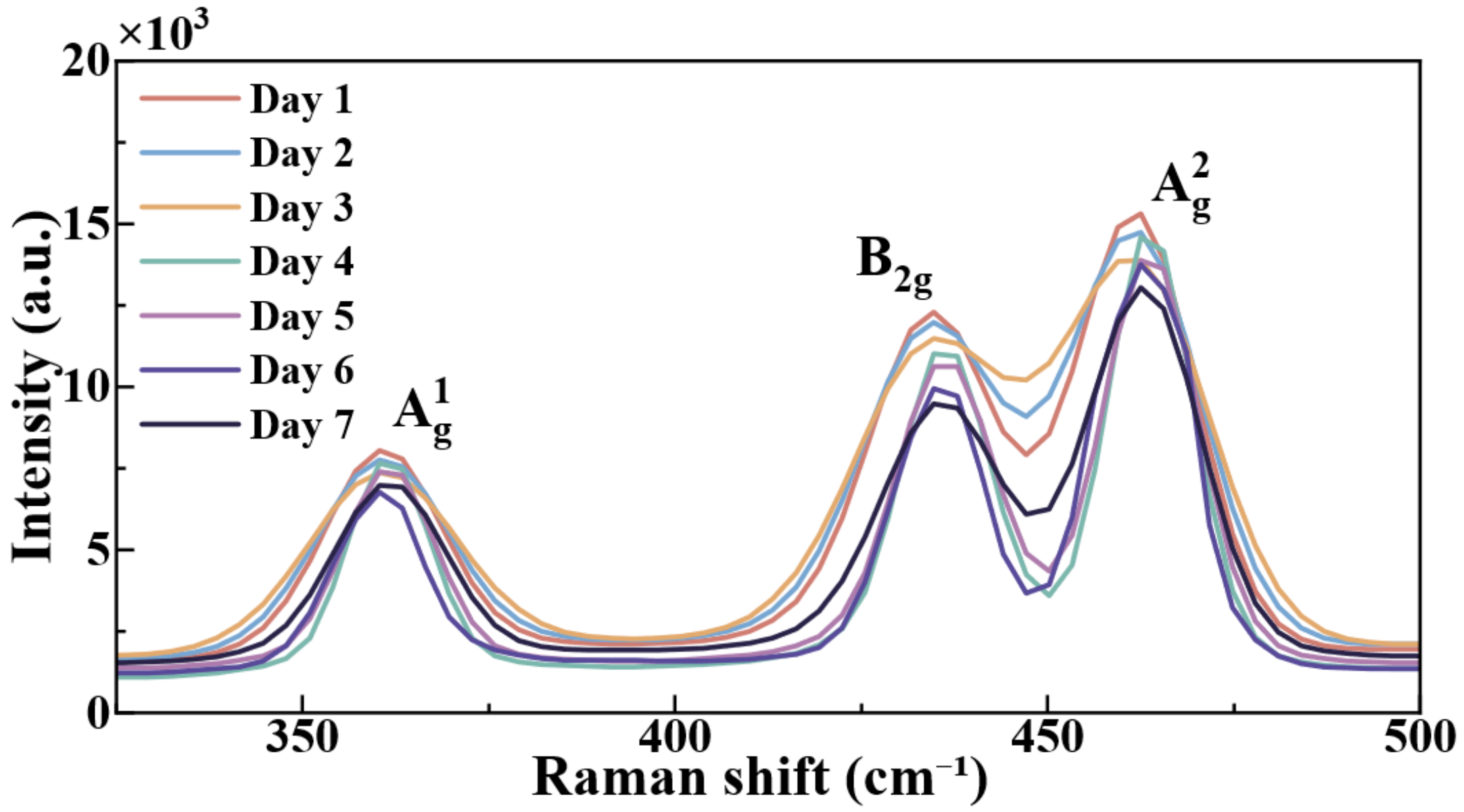

"**Fig. S1 Raman spectra of BP-COOH during storage from Day 1 to Day 7.** Raman spectra of BP-COOH recorded after storage for 1, 2, 3, 4, 5, 6 and 7 days."

Supporting Information, lines 91–106:

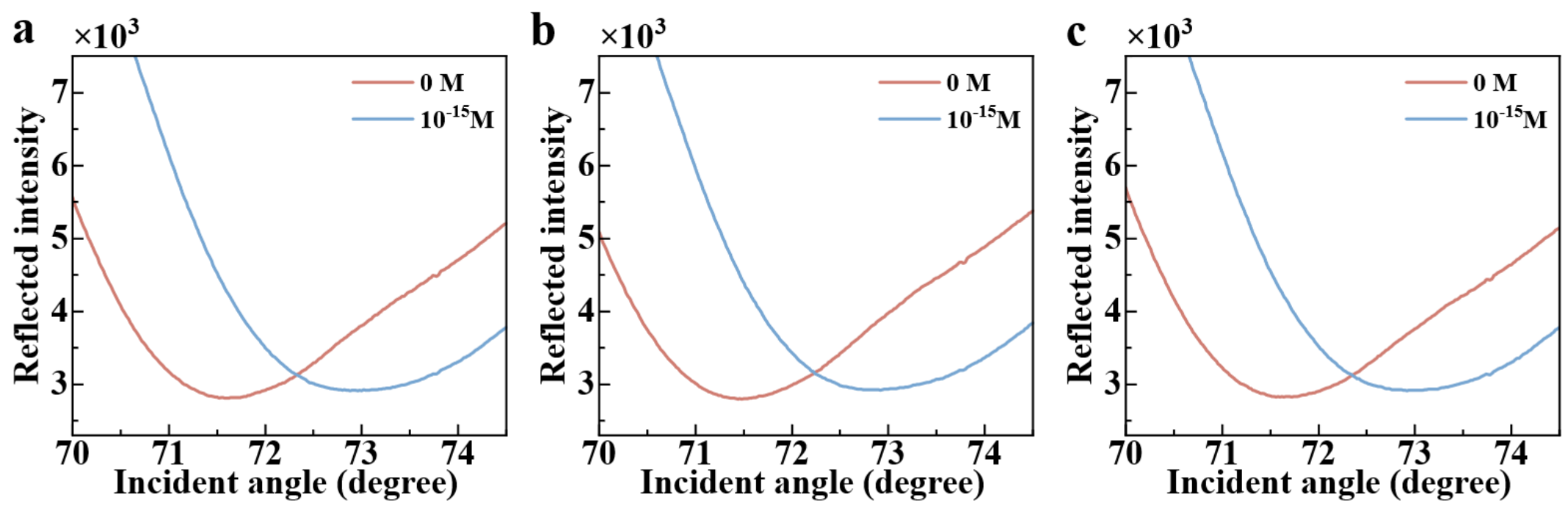

"**Fig. S18 Pb^{2+} sensing results obtained from Au+BP-COOH chips at storage time point Day 2. (a–c)** Angle-scanned reflectance curves of three independently prepared Au+BP-COOH chips measured for 0 M and 10^{-15} M Pb^{2+}. Day 2 corresponds to chips measured after 1 day of storage."

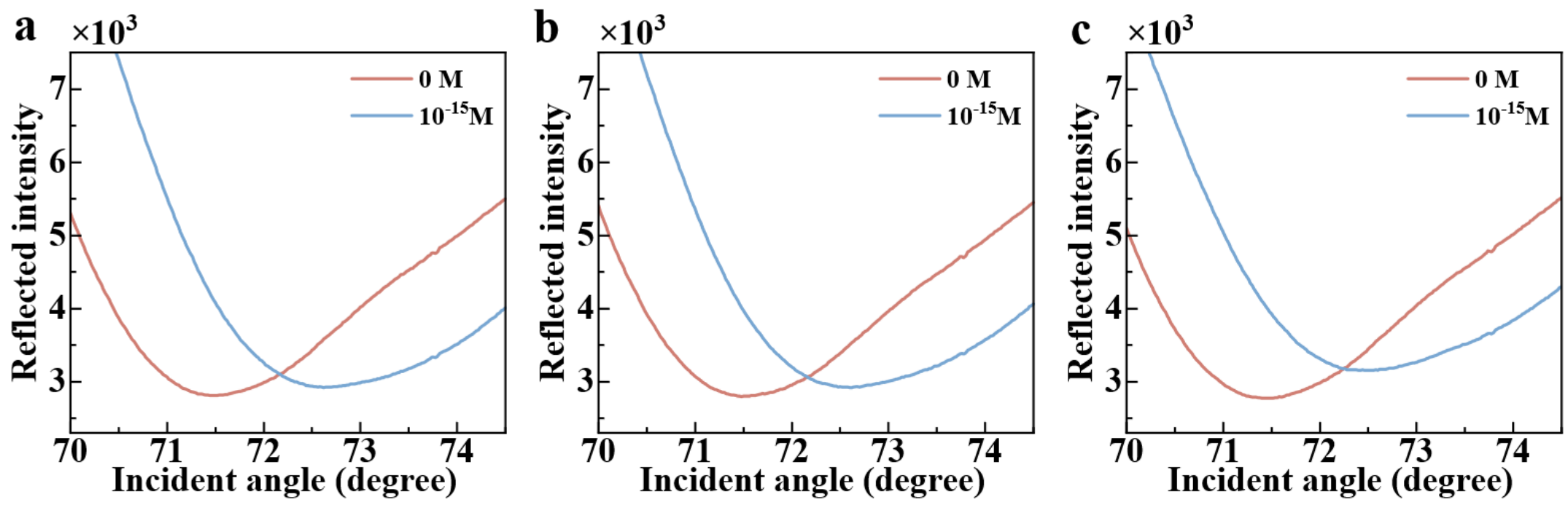

"**Fig. S19 Pb^{2+} sensing results obtained from Au+BP-COOH chips at storage time point Day 3. (a–c)** Angle-scanned reflectance curves of three independently prepared Au+BP-COOH chips measured for 0 M and 10^{-15} M Pb^{2+}. Day 3 corresponds to chips measured after 2 days of storage."

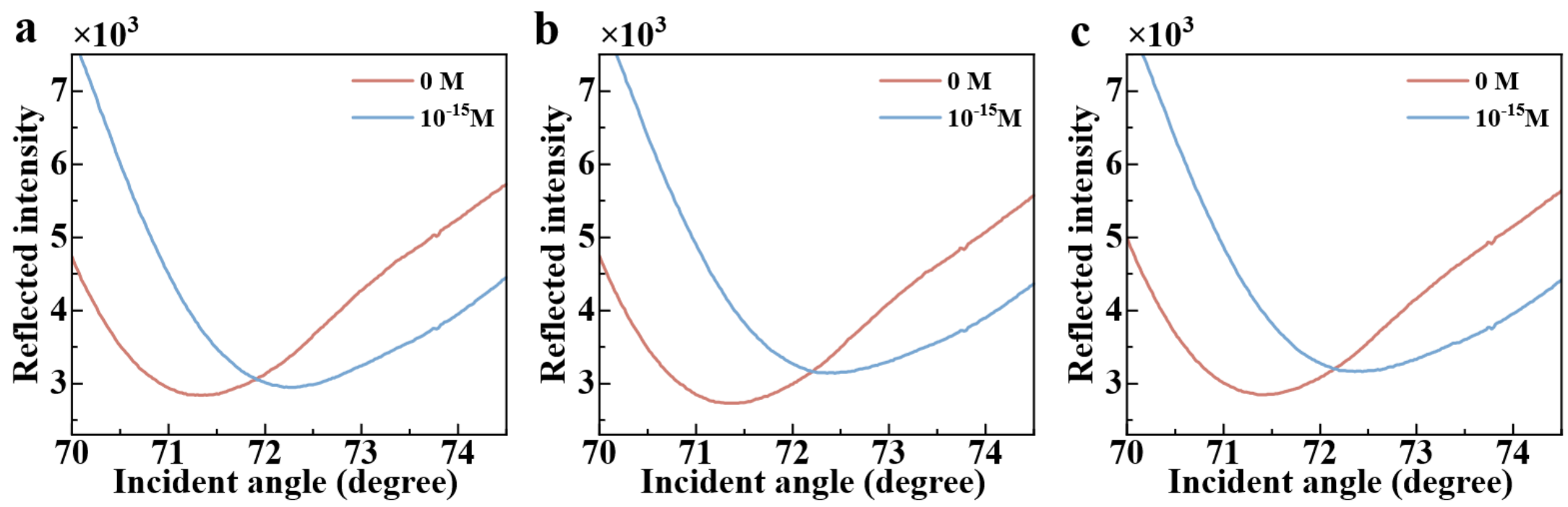

"Fig. S20 Pb^{2+} sensing results obtained from Au+BP-COOH chips at storage time point Day 4. (a–c) Angle-scanned reflectance curves of three independently prepared Au+BP-COOH chips measured for 0 M and 10^{-15} M Pb^{2+}. Day 4 corresponds to chips measured after 3 days of storage."

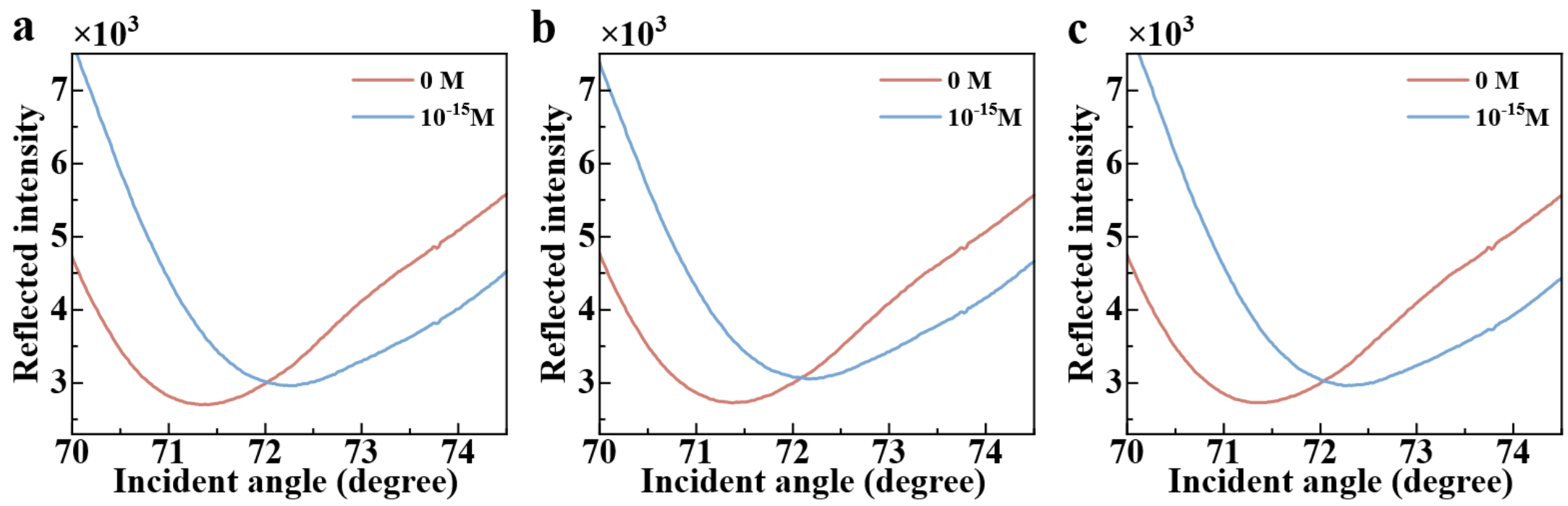

"Fig. S21 Pb^{2+} sensing results obtained from Au+BP-COOH chips at storage time point Day 5. (a–c) Angle-scanned reflectance curves of three independently prepared Au+BP-COOH chips measured for 0 M and 10^{-15} M Pb^{2+}. Day 5 corresponds to chips measured after 4 days of storage."

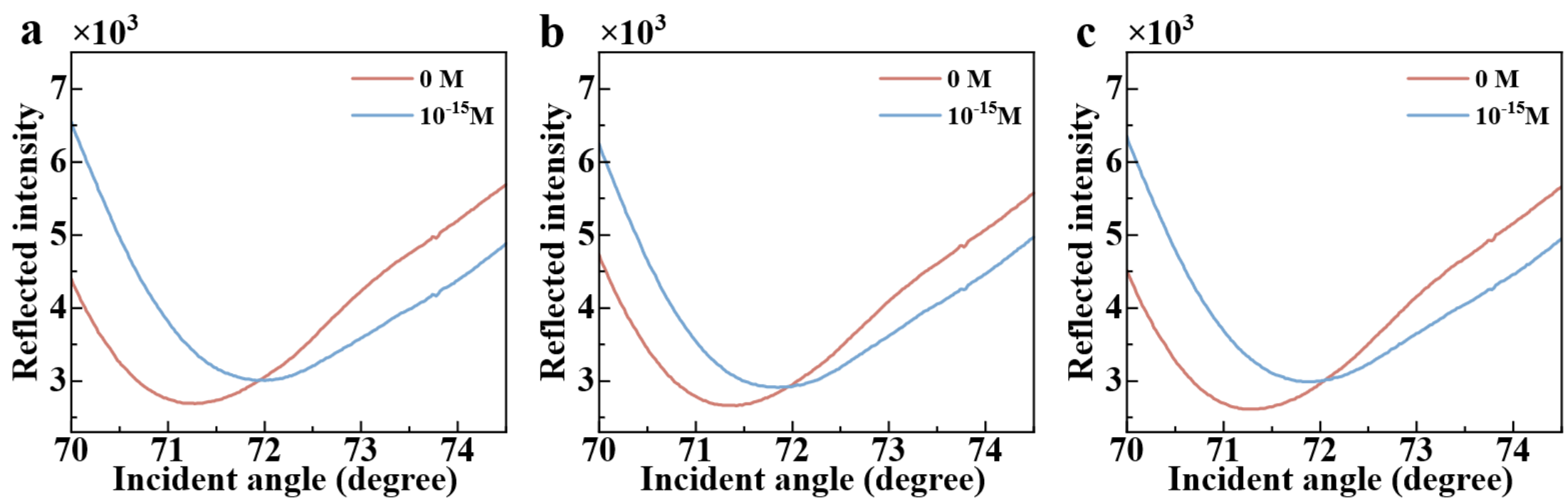

"Fig. S22 Pb^{2+} sensing results obtained from Au+BP-COOH chips at storage time point Day 6. (a–c) Angle-scanned reflectance curves of three independently prepared Au+BP-COOH chips measured for 0 M and 10^{-15} M Pb^{2+}. Day 6 corresponds to chips measured after 5 days of storage."

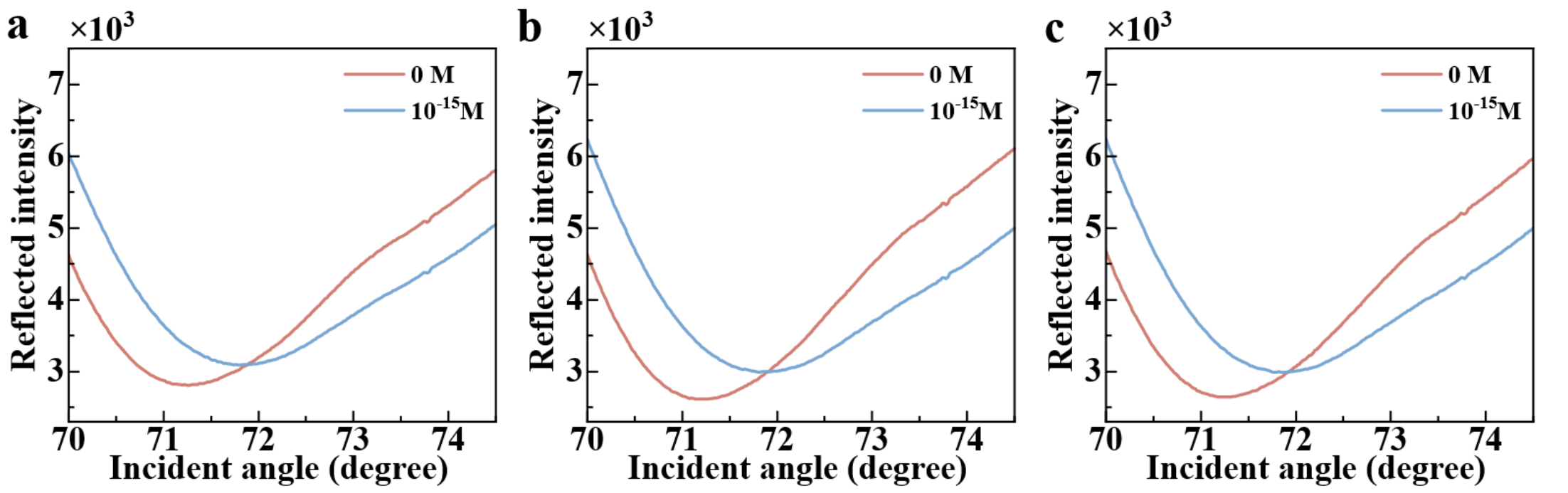

"Fig. S23 Pb^{2+} sensing results obtained from Au+BP-COOH chips at storage time point Day 7. (a–c) Angle-scanned reflectance curves of three independently prepared Au+BP-COOH chips measured for 0 M and 10^{-15} M Pb^{2+}. Day 7 corresponds to chips measured after 6 days of storage."

Supporting Information, lines 108–112:

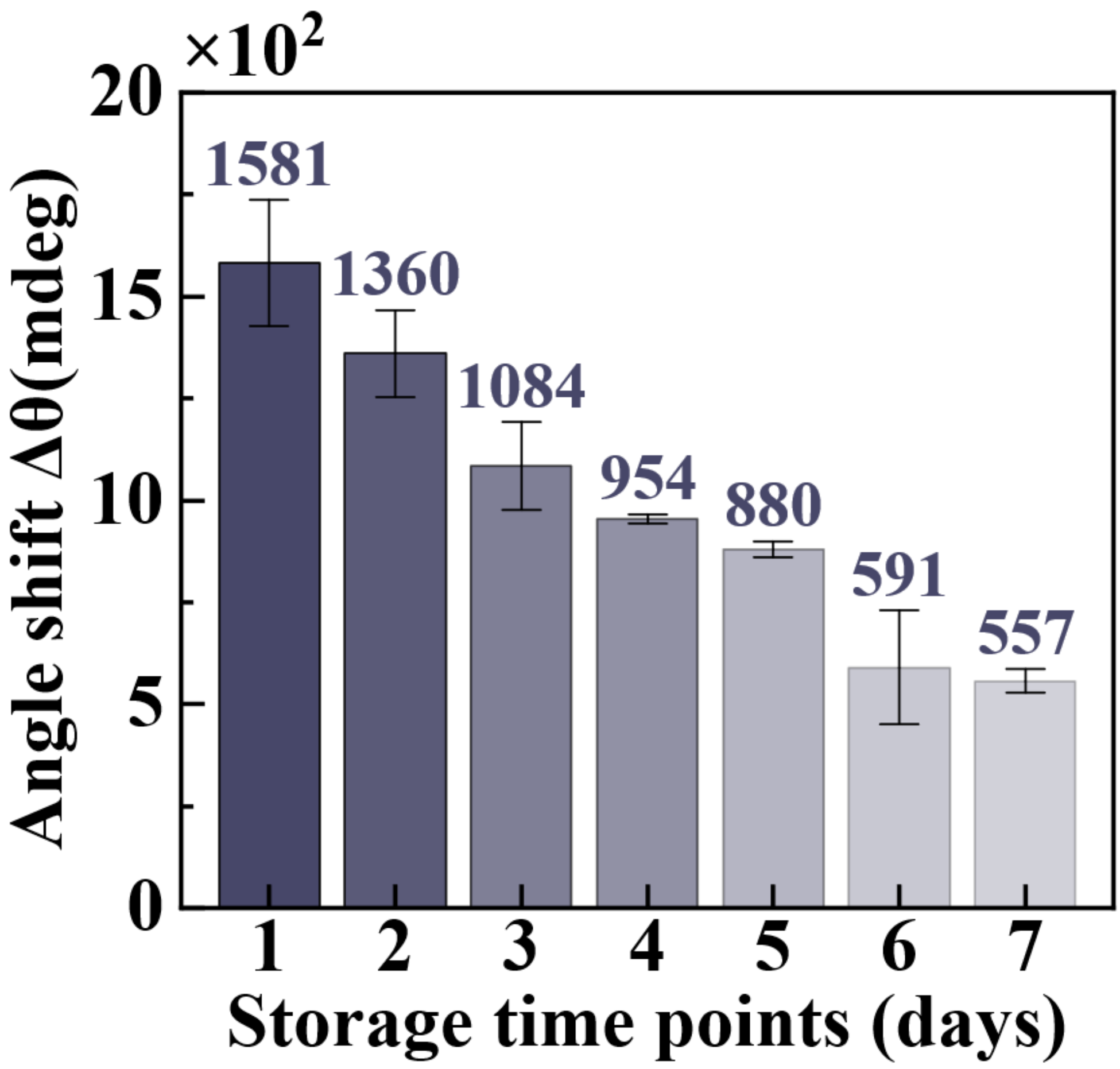

"Fig. S24 Storage-stability statistics of Au+BP-COOH chips measured with 10^{-15} M Pb^{2+} at different storage time points. Resonance-angle shift Δθ of Au+BP-COOH chips measured with 10^{-15} M Pb^{2+} from Day 1 to Day 7. Day 1 corresponds to freshly prepared chips measured immediately after fabrication, and Days 2–7 correspond to chips measured after 1, 2, 3, 4, 5 and 6 days of storage, respectively. Error bars are from independently prepared Au+BP-COOH chips."

Comment 4

If this binding is not an enzymatic reaction but rather a chemical coordination, can it be reversible? Recovery and regeneration characteristics of the sensor surface should be examined and discussed.

Response:

We thank the reviewer for this helpful suggestion. We agree that, because the interaction between Pb^{2+} and BP-COOH is proposed to be mainly based on chemical coordination with oxygen-containing interfacial sites rather than an enzymatic reaction, the recovery and regeneration behavior of the sensing surface should be experimentally evaluated.

In the revised manuscript, we added a regeneration experiment using 10^{-18} M Pb^{2+}. After each Pb^{2+} sensing cycle, the Au+BP-COOH chip was treated with 10 mM EDTA solution, followed by rinsing with pure water until the baseline became stable. The regenerated chip was then used for the next Pb^{2+} sensing cycle. Cycle 0 denotes the measurement before regeneration, whereas Cycles 1–5 denote measurements after each regeneration treatment. As shown in Fig. S17, the Pb^{2+} response remains distinguishable after five regeneration cycles. This result indicates that the Pb^{2+} coordination interaction on Au+BP-COOH can be partially reversed by EDTA treatment and that the sensing surface retains regeneration capability under the tested conditions.

We have added this regeneration experiment to the revised manuscript and Supporting Information, and described the experimental procedure in the Methods section.

Changes made in the manuscript:

Manuscript, lines 301–303:

"The regeneration behavior and storage stability of Au+BP-COOH were also evaluated. The regeneration experiment was performed using 10^{-18} M Pb^{2+} on the Au+BP-COOH chip shown in Fig. S15. As shown in Fig. S17, the Pb^{2+} response remains distinguishable after five regeneration cycles."

Manuscript, lines 444–448:

"For regeneration measurements, the Au+BP-COOH chip shown in Fig. S15 was used, and 10^{-18} M Pb^{2+} was selected for the regeneration test. After each Pb^{2+} sensing cycle, the Au+BP-COOH chip was treated with 10 mM EDTA solution, followed by rinsing with pure water until the baseline became stable. The regenerated chip was then used for the next Pb^{2+} sensing cycle. Cycle 0 denotes the measurement before regeneration, whereas Cycles 1–5 denote measurements after each regeneration treatment."

Supporting Information, lines 86–89:

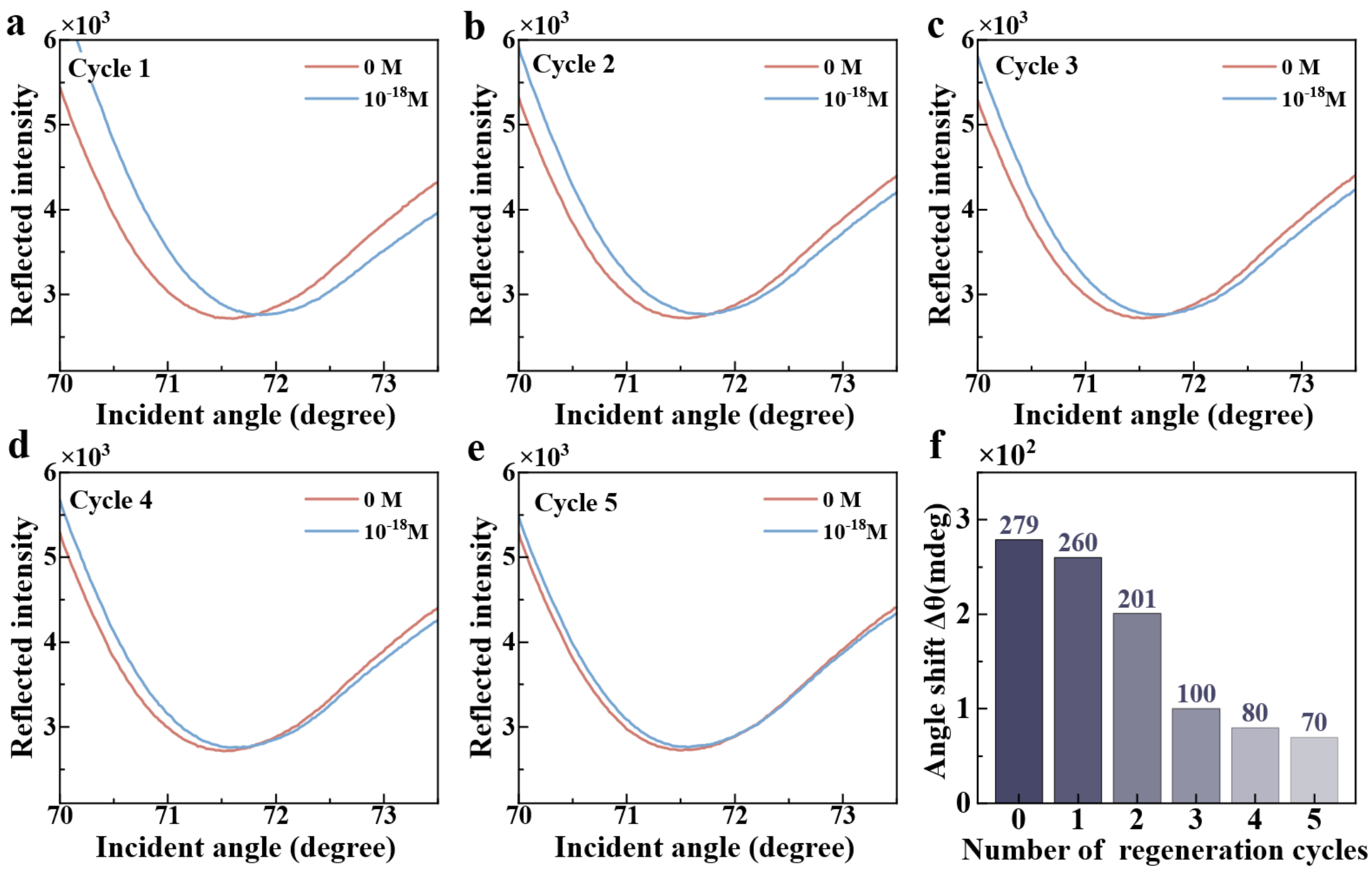

“Fig. S17 Regeneration results obtained from the Au+BP-COOH chip shown in Fig. S15 using 10^{-18} M Pb^{2+}. (a–e) Angle-scanned reflectance curves recorded for 0 M and 10^{-18} M Pb^{2+} in Cycles 1, 2, 3, 4 and 5, respectively. **(f)** Resonance-angle shift Δθ as a function of the number of regeneration cycles. The value at 0 corresponds to the initial 10^{-18} M Pb^{2+} sensing response before regeneration, and the values at 1–5 correspond to the responses after 1–5 regeneration cycles, respectively.”

Comment 5

The two configurations are labeled "SPR" and "SPR+BP-COOH" throughout the manuscript, but since SPR refers to the phenomenon itself rather than a specific sample, this is a bit confusing. I would suggest renaming them as "Au" and "Au+BP-COOH" for clarity.

Response:

We thank the reviewer for this helpful suggestion on terminology. We agree that “SPR” refers to the optical phenomenon and the sensing technique rather than a specific sample configuration. To avoid ambiguity, we have revised the sample naming throughout the manuscript and Supporting Information. The unmodified Au-film sensing chip is now referred to as **bare Au**, and the BP-COOH-modified Au-film sensing chip is referred to as **Au+BP-COOH**. We also changed the related figure labels, figure captions, Results, Discussion and Methods accordingly. For example, the revised Fig. 5 now uses **Au+BP-COOH** and **bare Au** instead of the previous “SPR+BP-COOH” and “SPR” labels.

Changes made in the manuscript:

Manuscript, lines 20–28:

“Lead ion (Pb^{2+}) contamination poses a severe and persistent threat to environmental safety and human health, demanding ultrasensitive and selective detection strategies. Herein, we fabricate a carboxyl-functionalized black phosphorene modified surface plasmon resonance sensor (Au+BP-COOH) with built-in electric field enhancement for attomolar-level and highly selective Pb^{2+}

detection. The Au/BP-COOH junction generates a strong interfacial built-in electric field, which markedly strengthens near-field electromagnetic coupling and amplifies the SPR optical response, thereby significantly boosting detection sensitivity. Meanwhile, abundant oxygen-containing sites on BP-COOH enable efficient and preferential interaction with Pb^{2+}. Impressively, this sensor achieves an ultralow detection limit of 10^{-19} M, 14 orders of magnitude lower than that of bare Au, and exhibits outstanding selectivity against Cd^{2+}, Cu^{2+}, Zn^{2+}, Ni^{2+}, Ca^{2+}, and Mg^{2+}."

Manuscript, lines 105–112:

"Here, we construct a built-in electric field-enhanced Au+BP-COOH by directly stacking a BP-COOH functional layer onto a prism-coupled Au-film SPR platform. In this design, the BP layer is intended to enhance the angular SPR response, while the oxygen-containing chemical environment of the BP-COOH functional layer provides favorable interfacial sites for Pb^{2+} interaction. Unlike recognition strategies that depend on DNAzymes, labels, or multistep amplification reactions, this structure retains the simplicity of a bare-Au SPR platform while directly engineering the sensing interface itself. Under the same sensing framework, the limit of detection for Pb^{2+} reaches 10^{-19} M for the proposed built-in electric field-enhanced Au+BP-COOH, whereas bare Au shows a distinguishable Pb^{2+} response from 10^{-5} M. This study develops a simple yet effective modification route, which advances SPR sensing for ultratrace Pb^{2+} monitoring."

Manuscript, lines 242–252:

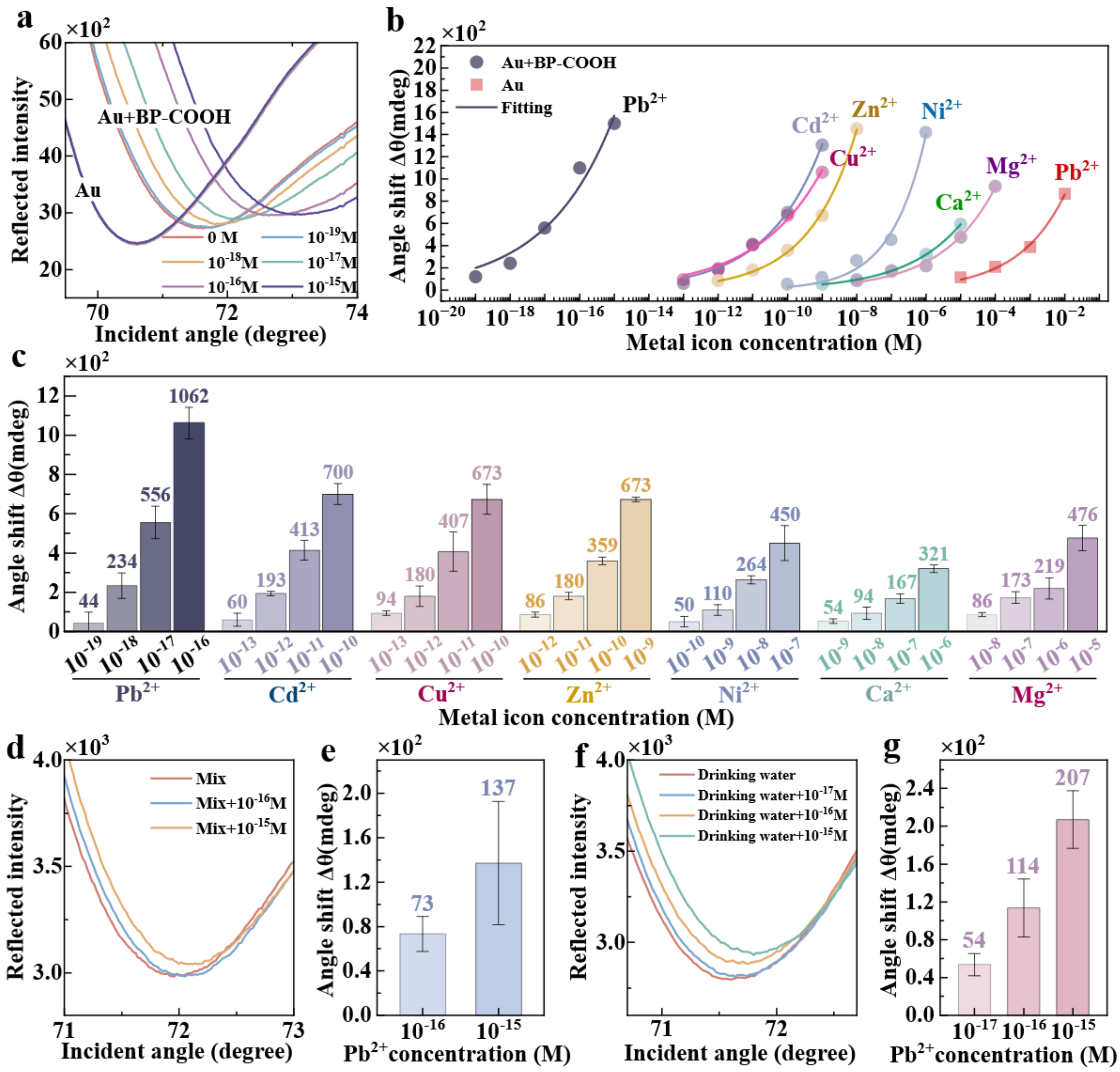

"Fig. 5. Pb^{2+} sensing response of Au+BP-COOH and comparison with other metal ions in pure water, mixed-ion solution, and drinking water. (a) Angle-scanned reflectance curves of Au+BP-COOH measured for Pb^{2+} concentrations of 0, 10^{-19}, 10^{-18}, 10^{-17}, 10^{-16} and 10^{-15} M, together with the reflectance curve of bare Au. (b) Resonance-angle shift $\Delta\theta$ as a function of metal-ion concentration for Au+BP-COOH and bare Au. The data points are mean values from the corresponding statistical results in c and Fig. S4, and the solid lines represent nonlinear fitting curves. (c) Resonance-angle shift $\Delta\theta$ values of Au+BP-COOH measured at different concentrations of Pb^{2+}, Cd^{2+}, Cu^{2+}, Zn^{2+}, Ni^{2+}, Ca^{2+} and Mg^{2+}. The Pb^{2+} data are from the ten independently prepared Au+BP-COOH chips shown in Figs. S6–S15 and statistically summarized in Fig. S16, and the data for Cd^{2+}, Cu^{2+}, Zn^{2+}, Ni^{2+}, Ca^{2+} and Mg^{2+} are from the three independently prepared Au+BP-COOH chips shown in Figs. S25–S30. (d) Angle-scanned reflectance curves of Au+BP-COOH measured in Mix, Mix + 10^{-16} M Pb^{2+} and Mix + 10^{-15} M Pb^{2+}. (e) Net resonance-angle shift $\Delta\theta$ as a function of Pb^{2+} concentration in the mixed-ion solution. The statistical data are from Fig. S31. (f) Angle-scanned reflectance curves of Au+BP-COOH measured in drinking water, drinking water + 10^{-17} M Pb^{2+}, drinking water + 10^{-16} M Pb^{2+} and drinking water + 10^{-15} M Pb^{2+}. (g) Net resonance-angle shift $\Delta\theta$ as a function of Pb^{2+} concentration in drinking water. The statistical data are from Fig. S32."

Manuscript, lines 384–396:

"**Fabrication of Au+BP-COOH**

BP-COOH was stored at −20 °C under light protection before use. For sensing experiments, 0.0001 g BP-COOH was dispersed in 1 mL pure water in the clean laboratory and sonicated in an ice-water bath for 480 s. The dispersion was freshly prepared before use.

The SPR substrate consisted of a 50 nm Au film with a 5 nm Cr adhesion layer. Before use, the Au-coated chip was rinsed once with ethanol, once with pure water and once again with ethanol. The cleaned Au chip without BP-COOH deposition was used as bare Au. For fabrication of Au+BP-COOH, 0.5 mL of the freshly prepared BP-COOH dispersion was drop-cast onto the Au film and dried naturally at 25 °C in the clean laboratory. The Au+BP control chips were prepared using the same drop-casting and drying procedure, except that the BP dispersion was used instead of the BP-COOH dispersion. The representative Au+BP-COOH structure used in the main-text sensing experiments had a thickness of approximately 13 nm. Additional independently prepared Au+BP-COOH chips were characterized by AFM, as shown in Fig. S2. During assembly of the SPR device, cedar wood oil (MACKLIN, C805296) was used as the refractive-index-matching medium between the Au chip and the BK7 prism."

Manuscript, lines 340–357:

"In summary, we constructed a surface plasmon resonance sensor modified with carboxyl-functionalized BP (Au+BP-COOH) and demonstrated its ultralow-concentration Pb^{2+} sensing capability on a prism-coupled Au-film SPR platform. The BP-COOH functional layer provides oxygen-containing interfacial sites for Pb^{2+} interaction, while also enhancing the optical response of the SPR interface. This combination is supported by the interfacial characterization, the refractive-index calibration of different Au-based interfaces, and the Pb^{2+} sensing comparison among bare Au, Au+BP and Au+BP-COOH. As a result, Au+BP-COOH enables distinguishable Pb^{2+} detection down to 10^{-19} M in pure water, whereas bare Au and Au+BP show distinguishable Pb^{2+} responses from 10^{-5} M and 10^{-7} M, respectively, under the same sensing framework.

In addition to the markedly extended detectable concentration regime, Au+BP-COOH also exhibits a substantially lower response range for Pb^{2+} than for Cd^{2+}, Cu^{2+}, Zn^{2+}, Ni^{2+}, Ca^{2+} and Mg^{2+}. The

time-dependent statistics from ten independently prepared Au+BP-COOH chips further clarify the detection behavior at ultralow Pb^{2+} concentrations, with consistent detectability from 10^{-18} to 10^{-15} M and a time-dependent statistical detection regime at 10^{-19} M. Mixed-ion interference and drinking-water measurements further show distinguishable Pb^{2+} responses in more complex matrix backgrounds. The regeneration and storage-stability tests also show that Au+BP-COOH retains distinguishable Pb^{2+} sensing responses under the tested conditions. Overall, this work shows that direct interfacial engineering with a BP-COOH functional layer can substantially improve the performance of conventional label-free Au-film SPR for Pb^{2+} detection. These results provide a simple route for extending traditional Au-film SPR toward ultratrace heavy-metal-ion sensing."

Comment 6

How would the sensing performance change if Graphene-COOH or MoS_2-COOH were used instead of BP-COOH, given similar thickness and the same functional group? This comparison would help isolate the contribution of the built-in electric field effect from the other two mechanisms, high-index confinement and interlayer coupling, which are not unique to BP.

Response:

We sincerely thank the reviewer for this insightful and constructive suggestion. We fully understand that the purpose of this comment is to isolate the BP-related interfacial contribution from the more general optical effects of two-dimensional overlayers, especially high-index confinement and interlayer coupling, which are not unique to BP. This comment helped us improve the mechanistic organization of the revised manuscript and clarify the respective roles of BP and BP-COOH in the sensing process.

To address this issue, we revised the optical-response section and added a comparative experimental refractive-index calibration using five Au-based interfaces: bare Au, Au+Graphene, Au+MoS_2, Au+BP and Au+BP-COOH (Fig. 3). Au+Graphene and Au+MoS_2 were introduced as reference two-dimensional overlayer structures because they also possess high-index confinement and interlayer-coupling-related optical characteristics. Under the same refractive-index calibration conditions, the angular sensitivities were 127 deg/RIU for bare Au, 157 deg/RIU for Au+Graphene, 168 deg/RIU for Au+MoS_2, 256 deg/RIU for Au+BP and 259 deg/RIU for Au+BP-COOH. Thus, although Au+Graphene and Au+MoS_2 increase the angular sensitivity relative to bare Au, their sensitivities remain lower than those of Au+BP and Au+BP-COOH. This comparison indicates that the BP-based overlayer provides a stronger optical response than the other two-dimensional overlayers examined here.

Although we did not introduce Graphene-COOH and MoS_2-COOH as additional Pb^{2+} sensing interfaces in this revision, we addressed the mechanistic purpose of the reviewer's suggestion through a controlled two-level comparison. Directly introducing carboxyl functionalization to different two-dimensional materials would add extra variables related to surface chemistry, functionalization degree and interfacial coverage. Therefore, we first compared the optical contribution of different two-dimensional overlayers without involving Pb^{2+} coordination chemistry. This first comparison shows that the BP-based interfaces, Au+BP and Au+BP-COOH, exhibit stronger angular SPR responses than Au+Graphene and Au+MoS_2.

The second comparison was designed to separate the BP-related optical contribution from the BP-COOH-related Pb^{2+} interaction contribution. In the refractive-index calibration, Au+BP and Au+BP-

COOH show very close angular sensitivities, 256 and 259 deg/RIU, respectively. This indicates that carboxyl functionalization preserves the BP-related optical response and does not produce a large additional refractive-index sensitivity increase by itself. We then compared Pb^{2+} sensing on bare Au, Au+BP and Au+BP-COOH. As shown in Figs. S4, S5 and Fig. 5, bare Au shows a distinguishable Pb^{2+} response from 10^{-5} M, Au+BP from 10^{-7} M, and Au+BP-COOH down to 10^{-19} M. Because Au+BP and Au+BP-COOH have similar refractive-index sensitivities but substantially different Pb^{2+} detectable concentration regimes, this comparison indicates that BP mainly contributes to the enhanced optical response, whereas the oxygen-containing interfacial sites in BP-COOH play a key role in the ultralow-concentration Pb^{2+} response.

Therefore, the revised experiments address the reviewer's mechanistic question from two complementary aspects: first, by comparing the optical response of BP-based interfaces with other two-dimensional overlayers, and second, by distinguishing the BP overlayer contribution from the BP-COOH functional-layer contribution in Pb^{2+} sensing.

Changes made in the manuscript:

Manuscript, lines 181–185:

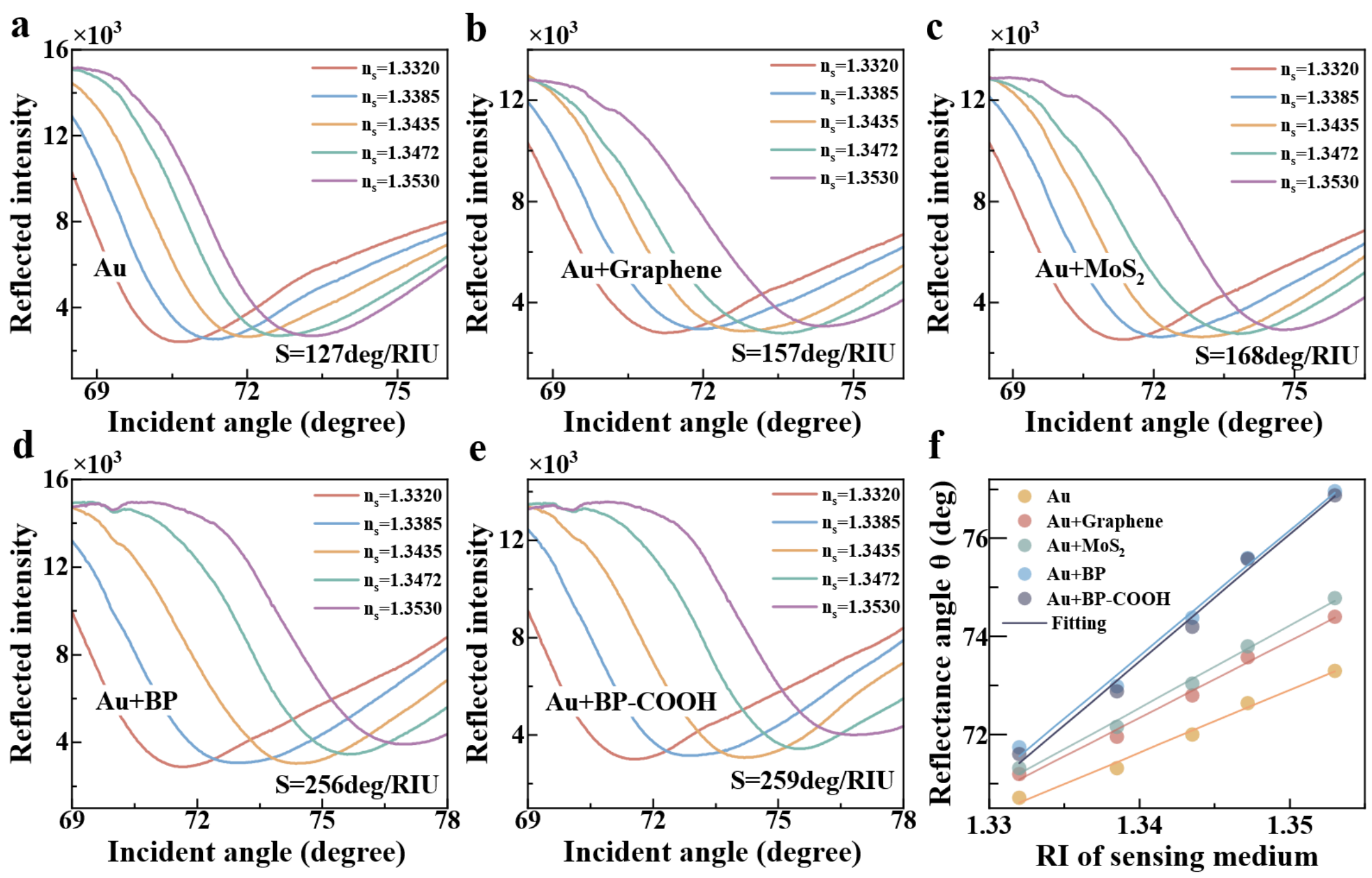

"**Fig. 3. Experimental refractive-index calibration of bare Au, Au+Graphene, Au+MoS_2, Au+BP and Au+BP-COOH. (a–e)** Angle-scanned reflectance curves of bare Au, Au+Graphene, Au+MoS_2, Au+BP and Au+BP-COOH measured in sensing media with refractive indices n_s = 1.3320, 1.3385, 1.3435, 1.3472 and 1.3530, respectively. **(f)** Resonance angle as a function of the refractive index of the sensing medium for bare Au, Au+Graphene, Au+MoS_2, Au+BP and Au+BP-COOH, together with linear fitting curves."

Manuscript, lines 193–209:

"To clarify the optical basis for the improved sensing performance of Au+BP-COOH, the refractive-index-dependent angular SPR responses of bare Au, Au+Graphene, Au+MoS_2, Au+BP and Au+BP-COOH were experimentally compared under the same sensing framework. As shown in Fig. 3a–e, the angle-scanned reflectance curves of all five structures shift toward larger incident angles when

the refractive index of the sensing medium increases from n_s = 1.3320 to 1.3530. However, the magnitude of the resonance-angle displacement is different for different interfacial structures.

The corresponding resonance angles are summarized in Fig. 3f. Linear fitting gives angular sensitivities of 127 deg/RIU for bare Au, 157 deg/RIU for Au+Graphene, 168 deg/RIU for Au+MoS_2, 256 deg/RIU for Au+BP and 259 deg/RIU for Au+BP-COOH. Compared with bare Au, the introduction of two-dimensional overlayers increases the angular response of the Au-film SPR structure to refractive-index variation. Au+Graphene and Au+MoS_2 were used as reference two-dimensional overlayer structures because they also possess high-index confinement and interlayer-coupling-related optical characteristics. However, their angular sensitivities remain lower than those of Au+BP and Au+BP-COOH under the same refractive-index calibration conditions. This comparison indicates that the BP-based overlayer provides a stronger optical response than the other two-dimensional overlayers examined here. In addition, the angular sensitivities of Au+BP and Au+BP-COOH are close to each other, indicating that carboxyl functionalization preserves the BP-related optical response while introducing oxygen-containing interfacial sites for subsequent Pb^{2+} interaction."

Manuscript, lines 210–215:

"The simulated angular SPR response and electric-field distribution of the Au-film structure with and without a BP overlayer are provided in Fig. S3. The simulation shows the same enhancement trend after introducing the BP overlayer and provides a theoretical reference for the BP-induced optical response. In the experimental Au+BP-COOH structure, the BP-COOH functional layer contains polymer-functionalized BP nanosheets, and therefore the experimental refractive-index calibration in Fig. 3 is used as the direct basis for evaluating the optical response of the actual sensing interface."

Manuscript, lines 259–264:

"To clarify the contribution of BP and BP-COOH to Pb^{2+} sensing, control measurements were performed on bare Au and Au+BP. The Pb^{2+} sensing results of bare Au and Au+BP are provided in Figs. S4 and S5, respectively. Bare Au shows a distinguishable Pb^{2+} response from 10^{-5} M, whereas Au+BP shows a distinguishable response from 10^{-7} M. This comparison indicates that the BP overlayer improves the Pb^{2+} sensing response relative to bare Au. By further introducing the BP-COOH functional layer, the detectable Pb^{2+} concentration is extended to 10^{-19} M, indicating that the oxygen-containing interfacial sites of BP-COOH play a key role in the ultralow-concentration Pb^{2+} response."

Supporting Information, lines 29–34:

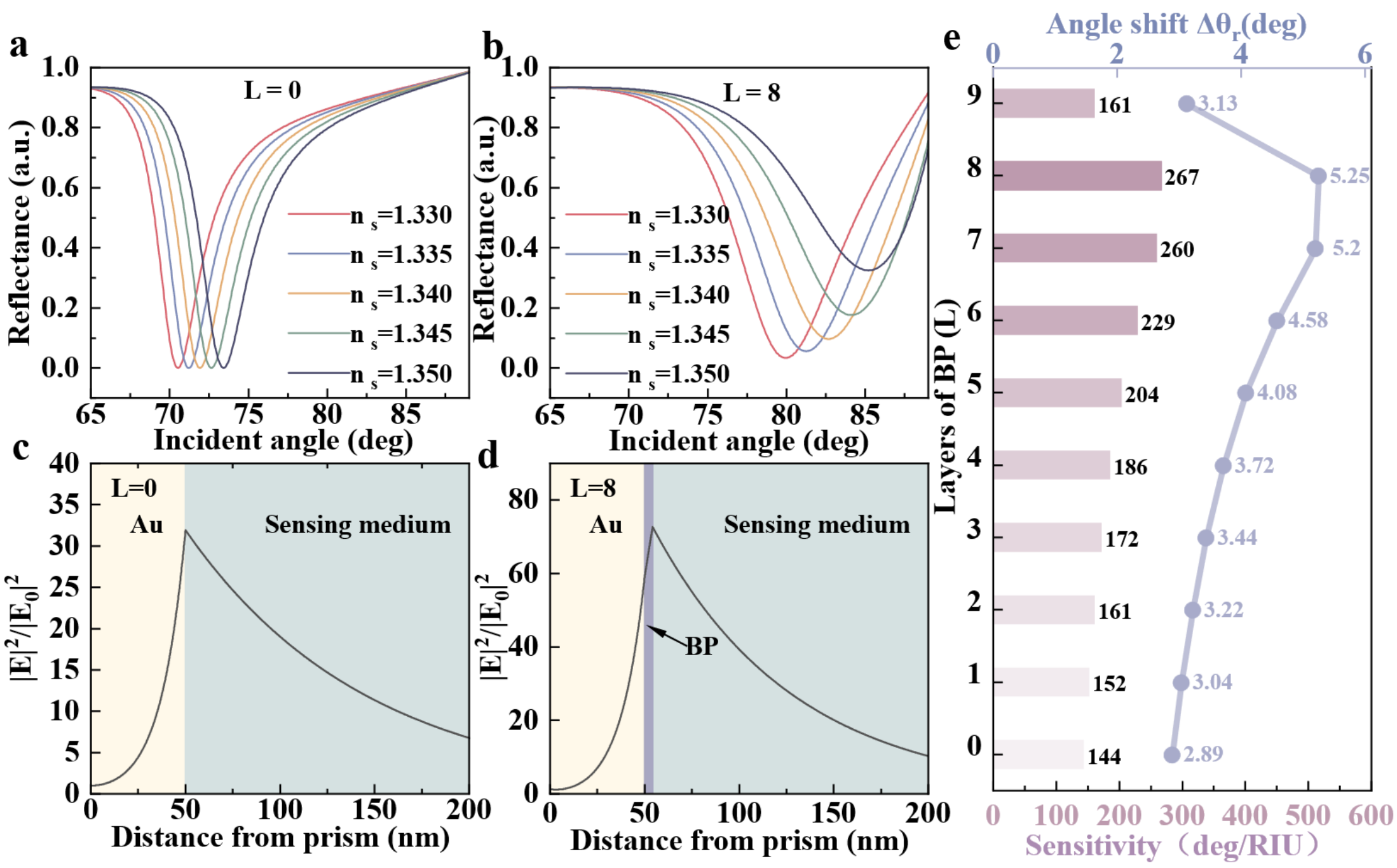

“**Fig. S3 Simulated angular SPR response, electric-field distribution, and layer-number-dependent sensitivity of the Au-film SPR structure with and without a BP overlayer. (a)** Simulated angle-scanned reflectance curves of bare Au with L = 0 at sensing-medium refractive indices n_s = 1.330, 1.335, 1.340, 1.345 and 1.350. **(b)** Simulated angle-scanned reflectance curves of the BP-modified Au structure with L = 8 at sensing-medium refractive indices n_s = 1.330, 1.335, 1.340, 1.345 and 1.350. **(c)** Normalized electric-field intensity distribution of bare Au with L = 0. **(d)** Normalized electric-field intensity distribution of the BP-modified Au structure with L = 8. **(e)** Simulated angular sensitivity and total resonance-angle shift as a function of the number of BP layers.”

Supporting Information, lines 36–40:

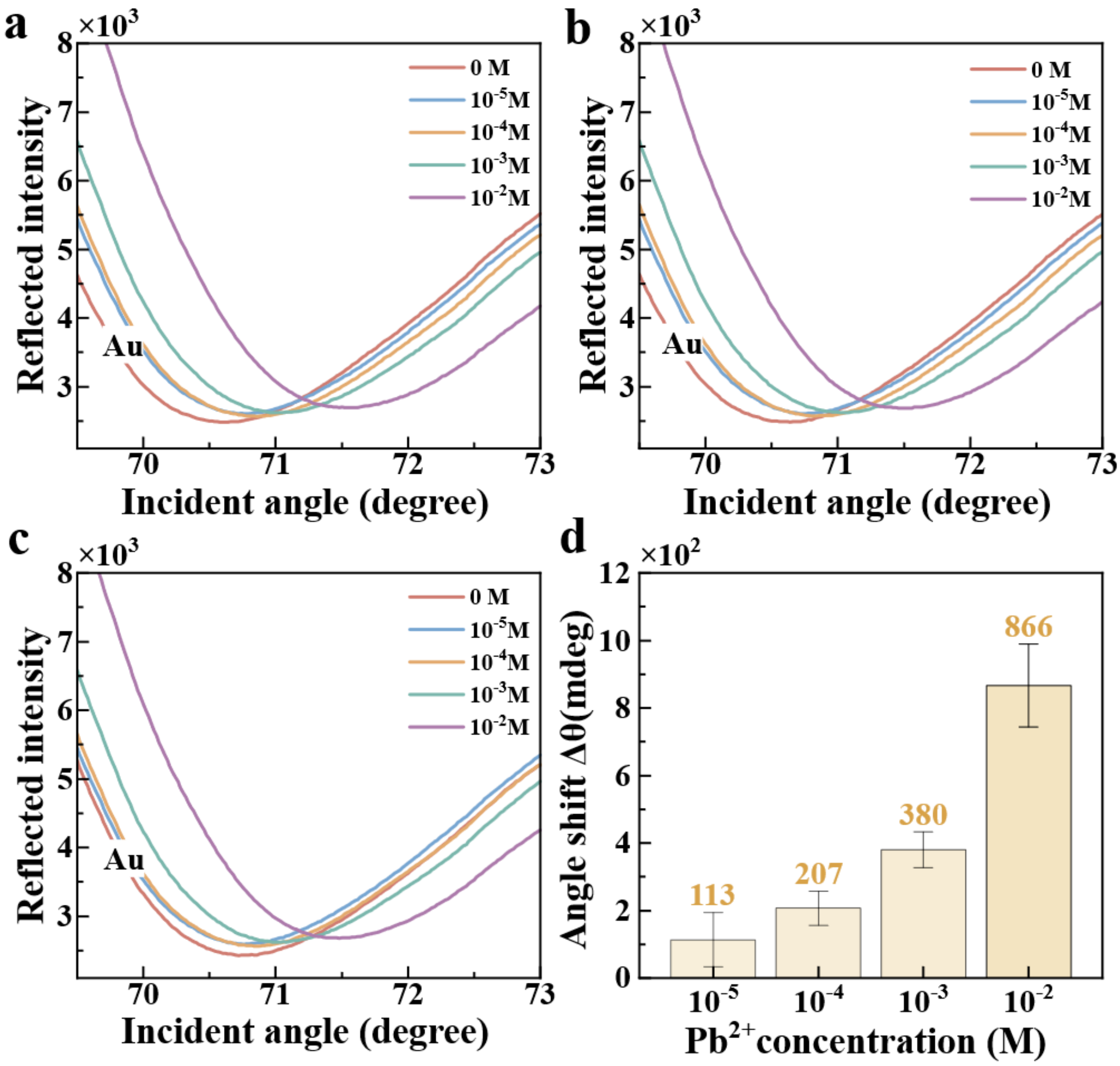

"**Fig. S4 Pb^{2+} sensing results obtained from bare Au. (a–c)** Angle-scanned reflectance curves of three independently prepared bare Au chips measured for Pb^{2+} concentrations of 0, 10^{-5}, 10^{-4}, 10^{-3} and 10^{-2} M. **(d)** Resonance-angle shift Δθ as a function of Pb^{2+} concentration for bare Au."

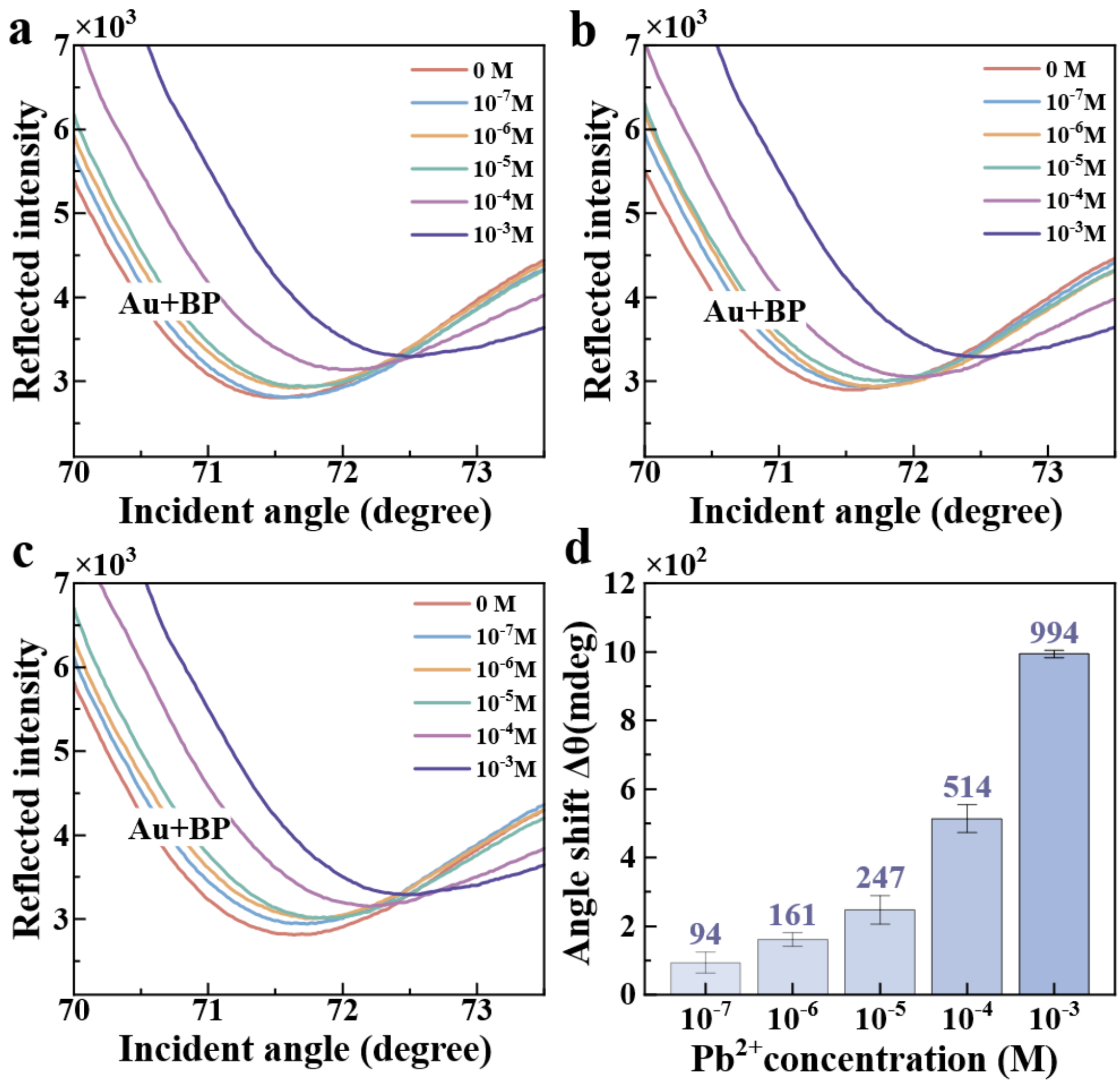

"Fig. S5 Pb^{2+} sensing results obtained from Au+BP. (a–c) Angle-scanned reflectance curves of three independently prepared Au+BP chips measured for Pb^{2+} concentrations of 0, 10^{-7}, 10^{-6}, 10^{-5}, 10^{-4} and 10^{-3} M. **(d)** Resonance-angle shift Δθ as a function of Pb^{2+} concentration for Au+BP."

Comment 7

The origin of Pb^{2+} selectivity is unclear. Interaction with –COOH can happen with other divalent ions as well. A more detailed explanation of why Pb^{2+} binds preferentially, in terms of ionic radius, HSAB theory, binding constants, or any other solid chemical reasoning, should be presented.

Response:

We sincerely thank the reviewer for this important comment. We fully understand the reviewer's concern that the presence of –COOH groups alone cannot be used to claim exclusive Pb^{2+} recognition, because other divalent ions can also interact with carboxylate and other oxygen-containing groups. Therefore, in the revised manuscript, we avoided implying an exclusive interaction and instead clarified the result as a preferential response toward Pb^{2+} under the present sensing conditions.

To support this point experimentally, we added single-ion selectivity measurements for Cd^{2+}, Cu^{2+}, Zn^{2+}, Ni^{2+}, Ca^{2+} and Mg^{2+} using independently prepared Au+BP-COOH chips. As shown in Fig. 5b,c

and Figs. S25–S30, the detectable concentration regime for Pb^{2+} is substantially lower than those of the other tested divalent ions under identical measurement conditions. The LODs for Cd^{2+}, Cu^{2+}, Zn^{2+}, Ni^{2+}, Ca^{2+} and Mg^{2+} are 10^{-13} M, 10^{-13} M, 10^{-12} M, 10^{-10} M, 10^{-9} M and 10^{-8} M, respectively, whereas Au+BP-COOH shows Pb^{2+} response down to 10^{-19} M in pure water. We also added a mixed-ion interference experiment. In this experiment, the mixed-ion solution containing Cd^{2+}, Cu^{2+}, Zn^{2+}, Ni^{2+}, Ca^{2+} and Mg^{2+} was first used as the matrix baseline, and the net Pb^{2+} response was obtained after subtracting this Mix baseline. As shown in Fig. 5d,e and Fig. S31, Pb^{2+} remains distinguishable in the mixed-ion solution at 10^{-16} M. These experiments show that the enhanced response to Pb^{2+} is not only observed in single-ion measurements but is also retained in the presence of competing divalent ions.

We also added a more detailed chemical explanation in the revised manuscript. The preferential response toward Pb^{2+} is attributed to stronger interfacial interaction between Pb^{2+} and oxygen-containing sites in BP-COOH. Pb^{2+} is a highly polarizable borderline-soft divalent ion and can form stable inner-sphere coordination with carboxylate and other oxygen-containing sites. In contrast, Ca^{2+} and Mg^{2+} are hard divalent ions with strong hydration, so their interaction with surface carboxylate sites is more strongly affected by hydration-shell retention. Transition-metal ions such as Ni^{2+}, Zn^{2+}, Cu^{2+} and Cd^{2+} can also interact with oxygen-containing groups, but their responses occur in higher concentration ranges under the same sensing conditions. This interpretation is consistent with previous reports on preferential Pb^{2+} adsorption at activated oxygen-containing sites on two-dimensional surfaces and with HSAB-based selectivity discussions in two-dimensional-material ion sensing.

Changes made in the manuscript:

Manuscript, lines 242–252:

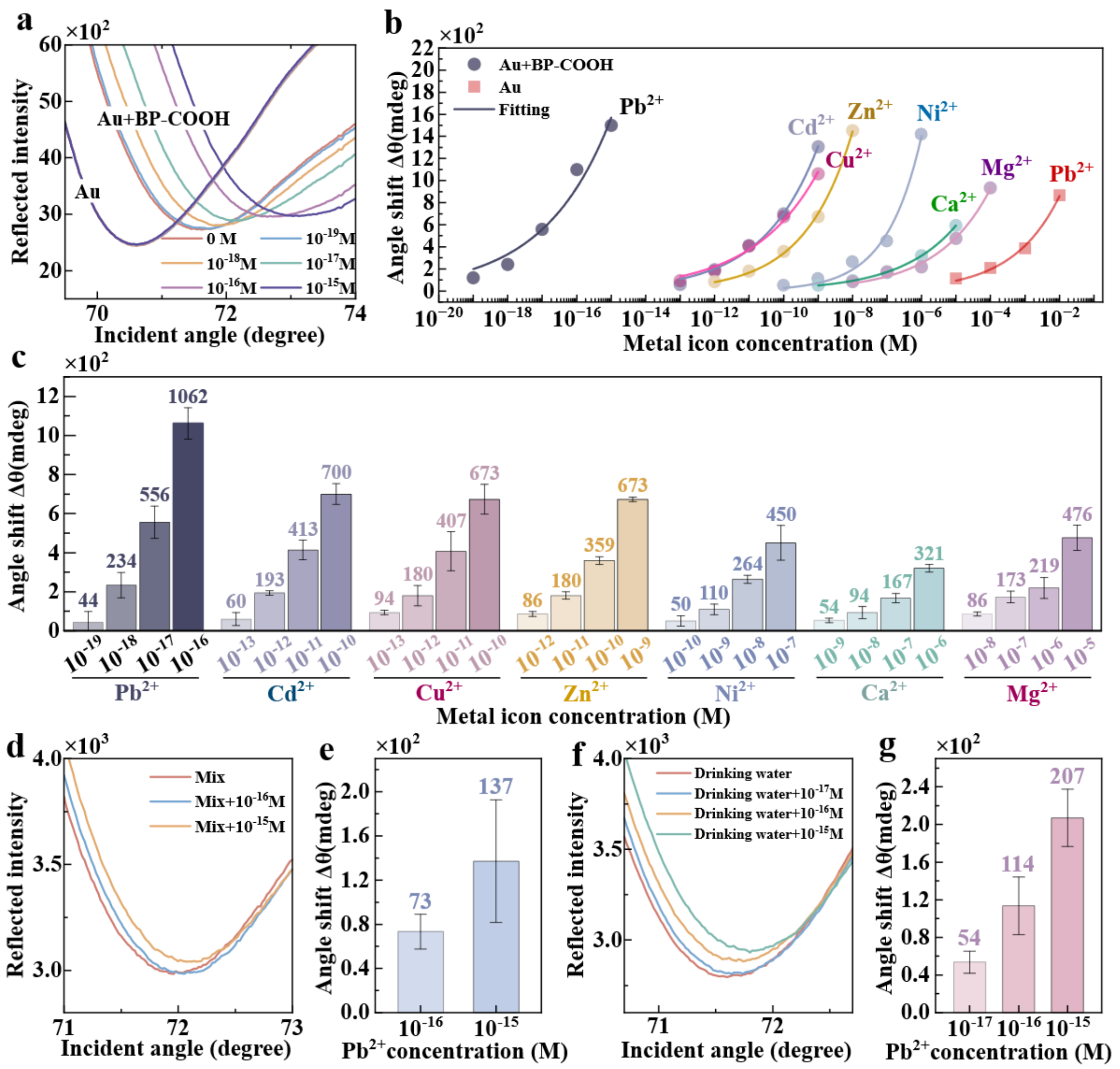

“Fig. 5. Pb^{2+} sensing response of Au+BP-COOH and comparison with other metal ions in pure water, mixed-ion solution, and drinking water. **(a)** Angle-scanned reflectance curves of Au+BP-COOH measured for Pb^{2+} concentrations of 0, 10^{-19}, 10^{-18}, 10^{-17}, 10^{-16} and 10^{-15} M, together with the reflectance curve of bare Au. **(b)** Resonance-angle shift Δθ as a function of metal-ion concentration for Au+BP-COOH and bare Au. The data points are mean values from the corresponding statistical results in c and Fig. S4, and the solid lines represent nonlinear fitting curves. **(c)** Resonance-angle shift Δθ values of Au+BP-COOH measured at different concentrations of Pb^{2+}, Cd^{2+}, Cu^{2+}, Zn^{2+}, Ni^{2+}, Ca^{2+} and Mg^{2+}. The Pb^{2+} data are from the ten independently prepared Au+BP-COOH chips shown in Figs. S6–S15 and statistically summarized in Fig. S16, and the data for Cd^{2+}, Cu^{2+}, Zn^{2+}, Ni^{2+}, Ca^{2+} and Mg^{2+} are from the three independently prepared Au+BP-COOH chips shown in Figs. S25–S30. **(d)** Angle-scanned reflectance curves of Au+BP-COOH measured in Mix, Mix + 10^{-16} M Pb^{2+} and Mix + 10^{-15} M Pb^{2+}. **(e)** Net resonance-angle shift Δθ as a function of Pb^{2+} concentration in the mixed-ion solution. The statistical data are from Fig. S31. **(f)** Angle-scanned reflectance curves of Au+BP-COOH measured in drinking water, drinking water + 10^{-17} M Pb^{2+}, drinking water + 10^{-16} M Pb^{2+} and drinking water + 10^{-15} M Pb^{2+}. **(g)** Net resonance-angle shift Δθ as a function of Pb^{2+} concentration in drinking water. The statistical data are from Fig. S32.”

Manuscript, lines 278–293:

“The selectivity of Au+BP-COOH was further evaluated by comparing its responses to other

divalent metal ions. As shown in Fig. 5b,c, the response to Pb^{2+} appears in a substantially lower concentration regime than those of Cd^{2+}, Cu^{2+}, Zn^{2+}, Ni^{2+}, Ca^{2+} and Mg^{2+} under identical measurement conditions. The corresponding single-ion sensing results for Cd^{2+}, Cu^{2+}, Zn^{2+}, Ni^{2+}, Ca^{2+} and Mg^{2+} are provided in Figs. S25–S30. The LODs for the other metal ions are 10^{-13} M for Cd^{2+}, 10^{-13} M for Cu^{2+}, 10^{-12} M for Zn^{2+}, 10^{-10} M for Ni^{2+}, 10^{-9} M for Ca^{2+} and 10^{-8} M for Mg^{2+}. These results show that Au+BP-COOH exhibits a stronger response to Pb^{2+} than to the other tested divalent ions within the present sensing system.

The preferential response toward Pb^{2+} can be attributed to the stronger interfacial interaction between Pb^{2+} and oxygen-containing sites in BP-COOH. Pb^{2+} is a highly polarizable borderline-soft divalent ion and can form stable inner-sphere coordination with carboxylate and other oxygen-containing sites. In contrast, Ca^{2+} and Mg^{2+} are hard divalent ions with strong hydration, and their interaction with surface carboxylate sites is more strongly affected by hydration-shell retention. Transition-metal ions such as Ni^{2+}, Zn^{2+}, Cu^{2+} and Cd^{2+} can also interact with oxygen-containing groups, but their response occurs in higher concentration ranges under the same sensing conditions. This interpretation is consistent with previous reports on preferential Pb^{2+} adsorption at activated oxygen-containing sites on two-dimensional surfaces [31] and with the HSAB-based selectivity discussion used in two-dimensional-material ion sensing [22].”

[31] Peng, Q. et al. Unique lead adsorption behavior of activated hydroxyl group in two-dimensional titanium carbide. Journal of the American Chemical Society 136, 4113–4116 (2014).

[22] Liu, L. et al. Grain-boundary-rich polycrystalline monolayer WS_2 film for attomolar-level Hg^{2+} sensors. Nature Communications 12, 3870 (2021).

Manuscript, lines 294–300:

“Mixed-ion interference and real-sample measurements were further performed to evaluate the sensing response under more complex matrix conditions. In the mixed-ion experiment, Mix was used as the matrix background, and the net Pb^{2+} response was obtained by subtracting the Mix baseline from the Mix + Pb^{2+} response. As shown in Fig. 5d,e and Fig. S31, Pb^{2+} remains distinguishable in the mixed-ion solution at 10^{-16} M. In the real-sample experiment, drinking water was used as the matrix background, and the net Pb^{2+} response was obtained by subtracting the drinking-water baseline from the drinking water + Pb^{2+} response. As shown in Fig. 5f,g and Fig. S32, Pb^{2+} remains distinguishable in drinking water at 10^{-17} M. The water-quality background of the drinking-water sample is provided in Table S1.”

Supporting Information, lines 114–136:

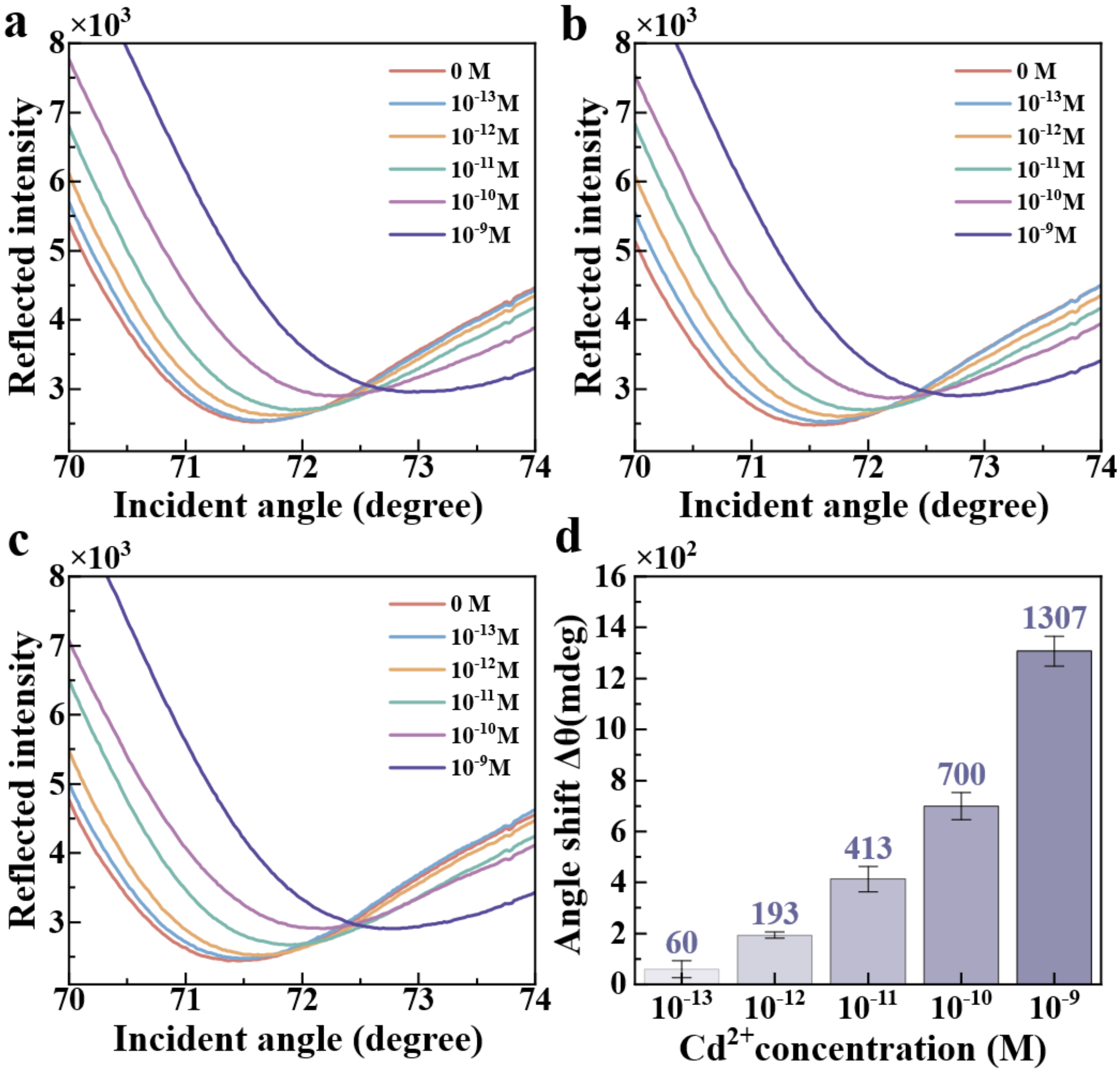

“**Fig. S25 Cd^{2+} sensing results obtained from Au+BP-COOH. (a–c)** Angle-scanned reflectance curves of three independently prepared Au+BP-COOH chips measured for Cd^{2+} concentrations of 0, 10^{-13}, 10^{-12}, 10^{-11}, 10^{-10} and 10^{-9} M. **(d)** Resonance-angle shift Δθ as a function of Cd^{2+} concentration for Au+BP-COOH.”

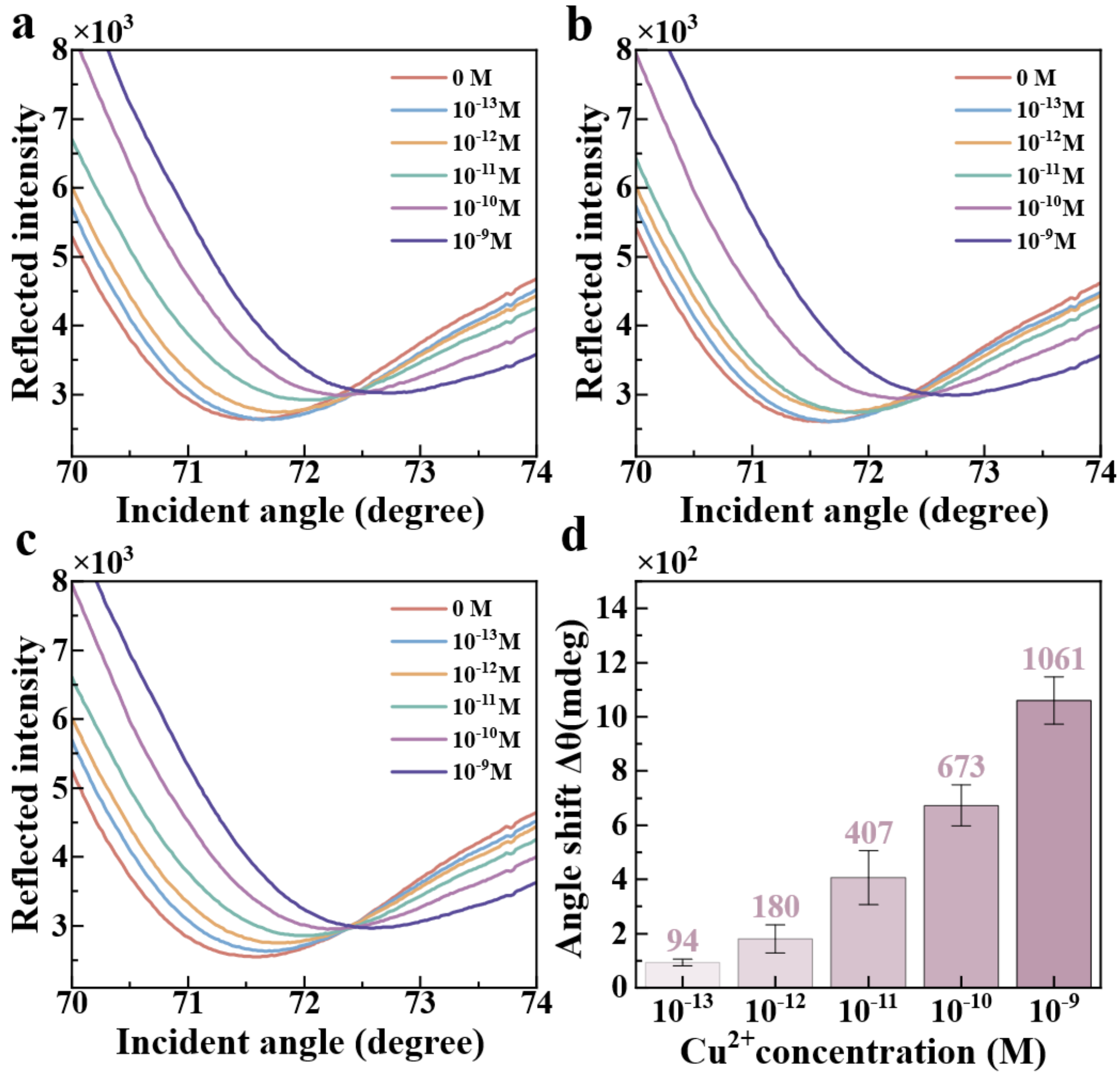

“**Fig. S26 Cu^{2+} sensing results obtained from Au+BP-COOH. (a–c)** Angle-scanned reflectance curves of three independently prepared Au+BP-COOH chips measured for Cu^{2+} concentrations of 0, 10^{-13}, 10^{-12}, 10^{-11}, 10^{-10} and 10^{-9} M. **(d)** Resonance-angle shift $\Delta\theta$ as a function of Cu^{2+} concentration for Au+BP-COOH.”

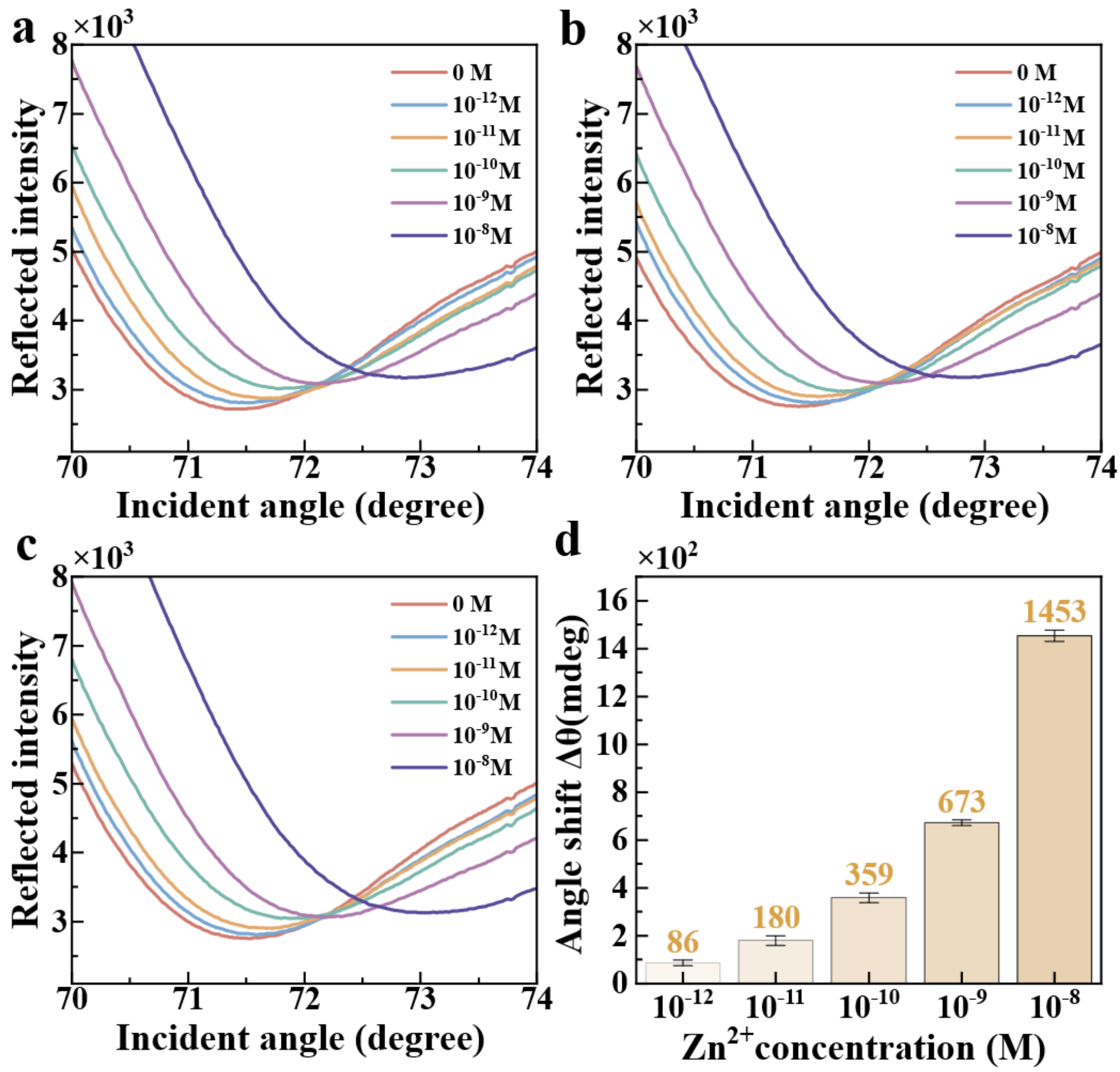

“**Fig. S27 Zn^{2+} sensing results obtained from Au+BP-COOH. (a–c)** Angle-scanned reflectance curves of three independently prepared Au+BP-COOH chips measured for Zn^{2+} concentrations of 0, 10^{-12}, 10^{-11}, 10^{-10}, 10^{-9} and 10^{-8} M. **(d)** Resonance-angle shift Δθ as a function of Zn^{2+} concentration for Au+BP-COOH.”

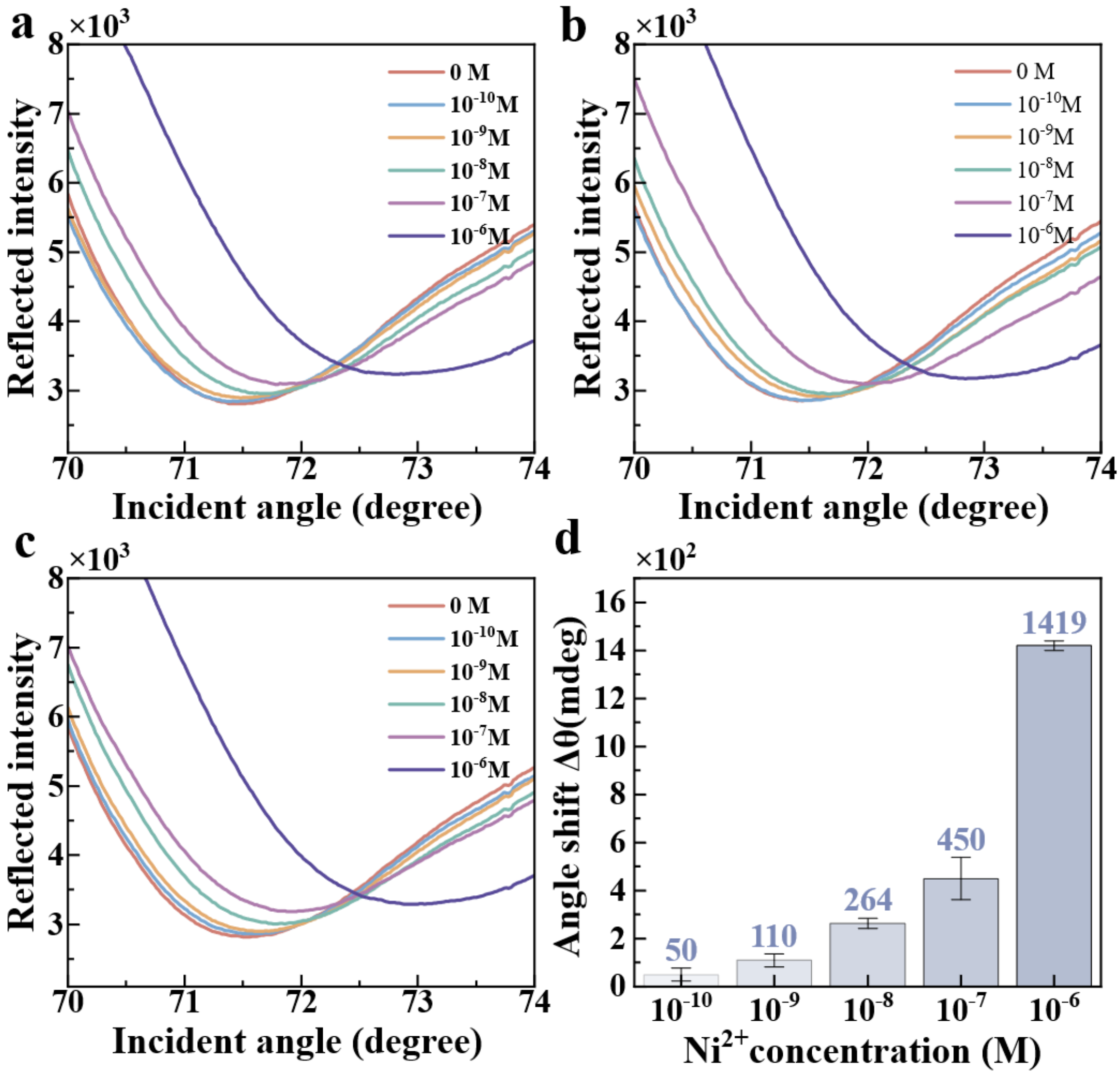

“**Fig. S28 Ni²⁺ sensing results obtained from Au+BP-COOH. (a–c)** Angle-scanned reflectance curves of three independently prepared Au+BP-COOH chips measured for Ni²⁺ concentrations of 0, 10^{-10}, 10^{-9}, 10^{-8}, 10^{-7} and 10^{-6} M. **(d)** Resonance-angle shift Δθ as a function of Ni²⁺ concentration for Au+BP-COOH.”

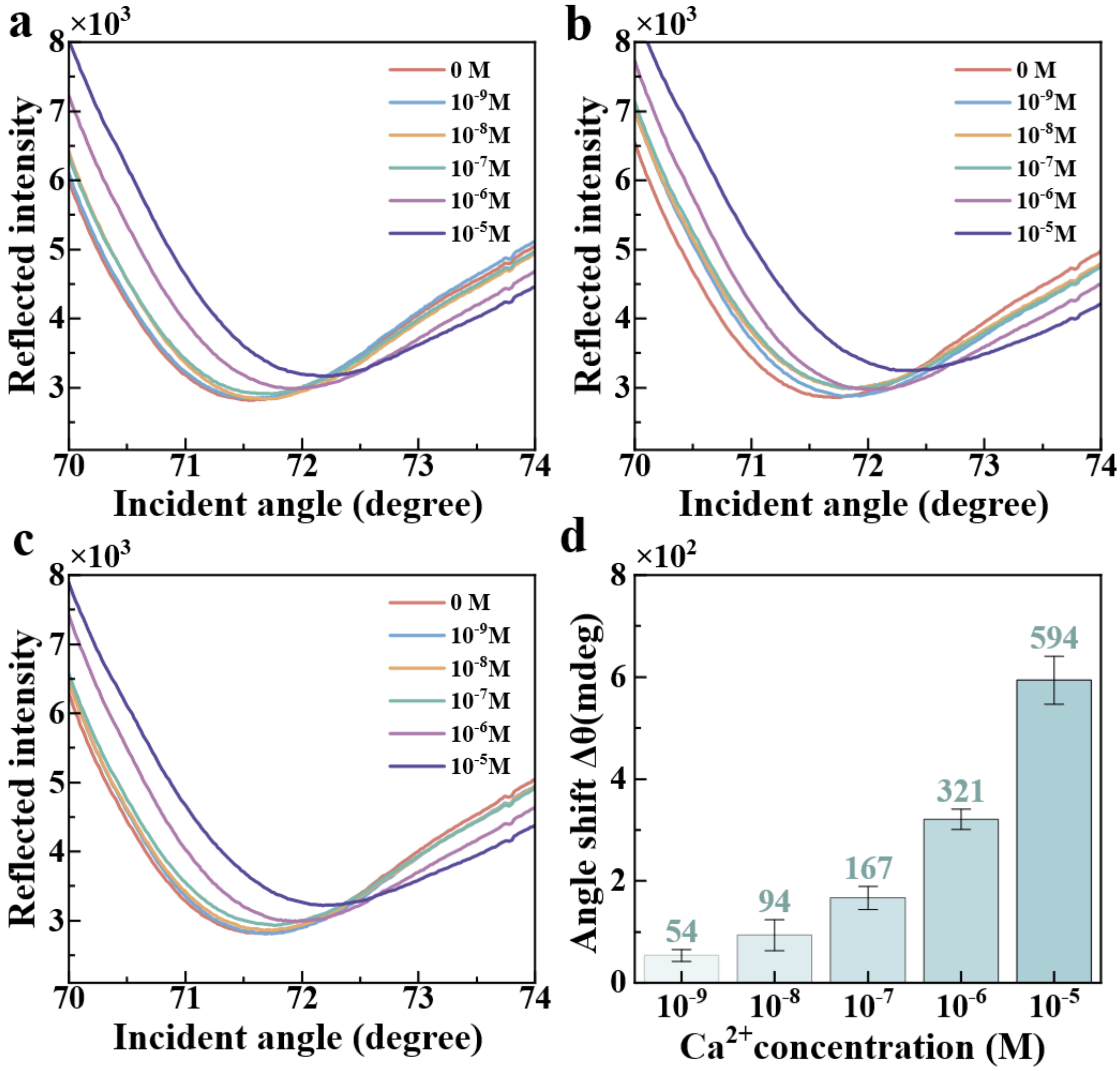

"**Fig. S29 Ca^{2+} sensing results obtained from Au+BP-COOH. (a–c)** Angle-scanned reflectance curves of three independently prepared Au+BP-COOH chips measured for Ca^{2+} concentrations of 0, 10^{-9}, 10^{-8}, 10^{-7}, 10^{-6} and 10^{-5} M. **(d)** Resonance-angle shift Δθ as a function of Ca^{2+} concentration for Au+BP-COOH."

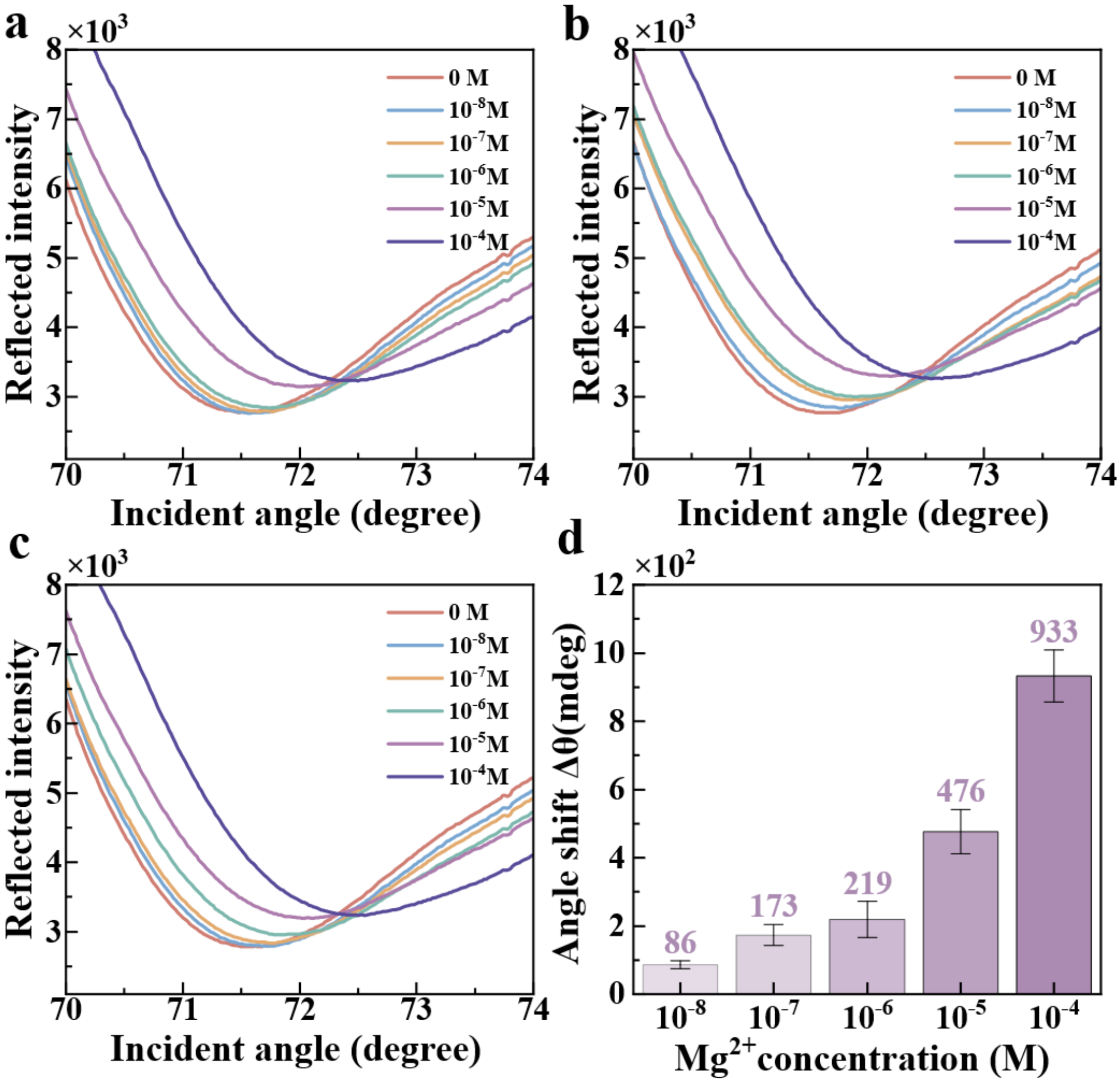

“Fig. S30 Mg^{2+} sensing results obtained from Au+BP-COOH. (a–c) Angle-scanned reflectance curves of three independently prepared Au+BP-COOH chips measured for Mg^{2+} concentrations of 0, 10^{-8}, 10^{-7}, 10^{-6}, 10^{-5} and 10^{-4} M. **(d)** Resonance-angle shift $\Delta\theta$ as a function of Mg^{2+} concentration for Au+BP-COOH.”

Supporting Information, lines 138–141:

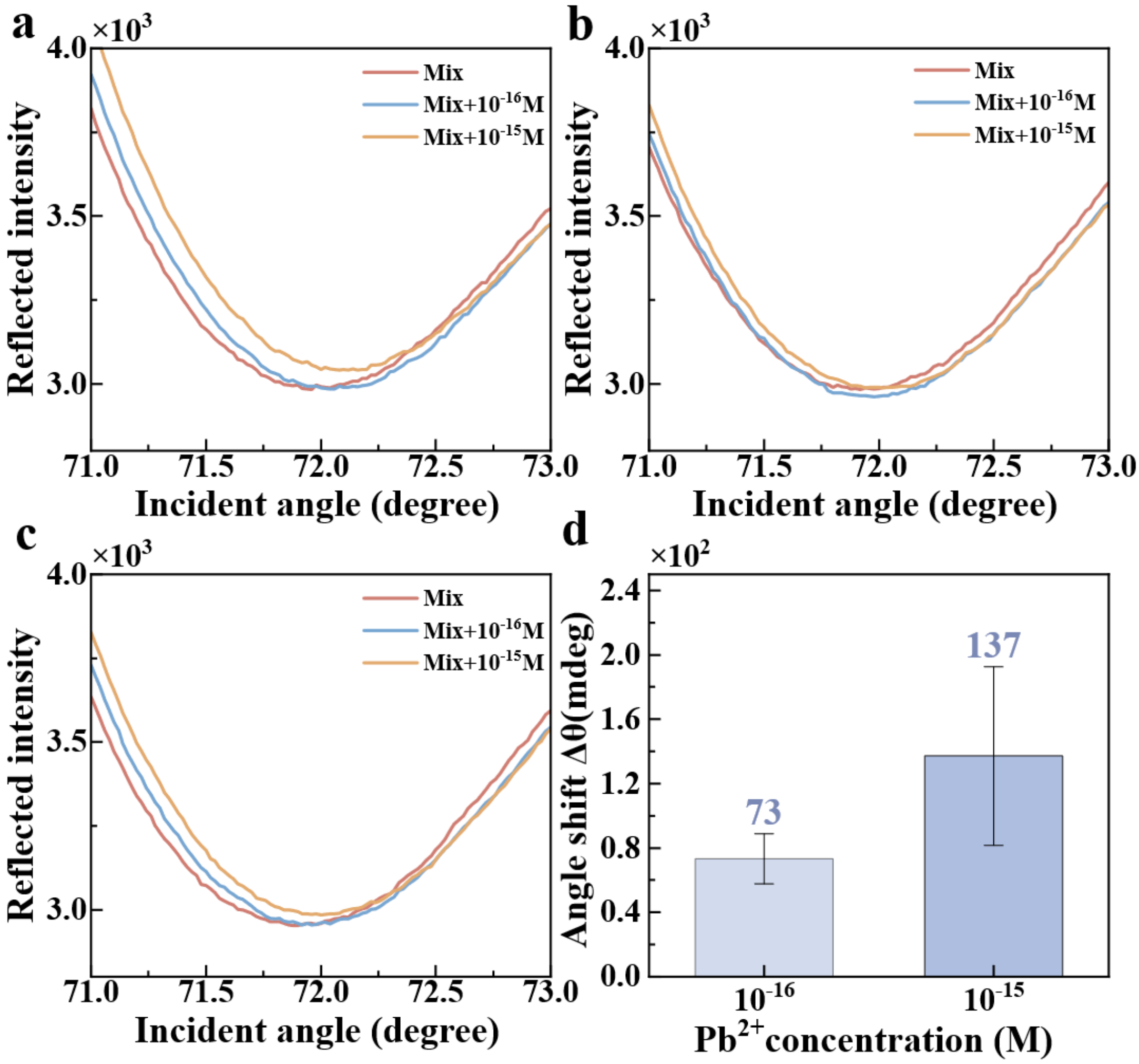

"**Fig. S31 Mixed-ion interference sensing results obtained from Au+BP-COOH. (a–c)** Angle-scanned reflectance curves of three independently prepared Au+BP-COOH chips measured in Mix, Mix + 10^{-16} M Pb^{2+} and Mix + 10^{-15} M Pb^{2+}. Mix represents the mixed-ion solution containing Cd^{2+}, Cu^{2+}, Zn^{2+}, Ni^{2+}, Ca^{2+} and Mg^{2+}. **(d)** Net resonance-angle shift Δθ as a function of Pb^{2+} concentration in the mixed-ion solution. Error bars are from three independently prepared Au+BP-COOH chips."

Reviewer 2

The authors report a carboxyl-functionalized black phosphorene-modified surface plasmon resonance sensor enabled by built-in electric field enhancement, which achieves attomolar-level and highly selective detection of Pb2+. The research topic is timely and of great significance. The integration of interfacial optical enhancement and oxygen-containing recognition sites possesses distinct scientific appeal. This manuscript is of high academic quality with evident originality, showing promising application prospects in label-free heavy metal ion detection. I thus recommend its publication after appropriate revisions.

Response to Reviewer 2

We sincerely thank Reviewer 2 for the positive evaluation of our manuscript and for recognizing the significance, originality, and potential application value of this work. We are particularly encouraged by the reviewer's comments on the integration of interfacial optical enhancement with oxygen-containing recognition sites for label-free Pb^{2+} detection.

Following the reviewer's constructive suggestions, we have carefully revised the manuscript to improve the clarity, rigor, and completeness of the study. In the revised manuscript, we further clarified the definition of the detection limit and the distinguishability threshold, added independent-chip statistics and error bars, strengthened the comparison among different Au-based interfaces, provided additional evidence for BP-COOH stability and sensor regeneration, and expanded the discussion of Pb^{2+} selectivity and matrix interference. We also added mixed-ion and drinking-water measurements to better evaluate the applicability of Au+BP-COOH under more complex sensing conditions.

We believe that these revisions have significantly improved the mechanistic clarity and experimental reliability of the manuscript. The detailed responses to the reviewer's specific comments are provided below.

Comment 1

The use of "selectivity" and "specificity" should be clarified. The manuscript shows a much stronger response toward Pb^{2+} than toward Cd^{2+}, Cu^{2+}, Zn^{2+}, Ni^{2+}, Ca^{2+}, and Mg^{2+}. The authors should use the terms "selectivity" and "specificity" carefully and consistently.

Response:

We thank the reviewer for this helpful comment. We agree that the terms "selectivity" and "specificity" should be used carefully. In the revised manuscript, we avoided using "specificity" to describe the sensing behavior, because the interaction between BP-COOH and Pb^{2+} is based on chemical coordination with oxygen-containing sites rather than an exclusive biological recognition process. Other divalent ions can also interact with carboxylate or oxygen-containing groups, so "specificity" would be too absolute.

Therefore, we consistently use **"selectivity"** to describe the experimentally observed stronger response toward Pb^{2+} than toward Cd^{2+}, Cu^{2+}, Zn^{2+}, Ni^{2+}, Ca^{2+} and Mg^{2+} under the same sensing conditions. We also use **"preferential interaction"** or **"preferential response"** when discussing the chemical origin of the stronger Pb^{2+} response, rather than implying exclusive binding.

This terminology is supported by the revised experimental results. As shown in Fig. 5b,c and Figs. S25–S30, Au+BP-COOH responds to Pb^{2+} in a much lower concentration regime than to the other tested divalent ions. The LODs for Cd^{2+}, Cu^{2+}, Zn^{2+}, Ni^{2+}, Ca^{2+} and Mg^{2+} are 10^{-13} M, 10^{-13} M, 10^{-12}

M, 10^{-10} M, 10^{-9} M and 10^{-8} M, respectively, whereas Pb^{2+} is detected down to 10^{-19} M in pure water. In addition, the mixed-ion experiment further shows that Pb^{2+} remains distinguishable in the presence of competing divalent ions at 10^{-16} M, after subtracting the mixed-ion matrix baseline (Fig. 5d,e and Fig. S31). Thus, the revised manuscript describes the sensing behavior as Pb^{2+} selectivity or preferential Pb^{2+} response, not Pb^{2+} specificity.

Changes made in the manuscript:

Manuscript, lines 20–28:

"Lead ion (Pb^{2+}) contamination poses a severe and persistent threat to environmental safety and human health, demanding ultrasensitive and selective detection strategies. Herein, we fabricate a carboxyl-functionalized black phosphorene modified surface plasmon resonance sensor (Au+BP-COOH) with built-in electric field enhancement for attomolar-level and highly selective Pb^{2+} detection. The Au/BP-COOH junction generates a strong interfacial built-in electric field, which markedly strengthens near-field electromagnetic coupling and amplifies the SPR optical response, thereby significantly boosting detection sensitivity. Meanwhile, abundant oxygen-containing sites on BP-COOH enable efficient and preferential interaction with Pb^{2+}. Impressively, this sensor achieves an ultralow detection limit of 10^{-19} M, 14 orders of magnitude lower than that of bare Au, and exhibits outstanding selectivity against Cd^{2+}, Cu^{2+}, Zn^{2+}, Ni^{2+}, Ca^{2+}, and Mg^{2+}."

Manuscript, lines 253–293:

"**Pb^{2+} sensing performance and selectivity of Au+BP-COOH.** The Pb^{2+} sensing performance of Au+BP-COOH is summarized in Fig. 5. As shown in Fig. 5a, when the Pb^{2+} concentration increases from 0 to 10^{-15} M, the angle-scanned reflectance curves of Au+BP-COOH exhibit concentration-dependent resonance-angle shifts. A distinguishable response is observed at 10^{-19} M in the representative curve. In contrast, the corresponding bare Au curve remains unresolved within the same low-concentration range, indicating that the BP-COOH functional layer markedly extends the detectable Pb^{2+} concentration regime toward much lower concentrations.

To clarify the contribution of BP and BP-COOH to Pb^{2+} sensing, control measurements were performed on bare Au and Au+BP. The Pb^{2+} sensing results of bare Au and Au+BP are provided in Figs. S4 and S5, respectively. Bare Au shows a distinguishable Pb^{2+} response from 10^{-5} M, whereas Au+BP shows a distinguishable response from 10^{-7} M. This comparison indicates that the BP overlayer improves the Pb^{2+} sensing response relative to bare Au. By further introducing the BP-COOH functional layer, the detectable Pb^{2+} concentration is extended to 10^{-19} M, indicating that the oxygen-containing interfacial sites of BP-COOH play a key role in the ultralow-concentration Pb^{2+} response.

To examine the statistical detection behavior of Au+BP-COOH at ultralow Pb^{2+} concentrations, time-dependent sensing measurements were performed on ten independently prepared Au+BP-COOH chips. The detection statistics are summarized in Table 1, and the corresponding individual sensing results are provided in Figs. S6–S15. At 10^{-19} M Pb^{2+}, 1/10, 2/10, 4/10 and 4/10 chips show distinguishable responses at 30, 60, 90 and 120 s, respectively, indicating that this concentration is located in a time-dependent statistical detection regime. In contrast, at 10^{-18} M and higher Pb^{2+} concentrations, all ten chips show distinguishable responses at each detection time. The statistical resonance-angle shifts obtained from these ten chips are summarized in Fig. S16. These results show the time-dependent detectability of Au+BP-COOH at 10^{-19} M and the consistent detectability from 10^{-18} to 10^{-15} M.

The concentration dependence of the resonance-angle response is further summarized in Fig. 5b. For Au+BP-COOH, the resonance-angle shift $\Delta\theta$ increases with increasing Pb^{2+} concentration, and the fitted curve extends into the ultralow-concentration regime. Under the same sensing framework, bare Au only begins to show distinguishable Pb^{2+} responses at much higher concentrations. Therefore, relative to bare Au, the BP-COOH functional layer shifts the detectable Pb^{2+} concentration regime toward substantially lower concentrations on the same optical platform.

The selectivity of Au+BP-COOH was further evaluated by comparing its responses to other divalent metal ions. As shown in Fig. 5b,c, the response to Pb^{2+} appears in a substantially lower concentration regime than those of Cd^{2+}, Cu^{2+}, Zn^{2+}, Ni^{2+}, Ca^{2+} and Mg^{2+} under identical measurement conditions. The corresponding single-ion sensing results for Cd^{2+}, Cu^{2+}, Zn^{2+}, Ni^{2+}, Ca^{2+} and Mg^{2+} are provided in Figs. S25–S30. The LODs for the other metal ions are 10^{-13} M for Cd^{2+}, 10^{-13} M for Cu^{2+}, 10^{-12} M for Zn^{2+}, 10^{-10} M for Ni^{2+}, 10^{-9} M for Ca^{2+} and 10^{-8} M for Mg^{2+}. These results show that Au+BP-COOH exhibits a stronger response to Pb^{2+} than to the other tested divalent ions within the present sensing system.

The preferential response toward Pb^{2+} can be attributed to the stronger interfacial interaction between Pb^{2+} and oxygen-containing sites in BP-COOH. Pb^{2+} is a highly polarizable borderline-soft divalent ion and can form stable inner-sphere coordination with carboxylate and other oxygen-containing sites. In contrast, Ca^{2+} and Mg^{2+} are hard divalent ions with strong hydration, and their interaction with surface carboxylate sites is more strongly affected by hydration-shell retention. Transition-metal ions such as Ni^{2+}, Zn^{2+}, Cu^{2+} and Cd^{2+} can also interact with oxygen-containing groups, but their response occurs in higher concentration ranges under the same sensing conditions. This interpretation is consistent with previous reports on preferential Pb^{2+} adsorption at activated oxygen-containing sites on two-dimensional surfaces [31] and with the HSAB-based selectivity discussion used in two-dimensional-material ion sensing [22]."

Manuscript, lines 294–300:

"Mixed-ion interference and real-sample measurements were further performed to evaluate the sensing response under more complex matrix conditions. In the mixed-ion experiment, Mix was used as the matrix background, and the net Pb^{2+} response was obtained by subtracting the Mix baseline from the Mix + Pb^{2+} response. As shown in Fig. 5d,e and Fig. S31, Pb^{2+} remains distinguishable in the mixed-ion solution at 10^{-16} M. In the real-sample experiment, drinking water was used as the matrix background, and the net Pb^{2+} response was obtained by subtracting the drinking-water baseline from the drinking water + Pb^{2+} response. As shown in Fig. 5f,g and Fig. S32, Pb^{2+} remains distinguishable in drinking water at 10^{-17} M. The water-quality background of the drinking-water sample is provided in Table S1."

Comment 2

A mixed-ion interference experiment is suggested to further support the selectivity study. The current selectivity data are impressive. To further support the practical selectivity of the SPR+BP-COOH sensor, the authors are encouraged to add a mixed-ion interference experiment, in which Pb^{2+} is detected in the presence of common interfering ions.

Response:

We sincerely thank the reviewer for this constructive suggestion. We agree that single-ion selectivity measurements alone are not sufficient to demonstrate practical selectivity, because real samples

usually contain multiple coexisting metal ions. Therefore, in the revised manuscript, we added a mixed-ion interference experiment to evaluate whether Pb^{2+} can still be distinguished in the presence of common interfering divalent ions.

In the revised experiment, the mixed-ion solution, denoted as Mix, contained Cd^{2+}, Cu^{2+}, Zn^{2+}, Ni^{2+}, Ca^{2+} and Mg^{2+}. These ions were added at their corresponding LOD concentrations determined from the single-ion experiments: 10^{-13} M Cd^{2+}, 10^{-13} M Cu^{2+}, 10^{-12} M Zn^{2+}, 10^{-10} M Ni^{2+}, 10^{-9} M Ca^{2+} and 10^{-8} M Mg^{2+}. The Mix solution was first measured as the matrix baseline. Then Pb^{2+} was introduced into this mixed-ion background to obtain Mix + 10^{-16} M Pb^{2+} and Mix + 10^{-15} M Pb^{2+}. To exclude the background contribution of the interfering ions, the net Pb^{2+} response was calculated by subtracting the Mix baseline from the Mix + Pb^{2+} response.

As shown in Fig. 5d,e and Fig. S31, Pb^{2+} remains distinguishable in the mixed-ion solution at 10^{-16} M. The experiment was performed using three independently prepared Au+BP-COOH chips, and the error bars in Fig. S31 are from these independent chips. This result further supports the practical selectivity of Au+BP-COOH toward Pb^{2+} in the presence of competing divalent ions.

Changes made in the manuscript:

Manuscript, lines 242–252:

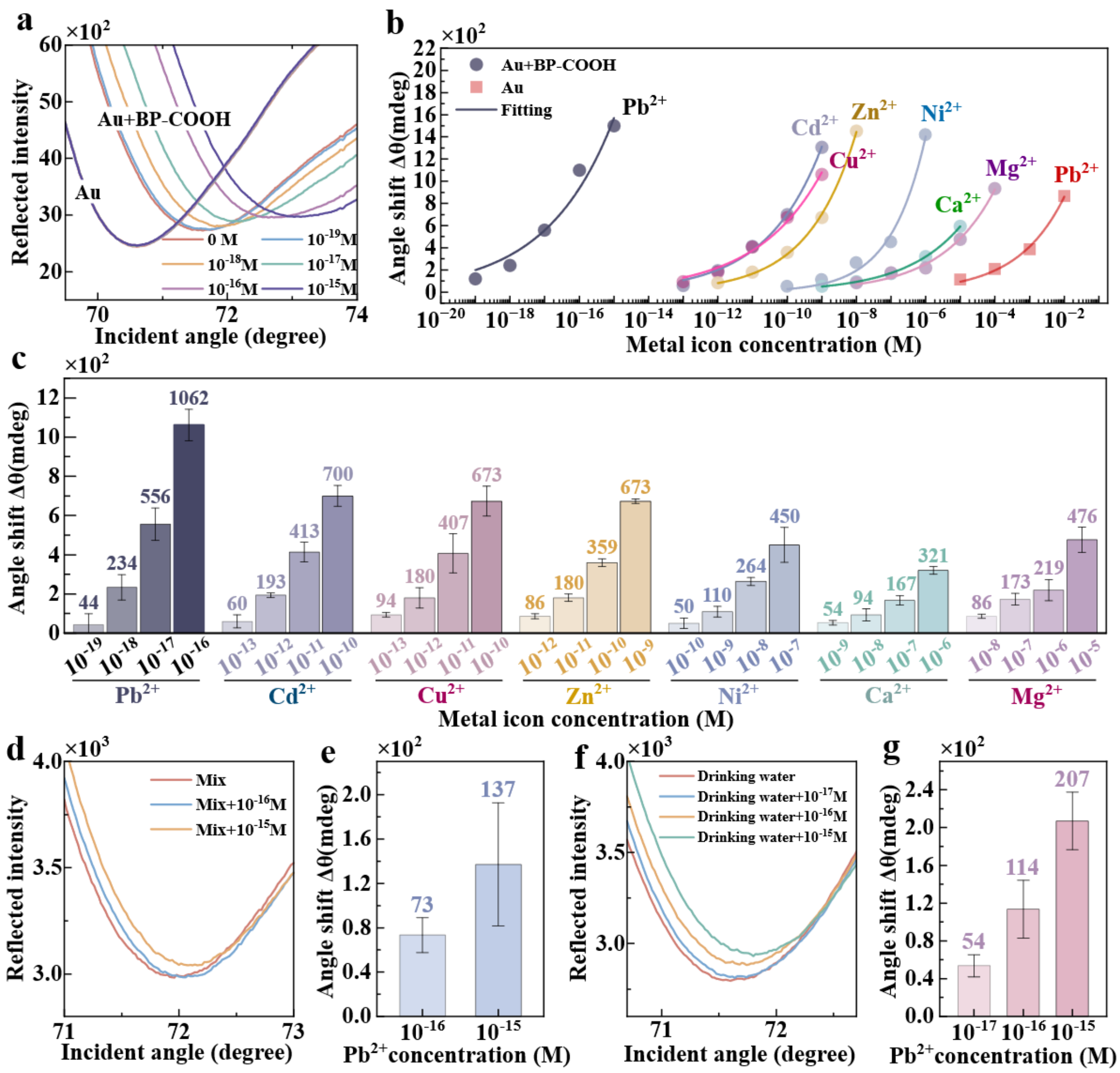

"Fig. 5. Pb^{2+} sensing response of Au+BP-COOH and comparison with other metal ions in pure

water, mixed-ion solution, and drinking water. (a) Angle-scanned reflectance curves of Au+BP-COOH measured for Pb^{2+} concentrations of 0, 10^{-19}, 10^{-18}, 10^{-17}, 10^{-16} and 10^{-15} M, together with the reflectance curve of bare Au. **(b)** Resonance-angle shift $\Delta\theta$ as a function of metal-ion concentration for Au+BP-COOH and bare Au. The data points are mean values from the corresponding statistical results in c and Fig. S4, and the solid lines represent nonlinear fitting curves. **(c)** Resonance-angle shift $\Delta\theta$ values of Au+BP-COOH measured at different concentrations of Pb^{2+}, Cd^{2+}, Cu^{2+}, Zn^{2+}, Ni^{2+}, Ca^{2+} and Mg^{2+}. The Pb^{2+} data are from the ten independently prepared Au+BP-COOH chips shown in Figs. S6–S15 and statistically summarized in Fig. S16, and the data for Cd^{2+}, Cu^{2+}, Zn^{2+}, Ni^{2+}, Ca^{2+} and Mg^{2+} are from the three independently prepared Au+BP-COOH chips shown in Figs. S25–S30. **(d)** Angle-scanned reflectance curves of Au+BP-COOH measured in Mix, Mix + 10^{-16} M Pb^{2+} and Mix + 10^{-15} M Pb^{2+}. **(e)** Net resonance-angle shift $\Delta\theta$ as a function of Pb^{2+} concentration in the mixed-ion solution. The statistical data are from Fig. S31. **(f)** Angle-scanned reflectance curves of Au+BP-COOH measured in drinking water, drinking water + 10^{-17} M Pb^{2+}, drinking water + 10^{-16} M Pb^{2+} and drinking water + 10^{-15} M Pb^{2+}. **(g)** Net resonance-angle shift $\Delta\theta$ as a function of Pb^{2+} concentration in drinking water. The statistical data are from Fig. S32."

Manuscript, lines 294–297:

"Mixed-ion interference and real-sample measurements were further performed to evaluate the sensing response under more complex matrix conditions. In the mixed-ion experiment, Mix was used as the matrix background, and the net Pb^{2+} response was obtained by subtracting the Mix baseline from the Mix + Pb^{2+} response. As shown in Fig. 5d,e and Fig. S31, Pb^{2+} remains distinguishable in the mixed-ion solution at 10^{-16} M."

Manuscript, lines 425–430:

"**Mixed-ion interference and drinking-water measurements**

For the mixed-ion interference experiment, a mixed-ion solution was used as the matrix background. The Mix solution contained Cd^{2+}, Cu^{2+}, Zn^{2+}, Ni^{2+}, Ca^{2+} and Mg^{2+} at 10^{-13}, 10^{-13}, 10^{-12}, 10^{-10}, 10^{-9} and 10^{-8} M, respectively. The Mix solution was first measured as the matrix baseline. Then, Pb^{2+} was added into the Mix solution to obtain Mix + 10^{-16} M Pb^{2+} and Mix + 10^{-15} M Pb^{2+}. The net Pb^{2+} response was calculated by subtracting the Mix baseline from the Mix + Pb^{2+} response."

Supporting Information, lines 138–141:

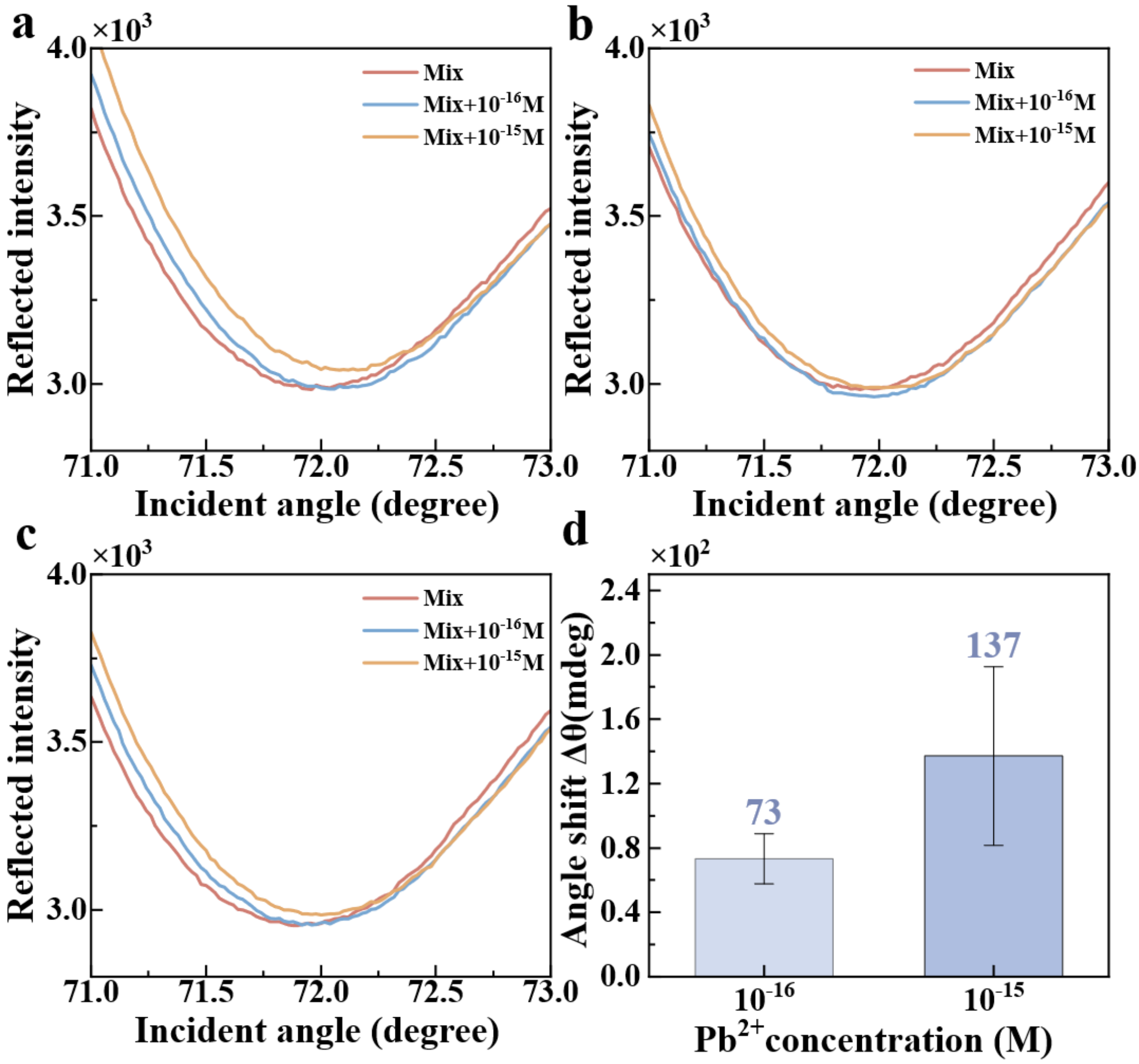

"**Fig. S31 Mixed-ion interference sensing results obtained from Au+BP-COOH. (a–c)** Angle-scanned reflectance curves of three independently prepared Au+BP-COOH chips measured in Mix, Mix + 10^{-16} M Pb^{2+} and Mix + 10^{-15} M Pb^{2+}. Mix represents the mixed-ion solution containing Cd^{2+}, Cu^{2+}, Zn^{2+}, Ni^{2+}, Ca^{2+} and Mg^{2+}. **(d)** Net resonance-angle shift Δθ as a function of Pb^{2+} concentration in the mixed-ion solution. Error bars are from three independently prepared Au+BP-COOH chips."

Comment 3

More details on ultralow-concentration preparation would be helpful. The authors have described the stepwise dilution procedure. To further support the 10^{-19} M detection claim, it would be useful to briefly clarify container pretreatment, water purity, and whether the ultralow-concentration Pb^{2+} solutions were freshly prepared before measurement.

Response:

We thank the reviewer for this helpful suggestion. We understand that, for ultralow-concentration Pb^{2+} measurements, the dilution procedure itself is not sufficient unless possible carryover and background contamination during solution preparation are also carefully controlled. Therefore, we revised the Methods section to provide more details on the preparation of ultralow-concentration ion solutions.

In the revised manuscript, we clarified that all ion solutions were prepared in a clean laboratory. The

purchased single-element standard solutions were first converted to 10^{-2} M stock solutions and then diluted stepwise by a factor of 10 using pure water to obtain the required concentration series. For each target concentration and each dilution step, a fresh container and a fresh pipette tip were used. Containers and pipette tips were not reused, which was intended to minimize carryover between different concentration levels. We also clarified that the ultralow-concentration solutions were freshly prepared before measurement.

In addition, the sensing results were not interpreted only from a single ultralow-concentration curve. The 10^{-19} M Pb^{2+} response was further evaluated by time-dependent measurements using ten independently prepared Au+BP-COOH chips, with individual results shown in Figs. S6–S15 and statistical results shown in Fig. S16. The distinguishability threshold was also defined based on the angular scanning step and the pure-water baseline stability of Au+BP-COOH, as shown in Fig. S33. These procedures and controls were added to make the ultralow-concentration preparation and measurement more transparent.

Changes made in the manuscript:

Manuscript, lines 411–416:

"**Preparation of ion solutions**

All ion solutions were prepared in the clean laboratory. The purchased single-element standard solutions were first converted to 10^{-2} M stock solutions and then diluted stepwise by a factor of 10 using pure water to obtain the required concentration series. The pH of the test solutions remained at 6.3, and all ions were measured under the same matrix conditions. For each target concentration and each dilution step, a fresh container and a fresh pipette tip were used to minimize carryover between concentrations. Containers and pipette tips were not reused, and the ultralow-concentration solutions were freshly prepared before measurement."

Manuscript, lines 265–272:

"To examine the statistical detection behavior of Au+BP-COOH at ultralow Pb^{2+} concentrations, time-dependent sensing measurements were performed on ten independently prepared Au+BP-COOH chips. The detection statistics are summarized in Table 1, and the corresponding individual sensing results are provided in Figs. S6–S15. At 10^{-19} M Pb^{2+}, 1/10, 2/10, 4/10 and 4/10 chips show distinguishable responses at 30, 60, 90 and 120 s, respectively, indicating that this concentration is located in a time-dependent statistical detection regime. In contrast, at 10^{-18} M and higher Pb^{2+} concentrations, all ten chips show distinguishable responses at each detection time. The statistical resonance-angle shifts obtained from these ten chips are summarized in Fig. S16. These results show the time-dependent detectability of Au+BP-COOH at 10^{-19} M and the consistent detectability from 10^{-18} to 10^{-15} M."

Manuscript, lines 419–423:

"The resonance-angle shift $\Delta\theta$ was used to evaluate the sensing response. A response was considered distinguishable when $\Delta\theta$ exceeded 21 mdeg. This operational distinguishability threshold was selected according to the angular scanning step of the SPR measurement and the baseline stability of Au+BP-COOH in pure water. Repeated pure-water measurements on the same Au+BP-COOH chip over 50 min showed a maximum resonance-angle drift of 21 mdeg and a standard deviation of 0.00714 deg (Fig. S33). The LOD was defined as the lowest concentration at which a distinguishable response was obtained under the corresponding sensing condition."

Supporting Information, lines 81–83:

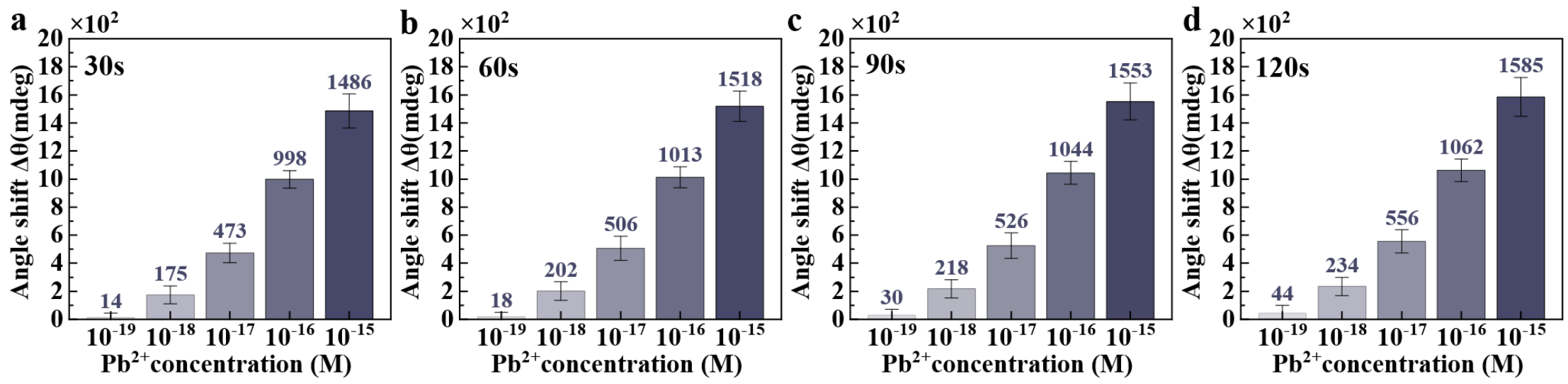

"**Fig. S16 Statistical Pb^{2+} sensing results obtained from the ten Au+BP-COOH chips shown in Figs. S6–S15 at different detection times. (a–d)** Resonance-angle shift Δθ as a function of Pb^{2+} concentration at 30, 60, 90 and 120 s, respectively. Error bars are from the ten independently prepared Au+BP-COOH chips shown in Figs. S6–S15."

Supporting Information, lines 158–160:

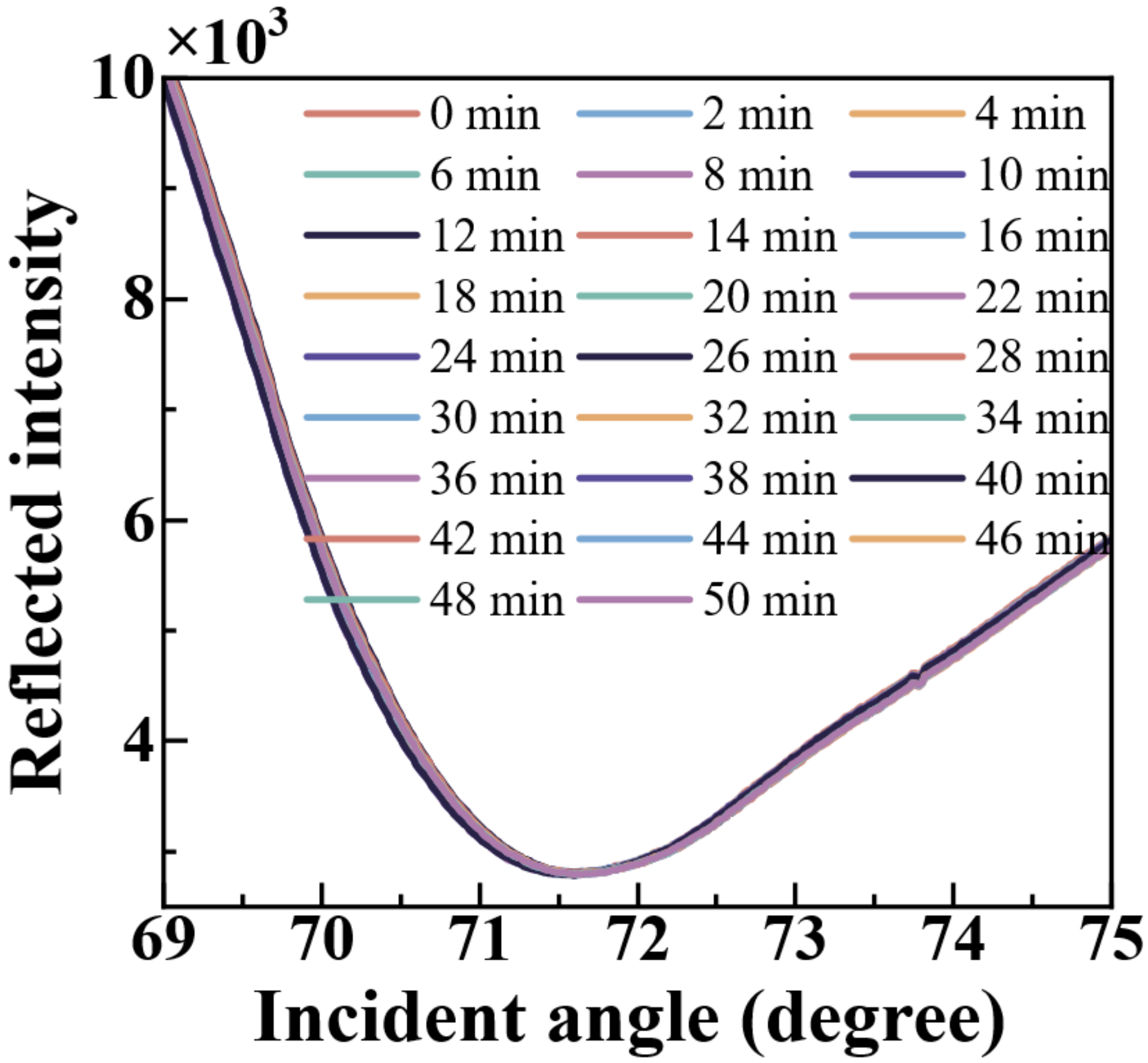

"**Fig. S33 Baseline stability of Au+BP-COOH in pure water.** Angle-scanned reflectance curves recorded on the same Au+BP-COOH chip in pure water at 2 min intervals over 50 min. The maximum resonance-angle drift during the 50 min measurement was 21 mdeg, and the standard deviation of the resonance angle was 0.00714 deg."

Comment 4

The baseline definition should be clarified. The manuscript defines Δθ relative to pure water. The authors should briefly specify whether a pure-water baseline was measured before each ion-sensing

experiment and whether the same baseline procedure was applied to Pb^{2+} and competing-ion measurements.

Response:

We thank the reviewer for this helpful comment. We agree that the baseline definition should be stated more explicitly, because the resonance-angle shift $\Delta\theta$ is meaningful only when the reference baseline is clearly defined.

In the revised manuscript, we clarified that, for pure-water and single-ion sensing measurements, pure water was first measured on the same chip as the baseline before introducing the ion solution. The resonance-angle shift $\Delta\theta$ was then calculated relative to this corresponding pure-water baseline. This same baseline procedure was applied to Pb^{2+} and to the competing-ion measurements, including Cd^{2+}, Cu^{2+}, Zn^{2+}, Ni^{2+}, Ca^{2+} and Mg^{2+}. Therefore, the Pb^{2+} response and the competing-ion responses were evaluated using the same baseline definition under the same matrix condition.

For the time-dependent Pb^{2+} detection statistics, ten independently prepared Au+BP-COOH chips were measured from pure water to increasing Pb^{2+} concentrations, and the pure-water measurement served as the baseline for each chip. For the single-ion selectivity measurements, three independently prepared Au+BP-COOH chips were used for each competing ion, and the corresponding pure-water baseline was measured before the ion response was evaluated.

For the mixed-ion and drinking-water experiments, we used the corresponding matrix solution as the baseline instead of pure water. In the mixed-ion experiment, Mix was first measured as the matrix baseline, and the net Pb^{2+} response was calculated by subtracting the Mix baseline from the Mix + Pb^{2+} response. In the drinking-water experiment, drinking water was first measured as the matrix baseline, and the net Pb^{2+} response was calculated by subtracting the drinking-water baseline from the drinking water + Pb^{2+} response. These definitions are now clarified in the Methods section and in the Results discussion.

Changes made in the manuscript:

Manuscript, lines 398–410:

"SPR measurements were performed on a Dynechem SPR system using 650 nm incident light at 25 °C. The angular scanning range was 65°–80°, and the angular step was 19–21 mdeg. For each measurement, 400 μL of sample solution was introduced into the flow cell at a flow rate of 200 μL/min. The resonance angle was directly read from the angle corresponding to the minimum reflectance point without curve fitting.

For Pb^{2+} time-dependent sensing measurements, the SPR response was recorded at 30, 60, 90 and 120 s. Unless otherwise specified, the resonance-angle shift $\Delta\theta$ used for statistical comparison was extracted at 120 s. For pure-water sensing measurements and single-ion sensing measurements, pure water was first measured on the same chip as the baseline before the ion solution was introduced, and $\Delta\theta$ was defined relative to this corresponding pure-water baseline. The same baseline procedure was applied to Pb^{2+} and to the competing-ion measurements. For mixed-ion and drinking-water measurements, the net $\Delta\theta$ was obtained by subtracting the corresponding matrix baseline.

For the time-dependent Pb^{2+} detection statistics, ten independently prepared Au+BP-COOH chips were measured from pure water to increasing Pb^{2+} concentrations. For single-ion selectivity, mixed-ion interference and drinking-water measurements, three independently prepared Au+BP-COOH chips were used unless otherwise specified. Error bars represent the standard deviation from independently prepared chips."

Manuscript, lines 294–300:

"Mixed-ion interference and real-sample measurements were further performed to evaluate the sensing response under more complex matrix conditions. In the mixed-ion experiment, Mix was used as the matrix background, and the net Pb^{2+} response was obtained by subtracting the Mix baseline from the Mix + Pb^{2+} response. As shown in Fig. 5d,e and Fig. S31, Pb^{2+} remains distinguishable in the mixed-ion solution at 10^{-16} M. In the real-sample experiment, drinking water was used as the matrix background, and the net Pb^{2+} response was obtained by subtracting the drinking-water baseline from the drinking water + Pb^{2+} response. As shown in Fig. 5f,g and Fig. S32, Pb^{2+} remains distinguishable in drinking water at 10^{-17} M. The water-quality background of the drinking-water sample is provided in Table S1."

Manuscript, lines 426–435:

"For the mixed-ion interference experiment, a mixed-ion solution was used as the matrix background. The Mix solution contained Cd^{2+}, Cu^{2+}, Zn^{2+}, Ni^{2+}, Ca^{2+} and Mg^{2+} at 10^{-13}, 10^{-13}, 10^{-12}, 10^{-10}, 10^{-9} and 10^{-8} M, respectively. The Mix solution was first measured as the matrix baseline. Then, Pb^{2+} was added into the Mix solution to obtain Mix + 10^{-16} M Pb^{2+} and Mix + 10^{-15} M Pb^{2+}. The net Pb^{2+} response was calculated by subtracting the Mix baseline from the Mix + Pb^{2+} response. Drinking water was used as the real-sample matrix for Pb^{2+} sensing. The water-quality background of the drinking-water sample is provided in Table S1. During measurement, drinking water was first measured as the matrix baseline. Then, Pb^{2+} was added into drinking water to obtain drinking water + 10^{-17} M Pb^{2+}, drinking water + 10^{-16} M Pb^{2+} and drinking water + 10^{-15} M Pb^{2+}. The net Pb^{2+} response was calculated by subtracting the drinking-water baseline from the drinking water + Pb^{2+} response."

Comment 5

The LOD comparison in Figure 6 should be described more clearly. I noticed that the LOD values reported in different sensing systems may use different units or concentration expressions. If the authors have converted these values into the same unit for comparison, this should be clearly stated in the figure caption or main text to make the comparison more transparent and rigorous.

Response:

We thank the reviewer for this helpful suggestion. We agree that the LOD comparison in Fig. 6 should clearly state how values from different reports were compared. In the revised manuscript, we clarified that the LOD values from the representative literature reports were converted to the same molar concentration unit, M, before plotting in Fig. 6. We also listed the converted LOD values in the main text for SPR sensors, fluorescent probes, colorimetric/LSPR probes, and electrochemical/PEC biosensors to make the comparison more transparent.

Changes made in the manuscript:

Manuscript, lines 316–318:

"**Fig. 6. Comparison of the Pb^{2+} LOD of this work with representative reports from four sensing categories.** LOD values from representative SPR sensors, fluorescent probes, colorimetric/LSPR probes, and electrochemical/PEC biosensors are plotted on the concentration axis, together with the result of this work. All LOD values were converted to molar concentration (M) before plotting. The corresponding references are [9–13,17–20,33–39]."

Manuscript, lines 319–329:

"**Comparison with representative Pb^{2+} sensing strategies.** To position the present sensing performance in the context of reported Pb^{2+} detection technologies, the LOD of Au+BP-COOH was compared with representative studies from four categories, including SPR sensors, fluorescent probes, colorimetric/LSPR probes, and electrochemical/PEC biosensors, as summarized in Fig. 6. Within the representative SPR studies, previously reported LODs span from 4.8×10^{-9} M to 5.0×10^{-15} M, including 4.8×10^{-9} M for a fiber-based SPR sensor [19], 8.0×10^{-11} M for a label-free SPR platform based on DNAzyme-gold nanoparticle conjugates [17], and 5.0×10^{-15} M for an amplified SPR format using a Pb^{2+}-dependent DNAzyme and a hemin/G-quadruplex label [20]. Representative fluorescent probes reported LODs of 4.8×10^{-8} M, 9.6×10^{-11} M, and 3.8×10^{-9} M [9,10,33]. Representative colorimetric/LSPR probes reported LODs in the range of 1.0×10^{-7} to 2.4×10^{-12} M [18,34-37]. Representative electrochemical and PEC biosensors further reached 1.5×10^{-11} M, 1.67×10^{-14} M, 1.26×10^{-15} M, 1.67×10^{-11} M, and 7.0×10^{-17} M, respectively [11-13,38,39]."

Reviewer 3

The work by Yang et al. reports an interesting approach to ultrasensitive Pb2+ detection by engineering a carboxyl-functionalized black phosphorene layer onto a prism-coupled Au-film SPR platform. this sensor achieves an ultralow detection 27 limit of 10–19 M, 13 orders of magnitude lower than that of bare Au-SPR, and exhibits outstanding selectivity against Cd2+, Cu2+, Zn2+, Ni2+, Ca2+, and Mg2+. The topic is of considerable relevance to the analytical and environmental sensing communities. The paper is generally well structured and the motivation is clearly articulated. However, the manuscript in its present form does not provide sufficient experimental validation and analytical rigor to fully support several of the central claims. The following suggestions/questions should be carefully addressed by the authors.

Response to Reviewer 3

We sincerely thank Reviewer #3 for the careful and critical evaluation of our manuscript. We are grateful that the reviewer recognized the relevance of the topic, the interest of the sensing strategy, and the overall organization of the work. At the same time, we fully appreciate the reviewer's central concern that several key claims, especially the ultralow Pb^{2+} detection limit, the selectivity, and the proposed interfacial enhancement mechanism, require stronger experimental validation and clearer analytical support.

We have taken these comments very seriously. In the revised manuscript, we made substantial revisions and added new experimental data to improve the rigor and transparency of the study. Specifically, we clarified the definition of the detection limit and quantified the distinguishability threshold using both the angular scanning step and a pure-water baseline stability test. We further added time-dependent Pb^{2+} sensing statistics from ten independently prepared Au+BP-COOH chips to address the finite-ion-number issue at 10^{-19} M. The revised manuscript now clearly distinguishes the time-dependent statistical detection behavior at 10^{-19} M from the consistent detectability observed from 10^{-18} to 10^{-15} M.

We also strengthened the selectivity study by adding independent-chip measurements for Cd^{2+}, Cu^{2+}, Zn^{2+}, Ni^{2+}, Ca^{2+} and Mg^{2+}, together with a mixed-ion interference experiment and drinking-water measurements. To better support the mechanistic interpretation, we added refractive-index calibration experiments comparing bare Au, Au+Graphene, Au+MoS_2, Au+BP and Au+BP-COOH, and we added Pb^{2+} sensing controls using bare Au and Au+BP. These comparisons help separate the BP-related optical contribution from the BP-COOH-related Pb^{2+} interaction contribution. We also revised the discussion of Pb^{2+} selectivity by adding chemical reasoning based on oxygen-containing coordination sites, hydration effects, ion polarizability and HSAB considerations.

In addition, we added experimental evidence for BP-COOH stability, chip-to-chip reproducibility, storage stability, and regeneration behavior. Raman spectra of BP-COOH during Day 1–Day 7 storage, AFM characterization of independently prepared Au+BP-COOH chips, storage-stability measurements, and EDTA-based regeneration tests are now included in the revised manuscript and Supporting Information. The solution-preparation procedure, baseline definition, flow-cell measurement conditions, and treatment of ultralow-concentration solutions have also been clarified in the Methods section.

Overall, the reviewer's comments helped us substantially improve both the experimental foundation and the logical presentation of the manuscript. We hope that the revised version now provides a clearer and more rigorous basis for the reported Au+BP-COOH SPR sensing performance. Detailed

point-by-point responses are provided below.

Comment 1

The built-in electric field mechanism is not directly supported. The manuscript mainly relies on an energy-level schematic, but provides no direct evidence to verify the proposed Au/BP interfacial field. The authors should supply a physically rigorous explanation, grounded in SPR optics, for how an interfacial field would enhance angular sensitivity.

Response:

We sincerely thank the reviewer for this critical and very valuable comment. We fully understand that the reviewer is concerned about the physical basis of the proposed enhancement mechanism, especially how the Au/BP-based interface modifies the SPR optical response and leads to a higher angular sensitivity. This comment helped us substantially reorganize the mechanism section and strengthen the connection between the experimental SPR response and the proposed interfacial field effect.

In the revised manuscript, we added an experimental refractive-index calibration using five Au-based interfaces: bare Au, Au+Graphene, Au+MoS_2, Au+BP and Au+BP-COOH (Fig. 3). Au+Graphene and Au+MoS_2 were included as reference two-dimensional overlayer structures because they also possess high-index confinement and interlayer-coupling-related optical characteristics. Therefore, this comparison allows us to evaluate whether the enhanced angular response is a general consequence of adding a two-dimensional overlayer or is more strongly associated with the BP-based interface. Under the same refractive-index variation, the measured angular sensitivities are 127 deg/RIU for bare Au, 157 deg/RIU for Au+Graphene, 168 deg/RIU for Au+MoS_2, 256 deg/RIU for Au+BP and 259 deg/RIU for Au+BP-COOH. Although Au+Graphene and Au+MoS_2 both increase the angular sensitivity relative to bare Au, their sensitivities remain lower than those of Au+BP and Au+BP-COOH. Therefore, the stronger angular response of the BP-based interfaces is supported by experimental SPR refractive-index calibration.

We also revised the physical explanation to make it more closely connected to SPR optics. In angular SPR measurements, the resonance angle is determined by the phase-matching condition between the in-plane wave vector of incident light and the surface plasmon mode at the metal/dielectric interface. When the evanescent field is more strongly confined near the sensing interface, the surface plasmon mode becomes more sensitive to refractive-index perturbations in the sensing medium, resulting in a larger resonance-angle shift for the same refractive-index change. In the revised manuscript, we explain that the BP overlayer modifies the optical boundary condition at the Au interface. The relatively high refractive index of BP increases the dielectric contrast at the plasmonic interface, compresses the effective mode volume of the evanescent field, and strengthens local field confinement near the sensing region. The multilayer BP structure can further contribute through interlayer coupling, which enhances optical confinement (Fig. 4a,b).

For the built-in electric field, we clarified the proposed physical origin in terms of interfacial charge redistribution after Au/BP contact. Because Au and BP have different work functions, contact between these two components can drive directional charge redistribution at the interface, leading to the formation of an interfacial built-in electric field. In the SPR structure, this charge-transfer-induced interfacial field can strengthen local electromagnetic coupling near the sensing region and further regulate the local field environment at the Au/BP interface (Fig. 4c,d). The transfer-matrix simulation in Fig. S3 provides theoretical support for the enhanced angular response and electric-

field distribution after introducing a BP overlayer, while the experimental refractive-index calibration in Fig. 3 provides the direct experimental basis for evaluating the actual Au+BP-COOH sensing interface.

Thus, the revised manuscript addresses the reviewer's concern through three connected levels of evidence and explanation: first, Fig. 3 experimentally verifies the enhanced angular sensitivity of Au+BP and Au+BP-COOH; second, Fig. 4a,b explains the SPR-optical origin of the stronger near-field response through dielectric contrast, field confinement and interlayer coupling; and third, Fig. 4c,d explains how Au/BP interfacial charge redistribution can generate a built-in electric field that contributes to local electromagnetic coupling. Together with the simulation results in Fig. S3, these revisions provide a more rigorous optical and physical basis for the sensitivity enhancement observed in the BP-based SPR interfaces.

Changes made in the manuscript:

Manuscript, lines 182–185:

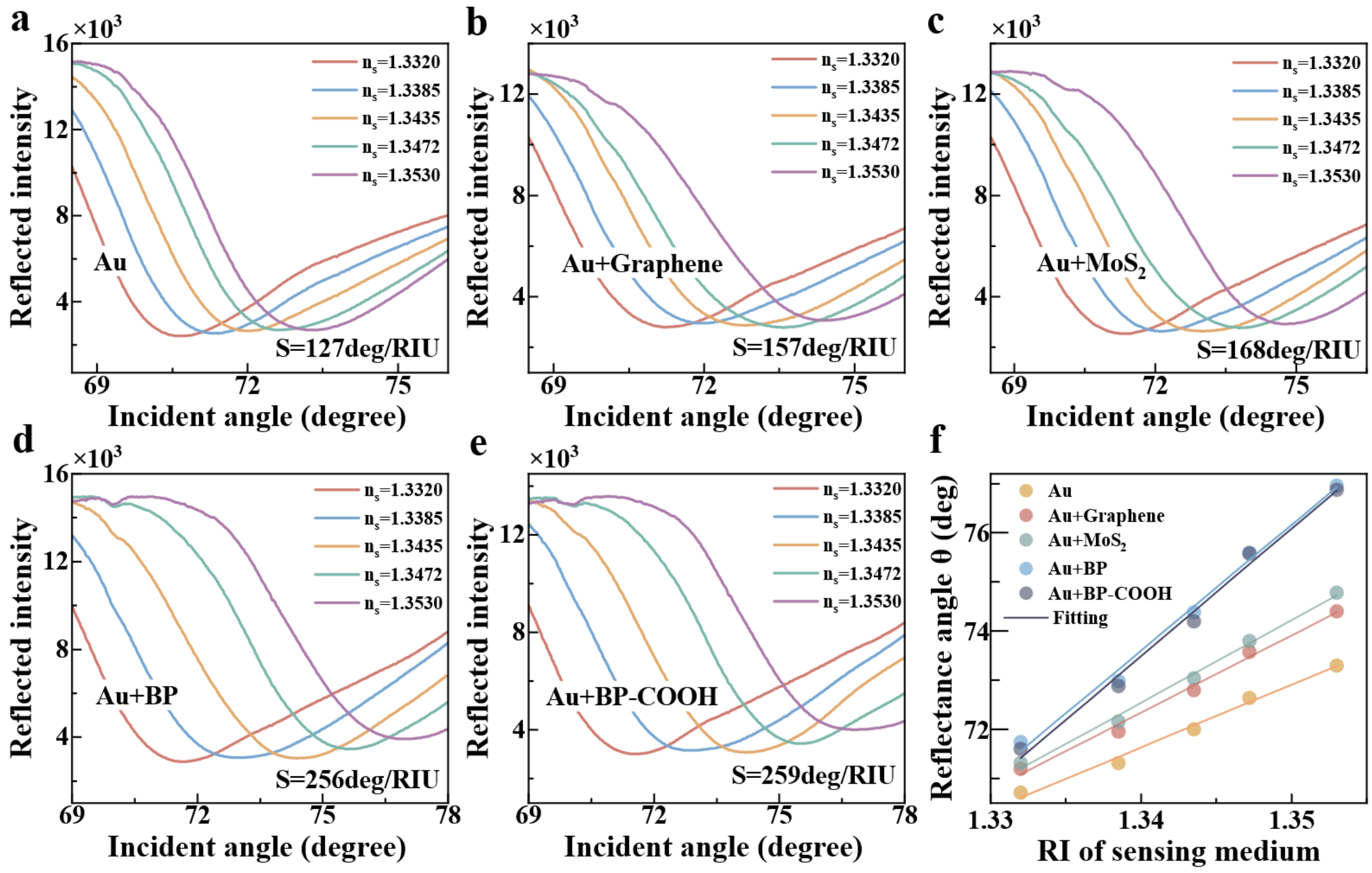

"**Fig. 3. Experimental refractive-index calibration of bare Au, Au+Graphene, Au+MoS_2, Au+BP and Au+BP-COOH. (a–e)** Angle-scanned reflectance curves of bare Au, Au+Graphene, Au+MoS_2, Au+BP and Au+BP-COOH measured in sensing media with refractive indices n_s = 1.3320, 1.3385, 1.3435, 1.3472 and 1.3530, respectively. **(f)** Resonance angle as a function of the refractive index of the sensing medium for bare Au, Au+Graphene, Au+MoS_2, Au+BP and Au+BP-COOH, together with linear fitting curves."

Manuscript, lines 187–192:

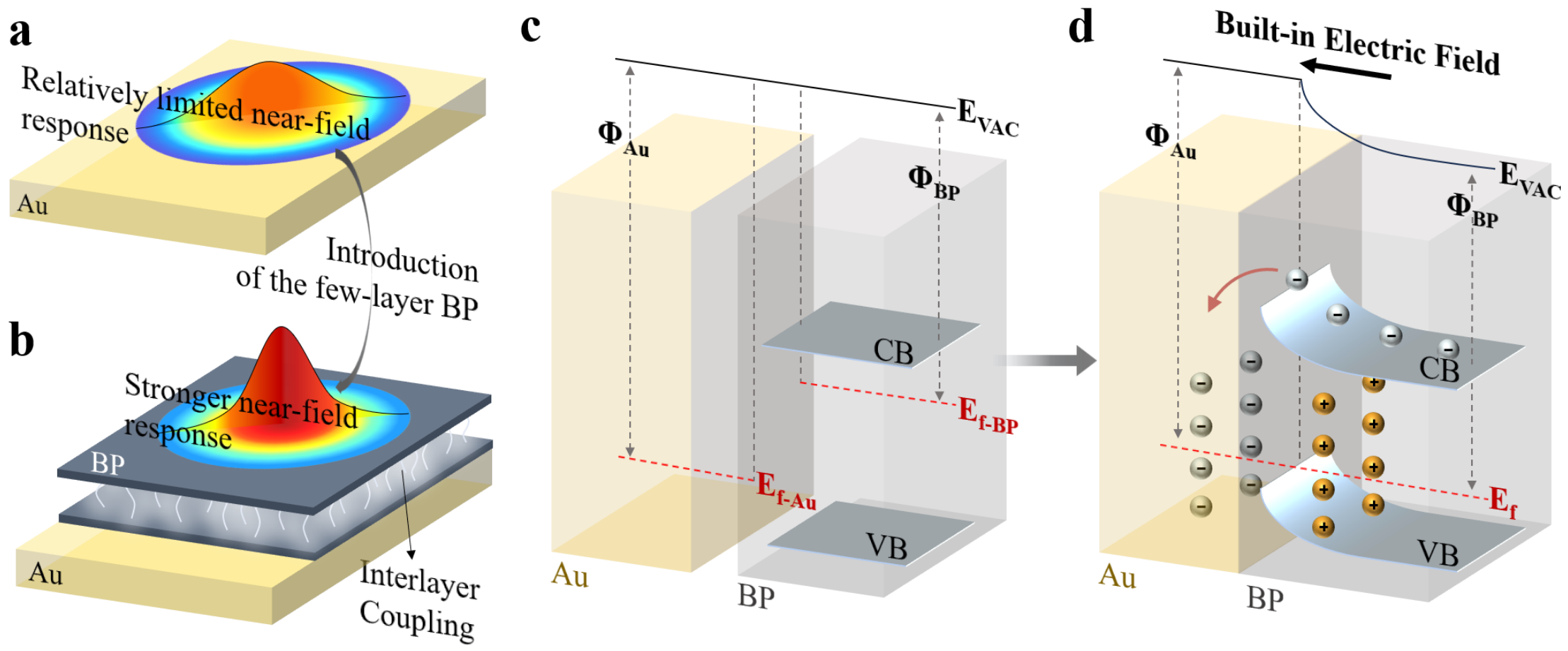

"**Fig. 4. Schematic physical interpretation of BP-enhanced near-field response at the SPR interface.** **(a)** Schematic near-field distribution at the Au/sensing-medium interface, showing a relatively limited near-field response. **(b)** Schematic near-field distribution after introduction of the BP overlayer, showing enhanced field confinement and a stronger near-field response; interlayer coupling within the multilayer BP structure is also illustrated. **(c)** Schematic energy-level alignment of Au and BP before interfacial charge redistribution, showing the work functions and band positions of the two components. **(d)** Schematic interfacial charge-transfer process after Au/BP contact, showing the formation of a built-in electric field and the resulting enhancement of local field coupling at the interface."

Manuscript, lines 193–215:

"**Optical basis and physical interpretation of sensitivity enhancement by the BP-based overlayer.** To clarify the optical basis for the improved sensing performance of Au+BP-COOH, the refractive-index-dependent angular SPR responses of bare Au, Au+Graphene, Au+MoS_2, Au+BP and Au+BP-COOH were experimentally compared under the same sensing framework. As shown in Fig. 3a–e, the angle-scanned reflectance curves of all five structures shift toward larger incident angles when the refractive index of the sensing medium increases from n_s = 1.3320 to 1.3530. However, the magnitude of the resonance-angle displacement is different for different interfacial structures.

The corresponding resonance angles are summarized in Fig. 3f. Linear fitting gives angular sensitivities of 127 deg/RIU for bare Au, 157 deg/RIU for Au+Graphene, 168 deg/RIU for Au+MoS_2, 256 deg/RIU for Au+BP and 259 deg/RIU for Au+BP-COOH. Compared with bare Au, the introduction of two-dimensional overlayers increases the angular response of the Au-film SPR structure to refractive-index variation. Au+Graphene and Au+MoS_2 were used as reference two-dimensional overlayer structures because they also possess high-index confinement and interlayer-coupling-related optical characteristics. However, their angular sensitivities remain lower than those of Au+BP and Au+BP-COOH under the same refractive-index calibration conditions. This comparison indicates that the BP-based overlayer provides a stronger optical response than the other two-dimensional overlayers examined here. In addition, the angular sensitivities of Au+BP and Au+BP-COOH are close to each other, indicating that carboxyl functionalization preserves the BP-related optical response while introducing oxygen-containing interfacial sites for subsequent Pb^{2+} interaction.

The simulated angular SPR response and electric-field distribution of the Au-film structure with and without a BP overlayer are provided in Fig. S3. The simulation shows the same enhancement trend

after introducing the BP overlayer and provides a theoretical reference for the BP-induced optical response. In the experimental Au+BP-COOH structure, the BP-COOH functional layer contains polymer-functionalized BP nanosheets, and therefore the experimental refractive-index calibration in Fig. 3 is used as the direct basis for evaluating the optical response of the actual sensing interface."

Manuscript, lines 216–231:

"The physical origin of this enhanced near-field response is schematically summarized in Fig. 4. As illustrated in Fig. 4a,b, the introduction of BP changes the interfacial optical environment of the Au-film SPR structure. BP possesses a relatively high refractive index over a broad spectral range, and its introduction onto the Au surface creates a stronger dielectric contrast at the plasmonic interface. Under this condition, the effective mode volume of the evanescent field is compressed, and the electromagnetic field becomes more strongly localized near the sensing interface. In addition, the multilayer structure of BP introduces interlayer coupling, which further enhances optical confinement and contributes to a stronger near-field response than that of the Au/sensing-medium structure.

A second contribution is illustrated in Fig. 4c,d. Because Au and BP have different work functions, contact between the two components can drive directional interfacial charge redistribution. This process leads to the formation of a built-in electric field at the Au/BP interface, which can further strengthen local electromagnetic coupling near the sensing region. In this sense, the role of BP in improving SPR sensitivity is not limited to adding an ultrathin overlayer, but also involves interfacial regulation of the local field environment. BP also exhibits a high carrier mobility, reported up to approximately 10^3 $cm^2/(V\cdot s)$ at room temperature [24], which is favorable for efficient interfacial charge response. Taken together, the high-index confinement effect, multilayer coupling effect, and interfacial charge-transfer effect provide a physically reasonable interpretation for the stronger optical response of Au+BP and Au+BP-COOH observed in Fig. 3."

Supporting Information, lines 29–34:

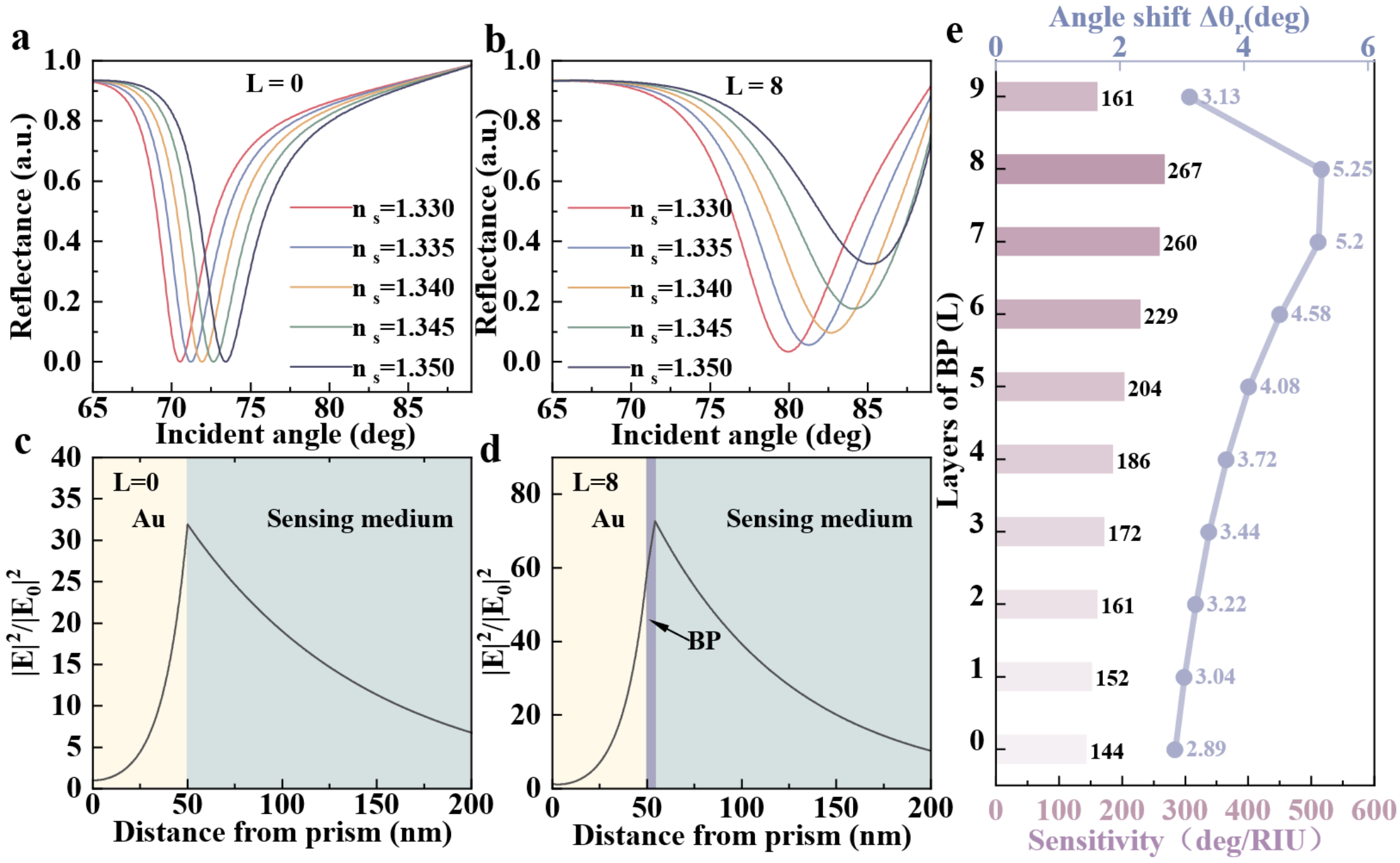

"Fig. S3 Simulated angular SPR response, electric-field distribution, and layer-number-

dependent sensitivity of the Au-film SPR structure with and without a BP overlayer. (a) Simulated angle-scanned reflectance curves of bare Au with L = 0 at sensing-medium refractive indices n_s = 1.330, 1.335, 1.340, 1.345 and 1.350. **(b)** Simulated angle-scanned reflectance curves of the BP-modified Au structure with L = 8 at sensing-medium refractive indices n_s = 1.330, 1.335, 1.340, 1.345 and 1.350. **(c)** Normalized electric-field intensity distribution of bare Au with L = 0. **(d)** Normalized electric-field intensity distribution of the BP-modified Au structure with L = 8. **(e)** Simulated angular sensitivity and total resonance-angle shift as a function of the number of BP layers."

Comment 2

The selectivity assessment in Fig. 5b and 5c is based exclusively on single-ion measurements and therefore does not provide a rigorous evaluation of selectivity under competitive conditions. The authors should perform competitive adsorption experiments, for example by measuring Pb^{2+} response in the presence of relevant interfering ions, such as Cu^{2+} and Cd^{2+}. In addition, the preferential response toward Pb^{2+} is currently attributed only to carboxylate coordination, which is insufficient to explain the markedly weaker response to other divalent ions. Stronger chemical evidence, binding analysis, or mechanistic support is therefore needed to substantiate the proposed selectivity mechanism.

Response:

We sincerely thank the reviewer for this careful and important criticism. We fully understand that single-ion measurements provide an initial evaluation of ion response, whereas competitive matrix measurements are needed to more rigorously assess selectivity under practical sensing conditions. We also appreciate the reviewer's point that carboxylate coordination by itself does not sufficiently explain the markedly different responses among Pb^{2+} and other divalent ions. This comment was very helpful for strengthening both the experimental selectivity evaluation and the chemical explanation of the Pb^{2+} preferential response.

To address the competitive selectivity concern, we added a mixed-ion interference experiment. In this experiment, the mixed-ion solution, denoted as Mix, contained Cd^{2+}, Cu^{2+}, Zn^{2+}, Ni^{2+}, Ca^{2+} and Mg^{2+}. Thus, the interfering ions specifically mentioned by the reviewer, Cu^{2+} and Cd^{2+}, were included together with other common divalent ions. The concentrations of the interfering ions were set at their corresponding LOD values obtained from the single-ion measurements: 10^{-13} M Cd^{2+}, 10^{-13} M Cu^{2+}, 10^{-12} M Zn^{2+}, 10^{-10} M Ni^{2+}, 10^{-9} M Ca^{2+} and 10^{-8} M Mg^{2+}. The Mix solution was first measured as the matrix baseline, and Pb^{2+} was then added into this competitive ion background to obtain Mix + 10^{-16} M Pb^{2+} and Mix + 10^{-15} M Pb^{2+}. The net Pb^{2+} response was calculated by subtracting the Mix baseline from the Mix + Pb^{2+} response. As shown in Fig. 5d,e and Fig. S31, Pb^{2+} remains distinguishable in the mixed-ion solution at 10^{-16} M. This experiment was performed using three independently prepared Au+BP-COOH chips, and the error bars are shown in Fig. S31.

We also revised the chemical explanation of Pb^{2+} selectivity. In the original version, the discussion focused mainly on Pb^{2+} interaction with carboxylate sites, which did not sufficiently explain why other divalent ions produced weaker responses. In the revised manuscript, we describe the sensing behavior as a preferential Pb^{2+} response and further explain this preference using oxygen-containing coordination sites, hydration effects, ion polarizability and HSAB considerations. The preferential response is attributed to the stronger interfacial interaction between Pb^{2+} and oxygen-containing

sites in BP-COOH. Pb^{2+} is a highly polarizable borderline-soft divalent ion and can form stable inner-sphere coordination with carboxylate and other oxygen-containing sites. In contrast, Ca^{2+} and Mg^{2+} are hard divalent ions with strong hydration, and their interaction with surface carboxylate sites is more strongly affected by hydration-shell retention. Transition-metal ions such as Ni^{2+}, Zn^{2+}, Cu^{2+} and Cd^{2+} can also interact with oxygen-containing groups, but their responses occur in higher concentration ranges under the same sensing conditions. This interpretation is supported by the single-ion measurements in Fig. 5b,c and Figs. S25–S30, the mixed-ion interference experiment in Fig. 5d,e and Fig. S31, and the added discussion based on oxygen-containing coordination sites, hydration effects, ion polarizability and HSAB considerations.

Changes made in the manuscript:

Manuscript, lines 242–252:

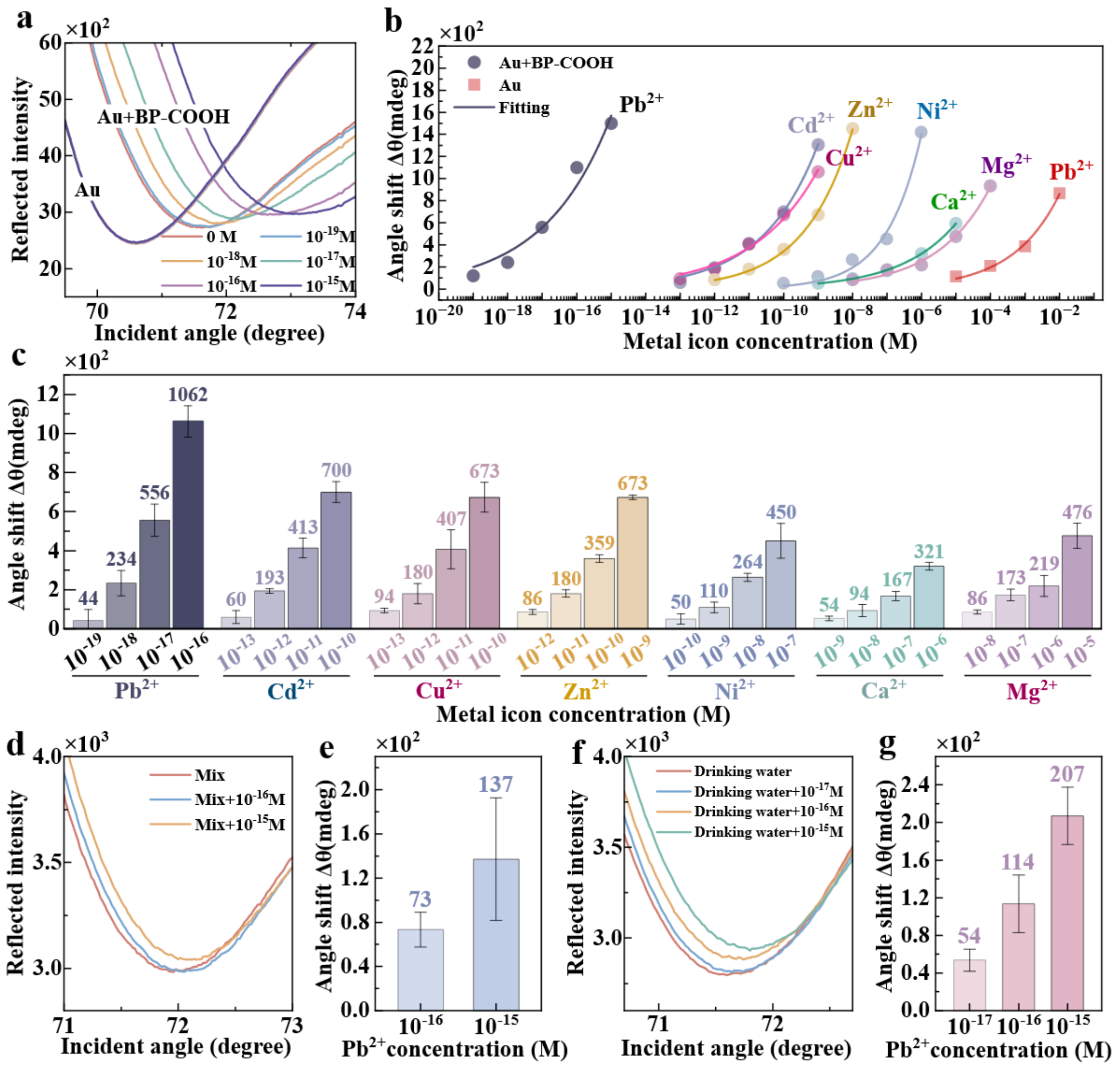

"Fig. 5. Pb^{2+} sensing response of Au+BP-COOH and comparison with other metal ions in pure water, mixed-ion solution, and drinking water. (a) Angle-scanned reflectance curves of Au+BP-COOH measured for Pb^{2+} concentrations of 0, 10^{-19}, 10^{-18}, 10^{-17}, 10^{-16} and 10^{-15} M, together with the reflectance curve of bare Au. **(b)** Resonance-angle shift Δθ as a function of metal-ion concentration for Au+BP-COOH and bare Au. The data points are mean values from the corresponding statistical results in c and Fig. S4, and the solid lines represent nonlinear fitting curves.

(c) Resonance-angle shift Δθ values of Au+BP-COOH measured at different concentrations of Pb^{2+}, Cd^{2+}, Cu^{2+}, Zn^{2+}, Ni^{2+}, Ca^{2+} and Mg^{2+}. The Pb^{2+} data are from the ten independently prepared Au+BP-COOH chips shown in Figs. S6–S15 and statistically summarized in Fig. S16, and the data for Cd^{2+}, Cu^{2+}, Zn^{2+}, Ni^{2+}, Ca^{2+} and Mg^{2+} are from the three independently prepared Au+BP-COOH chips shown in Figs. S25–S30. **(d)** Angle-scanned reflectance curves of Au+BP-COOH measured in Mix, Mix + 10^{-16} M Pb^{2+} and Mix + 10^{-15} M Pb^{2+}. **(e)** Net resonance-angle shift Δθ as a function of Pb^{2+} concentration in the mixed-ion solution. The statistical data are from Fig. S31. **(f)** Angle-scanned reflectance curves of Au+BP-COOH measured in drinking water, drinking water + 10^{-17} M Pb^{2+}, drinking water + 10^{-16} M Pb^{2+} and drinking water + 10^{-15} M Pb^{2+}. **(g)** Net resonance-angle shift Δθ as a function of Pb^{2+} concentration in drinking water. The statistical data are from Fig. S32."

Manuscript, lines 278–293:

"The selectivity of Au+BP-COOH was further evaluated by comparing its responses to other divalent metal ions. As shown in Fig. 5b,c, the response to Pb^{2+} appears in a substantially lower concentration regime than those of Cd^{2+}, Cu^{2+}, Zn^{2+}, Ni^{2+}, Ca^{2+} and Mg^{2+} under identical measurement conditions. The corresponding single-ion sensing results for Cd^{2+}, Cu^{2+}, Zn^{2+}, Ni^{2+}, Ca^{2+} and Mg^{2+} are provided in Figs. S25–S30. The LODs for the other metal ions are 10^{-13} M for Cd^{2+}, 10^{-13} M for Cu^{2+}, 10^{-12} M for Zn^{2+}, 10^{-10} M for Ni^{2+}, 10^{-9} M for Ca^{2+} and 10^{-8} M for Mg^{2+}. These results show that Au+BP-COOH exhibits a stronger response to Pb^{2+} than to the other tested divalent ions within the present sensing system.

The preferential response toward Pb^{2+} can be attributed to the stronger interfacial interaction between Pb^{2+} and oxygen-containing sites in BP-COOH. Pb^{2+} is a highly polarizable borderline-soft divalent ion and can form stable inner-sphere coordination with carboxylate and other oxygen-containing sites. In contrast, Ca^{2+} and Mg^{2+} are hard divalent ions with strong hydration, and their interaction with surface carboxylate sites is more strongly affected by hydration-shell retention. Transition-metal ions such as Ni^{2+}, Zn^{2+}, Cu^{2+} and Cd^{2+} can also interact with oxygen-containing groups, but their response occurs in higher concentration ranges under the same sensing conditions. This interpretation is consistent with previous reports on preferential Pb^{2+} adsorption at activated oxygen-containing sites on two-dimensional surfaces [31] and with the HSAB-based selectivity discussion used in two-dimensional-material ion sensing [22]."

[31] Peng, Q. et al. Unique lead adsorption behavior of activated hydroxyl group in two-dimensional titanium carbide. Journal of the American Chemical Society 136, 4113–4116 (2014).

[22] Liu, L. et al. Grain-boundary-rich polycrystalline monolayer WS_2 film for attomolar-level Hg^{2+} sensors. Nature Communications 12, 3870 (2021).

Manuscript, lines 294–300:

"Mixed-ion interference and real-sample measurements were further performed to evaluate the sensing response under more complex matrix conditions. In the mixed-ion experiment, Mix was used as the matrix background, and the net Pb^{2+} response was obtained by subtracting the Mix baseline from the Mix + Pb^{2+} response. As shown in Fig. 5d,e and Fig. S31, Pb^{2+} remains distinguishable in the mixed-ion solution at 10^{-16} M. In the real-sample experiment, drinking water was used as the matrix background, and the net Pb^{2+} response was obtained by subtracting the drinking-water baseline from the drinking water + Pb^{2+} response. As shown in Fig. 5f,g and Fig. S32, Pb^{2+} remains distinguishable in drinking water at 10^{-17} M. The water-quality background of the drinking-water sample is provided in Table S1."

Manuscript, lines 426–430:

"For the mixed-ion interference experiment, a mixed-ion solution was used as the matrix background. The Mix solution contained Cd^{2+}, Cu^{2+}, Zn^{2+}, Ni^{2+}, Ca^{2+} and Mg^{2+} at 10^{-13}, 10^{-13}, 10^{-12}, 10^{-10}, 10^{-9} and 10^{-8} M, respectively. The Mix solution was first measured as the matrix baseline. Then, Pb^{2+} was added into the Mix solution to obtain Mix + 10^{-16} M Pb^{2+} and Mix + 10^{-15} M Pb^{2+}. The net Pb^{2+} response was calculated by subtracting the Mix baseline from the Mix + Pb^{2+} response."

Supporting Information, lines 114–141:

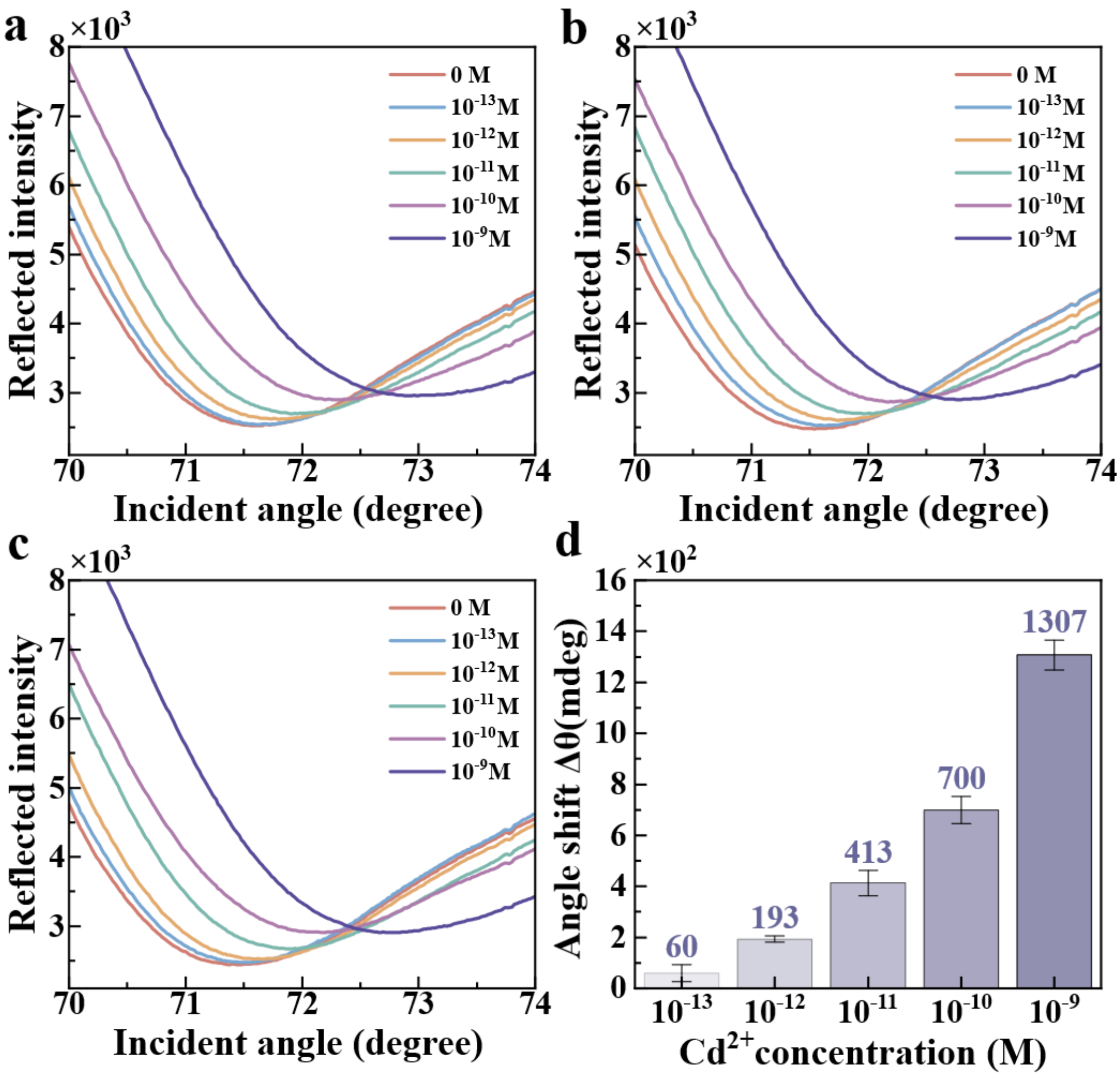

"**Fig. S25 Cd^{2+} sensing results obtained from Au+BP-COOH. (a–c)** Angle-scanned reflectance curves of three independently prepared Au+BP-COOH chips measured for Cd^{2+} concentrations of 0, 10^{-13}, 10^{-12}, 10^{-11}, 10^{-10} and 10^{-9} M. **(d)** Resonance-angle shift Δθ as a function of Cd^{2+} concentration for Au+BP-COOH."

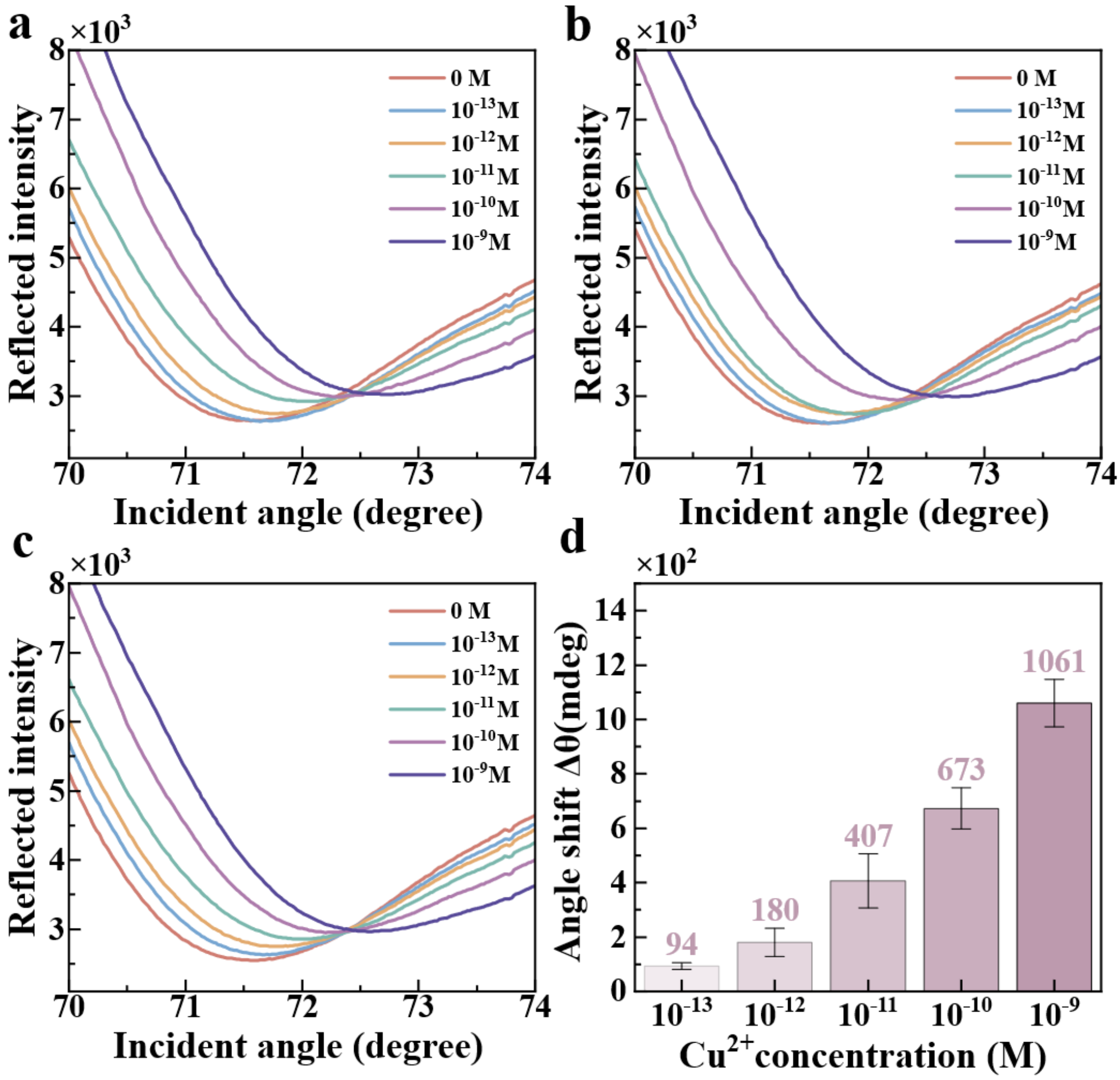

"Fig. S26 Cu^{2+} sensing results obtained from Au+BP-COOH. (a–c) Angle-scanned reflectance curves of three independently prepared Au+BP-COOH chips measured for Cu^{2+} concentrations of 0, 10^{-13}, 10^{-12}, 10^{-11}, 10^{-10} and 10^{-9} M. **(d)** Resonance-angle shift Δθ as a function of Cu^{2+} concentration for Au+BP-COOH."

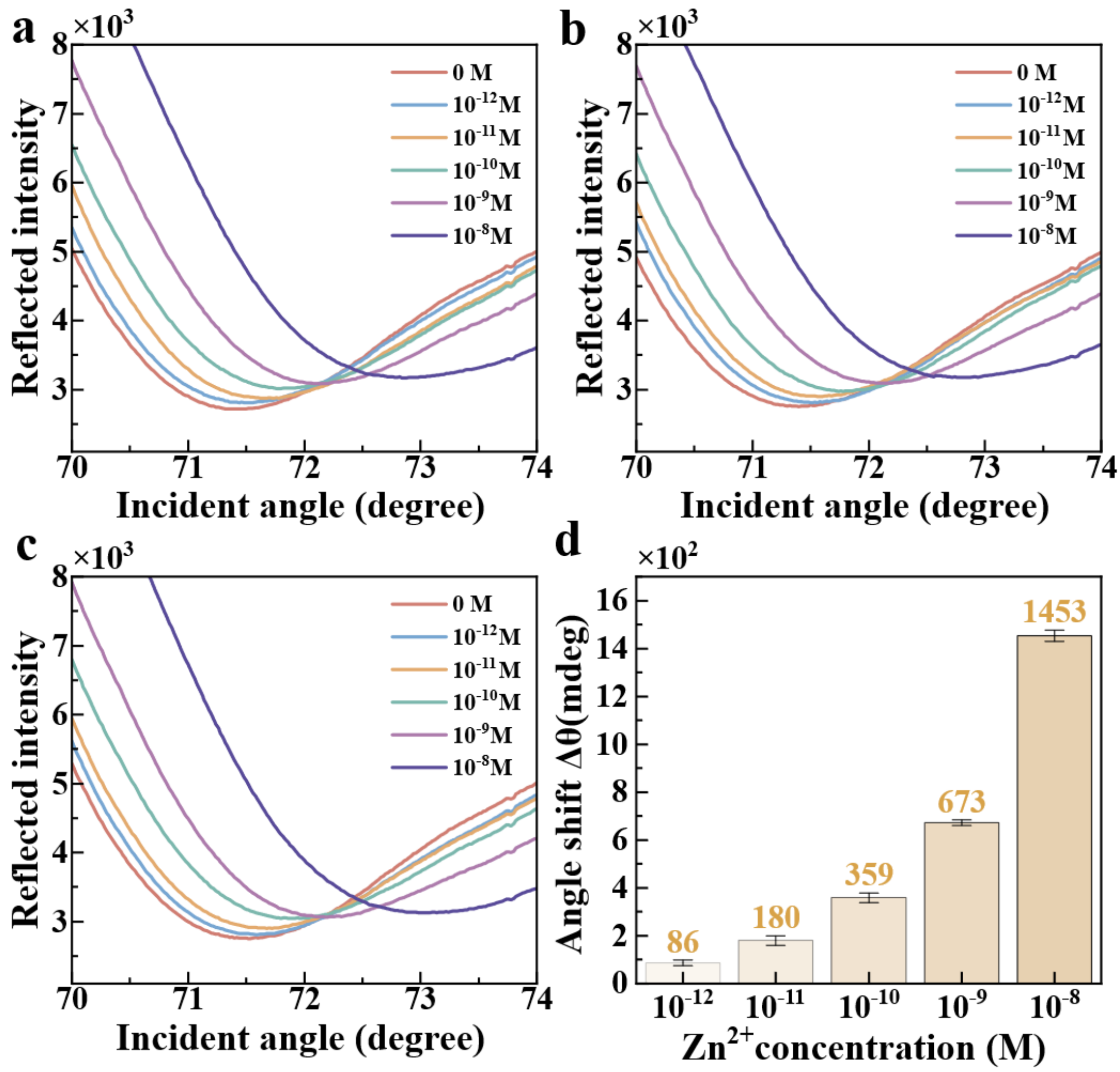

"Fig. S27 Zn^{2+} sensing results obtained from Au+BP-COOH. (a–c) Angle-scanned reflectance curves of three independently prepared Au+BP-COOH chips measured for Zn^{2+} concentrations of 0, 10^{-12}, 10^{-11}, 10^{-10}, 10^{-9} and 10^{-8} M. **(d)** Resonance-angle shift Δθ as a function of Zn^{2+} concentration for Au+BP-COOH."

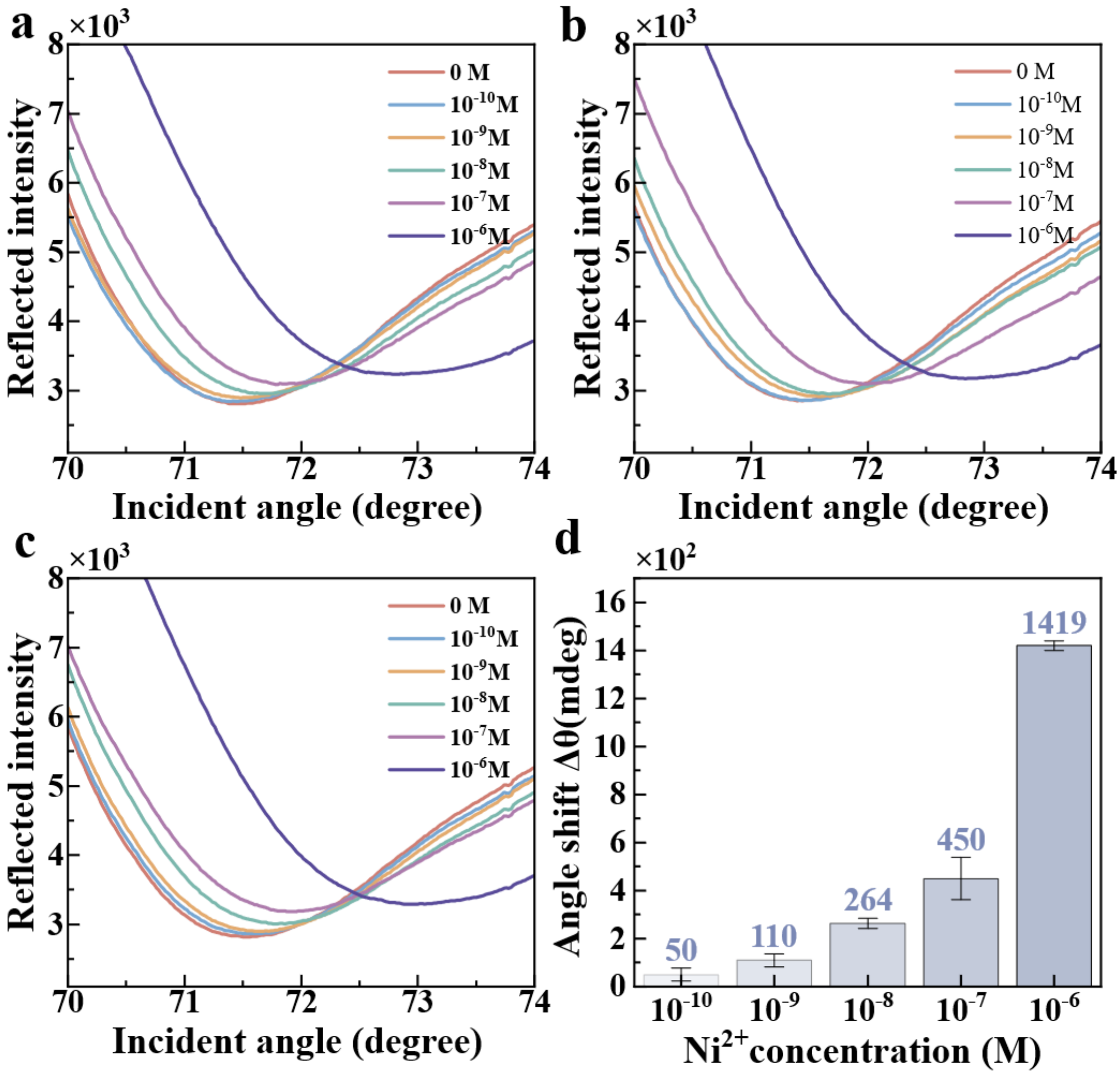

“Fig. S28 Ni^{2+} sensing results obtained from Au+BP-COOH. (a–c) Angle-scanned reflectance curves of three independently prepared Au+BP-COOH chips measured for Ni^{2+} concentrations of 0, 10^{-10}, 10^{-9}, 10^{-8}, 10^{-7} and 10^{-6} M. **(d)** Resonance-angle shift Δθ as a function of Ni^{2+} concentration for Au+BP-COOH.”

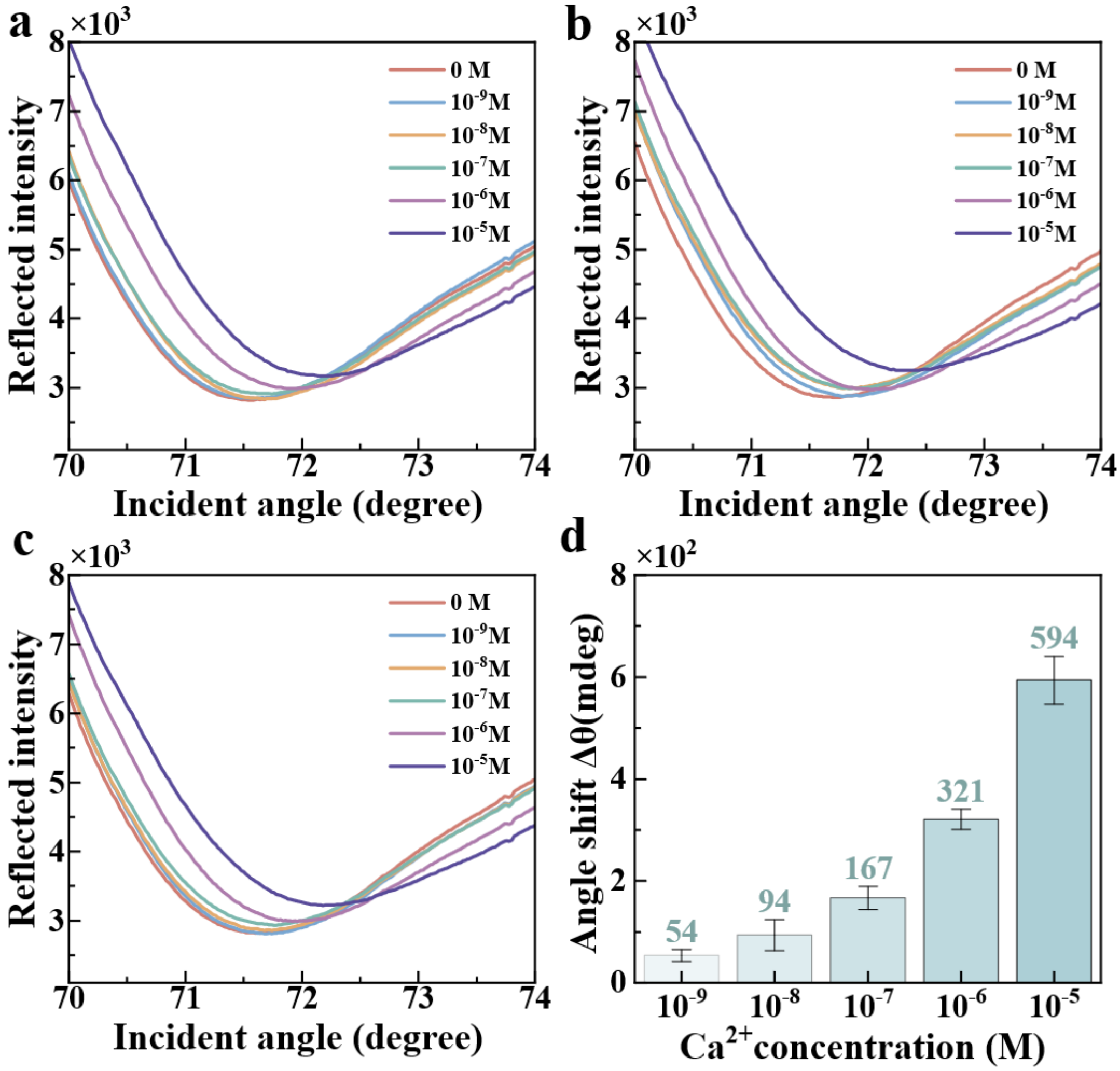

“**Fig. S29 Ca^{2+} sensing results obtained from Au+BP-COOH. (a–c)** Angle-scanned reflectance curves of three independently prepared Au+BP-COOH chips measured for Ca^{2+} concentrations of 0, 10^{-9}, 10^{-8}, 10^{-7}, 10^{-6} and 10^{-5} M. **(d)** Resonance-angle shift Δθ as a function of Ca^{2+} concentration for Au+BP-COOH.”

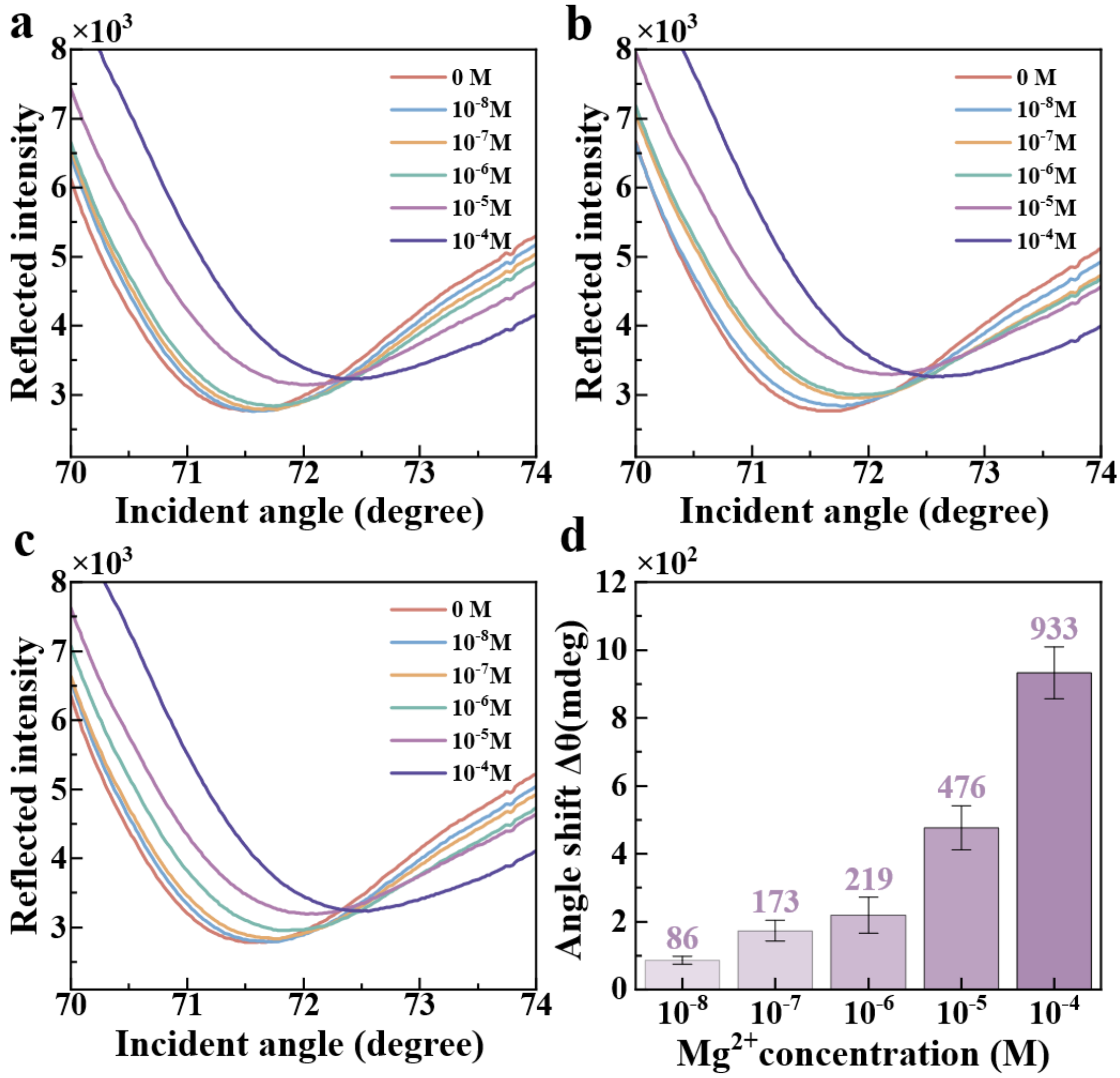

"Fig. S30 Mg^{2+} sensing results obtained from Au+BP-COOH. (a–c) Angle-scanned reflectance curves of three independently prepared Au+BP-COOH chips measured for Mg^{2+} concentrations of 0, 10^{-8}, 10^{-7}, 10^{-6}, 10^{-5} and 10^{-4} M. **(d)** Resonance-angle shift Δθ as a function of Mg^{2+} concentration for Au+BP-COOH."

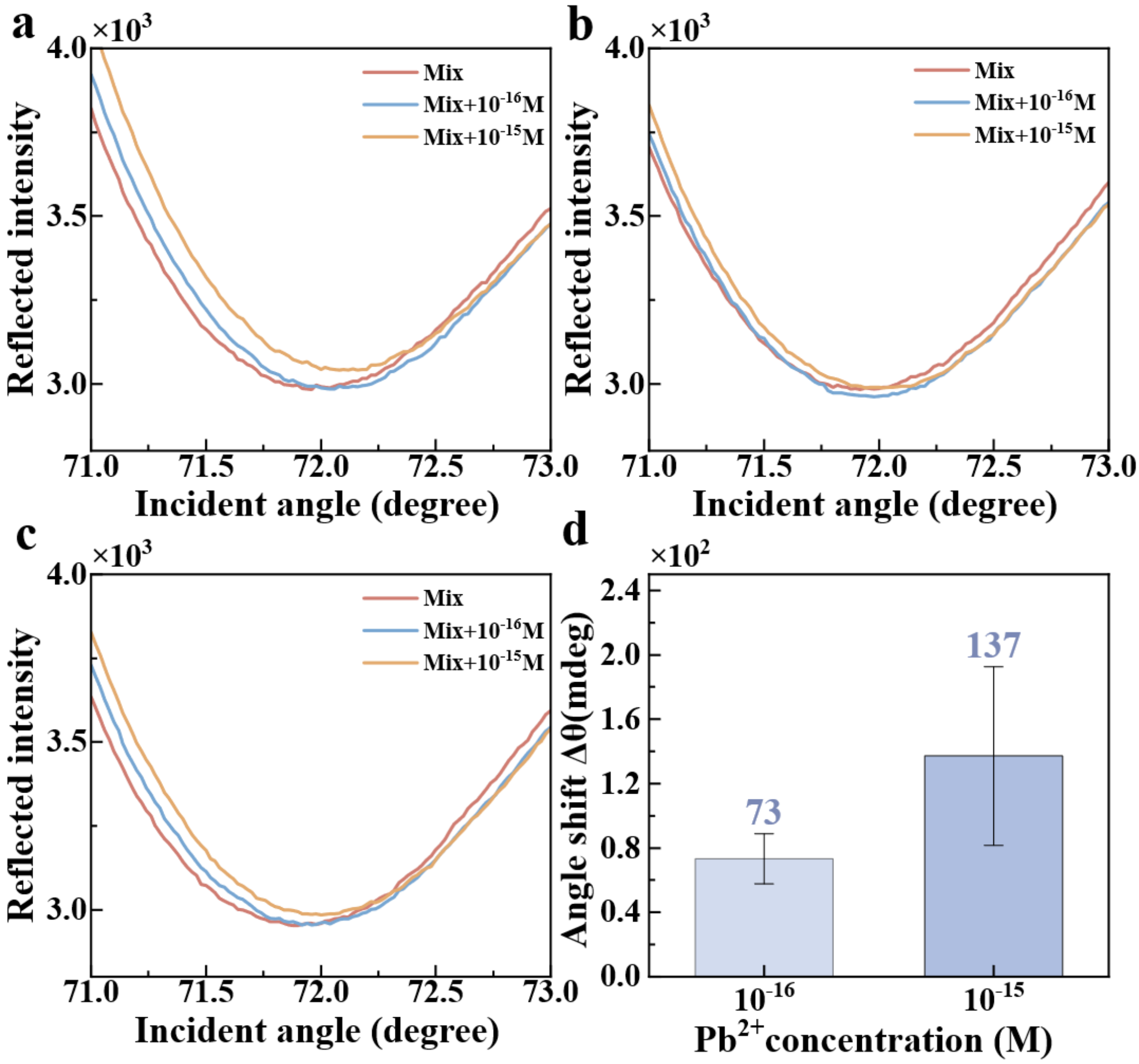

"**Fig. S31 Mixed-ion interference sensing results obtained from Au+BP-COOH. (a–c)** Angle-scanned reflectance curves of three independently prepared Au+BP-COOH chips measured in Mix, Mix + 10^{-16} M Pb^{2+} and Mix + 10^{-15} M Pb^{2+}. Mix represents the mixed-ion solution containing Cd^{2+}, Cu^{2+}, Zn^{2+}, Ni^{2+}, Ca^{2+} and Mg^{2+}. **(d)** Net resonance-angle shift Δθ as a function of Pb^{2+} concentration in the mixed-ion solution. Error bars are from three independently prepared Au+BP-COOH chips."

Comment 3

The oxidative degradation of BP under ambient and aqueous conditions is a well-recognized challenge. Although the authors suggest that PEI/PAA functionalization improves BP stability, no time-dependent stability data are provided. The authors should therefore include systematic stability tests, such as the sensing response of SPR+BP-COOH chips after storage for days to weeks under relevant conditions, together with XPS or Raman characterization to evaluate possible progressive P oxidation.

Response:

We sincerely thank the reviewer for raising this important and necessary concern. We fully understand that the stability of BP is a critical issue for evaluating the reliability and practical applicability of the Au+BP-COOH sensing interface, because oxidative degradation of BP under ambient and aqueous conditions is widely recognized. This comment helped us strengthen the

manuscript by adding time-dependent stability evidence at both the material-characterization level and the chip-sensing level.

In the revised manuscript, we added Raman characterization of BP-COOH from Day 1 to Day 7 to evaluate the structural retention of the BP-COOH functional material during storage. As shown in Fig. S1, the Raman spectra recorded over this period retain the BP-COOH Raman features, although slight intensity variations are observed. This result indicates that the BP-related Raman response is generally preserved during the tested storage period.

We also added chip-level storage-stability measurements using 10^{-15} M Pb^{2+} as a representative concentration. Day 1 corresponds to freshly prepared Au+BP-COOH chips measured immediately after fabrication, and Days 2–7 correspond to Au+BP-COOH chips measured after 1–6 days of storage. At each storage time point, pure water was first measured as the baseline, followed by 10^{-15} M Pb^{2+}. The sensing results obtained at different storage time points are provided in Figs. S18–S23, and the statistical results from Day 1 to Day 7 are summarized in Fig. S24. The Pb^{2+} response remains distinguishable at all tested storage time points, although the resonance-angle shift gradually decreases with storage time.

We appreciate that the reviewer suggested systematic stability tests over an extended storage period. In this revision, we evaluated the stability of BP-COOH and Au+BP-COOH over the Day 1–Day 7 timescale, which covers the preparation, storage and sensing period examined in the present study. The Raman spectra of BP-COOH from Day 1 to Day 7 provide time-dependent structural information, and the Pb^{2+} sensing statistics of Au+BP-COOH from Day 1 to Day 7 provide chip-level sensing evidence. Together, these results support the structural retention of BP-COOH and the distinguishable Pb^{2+} sensing response of Au+BP-COOH under the tested storage and measurement conditions.

Changes made in the manuscript:

Manuscript, lines 170–174:

"The structural retention of BP-COOH during storage was further examined by Raman spectroscopy. As shown in Fig. S1, Raman spectra recorded from Day 1 to Day 7 retain the Raman features of BP-COOH during this period. Although slight intensity variations are observed, the BP-related Raman response is generally preserved, indicating that the BP-COOH functional material maintains its main structural characteristics during short-term storage."

Manuscript, lines 301–307:

"The regeneration behavior and storage stability of Au+BP-COOH were also evaluated. The regeneration experiment was performed using 10^{-18} M Pb^{2+} on the Au+BP-COOH chip shown in Fig. S15. As shown in Fig. S17, the Pb^{2+} response remains distinguishable after five regeneration cycles. The storage stability was evaluated using 10^{-15} M Pb^{2+} as a representative concentration. The sensing results obtained at different storage time points are provided in Figs. S18–S23, and the corresponding statistical results from Day 1 to Day 7 are summarized in Fig. S24. The Pb^{2+} response remains distinguishable at all tested storage time points, although the resonance-angle shift gradually decreases with storage time."

Manuscript, lines 436–443:

"The storage stability of Au+BP-COOH chips was evaluated using 10^{-15} M Pb^{2+} as a representative concentration. Day 1 corresponds to freshly prepared Au+BP-COOH chips measured immediately after fabrication. Days 2–7 correspond to Au+BP-COOH chips measured after 1, 2, 3, 4, 5 and 6

days of storage, respectively. For Day 1, the statistical response was obtained from ten independently prepared Au+BP-COOH chips measured at 120 s. For Days 2–7, three independently prepared Au+BP-COOH chips were measured at each storage time point. At each time point, pure water was first measured as the baseline, followed by 10^{-15} M Pb^{2+}. The storage-stability response was defined as the net resonance-angle shift after subtracting the pure-water baseline."

Supporting Information, lines 21–22:

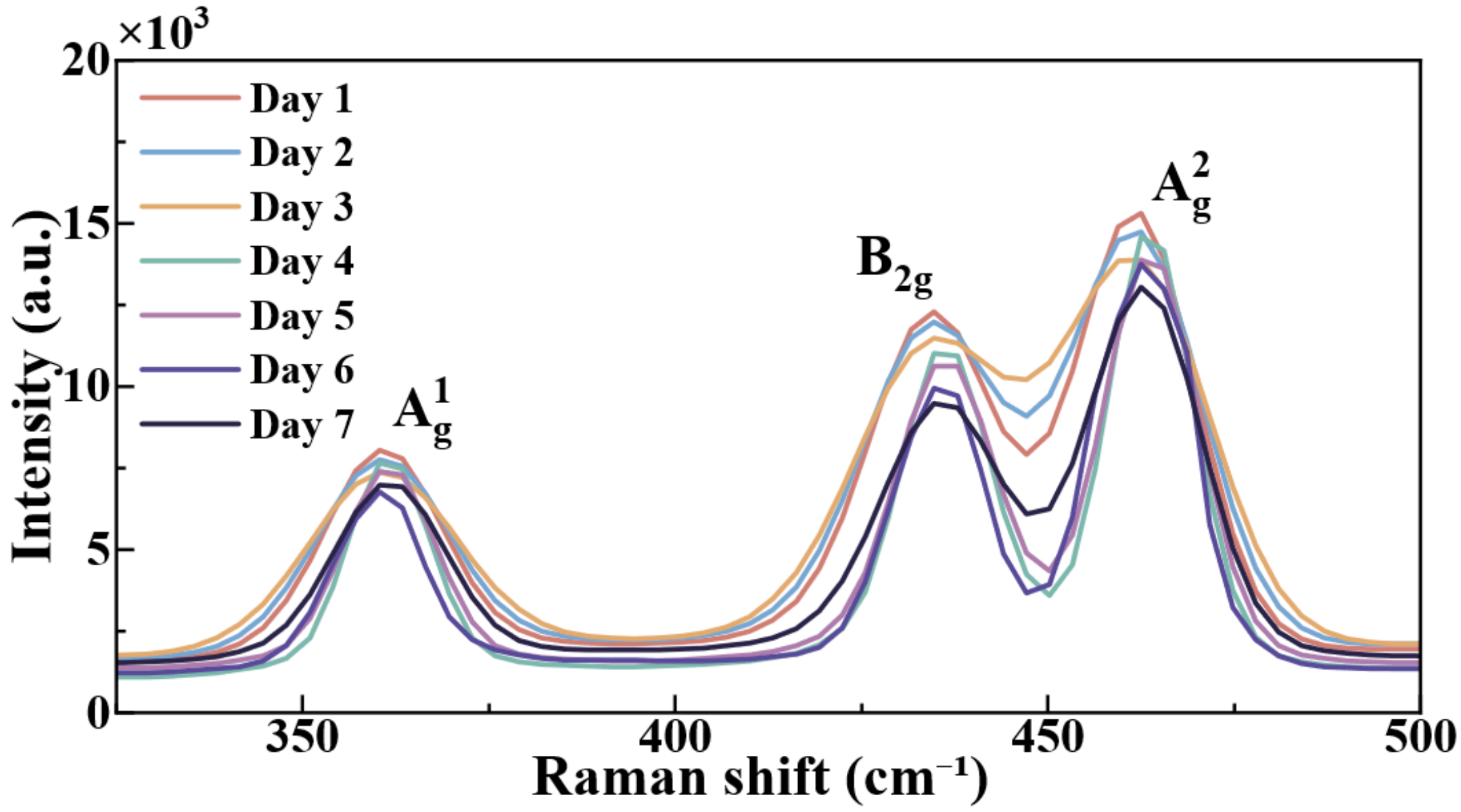

"Fig. S1 Raman spectra of BP-COOH during storage from Day 1 to Day 7. Raman spectra of BP-COOH recorded after storage for 1, 2, 3, 4, 5, 6 and 7 days."

Supporting Information, lines 90–107:

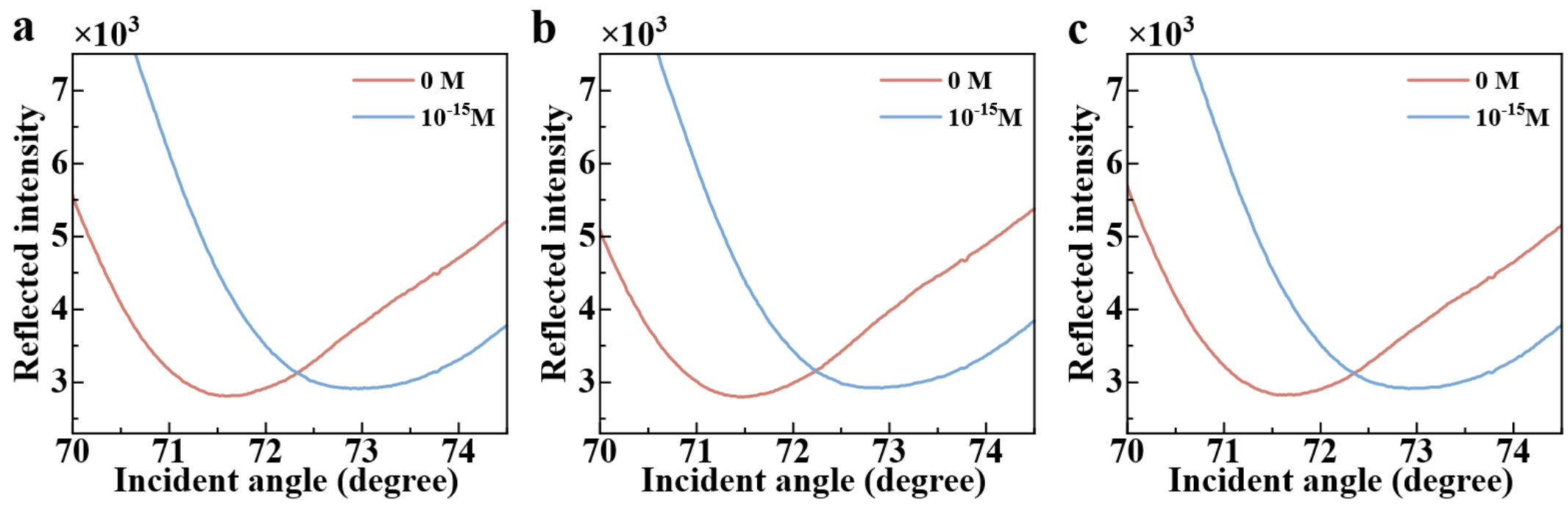

"**Fig. S18 Pb^{2+} sensing results obtained from Au+BP-COOH chips at storage time point Day 2. (a–c)** Angle-scanned reflectance curves of three independently prepared Au+BP-COOH chips measured for 0 M and 10^{-15} M Pb^{2+}. Day 2 corresponds to chips measured after 1 day of storage."

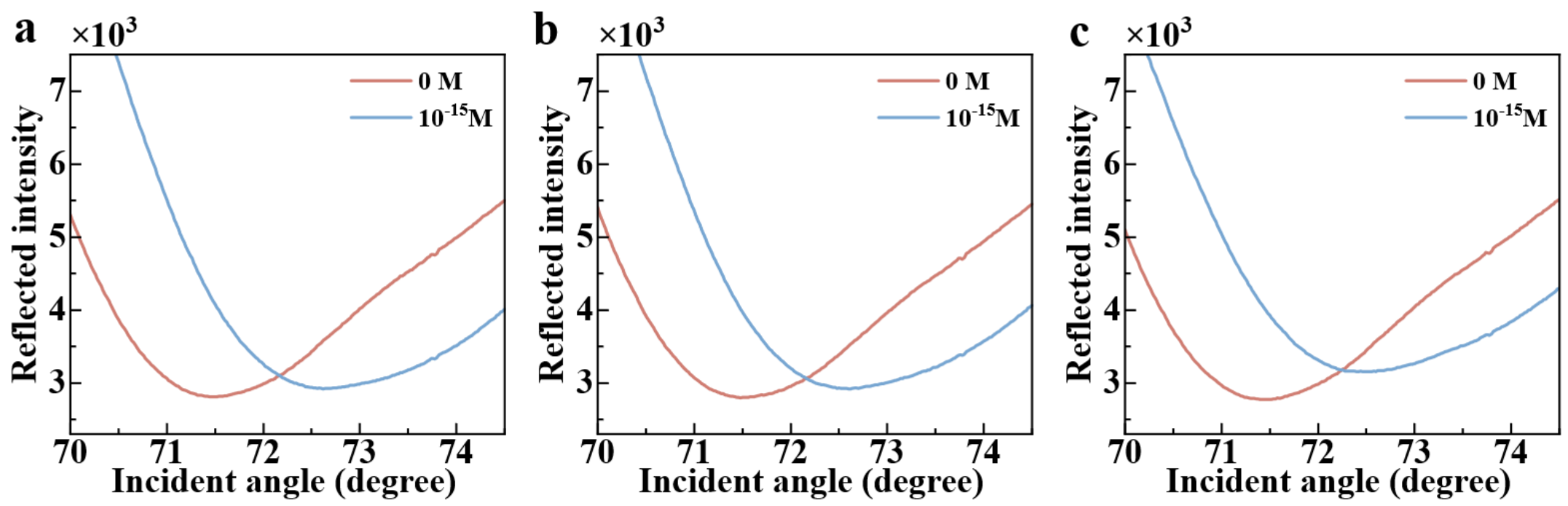

"Fig. S19 Pb^{2+} sensing results obtained from Au+BP-COOH chips at storage time point Day 3. (a–c) Angle-scanned reflectance curves of three independently prepared Au+BP-COOH chips measured for 0 M and 10^{-15} M Pb^{2+}. Day 3 corresponds to chips measured after 2 days of storage."

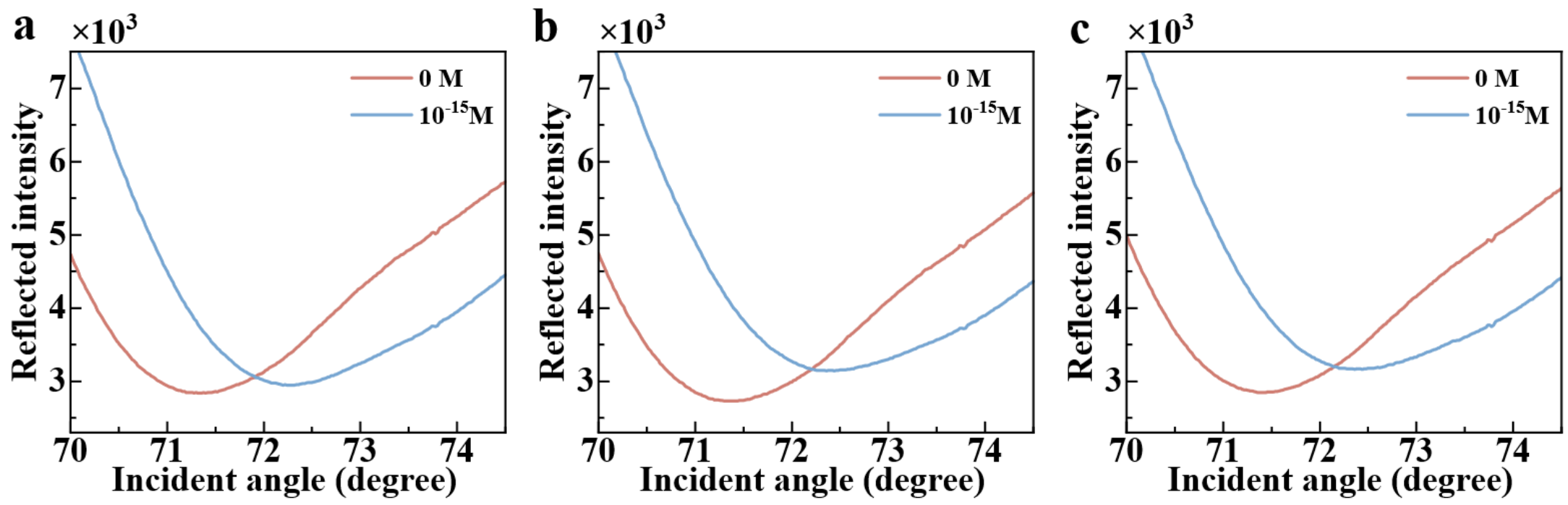

"Fig. S20 Pb^{2+} sensing results obtained from Au+BP-COOH chips at storage time point Day 4. (a–c) Angle-scanned reflectance curves of three independently prepared Au+BP-COOH chips measured for 0 M and 10^{-15} M Pb^{2+}. Day 4 corresponds to chips measured after 3 days of storage."

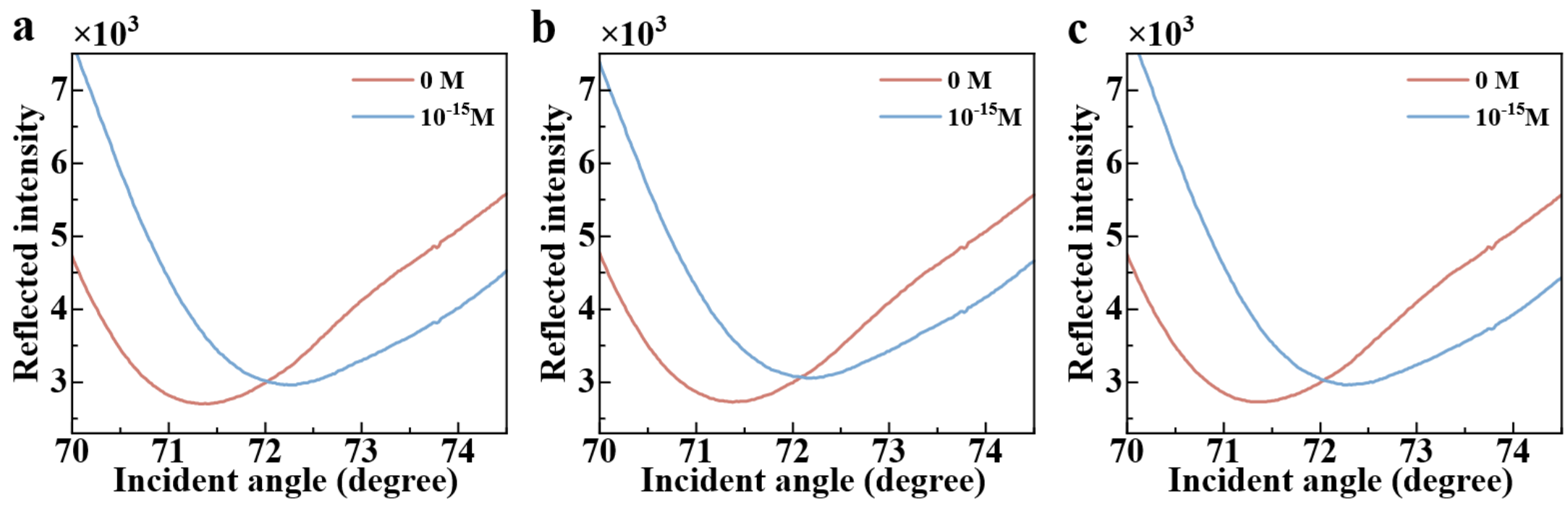

"Fig. S21 Pb^{2+} sensing results obtained from Au+BP-COOH chips at storage time point Day 5. (a–c) Angle-scanned reflectance curves of three independently prepared Au+BP-COOH chips measured for 0 M and 10^{-15} M Pb^{2+}. Day 5 corresponds to chips measured after 4 days of storage."

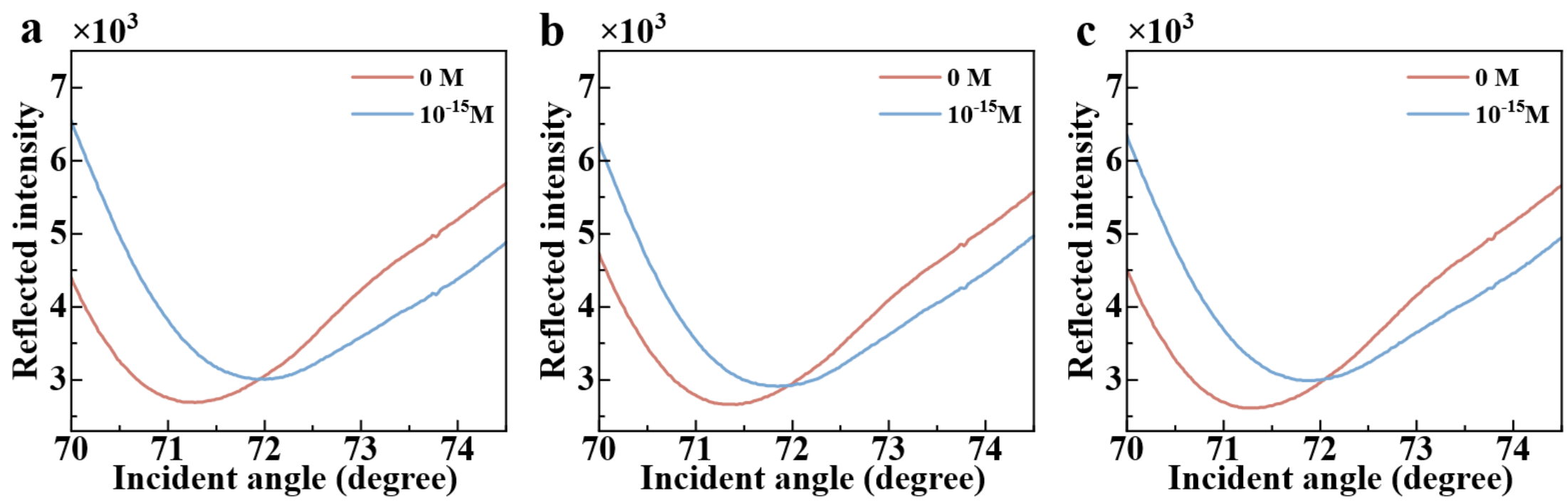

"Fig. S22 Pb^{2+} sensing results obtained from Au+BP-COOH chips at storage time point Day 6. (a–c) Angle-scanned reflectance curves of three independently prepared Au+BP-COOH chips measured for 0 M and 10^{-15} M Pb^{2+}. Day 6 corresponds to chips measured after 5 days of storage."

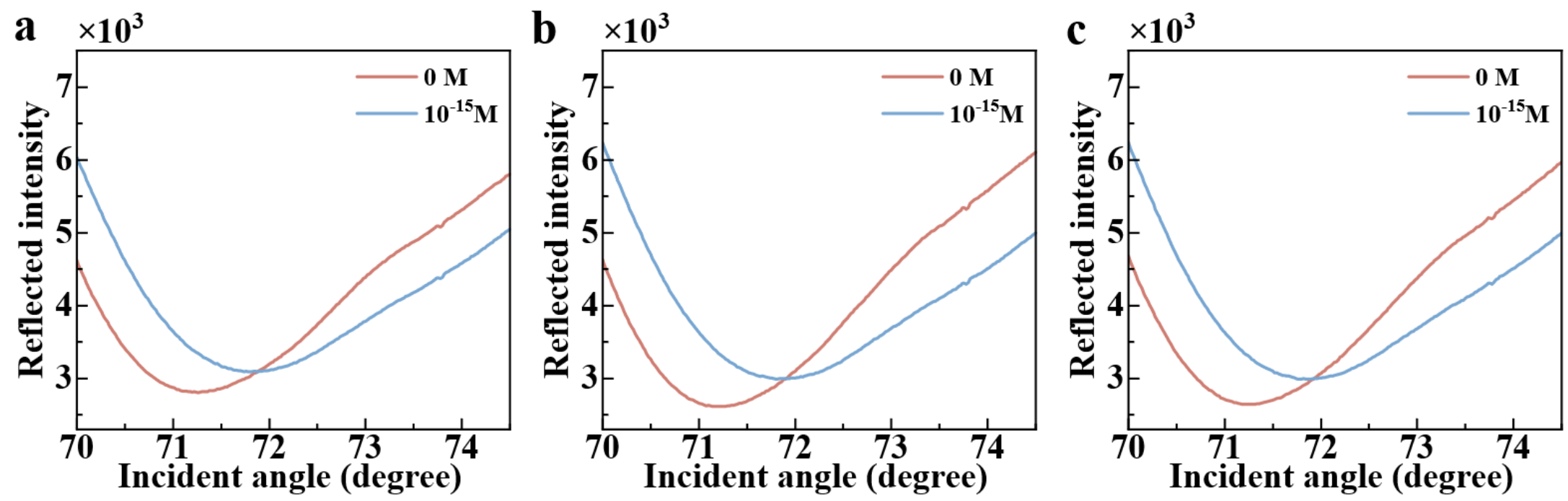

"Fig. S23 Pb^{2+} sensing results obtained from Au+BP-COOH chips at storage time point Day 7. (a–c) Angle-scanned reflectance curves of three independently prepared Au+BP-COOH chips measured for 0 M and 10^{-15} M Pb^{2+}. Day 7 corresponds to chips measured after 6 days of storage."

Supporting Information, lines 108–111:

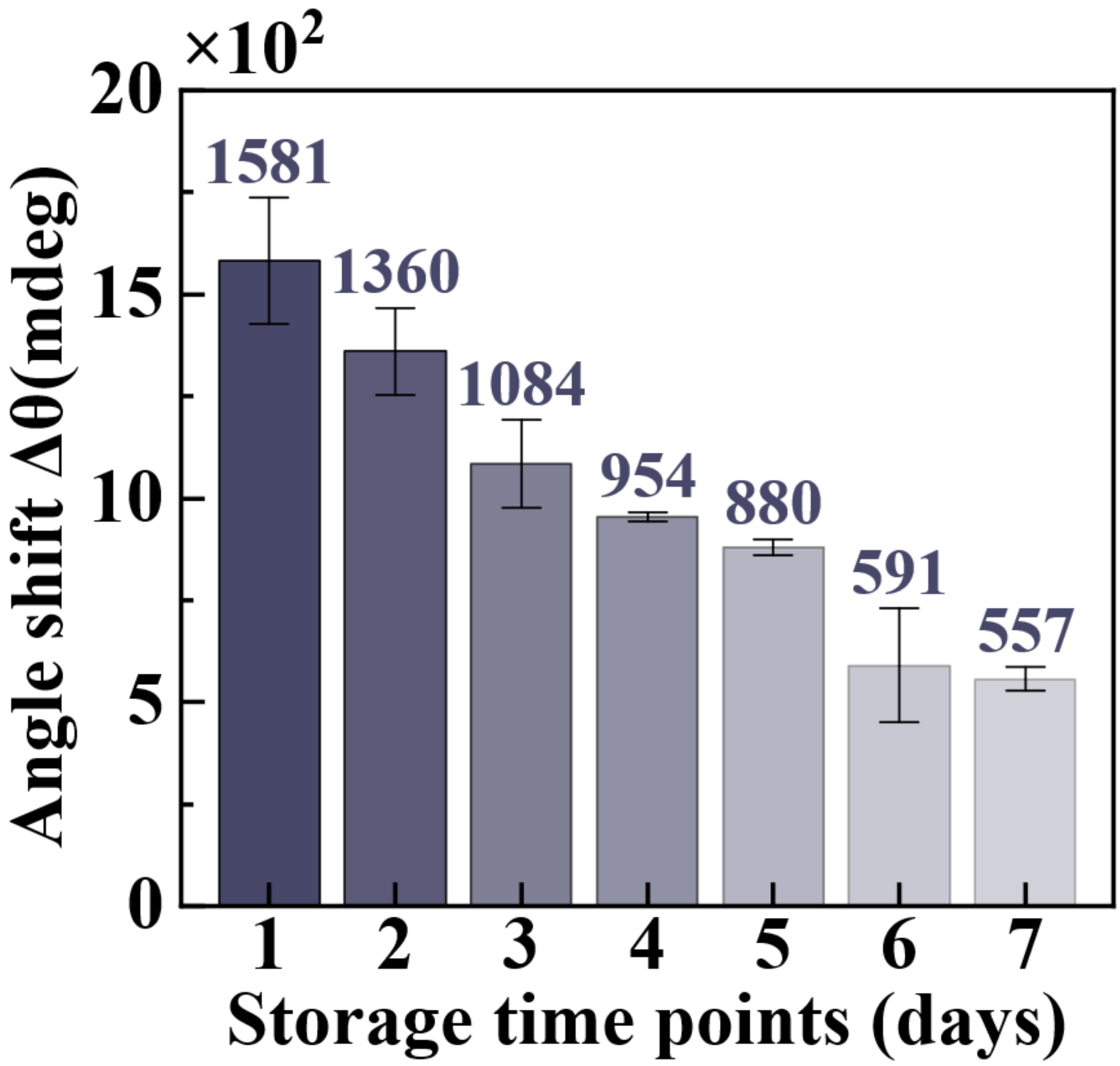

"**Fig. S24 Storage-stability statistics of Au+BP-COOH chips measured with 10^{-15} M Pb^{2+} at different storage time points.** Resonance-angle shift Δθ of Au+BP-COOH chips measured with 10^{-15} M Pb^{2+} from Day 1 to Day 7. Day 1 corresponds to freshly prepared chips measured immediately after fabrication, and Days 2–7 correspond to chips measured after 1, 2, 3, 4, 5 and 6 days of storage, respectively. Error bars are from independently prepared Au+BP-COOH chips."

Comment 4

All sensing experiments were conducted in pure water, which is insufficient to support the claim of "ultratrace heavy-metal monitoring in environmental and food-safety applications." The authors should validate the platform in representative real matrices using spiking-and-recovery tests and report the recovery rates.

Response:

We sincerely appreciate the reviewer's important suggestion. We fully understand that pure-water measurements cannot fully represent practical sensing conditions, because real samples contain background ions and matrix components that may influence the SPR baseline and the measured angular response. This comment helped us extend the validation of Au+BP-COOH from pure water to a representative real-sample matrix.

In the revised manuscript, we added Pb^{2+} spiking measurements in drinking water, which was selected as a representative aqueous real matrix closely related to Pb^{2+} exposure. The water-quality

background of the drinking-water sample was characterized and is provided in Table S1. During the real-sample measurement, drinking water was first measured as the matrix baseline. Pb^{2+} was then added into drinking water to obtain drinking water + 10^{-17} M Pb^{2+}, drinking water + 10^{-16} M Pb^{2+} and drinking water + 10^{-15} M Pb^{2+}. The net Pb^{2+} response was calculated by subtracting the drinking-water baseline from the drinking water + Pb^{2+} response.

As shown in Fig. 5f,g and Fig. S32, Au+BP-COOH shows a distinguishable Pb^{2+} response in drinking water at 10^{-17} M. The experiment was performed using three independently prepared Au+BP-COOH chips, and the error bars are shown in Fig. S32. Because the present SPR sensor directly reports Pb^{2+} response through resonance-angle shift and the LOD is defined by the distinguishability of the matrix-subtracted $\Delta\theta$ signal, the real-sample validation is presented as net $\Delta\theta$ after matrix-baseline correction. These added drinking-water spiking results support the applicability of Au+BP-COOH for ultralow Pb^{2+} detection in a more complex aqueous matrix.

Changes made in the manuscript:

Manuscript, lines 242–252:

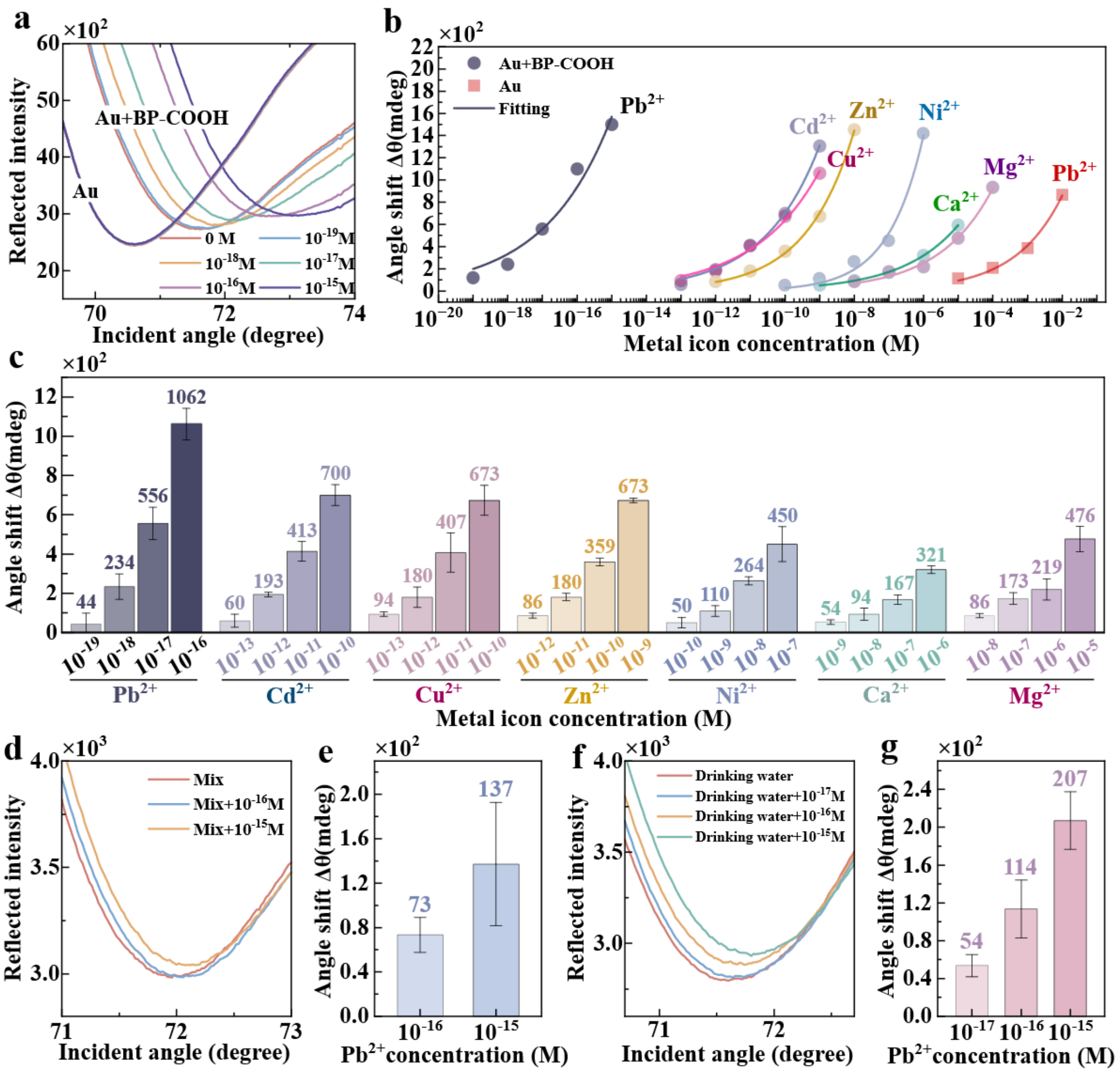

"Fig. 5. Pb^{2+} sensing response of Au+BP-COOH and comparison with other metal ions in pure water, mixed-ion solution, and drinking water. (a) Angle-scanned reflectance curves of Au+BP-COOH measured for Pb^{2+} concentrations of 0, 10^{-19}, 10^{-18}, 10^{-17}, 10^{-16} and 10^{-15} M, together with

the reflectance curve of bare Au. **(b)** Resonance-angle shift Δθ as a function of metal-ion concentration for Au+BP-COOH and bare Au. The data points are mean values from the corresponding statistical results in c and Fig. S4, and the solid lines represent nonlinear fitting curves. **(c)** Resonance-angle shift Δθ values of Au+BP-COOH measured at different concentrations of Pb^{2+}, Cd^{2+}, Cu^{2+}, Zn^{2+}, Ni^{2+}, Ca^{2+} and Mg^{2+}. The Pb^{2+} data are from the ten independently prepared Au+BP-COOH chips shown in Figs. S6–S15 and statistically summarized in Fig. S16, and the data for Cd^{2+}, Cu^{2+}, Zn^{2+}, Ni^{2+}, Ca^{2+} and Mg^{2+} are from the three independently prepared Au+BP-COOH chips shown in Figs. S25–S30. **(d)** Angle-scanned reflectance curves of Au+BP-COOH measured in Mix, Mix + 10^{-16} M Pb^{2+} and Mix + 10^{-15} M Pb^{2+}. **(e)** Net resonance-angle shift Δθ as a function of Pb^{2+} concentration in the mixed-ion solution. The statistical data are from Fig. S31. **(f)** Angle-scanned reflectance curves of Au+BP-COOH measured in drinking water, drinking water + 10^{-17} M Pb^{2+}, drinking water + 10^{-16} M Pb^{2+} and drinking water + 10^{-15} M Pb^{2+}. **(g)** Net resonance-angle shift Δθ as a function of Pb^{2+} concentration in drinking water. The statistical data are from Fig. S32."

Manuscript, lines 294–300:

"Mixed-ion interference and real-sample measurements were further performed to evaluate the sensing response under more complex matrix conditions. In the mixed-ion experiment, Mix was used as the matrix background, and the net Pb^{2+} response was obtained by subtracting the Mix baseline from the Mix + Pb^{2+} response. As shown in Fig. 5d,e and Fig. S31, Pb^{2+} remains distinguishable in the mixed-ion solution at 10^{-16} M. In the real-sample experiment, drinking water was used as the matrix background, and the net Pb^{2+} response was obtained by subtracting the drinking-water baseline from the drinking water + Pb^{2+} response. As shown in Fig. 5f,g and Fig. S32, Pb^{2+} remains distinguishable in drinking water at 10^{-17} M. The water-quality background of the drinking-water sample is provided in Table S1."

Manuscript, lines 308–315:

"Taken together, Fig. 5, Table 1 and the supporting results in Figs. S4–S32 establish the Pb^{2+} sensing performance and selectivity of Au+BP-COOH from five aspects. First, Au+BP-COOH enables ultralow-concentration Pb^{2+} sensing in pure water. Second, the comparison among bare Au, Au+BP and Au+BP-COOH distinguishes the BP-related optical contribution from the BP-COOH-related Pb^{2+} interaction contribution. Third, the time-dependent statistics of ten independently prepared Au+BP-COOH chips clarify the detection behavior at ultralow Pb^{2+} concentrations. Fourth, the detectable concentration regime for Pb^{2+} remains markedly lower than those of Cd^{2+}, Cu^{2+}, Zn^{2+}, Ni^{2+}, Ca^{2+} and Mg^{2+}. Fifth, Pb^{2+} can be detected in mixed-ion solution and drinking water after subtracting the corresponding matrix baselines, and Au+BP-COOH retains sensing responses in regeneration and storage-stability tests."

Manuscript, lines 431–435:

"Drinking water was used as the real-sample matrix for Pb^{2+} sensing. The water-quality background of the drinking-water sample is provided in Table S1. During measurement, drinking water was first measured as the matrix baseline. Then, Pb^{2+} was added into drinking water to obtain drinking water + 10^{-17} M Pb^{2+}, drinking water + 10^{-16} M Pb^{2+} and drinking water + 10^{-15} M Pb^{2+}. The net Pb^{2+} response was calculated by subtracting the drinking-water baseline from the drinking water + Pb^{2+} response."

Supporting Information, lines 143–146:

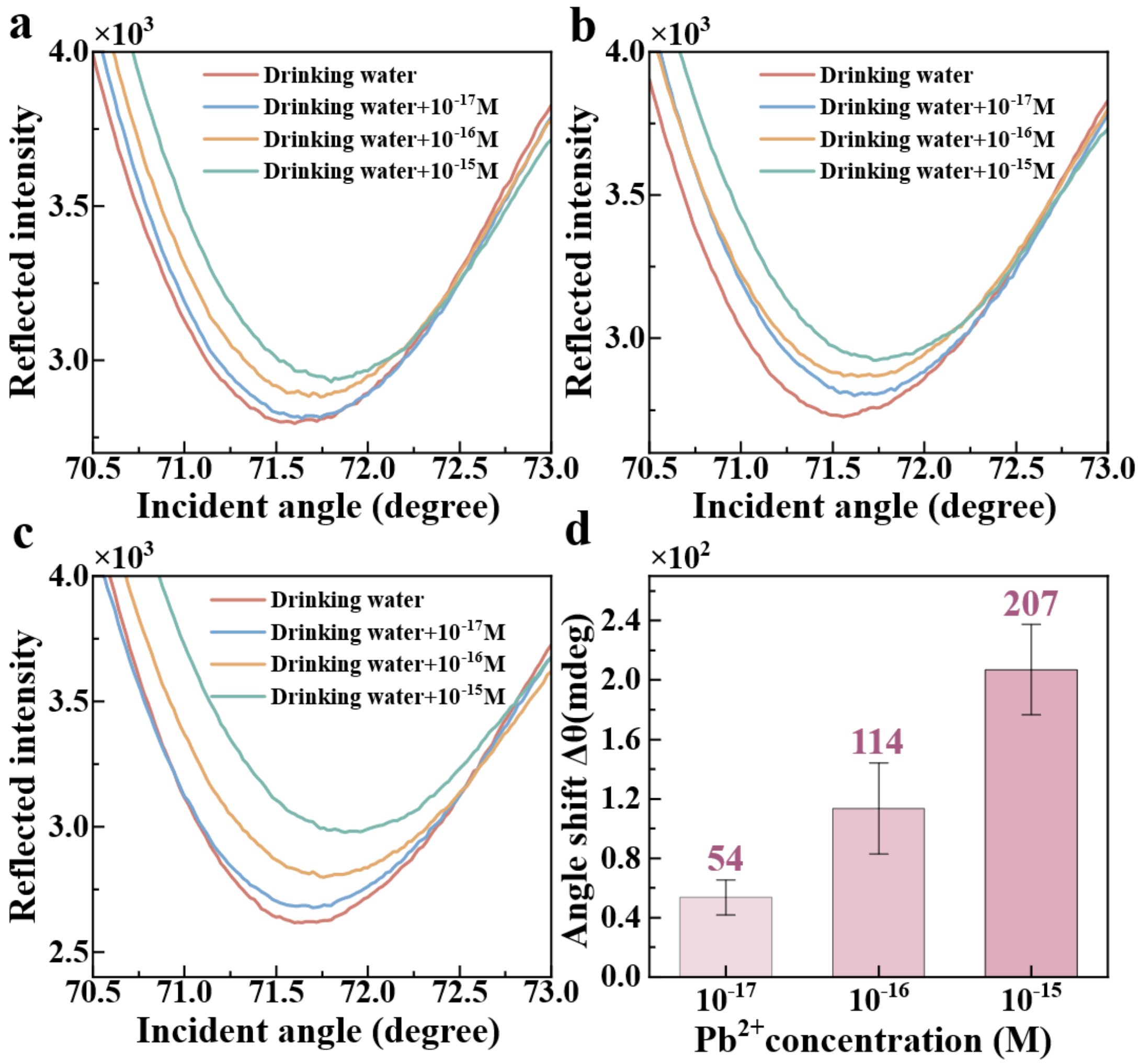

"**Fig. S32 Pb^{2+} sensing results obtained from Au+BP-COOH in drinking water. (a–c)** Angle-scanned reflectance curves of three independently prepared Au+BP-COOH chips measured in drinking water, drinking water + 10^{-17} M Pb^{2+}, drinking water + 10^{-16} M Pb^{2+} and drinking water + 10^{-15} M Pb^{2+}. **(d)** Net resonance-angle shift Δθ as a function of Pb^{2+} concentration in drinking water. Error bars are from three independently prepared Au+BP-COOH chips."

Supporting Information, lines 154–157:

"**Table S1** Water-quality background of the drinking-water sample used for real-sample Pb^{2+} sensing.

Parameter	Unit	Sample	QC duplicate	Mean / reported value	Relative deviation	Acceptance criterion	QC result
pH	—	7.0	7.0	7.0	0.00	±0.1 pH unit	Pass
Conductivity	μS cm^{-1}	70.6	72.4	71.5	0.6%	≤10%	Pass
Total dissolved solids	mg L^{-1}	58	62	60	3.3%	≤10%	Pass
Na^+	mg L^{-1}	2.71	2.66	2.69	0.9%	≤10%	Pass
K^+	mg L^{-1}	2.90	2.84	2.87	1.0%	≤10%	Pass
Mg^{2+}	mg L^{-1}	1.85	1.80	1.83	1.4%	≤10%	Pass
Ca^{2+}	mg L^{-1}	10.1	9.8	10.0	1.5%	≤10%	Pass
Cl^-	mg L^{-1}	2.40	2.45	2.43	1.0%	≤10%	Pass
NO_3^-	mg L^{-1}	1.74	1.77	1.76	0.9%	≤10%	Pass
SO_4^{2-}	mg L^{-1}	4.61	4.55	4.58	0.7%	≤10%	Pass
CO_3^{2-}	mg L^{-1}	n.a.	n.a.	n.a.	—	—	—
HCO_3^-	mg L^{-1}	36.8	39.3	38.1	3.2%	≤10%	Pass
Hg	μg L^{-1}	0.000	0.000	n.d.	0.0%	≤20%	Pass
Cu	μg L^{-1}	0.213	0.231	0.22	4.1%	≤20%	Pass
Pb	μg L^{-1}	0.000	0.000	n.d.	0.0%	≤20%	Pass
Cd	μg L^{-1}	0.005	0.002	n.d.	0.0%	≤20%	Pass
Zn	μg L^{-1}	0.127	0.120	0.124	2.8%	≤20%	Pass
Ni	μg L^{-1}	0.012	0.012	0.012	0.0%	≤20%	Pass
Total Cr	μg L^{-1}	n.d.	n.d.	n.d.	—	≤10%	Pass

Values are from duplicate external measurements of the drinking-water sample. Relative deviations were provided by the testing laboratory. n.d., not detected; n.a., not applicable. For Cd and total Cr, trace instrumental signals were recorded, but the final reported results were below the reporting/detection threshold and are therefore listed as n.d."

Comment 5

The LOD is defined as the lowest concentration at which the reflectance curve is distinguishable beyond the instrumental drift range, which does not follow the standard IUPAC criterion of LOD = 3σ/k. The authors should justify why the conventional IUPAC definition was not adopted, explain the basis for using the current criterion instead, and consider whether this choice affects the scientific rigor and comparability of the reported detection limit.

Response:

We are very grateful to the reviewer for this rigorous and important comment. We fully understand that the definition of LOD is central to the credibility and comparability of the reported sensing performance. This comment helped us clarify the analytical basis of the LOD definition and make the distinguishability criterion more transparent in the revised manuscript.

In the present SPR measurements, the primary readout is the resonance-angle shift Δθ, which is obtained directly from the minimum reflectance point in the angle-scanned SPR curve. The resonance angle was directly read without curve fitting, and the angular scanning step of the instrument was 19–21 mdeg. Given that the Pb^{2+} response was evaluated over a broad concentration range and exhibited a nonlinear concentration dependence, the use of a single calibration slope k would make the calculated LOD highly dependent on the selected fitting range and fitting model. Therefore, we used an experimentally quantified angular distinguishability criterion for this direct SPR angular readout.

The basis of this criterion is now explicitly described in the revised manuscript. Repeated pure-

water measurements were performed on the same Au+BP-COOH chip at 2 min intervals over 50 min. As shown in Fig. S33, the maximum resonance-angle drift was 21 mdeg, and the standard deviation of the resonance angle was 0.00714 deg, corresponding to 7.14 mdeg. Therefore, 3σ is 21.42 mdeg, which is close to both the measured maximum drift and the 19–21 mdeg angular scanning step. Based on these experimental values, a response was considered distinguishable when $\Delta\theta$ exceeded 21 mdeg.

The LOD was then defined as the lowest concentration at which a distinguishable response was obtained under the corresponding sensing condition. We have also clarified that the same distinguishability threshold was applied consistently to the sensing comparisons in this work. To improve comparability with literature reports, we further stated in the Fig. 6 caption that all reported LOD values from representative studies were converted to molar concentration before plotting. In this way, the revised manuscript provides both the experimental basis for the LOD criterion and the information needed to interpret the reported LOD in comparison with previous sensing systems.

Changes made in the manuscript:

Manuscript, lines 398–401:

"SPR measurements were performed on a Dynechem SPR system using 650 nm incident light at 25 °C. The angular scanning range was 65°–80°, and the angular step was 19–21 mdeg. For each measurement, 400 μL of sample solution was introduced into the flow cell at a flow rate of 200 μL/min. The resonance angle was directly read from the angle corresponding to the minimum reflectance point without curve fitting."

Manuscript, lines 419–424:

"The resonance-angle shift $\Delta\theta$ was used to evaluate the sensing response. A response was considered distinguishable when $\Delta\theta$ exceeded 21 mdeg. This operational distinguishability threshold was selected according to the angular scanning step of the SPR measurement and the baseline stability of Au+BP-COOH in pure water. Repeated pure-water measurements on the same Au+BP-COOH chip over 50 min showed a maximum resonance-angle drift of 21 mdeg and a standard deviation of 0.00714 deg (Fig. S33). The LOD was defined as the lowest concentration at which a distinguishable response was obtained under the corresponding sensing condition."

Supporting Information, lines 158–160:

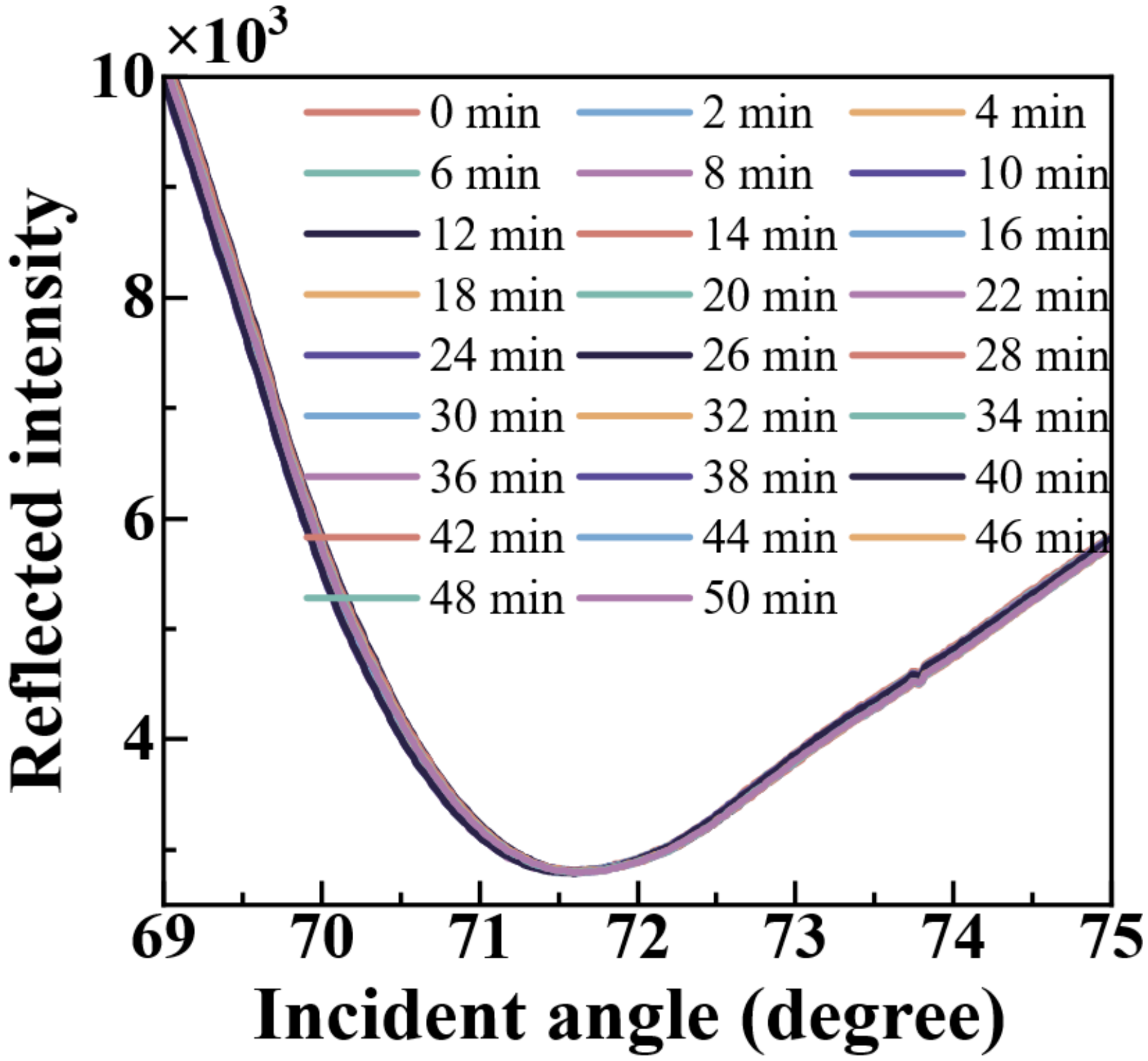

"**Fig. S33 Baseline stability of Au+BP-COOH in pure water.** Angle-scanned reflectance curves recorded on the same Au+BP-COOH chip in pure water at 2 min intervals over 50 min. The maximum resonance-angle drift during the 50 min measurement was 21 mdeg, and the standard deviation of the resonance angle was 0.00714 deg."

Comment 6

The optical simulation is oversimplified and does not match the experiment well. The optical simulation is based on a simplified transfer-matrix model of a bare Au/BP bilayer, in which the polymer-functionalized BP-COOH layer and its actual optical properties are not included, and the simulated optimal thickness is inconsistent with the experimental thickness, which needs explanation.

Response:

We are very grateful to the reviewer for this technically important comment. We fully understand that the reviewer is concerned about the relationship between the simplified optical simulation and the actual Au+BP-COOH sensing interface. The experimental interface contains polymer-functionalized BP-COOH nanosheets, while the transfer-matrix simulation uses an ideal Au/BP multilayer model. Therefore, the reviewer's concern helped us revise the manuscript so that the simulation, experimental calibration, and sensing controls are presented with clearer roles and a more consistent logic.

In the revised manuscript, the quantitative optical evaluation is based on experimental refractive-

index calibration rather than on the simplified simulation. We added a five-interface comparison including bare Au, Au+Graphene, Au+MoS_2, Au+BP and Au+BP-COOH (Fig. 3). Au+Graphene and Au+MoS_2 were included as reference two-dimensional overlayer structures because they also possess high-index confinement and interlayer-coupling-related optical characteristics. Therefore, this comparison evaluates whether the enhanced angular response is a general two-dimensional-overlayer effect or is more strongly associated with the BP-based interface. Under the same refractive-index calibration conditions, the angular sensitivities are 127 deg/RIU for bare Au, 157 deg/RIU for Au+Graphene, 168 deg/RIU for Au+MoS_2, 256 deg/RIU for Au+BP and 259 deg/RIU for Au+BP-COOH. This experimental comparison shows that BP-based interfaces provide a stronger angular SPR response than the other two-dimensional overlayers examined here.

This comparison also helps separate two different contributions. In the refractive-index calibration, Au+BP and Au+BP-COOH show very close angular sensitivities, 256 and 259 deg/RIU, respectively. This indicates that carboxyl functionalization preserves the BP-related optical response while introducing oxygen-containing interfacial sites. We then compared Pb^{2+} sensing on bare Au, Au+BP and Au+BP-COOH. Bare Au shows a distinguishable Pb^{2+} response from 10^{-5} M, Au+BP from 10^{-7} M, and Au+BP-COOH down to 10^{-19} M (Figs. S4, S5 and Fig. 5). Thus, the first comparison clarifies the BP-related optical contribution, while the second comparison distinguishes the BP overlayer contribution from the BP-COOH functional-layer contribution in Pb^{2+} sensing.

Regarding the apparent thickness inconsistency, the transfer-matrix simulation uses ideal continuous BP layers with model optical parameters, whereas the experimental Au+BP-COOH film is a polymer-functionalized BP nanosheet layer. Its measured AFM thickness reflects the actual nanosheet assembly, polymer functionalization, interfacial coverage and film morphology. Therefore, the simulated layer number and the experimentally measured film thickness represent different physical descriptions. In the revised manuscript, we clarified that the simulation provides a theoretical reference for the BP-overlayer-induced enhancement trend, while the experimental refractive-index calibration in Fig. 3 is used as the direct basis for evaluating the optical response of the actual sensing interface.

Overall, the revised logic is organized at two levels: Fig. 3 experimentally establishes the enhanced angular response of the actual Au+BP and Au+BP-COOH interfaces, and Figs. S4, S5 and Fig. 5 distinguish the BP-related optical contribution from the BP-COOH-related Pb^{2+} interaction contribution. Fig. S3 supports the same enhancement trend from the transfer-matrix optical model and provides the simulated field-distribution reference.

Changes made in the manuscript:

Manuscript, lines 181–185:

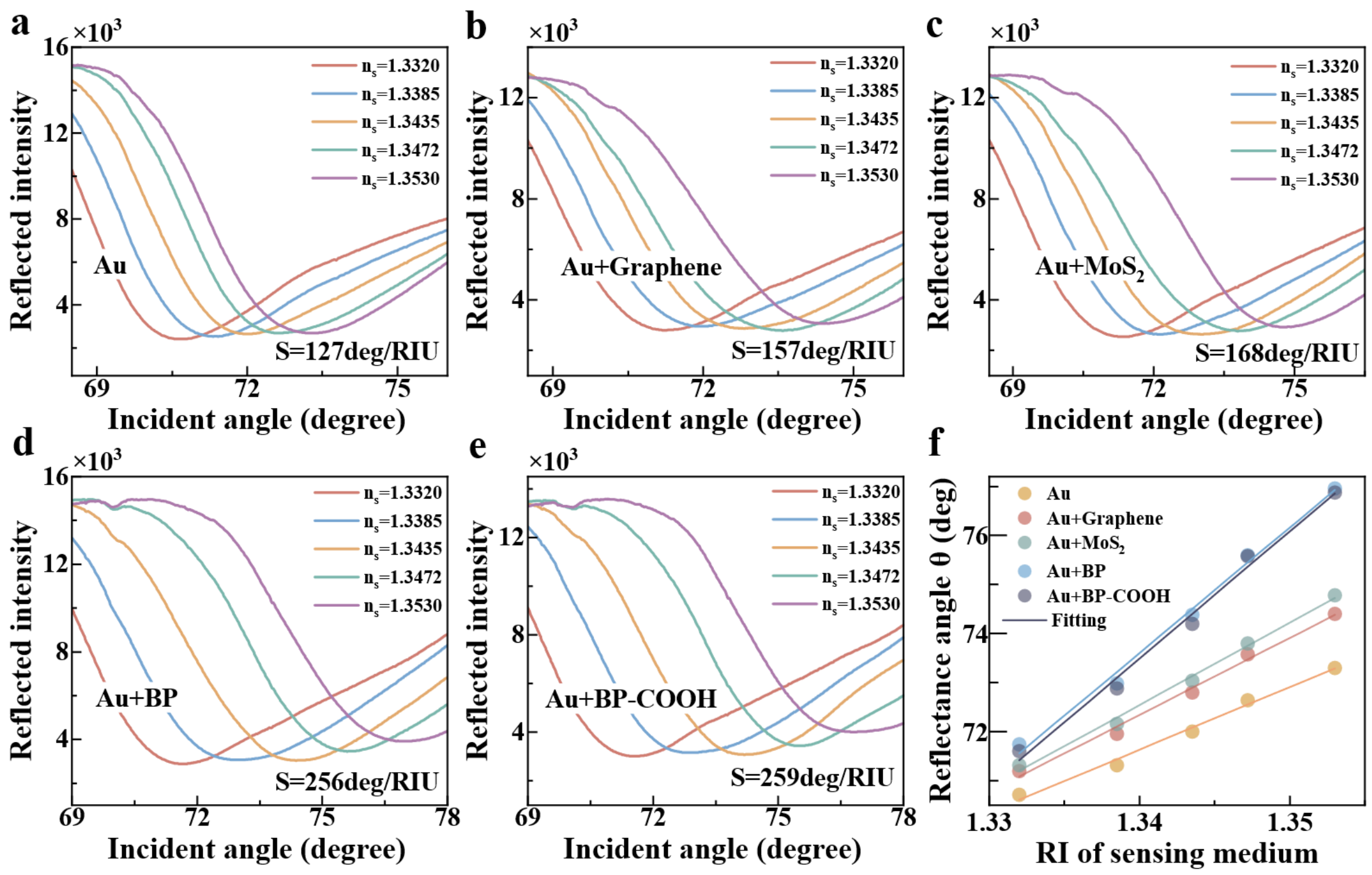

"**Fig. 3. Experimental refractive-index calibration of bare Au, Au+Graphene, Au+MoS_2, Au+BP and Au+BP-COOH. (a–e)** Angle-scanned reflectance curves of bare Au, Au+Graphene, Au+MoS_2, Au+BP and Au+BP-COOH measured in sensing media with refractive indices n_s = 1.3320, 1.3385, 1.3435, 1.3472 and 1.3530, respectively. **(f)** Resonance angle as a function of the refractive index of the sensing medium for bare Au, Au+Graphene, Au+MoS_2, Au+BP and Au+BP-COOH, together with linear fitting curves."

Manuscript, lines 193–215:

"**Optical basis and physical interpretation of sensitivity enhancement by the BP-based overlayer.** To clarify the optical basis for the improved sensing performance of Au+BP-COOH, the refractive-index-dependent angular SPR responses of bare Au, Au+Graphene, Au+MoS_2, Au+BP and Au+BP-COOH were experimentally compared under the same sensing framework. As shown in Fig. 3a–e, the angle-scanned reflectance curves of all five structures shift toward larger incident angles when the refractive index of the sensing medium increases from n_s = 1.3320 to 1.3530. However, the magnitude of the resonance-angle displacement is different for different interfacial structures.

The corresponding resonance angles are summarized in Fig. 3f. Linear fitting gives angular sensitivities of 127 deg/RIU for bare Au, 157 deg/RIU for Au+Graphene, 168 deg/RIU for Au+MoS_2, 256 deg/RIU for Au+BP and 259 deg/RIU for Au+BP-COOH. Compared with bare Au, the introduction of two-dimensional overlayers increases the angular response of the Au-film SPR structure to refractive-index variation. Au+Graphene and Au+MoS_2 were used as reference two-dimensional overlayer structures because they also possess high-index confinement and interlayer-coupling-related optical characteristics. However, their angular sensitivities remain lower than those of Au+BP and Au+BP-COOH under the same refractive-index calibration conditions. This comparison indicates that the BP-based overlayer provides a stronger optical response than the other two-dimensional overlayers examined here. In addition, the angular sensitivities of Au+BP and Au+BP-COOH are close to each other, indicating that carboxyl functionalization preserves the BP-related optical response while introducing oxygen-containing interfacial sites for subsequent Pb^{2+}

interaction.

The simulated angular SPR response and electric-field distribution of the Au-film structure with and without a BP overlayer are provided in Fig. S3. The simulation shows the same enhancement trend after introducing the BP overlayer and provides a theoretical reference for the BP-induced optical response. In the experimental Au+BP-COOH structure, the BP-COOH functional layer contains polymer-functionalized BP nanosheets, and therefore the experimental refractive-index calibration in Fig. 3 is used as the direct basis for evaluating the optical response of the actual sensing interface."

Manuscript, lines 216–237:

"The physical origin of this enhanced near-field response is schematically summarized in Fig. 4. As illustrated in Fig. 4a,b, the introduction of BP changes the interfacial optical environment of the Au-film SPR structure. BP possesses a relatively high refractive index over a broad spectral range, and its introduction onto the Au surface creates a stronger dielectric contrast at the plasmonic interface. Under this condition, the effective mode volume of the evanescent field is compressed, and the electromagnetic field becomes more strongly localized near the sensing interface. In addition, the multilayer structure of BP introduces interlayer coupling, which further enhances optical confinement and contributes to a stronger near-field response than that of the Au/sensing-medium structure.

A second contribution is illustrated in Fig. 4c,d. Because Au and BP have different work functions, contact between the two components can drive directional interfacial charge redistribution. This process leads to the formation of a built-in electric field at the Au/BP interface, which can further strengthen local electromagnetic coupling near the sensing region. In this sense, the role of BP in improving SPR sensitivity is not limited to adding an ultrathin overlayer, but also involves interfacial regulation of the local field environment. BP also exhibits a high carrier mobility, reported up to approximately 10^3 $cm^2/(V\cdot s)$ at room temperature [24], which is favorable for efficient interfacial charge response. Taken together, the high-index confinement effect, multilayer coupling effect, and interfacial charge-transfer effect provide a physically reasonable interpretation for the stronger optical response of Au+BP and Au+BP-COOH observed in Fig. 3.

Taken together, Fig. 3, Fig. 4 and Fig. S3 show that introducing the BP-based overlayer strengthens the interfacial optical response of the Au-film SPR structure to refractive-index variation. The experimental refractive-index calibration verifies the enhanced angular sensitivity of Au+BP and Au+BP-COOH, the simulation provides a theoretical reference for the BP-overlayer-induced optical response, and the schematic in Fig. 4 provides a physical interpretation of the enhanced near-field response. These results provide the optical basis for the subsequent improvement in Pb^{2+} sensing performance observed for Au+BP-COOH."

Manuscript, lines 259–264:

"To clarify the contribution of BP and BP-COOH to Pb^{2+} sensing, control measurements were performed on bare Au and Au+BP. The Pb^{2+} sensing results of bare Au and Au+BP are provided in Figs. S4 and S5, respectively. Bare Au shows a distinguishable Pb^{2+} response from 10^{-5} M, whereas Au+BP shows a distinguishable response from 10^{-7} M. This comparison indicates that the BP overlayer improves the Pb^{2+} sensing response relative to bare Au. By further introducing the BP-COOH functional layer, the detectable Pb^{2+} concentration is extended to 10^{-19} M, indicating that the oxygen-containing interfacial sites of BP-COOH play a key role in the ultralow-concentration Pb^{2+} response."

Supporting Information, lines 29–34:

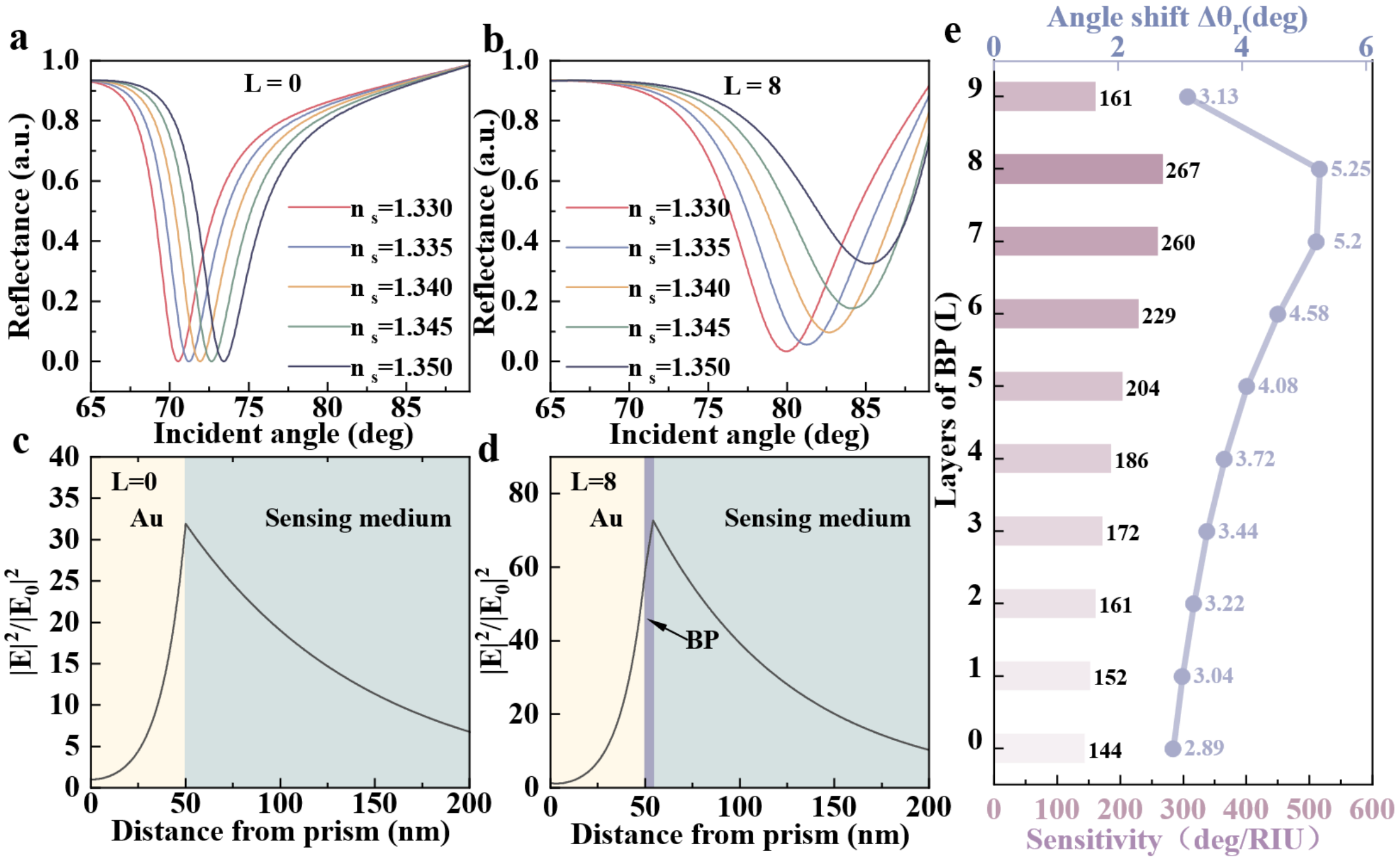

“Fig. S3 Simulated angular SPR response, electric-field distribution, and layer-number-dependent sensitivity of the Au-film SPR structure with and without a BP overlayer. (a) Simulated angle-scanned reflectance curves of bare Au with L = 0 at sensing-medium refractive indices n_s = 1.330, 1.335, 1.340, 1.345 and 1.350. **(b)** Simulated angle-scanned reflectance curves of the BP-modified Au structure with L = 8 at sensing-medium refractive indices n_s = 1.330, 1.335, 1.340, 1.345 and 1.350. **(c)** Normalized electric-field intensity distribution of bare Au with L = 0. **(d)** Normalized electric-field intensity distribution of the BP-modified Au structure with L = 8. **(e)** Simulated angular sensitivity and total resonance-angle shift as a function of the number of BP layers.”

Supporting Information, lines 36–40:

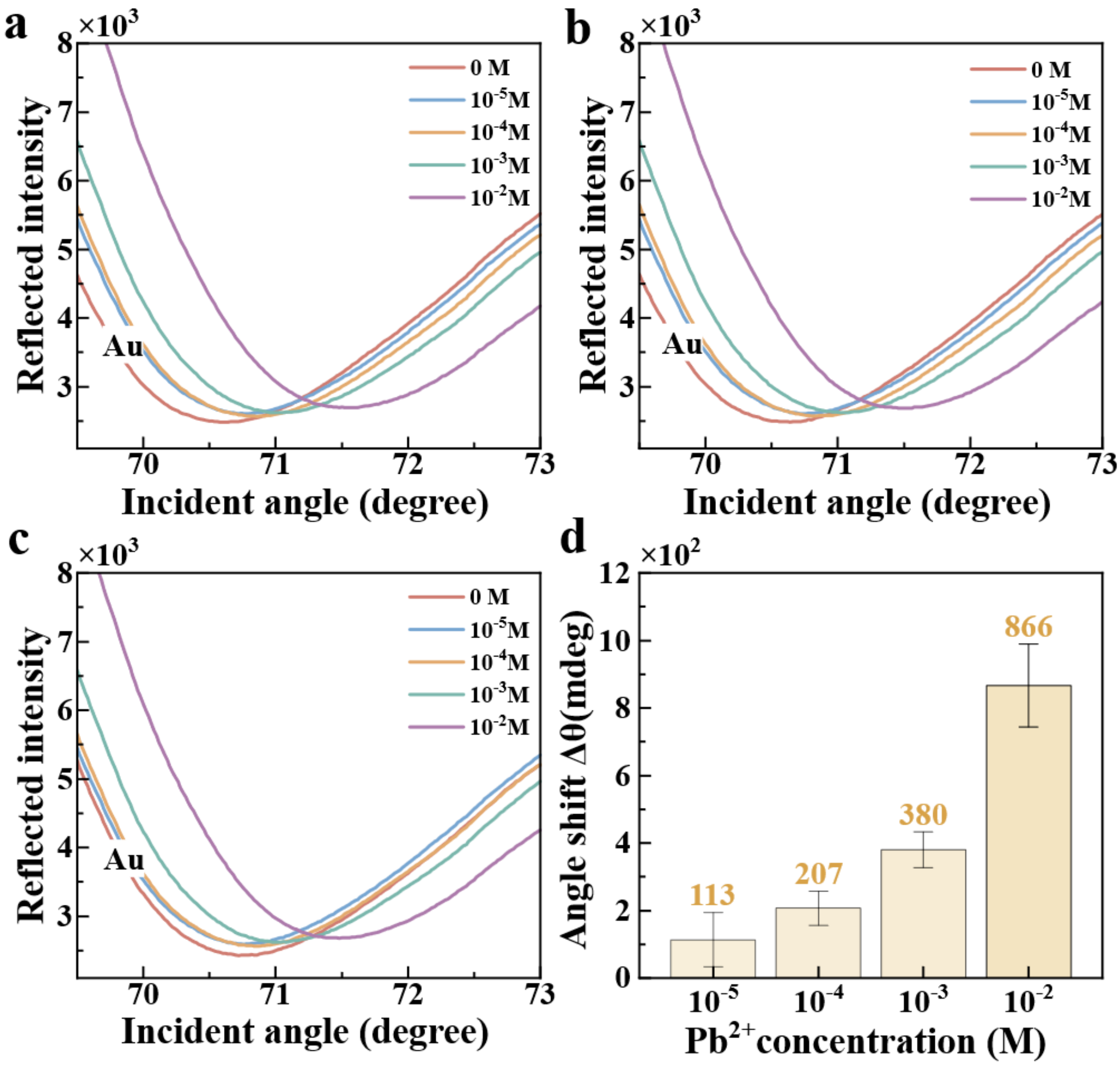

"**Fig. S4 Pb^{2+} sensing results obtained from bare Au. (a–c)** Angle-scanned reflectance curves of three independently prepared bare Au chips measured for Pb^{2+} concentrations of 0, 10^{-5}, 10^{-4}, 10^{-3} and 10^{-2} M. **(d)** Resonance-angle shift Δθ as a function of Pb^{2+} concentration for bare Au."

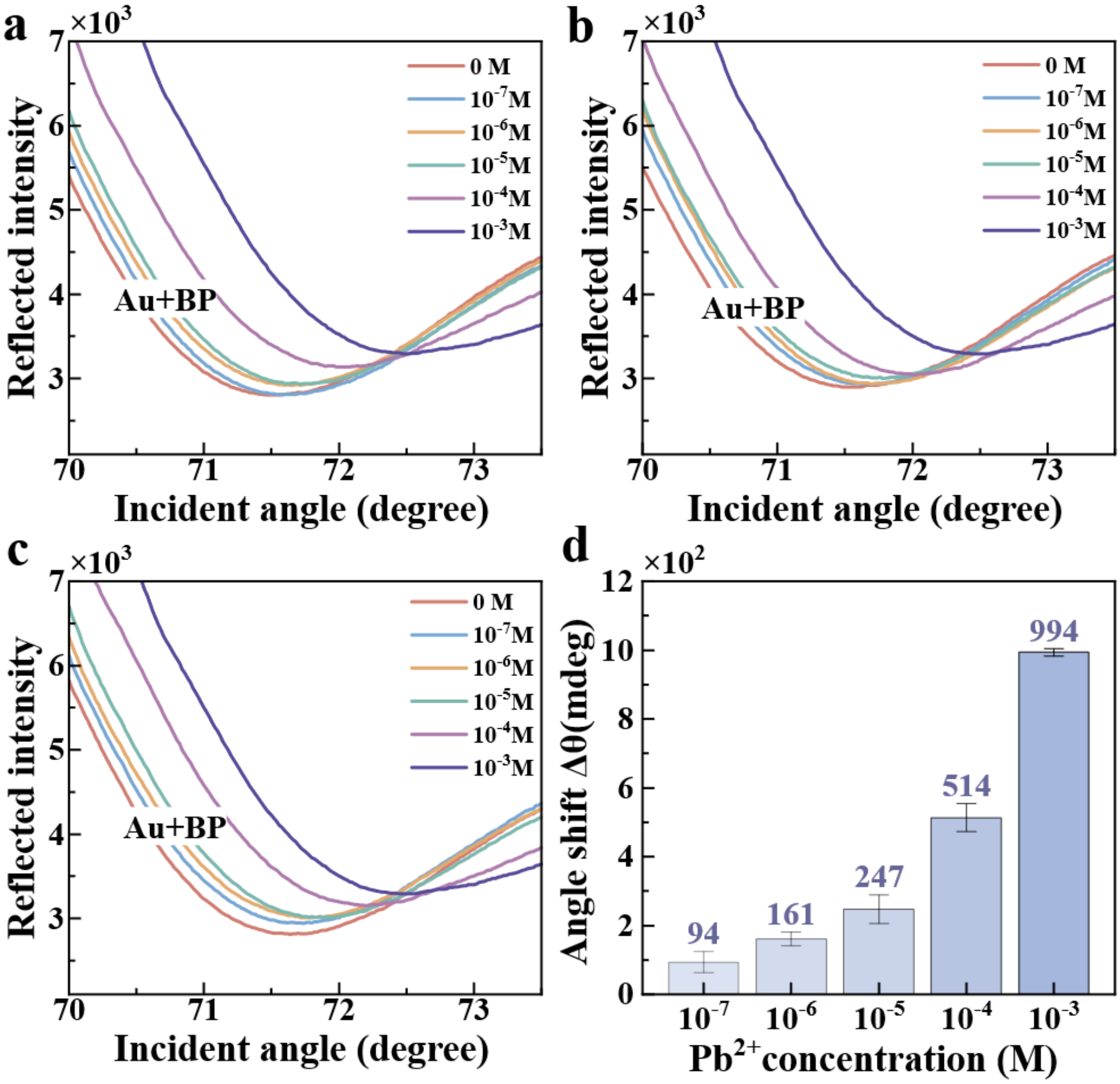

“**Fig. S5 Pb^{2+} sensing results obtained from Au+BP. (a–c)** Angle-scanned reflectance curves of three independently prepared Au+BP chips measured for Pb^{2+} concentrations of 0, 10^{-7}, 10^{-6}, 10^{-5}, 10^{-4} and 10^{-3} M. **(d)** Resonance-angle shift Δθ as a function of Pb^{2+} concentration for Au+BP.”

Comment 7

A quantitative inconsistency exists between the reported sensitivity enhancement and LOD improvement. The angular sensitivity increases only from 132 to 281 deg/RIU, whereas the LOD is claimed to improve from 10^{-6} M to 10^{-19} M. The authors should provide explanation for how such a modest optical gain and the BP-COOH affinity could together yield a 13-order-of-magnitude reduction in LOD.

Response:

We are very grateful to the reviewer for pointing out this important quantitative issue. We understand that the reviewer is concerned that the refractive-index sensitivity enhancement alone appears insufficient to explain the large improvement in the detectable Pb^{2+} concentration range. This comment helped us clarify that the LOD improvement in this work arises from the combined effect of optical transduction enhancement and interfacial Pb^{2+} interaction, rather than from bulk refractive-index sensitivity enhancement alone.

We first clarified the numerical comparison using the newly added control measurements. Based on three independently prepared bare Au chips, bare Au shows a distinguishable Pb^{2+} response from 10^{-5} M (Fig. S4). Based on three independently prepared Au+BP chips, Au+BP shows a distinguishable Pb^{2+} response from 10^{-7} M (Fig. S5). Under the same distinguishability criterion, Au+BP-COOH shows a distinguishable Pb^{2+} response down to 10^{-19} M (Fig. 5 and Table 1). Therefore, the improvement relative to bare Au is 14 orders of magnitude under the present experimental conditions. In the revised refractive-index calibration, the angular sensitivity increases from 127 deg/RIU for bare Au to 259 deg/RIU for Au+BP-COOH (Fig. 3). This comparison clarifies that the angular sensitivity enhancement provides the optical transduction basis, while the full LOD improvement must also involve the interfacial Pb^{2+} interaction introduced by BP-COOH.

The reason is that the refractive-index sensitivity and the Pb^{2+} detection limit describe two different aspects of the sensing process. The refractive-index calibration in Fig. 3 measures the optical transduction ability of the SPR interface toward bulk refractive-index changes. In contrast, the Pb^{2+} LOD is determined by both the optical response of the SPR interface and the interfacial accumulation/coordination of Pb^{2+} at the BP-COOH functional layer. Therefore, the LOD is not expected to scale linearly with the bulk refractive-index sensitivity.

To clarify this point, we added two levels of control comparison. The first level is the optical comparison among bare Au, Au+Graphene, Au+MoS_2, Au+BP and Au+BP-COOH. This comparison shows that the BP-based interfaces provide stronger angular SPR responses than bare Au and the other tested two-dimensional overlayers. Importantly, Au+BP and Au+BP-COOH show very close refractive-index sensitivities, 256 and 259 deg/RIU, respectively, indicating that carboxyl functionalization preserves the BP-related optical response.

The second level is the Pb^{2+} sensing comparison among bare Au, Au+BP and Au+BP-COOH. Bare Au shows a distinguishable Pb^{2+} response from 10^{-5} M, while Au+BP shows a distinguishable response from 10^{-7} M. This comparison indicates that the BP overlayer improves the Pb^{2+} sensing response relative to bare Au. After introducing the BP-COOH functional layer, the detectable Pb^{2+} concentration is extended to 10^{-19} M. Because Au+BP and Au+BP-COOH have similar refractive-index sensitivities but very different Pb^{2+} detectable concentration regimes, the large LOD improvement is attributed to the combined contribution of the BP-related optical response and the oxygen-containing interfacial sites of BP-COOH for Pb^{2+} interaction.

Thus, the revised manuscript clarifies that the refractive-index sensitivity enhancement provides the optical basis for improved SPR transduction, while the BP-COOH functional layer changes the interfacial Pb^{2+} interaction and shifts the detectable concentration regime. This combined optical-and-chemical contribution explains why the LOD improvement is much larger than the increase in bulk refractive-index sensitivity alone.

Changes made in the manuscript:

Manuscript, lines 193–215:

"To clarify the optical basis for the improved sensing performance of Au+BP-COOH, the refractive-index-dependent angular SPR responses of bare Au, Au+Graphene, Au+MoS_2, Au+BP and Au+BP-COOH were experimentally compared under the same sensing framework. As shown in Fig. 3a–e, the angle-scanned reflectance curves of all five structures shift toward larger incident angles when the refractive index of the sensing medium increases from n_s = 1.3320 to 1.3530. However, the magnitude of the resonance-angle displacement is different for different interfacial structures.

The corresponding resonance angles are summarized in Fig. 3f. Linear fitting gives angular sensitivities of 127 deg/RIU for bare Au, 157 deg/RIU for Au+Graphene, 168 deg/RIU for Au+MoS_2, 256 deg/RIU for Au+BP and 259 deg/RIU for Au+BP-COOH. Compared with bare Au, the introduction of two-dimensional overlayers increases the angular response of the Au-film SPR structure to refractive-index variation. Au+Graphene and Au+MoS_2 were used as reference two-dimensional overlayer structures because they also possess high-index confinement and interlayer-coupling-related optical characteristics. However, their angular sensitivities remain lower than those of Au+BP and Au+BP-COOH under the same refractive-index calibration conditions. This comparison indicates that the BP-based overlayer provides a stronger optical response than the other two-dimensional overlayers examined here. In addition, the angular sensitivities of Au+BP and Au+BP-COOH are close to each other, indicating that carboxyl functionalization preserves the BP-related optical response while introducing oxygen-containing interfacial sites for subsequent Pb^{2+} interaction.

The simulated angular SPR response and electric-field distribution of the Au-film structure with and without a BP overlayer are provided in Fig. S3. The simulation shows the same enhancement trend after introducing the BP overlayer and provides a theoretical reference for the BP-induced optical response. In the experimental Au+BP-COOH structure, the BP-COOH functional layer contains polymer-functionalized BP nanosheets, and therefore the experimental refractive-index calibration in Fig. 3 is used as the direct basis for evaluating the optical response of the actual sensing interface."

Manuscript, lines 259–264:

"To clarify the contribution of BP and BP-COOH to Pb^{2+} sensing, control measurements were performed on bare Au and Au+BP. The Pb^{2+} sensing results of bare Au and Au+BP are provided in Figs. S4 and S5, respectively. Bare Au shows a distinguishable Pb^{2+} response from 10^{-5} M, whereas Au+BP shows a distinguishable response from 10^{-7} M. This comparison indicates that the BP overlayer improves the Pb^{2+} sensing response relative to bare Au. By further introducing the BP-COOH functional layer, the detectable Pb^{2+} concentration is extended to 10^{-19} M, indicating that the oxygen-containing interfacial sites of BP-COOH play a key role in the ultralow-concentration Pb^{2+} response."

Manuscript, lines 308–315:

"Taken together, Fig. 5, Table 1 and the supporting results in Figs. S4–S32 establish the Pb^{2+} sensing performance and selectivity of Au+BP-COOH from five aspects. First, Au+BP-COOH enables ultralow-concentration Pb^{2+} sensing in pure water. Second, the comparison among bare Au, Au+BP and Au+BP-COOH distinguishes the BP-related optical contribution from the BP-COOH-related Pb^{2+} interaction contribution. Third, the time-dependent statistics of ten independently prepared Au+BP-COOH chips clarify the detection behavior at ultralow Pb^{2+} concentrations. Fourth, the detectable concentration regime for Pb^{2+} remains markedly lower than those of Cd^{2+}, Cu^{2+}, Zn^{2+}, Ni^{2+}, Ca^{2+} and Mg^{2+}. Fifth, Pb^{2+} can be detected in mixed-ion solution and drinking water after subtracting the corresponding matrix baselines, and Au+BP-COOH retains sensing responses in regeneration and storage-stability tests."

Supporting Information, lines 36–40:

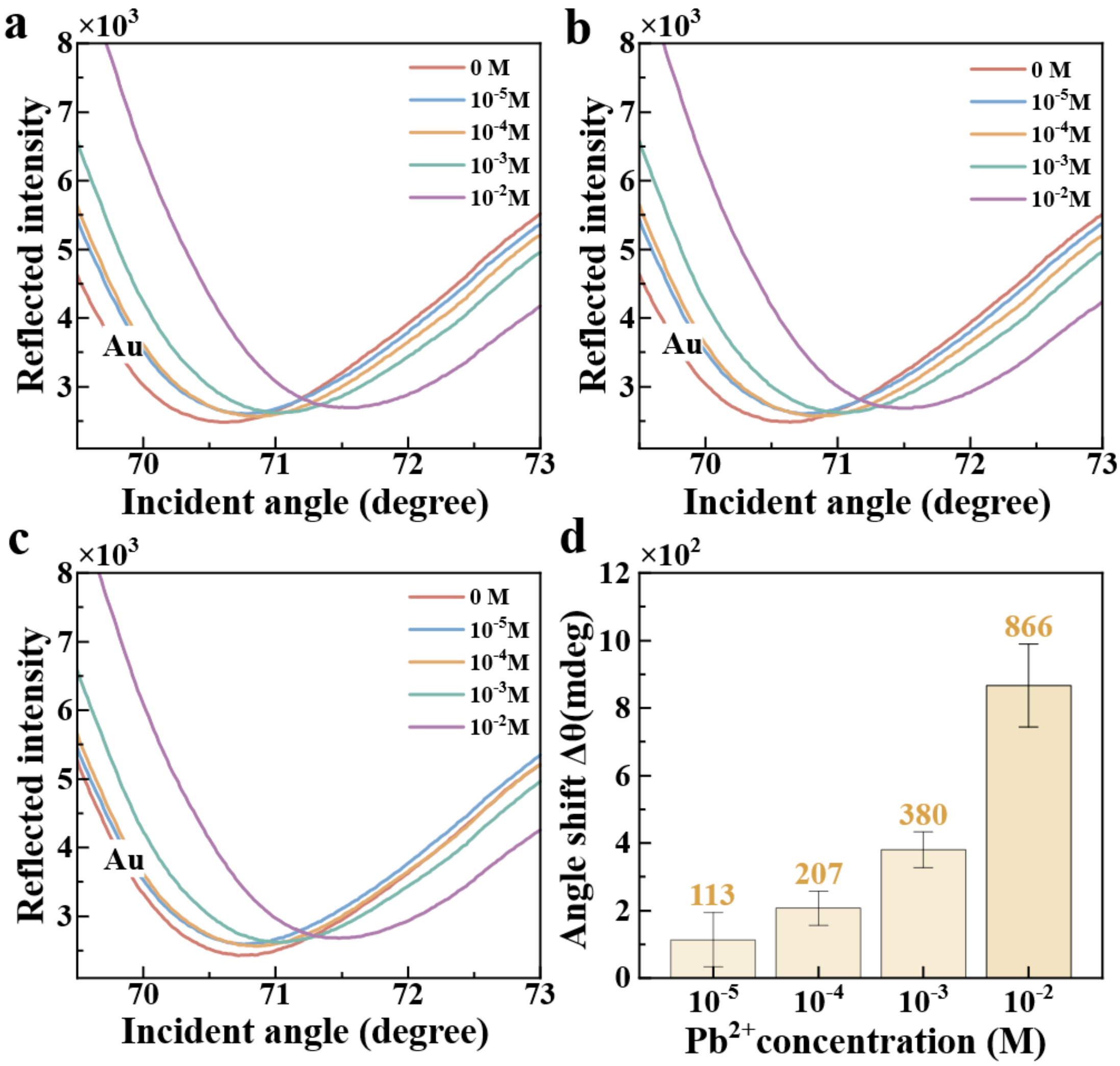

"**Fig. S4 Pb^{2+} sensing results obtained from bare Au. (a–c)** Angle-scanned reflectance curves of three independently prepared bare Au chips measured for Pb^{2+} concentrations of 0, 10^{-5}, 10^{-4}, 10^{-3} and 10^{-2} M. **(d)** Resonance-angle shift Δθ as a function of Pb^{2+} concentration for bare Au."

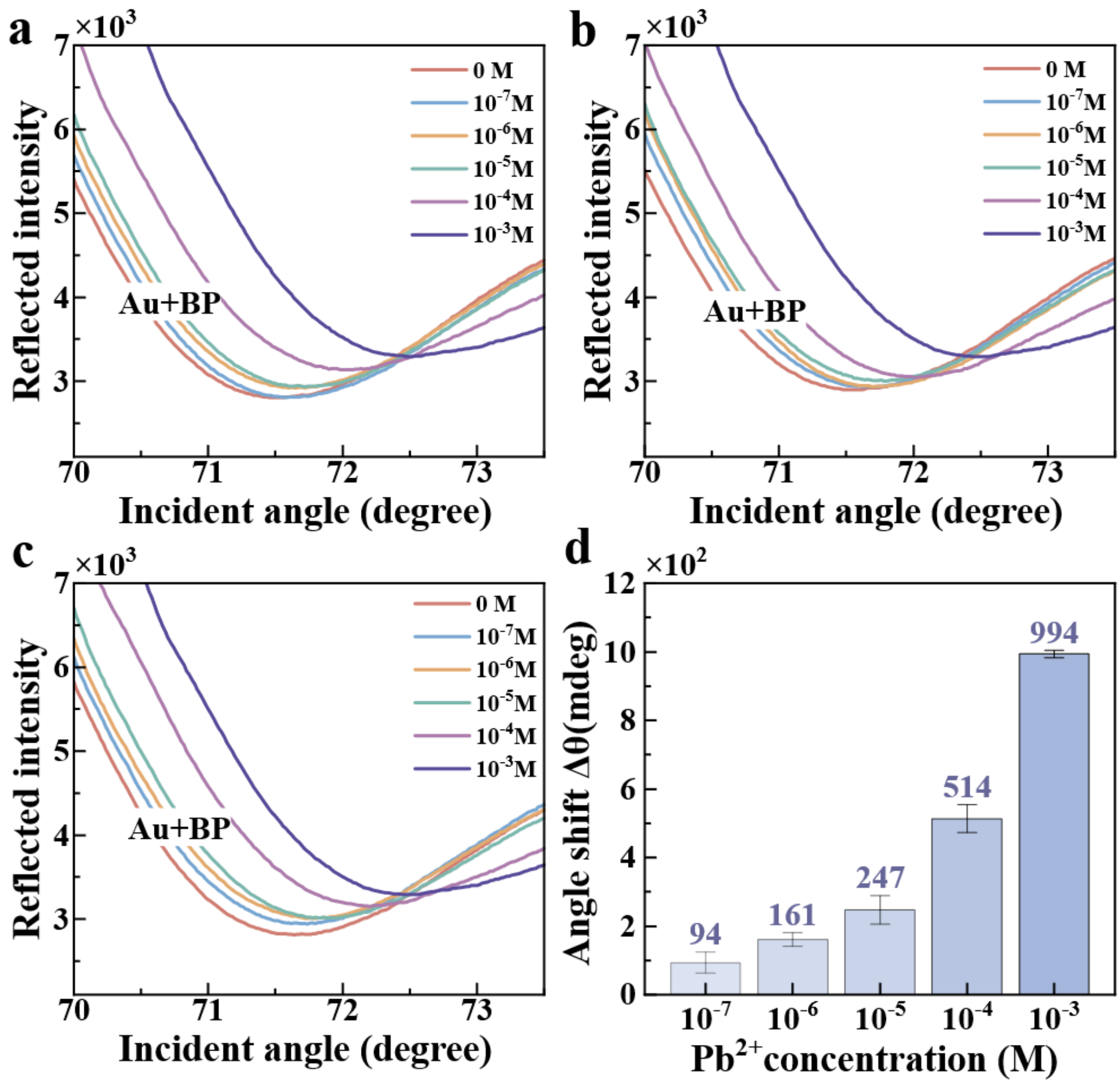

"Fig. S5 Pb^{2+} sensing results obtained from Au+BP. (a–c) Angle-scanned reflectance curves of three independently prepared Au+BP chips measured for Pb^{2+} concentrations of 0, 10^{-7}, 10^{-6}, 10^{-5}, 10^{-4} and 10^{-3} M. **(d)** Resonance-angle shift Δθ as a function of Pb^{2+} concentration for Au+BP."

Comment 8

The experimental protocol states that the SPR signal is collected 30 s after static sample introduction at 10^{-19} M in a 400 μL cell. Classical diffusion-limited transport theory predicts that analyte arrival at a planar surface under such conditions would require hours to days. The authors should clarify whether a non-classical transport or local preconcentration mechanism contributes to the sensing process and substantiate this possibility through quantitative kinetic analysis, transport modeling, or simulations of interfacial ion enrichment.

Response:

We deeply appreciate the reviewer's careful consideration of the transport issue at ultralow Pb^{2+} concentration. We fully understand the importance of this concern, because at 10^{-19} M the number of Pb^{2+} ions in 400 μL solution is extremely small, and the detection behavior at this concentration should be examined carefully. We apologize that the measurement protocol in the original manuscript was not described clearly enough, which may have led to the misunderstanding that the

sensing experiment was performed by static sample introduction in a quiescent 400 μL cell. This valuable comment prompted us to clarify the actual flow-cell measurement protocol and to further investigate the statistical detection behavior at the ultralow-concentration end.

In the revised manuscript, we clarified that the sensing measurements were performed in a flow cell. For each measurement, 400 μL of sample solution was introduced into the flow cell at a flow rate of 200 μL/min. The SPR response was recorded at 30, 60, 90 and 120 s. Therefore, the sample was delivered through the sensing cell during the measurement period. Under this flow-cell protocol, the measured response is related to sample delivery, ion arrival at the sensing region, and interfacial interaction between Pb^{2+} and BP-COOH.

We also understand the reviewer's deeper concern that, even under flow-cell measurement conditions, 10^{-19} M Pb^{2+} lies in a finite-ion-number regime and should not be evaluated only from one chip or one time point. Therefore, we performed time-dependent sensing measurements on ten independently prepared Au+BP-COOH chips at Pb^{2+} concentrations from 10^{-19} to 10^{-15} M. The individual time-dependent results are provided in Figs. S6–S15, and the statistical results are summarized in Fig. S16 and Table 1.

The added statistics show a clear concentration- and time-dependent detection behavior. At 10^{-19} M Pb^{2+}, 1/10, 2/10, 4/10 and 4/10 chips showed distinguishable responses at 30, 60, 90 and 120 s, respectively. Therefore, in the revised manuscript, we describe 10^{-19} M as a time-dependent statistical detection regime. In contrast, from 10^{-18} to 10^{-15} M, all ten independently prepared chips showed distinguishable responses at every detection time. These results clarify that the response at the lowest concentration is time-dependent and statistically distributed, while the responses from 10^{-18} to 10^{-15} M are consistently distinguishable under the same flow-cell measurement protocol.

Changes made in the manuscript:

Manuscript, Table 1, lines 238–240:

"**Table 1.** Time-dependent detection statistics of Pb^{2+} obtained from ten independently prepared Au+BP-COOH chips.

**Pb^{2+} concentration**	**30 s**	**60 s**	**90 s**	**120 s**
**10^{-19} M**	1/10	2/10	4/10	4/10
**10^{-18} M**	10/10	10/10	10/10	10/10
**10^{-17} M**	10/10	10/10	10/10	10/10
**10^{-16} M**	10/10	10/10	10/10	10/10
**10^{-15} M**	10/10	10/10	10/10	10/10

A chip was counted as distinguishable when the resonance-angle shift Δθ exceeded 21 mdeg. The statistics were obtained from ten independently prepared Au+BP-COOH chips shown in Figs. S6–S15."

Manuscript, Fig. 5, lines 242–252:

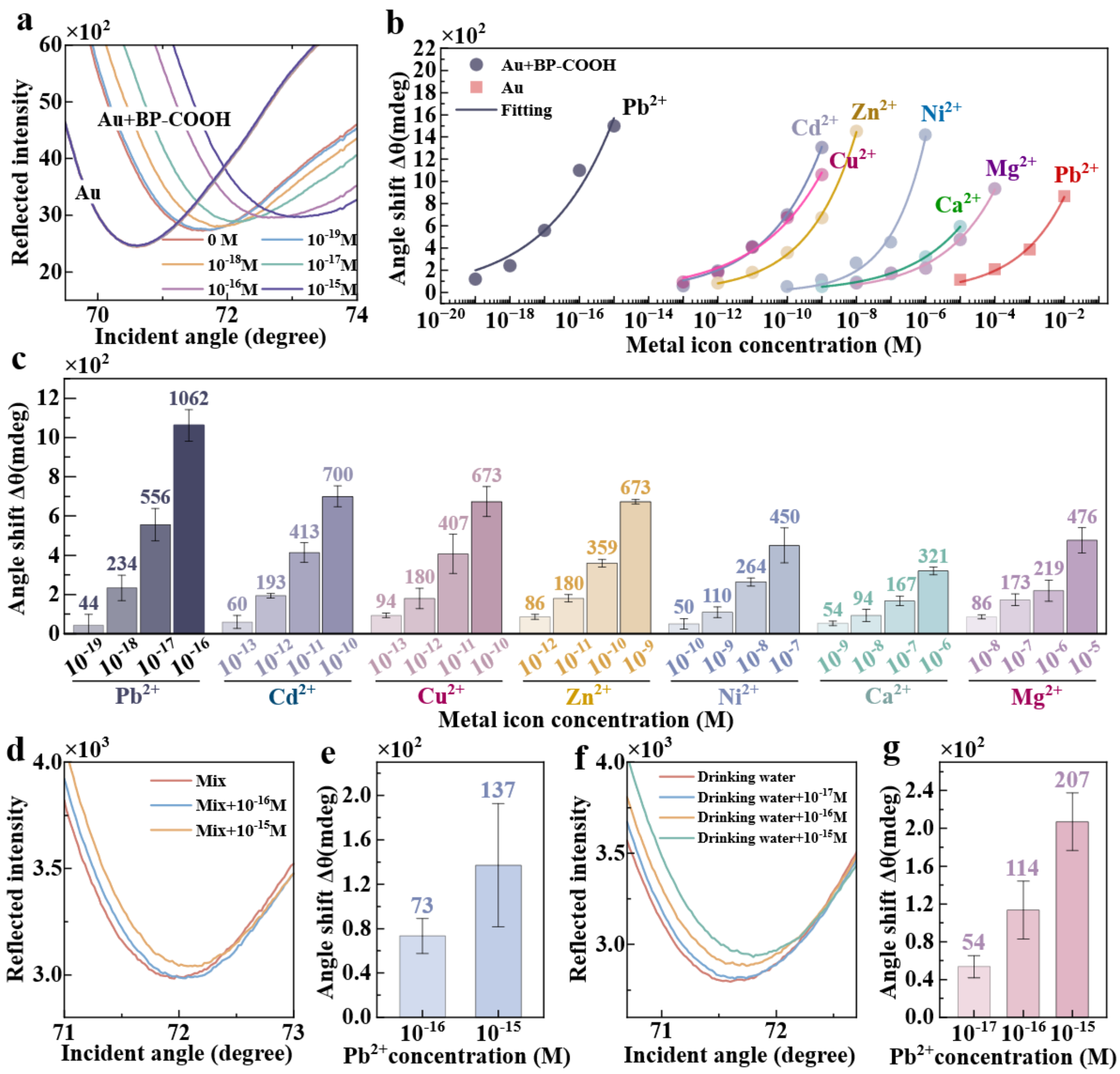

"Fig. 5. Pb^{2+} sensing response of Au+BP-COOH and comparison with other metal ions in pure water, mixed-ion solution, and drinking water. (a) Angle-scanned reflectance curves of Au+BP-COOH measured for Pb^{2+} concentrations of 0, 10^{-19}, 10^{-18}, 10^{-17}, 10^{-16} and 10^{-15} M, together with the reflectance curve of bare Au. **(b)** Resonance-angle shift Δθ as a function of metal-ion concentration for Au+BP-COOH and bare Au. The data points are mean values from the corresponding statistical results in c and Fig. S4, and the solid lines represent nonlinear fitting curves. **(c)** Resonance-angle shift Δθ values of Au+BP-COOH measured at different concentrations of Pb^{2+}, Cd^{2+}, Cu^{2+}, Zn^{2+}, Ni^{2+}, Ca^{2+} and Mg^{2+}. The Pb^{2+} data are from the ten independently prepared Au+BP-COOH chips shown in Figs. S6–S15 and statistically summarized in Fig. S16, and the data for Cd^{2+}, Cu^{2+}, Zn^{2+}, Ni^{2+}, Ca^{2+} and Mg^{2+} are from the three independently prepared Au+BP-COOH chips shown in Figs. S25–S30. **(d)** Angle-scanned reflectance curves of Au+BP-COOH measured in Mix, Mix + 10^{-16} M Pb^{2+} and Mix + 10^{-15} M Pb^{2+}. **(e)** Net resonance-angle shift Δθ as a function of Pb^{2+} concentration in the mixed-ion solution. The statistical data are from Fig. S31. **(f)** Angle-scanned reflectance curves of Au+BP-COOH measured in drinking water, drinking water + 10^{-17} M Pb^{2+}, drinking water + 10^{-16} M Pb^{2+} and drinking water + 10^{-15} M Pb^{2+}. **(g)** Net resonance-angle shift Δθ as a function of Pb^{2+} concentration in drinking water. The statistical data are from Fig. S32."

Manuscript, lines 265–272:

“To examine the statistical detection behavior of Au+BP-COOH at ultralow Pb^{2+} concentrations, time-dependent sensing measurements were performed on ten independently prepared Au+BP-COOH chips. The detection statistics are summarized in Table 1, and the corresponding individual sensing results are provided in Figs. S6–S15. At 10^{-19} M Pb^{2+}, 1/10, 2/10, 4/10 and 4/10 chips show distinguishable responses at 30, 60, 90 and 120 s, respectively, indicating that this concentration is located in a time-dependent statistical detection regime. In contrast, at 10^{-18} M and higher Pb^{2+} concentrations, all ten chips show distinguishable responses at each detection time. The statistical resonance-angle shifts obtained from these ten chips are summarized in Fig. S16. These results show the time-dependent detectability of Au+BP-COOH at 10^{-19} M and the consistent detectability from 10^{-18} to 10^{-15} M.”

Manuscript, lines 398–410:

“SPR measurements were performed on a Dynechem SPR system using 650 nm incident light at 25 °C. The angular scanning range was 65°–80°, and the angular step was 19–21 mdeg. For each measurement, 400 μL of sample solution was introduced into the flow cell at a flow rate of 200 μL/min. The resonance angle was directly read from the angle corresponding to the minimum reflectance point without curve fitting.

For Pb^{2+} time-dependent sensing measurements, the SPR response was recorded at 30, 60, 90 and 120 s. Unless otherwise specified, the resonance-angle shift $\Delta\theta$ used for statistical comparison was extracted at 120 s. For pure-water sensing measurements and single-ion sensing measurements, pure water was first measured on the same chip as the baseline before the ion solution was introduced, and $\Delta\theta$ was defined relative to this corresponding pure-water baseline. The same baseline procedure was applied to Pb^{2+} and to the competing-ion measurements. For mixed-ion and drinking-water measurements, the net $\Delta\theta$ was obtained by subtracting the corresponding matrix baseline.

For the time-dependent Pb^{2+} detection statistics, ten independently prepared Au+BP-COOH chips were measured from pure water to increasing Pb^{2+} concentrations. For single-ion selectivity, mixed-ion interference and drinking-water measurements, three independently prepared Au+BP-COOH chips were used unless otherwise specified. Error bars represent the standard deviation from independently prepared chips.”

Manuscript, lines 419–424:

“The resonance-angle shift $\Delta\theta$ was used to evaluate the sensing response. A response was considered distinguishable when $\Delta\theta$ exceeded 21 mdeg. This operational distinguishability threshold was selected according to the angular scanning step of the SPR measurement and the baseline stability of Au+BP-COOH in pure water. Repeated pure-water measurements on the same Au+BP-COOH chip over 50 min showed a maximum resonance-angle drift of 21 mdeg and a standard deviation of 0.00714 deg (Fig. S33). The LOD was defined as the lowest concentration at which a distinguishable response was obtained under the corresponding sensing condition.”

Supporting Information, lines 41–80:

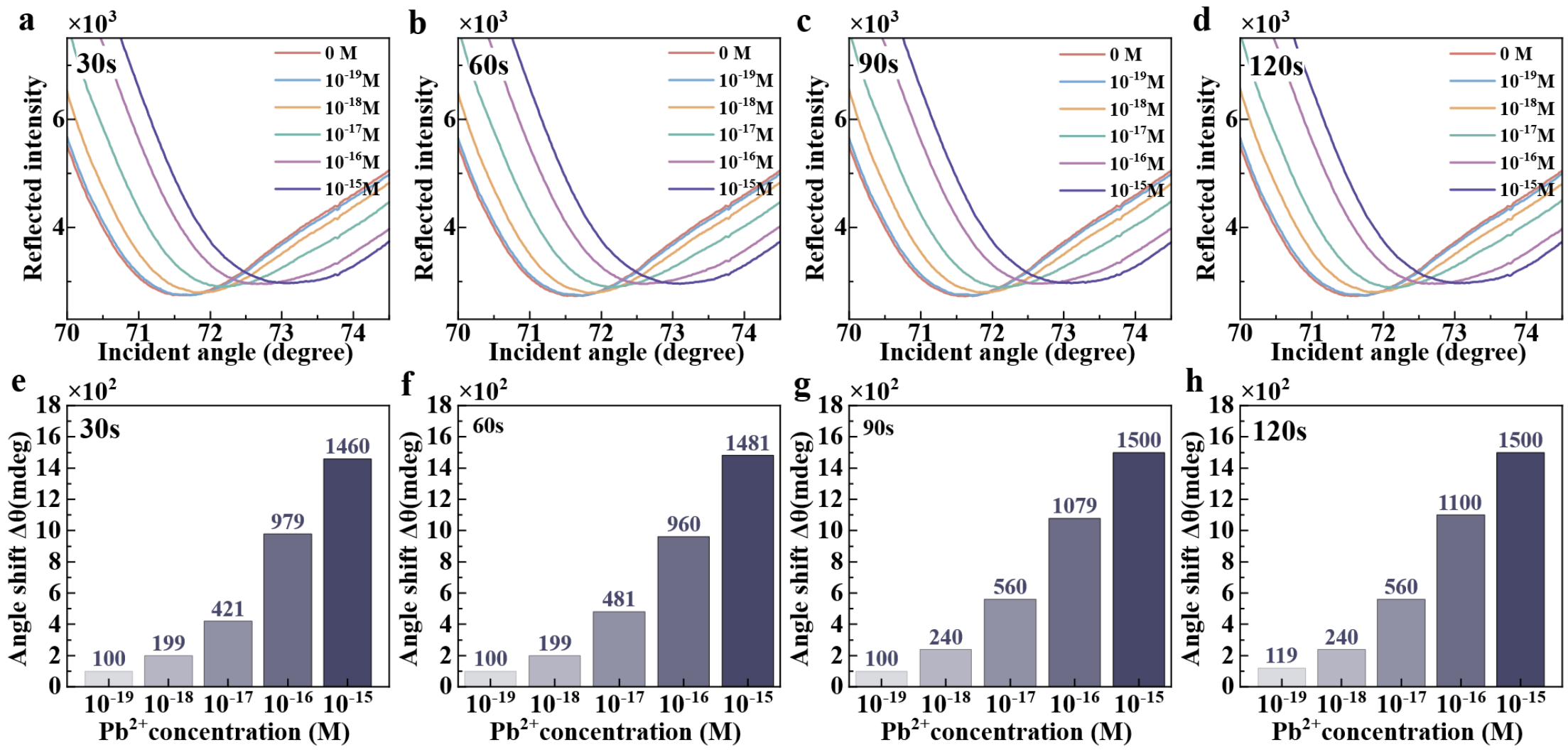

"Fig. S6 Time-dependent Pb^{2+} sensing results obtained from one Au+BP-COOH chip. (a–d) Angle-scanned reflectance curves measured for Pb^{2+} concentrations of 0, 10^{-19}, 10^{-18}, 10^{-17}, 10^{-16} and 10^{-15} M at 30, 60, 90 and 120 s, respectively. (e–h) Corresponding resonance-angle shift Δθ values at 30, 60, 90 and 120 s, respectively."

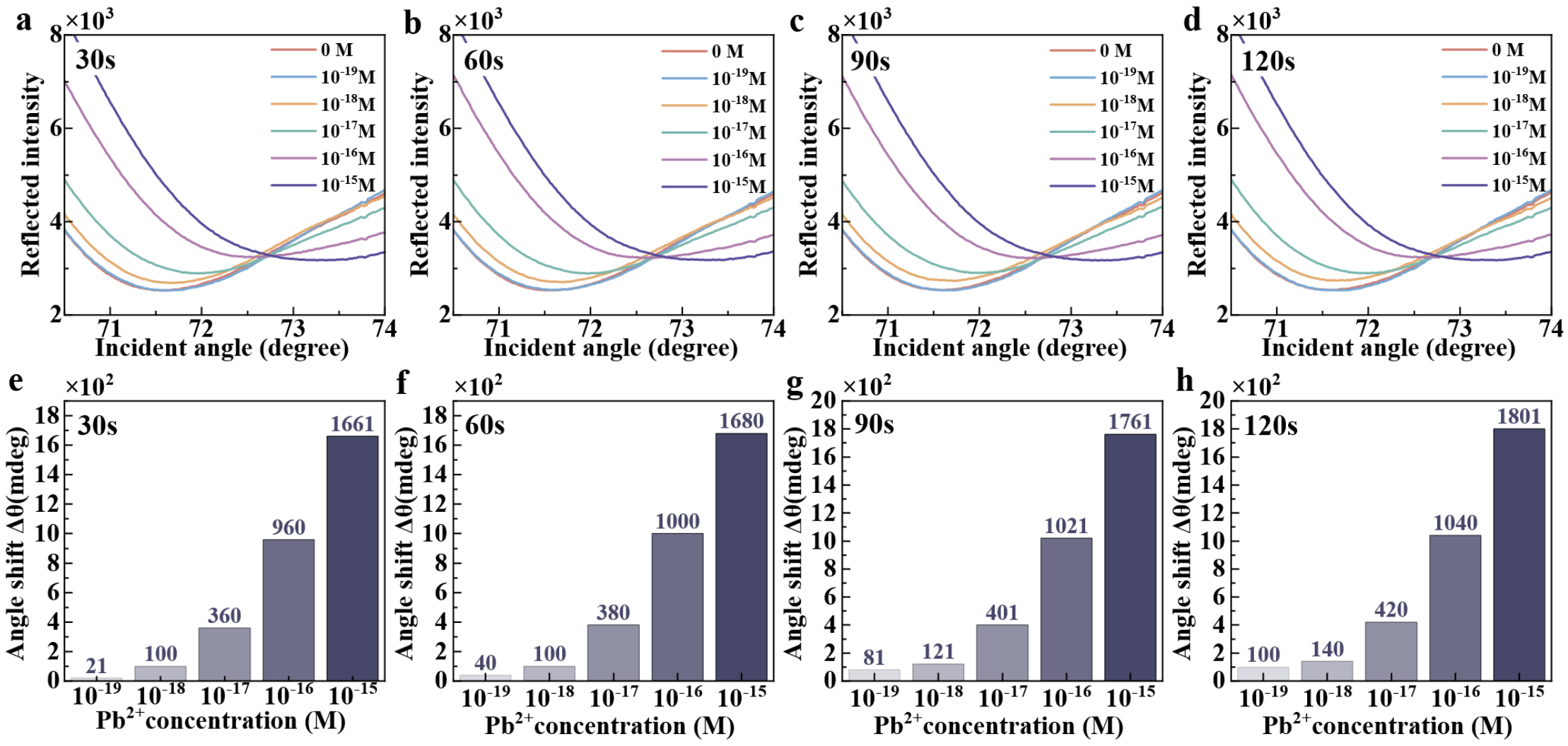

"Fig. S7 Time-dependent Pb^{2+} sensing results obtained from one Au+BP-COOH chip. (a–d) Angle-scanned reflectance curves measured for Pb^{2+} concentrations of 0, 10^{-19}, 10^{-18}, 10^{-17}, 10^{-16} and 10^{-15} M at 30, 60, 90 and 120 s, respectively. (e–h) Corresponding resonance-angle shift Δθ values at 30, 60, 90 and 120 s, respectively."

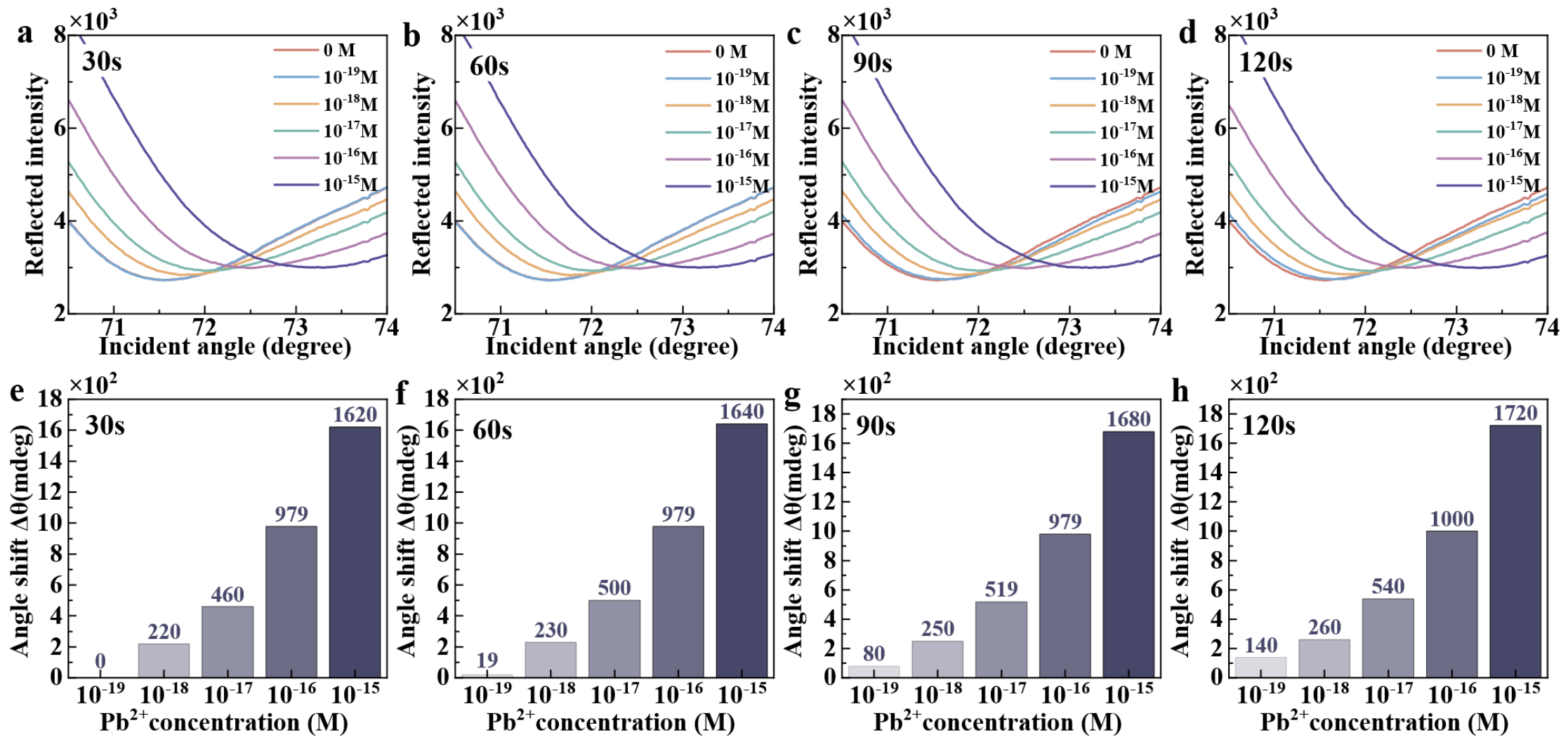

"Fig. S8 Time-dependent Pb^{2+} sensing results obtained from one Au+BP-COOH chip. (a–d) Angle-scanned reflectance curves measured for Pb^{2+} concentrations of 0, 10^{-19}, 10^{-18}, 10^{-17}, 10^{-16} and 10^{-15} M at 30, 60, 90 and 120 s, respectively. **(e–h)** Corresponding resonance-angle shift Δθ values at 30, 60, 90 and 120 s, respectively."

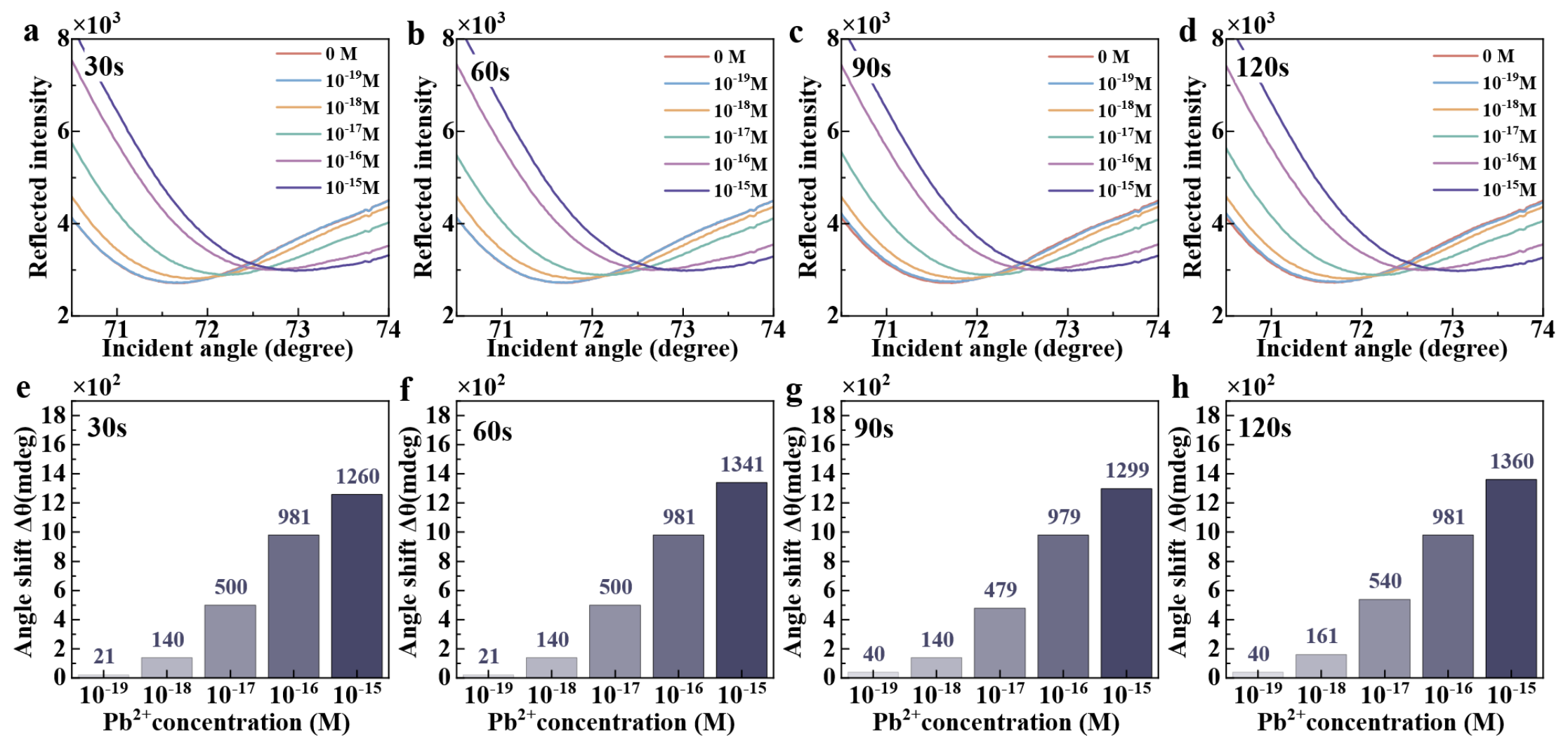

"Fig. S9 Time-dependent Pb^{2+} sensing results obtained from one Au+BP-COOH chip. (a–d) Angle-scanned reflectance curves measured for Pb^{2+} concentrations of 0, 10^{-19}, 10^{-18}, 10^{-17}, 10^{-16} and 10^{-15} M at 30, 60, 90 and 120 s, respectively. **(e–h)** Corresponding resonance-angle shift Δθ values at 30, 60, 90 and 120 s, respectively."

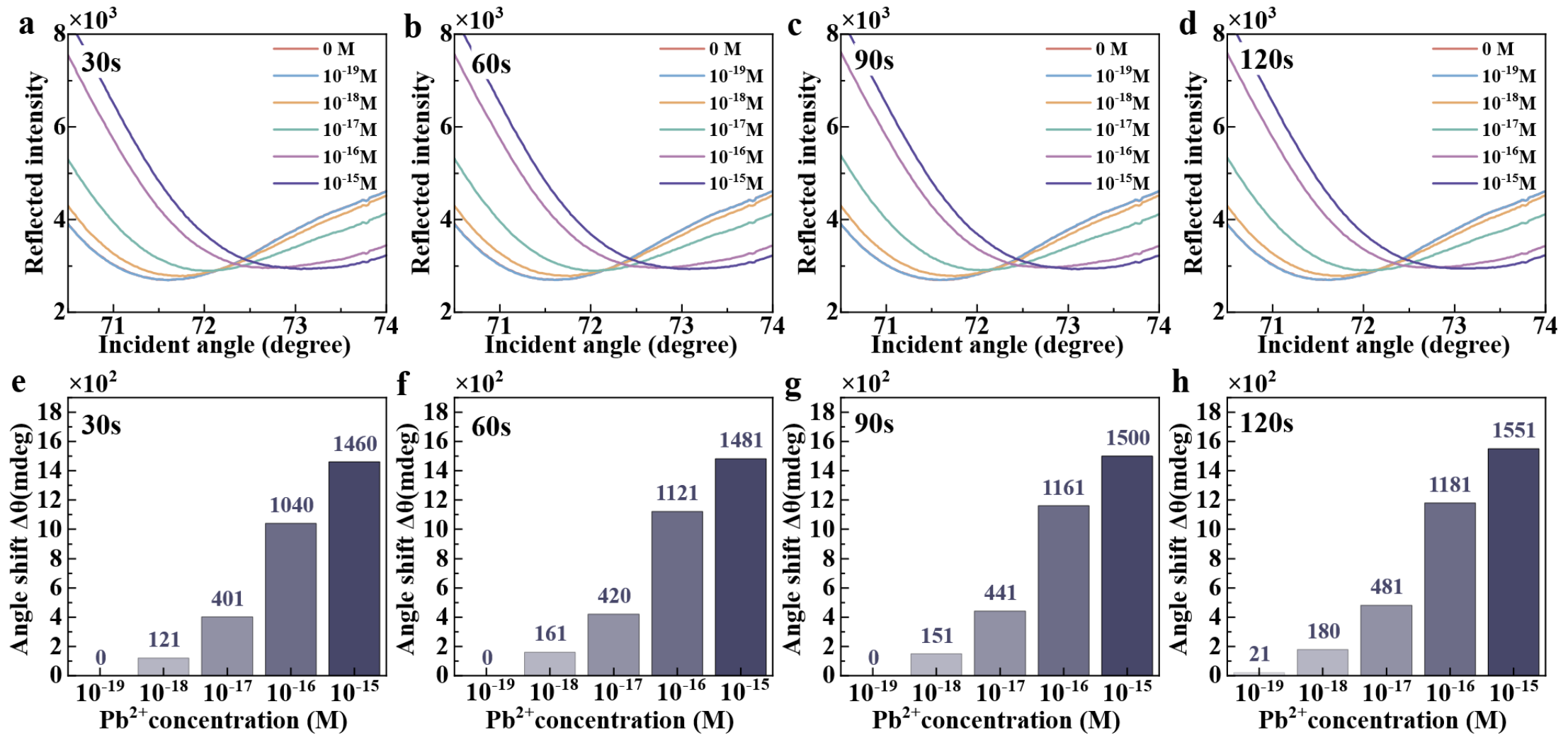

“Fig. S10 Time-dependent Pb^{2+} sensing results obtained from one Au+BP-COOH chip. (a–d) Angle-scanned reflectance curves measured for Pb^{2+} concentrations of 0, 10^{-19}, 10^{-18}, 10^{-17}, 10^{-16} and 10^{-15} M at 30, 60, 90 and 120 s, respectively. **(e–h)** Corresponding resonance-angle shift $\Delta\theta$ values at 30, 60, 90 and 120 s, respectively.”

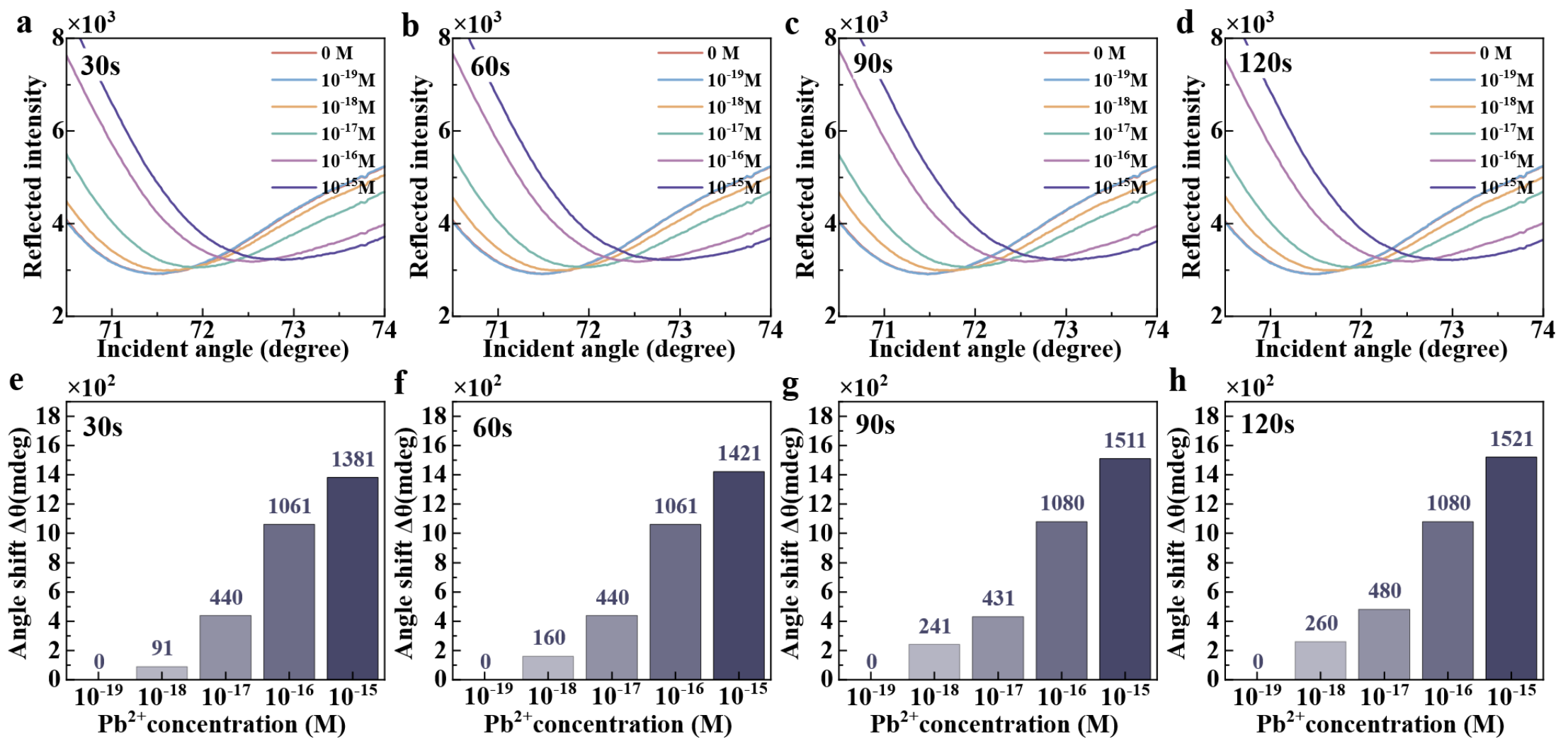

“Fig. S11 Time-dependent Pb^{2+} sensing results obtained from one Au+BP-COOH chip. (a–d) Angle-scanned reflectance curves measured for Pb^{2+} concentrations of 0, 10^{-19}, 10^{-18}, 10^{-17}, 10^{-16} and 10^{-15} M at 30, 60, 90 and 120 s, respectively. **(e–h)** Corresponding resonance-angle shift $\Delta\theta$ values at 30, 60, 90 and 120 s, respectively.”

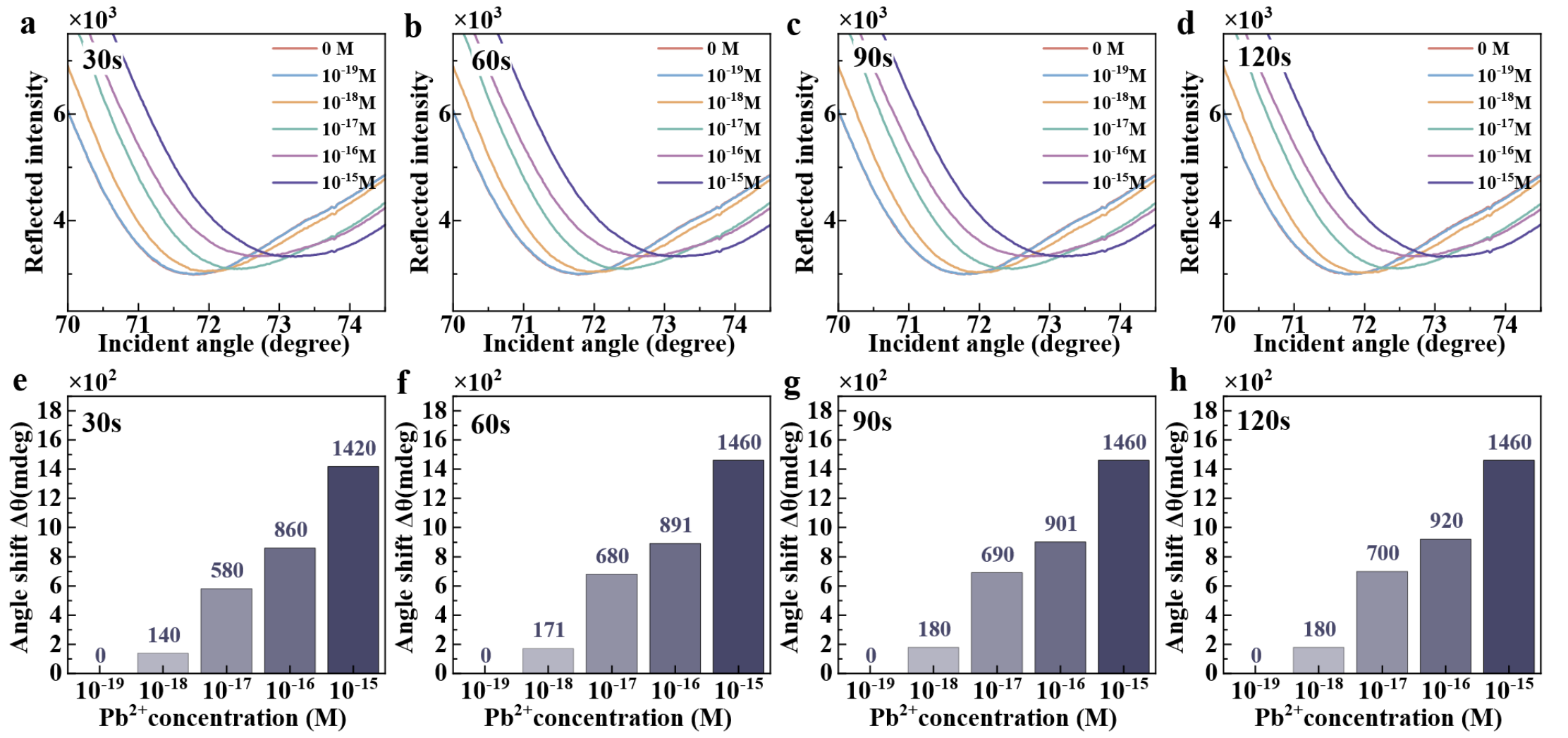

“Fig. S12 Time-dependent Pb^{2+} sensing results obtained from one Au+BP-COOH chip. (a–d) Angle-scanned reflectance curves measured for Pb^{2+} concentrations of 0, 10^{-19}, 10^{-18}, 10^{-17}, 10^{-16} and 10^{-15} M at 30, 60, 90 and 120 s, respectively. **(e–h)** Corresponding resonance-angle shift Δθ values at 30, 60, 90 and 120 s, respectively.”

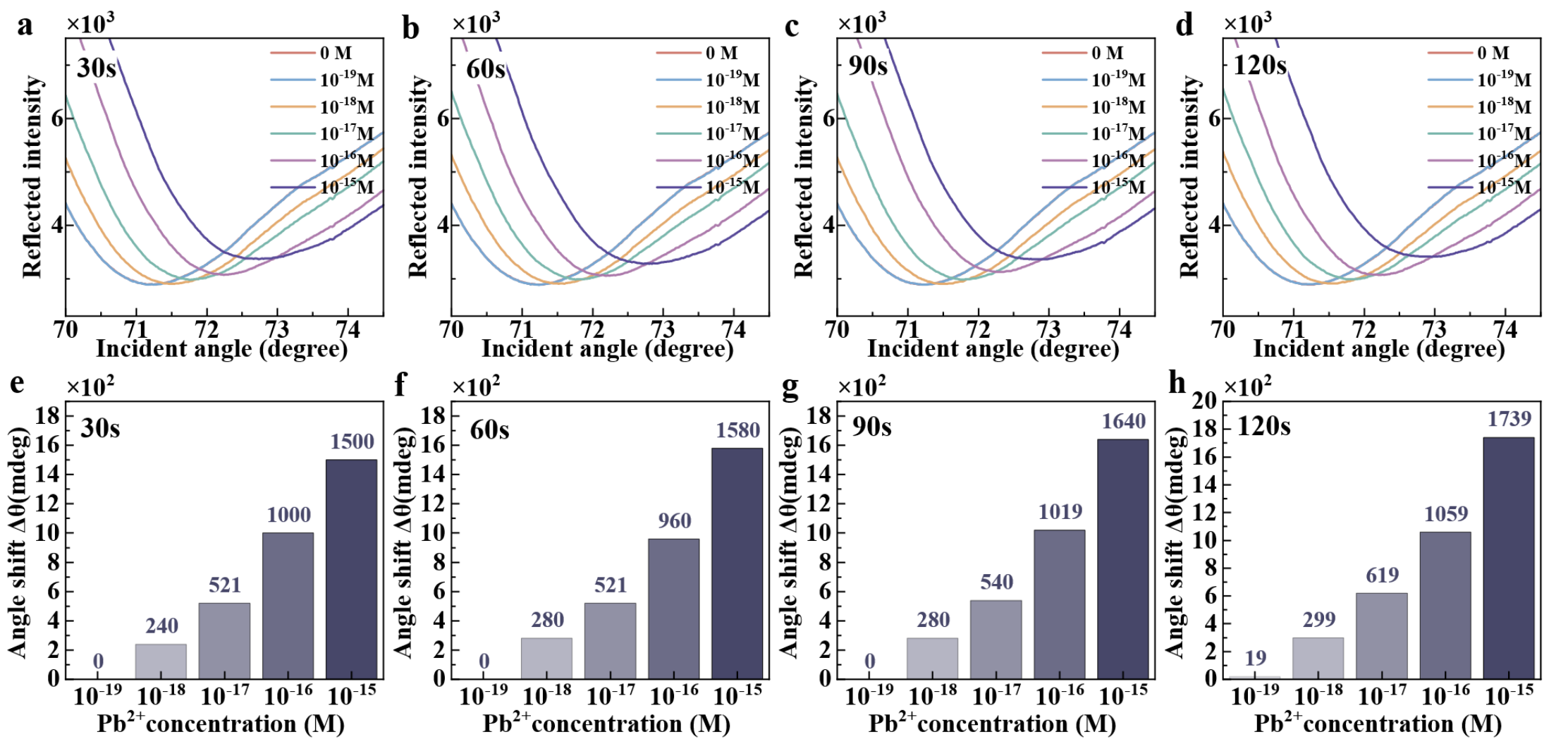

“Fig. S13 Time-dependent Pb^{2+} sensing results obtained from one Au+BP-COOH chip. (a–d) Angle-scanned reflectance curves measured for Pb^{2+} concentrations of 0, 10^{-19}, 10^{-18}, 10^{-17}, 10^{-16} and 10^{-15} M at 30, 60, 90 and 120 s, respectively. **(e–h)** Corresponding resonance-angle shift Δθ values at 30, 60, 90 and 120 s, respectively.”

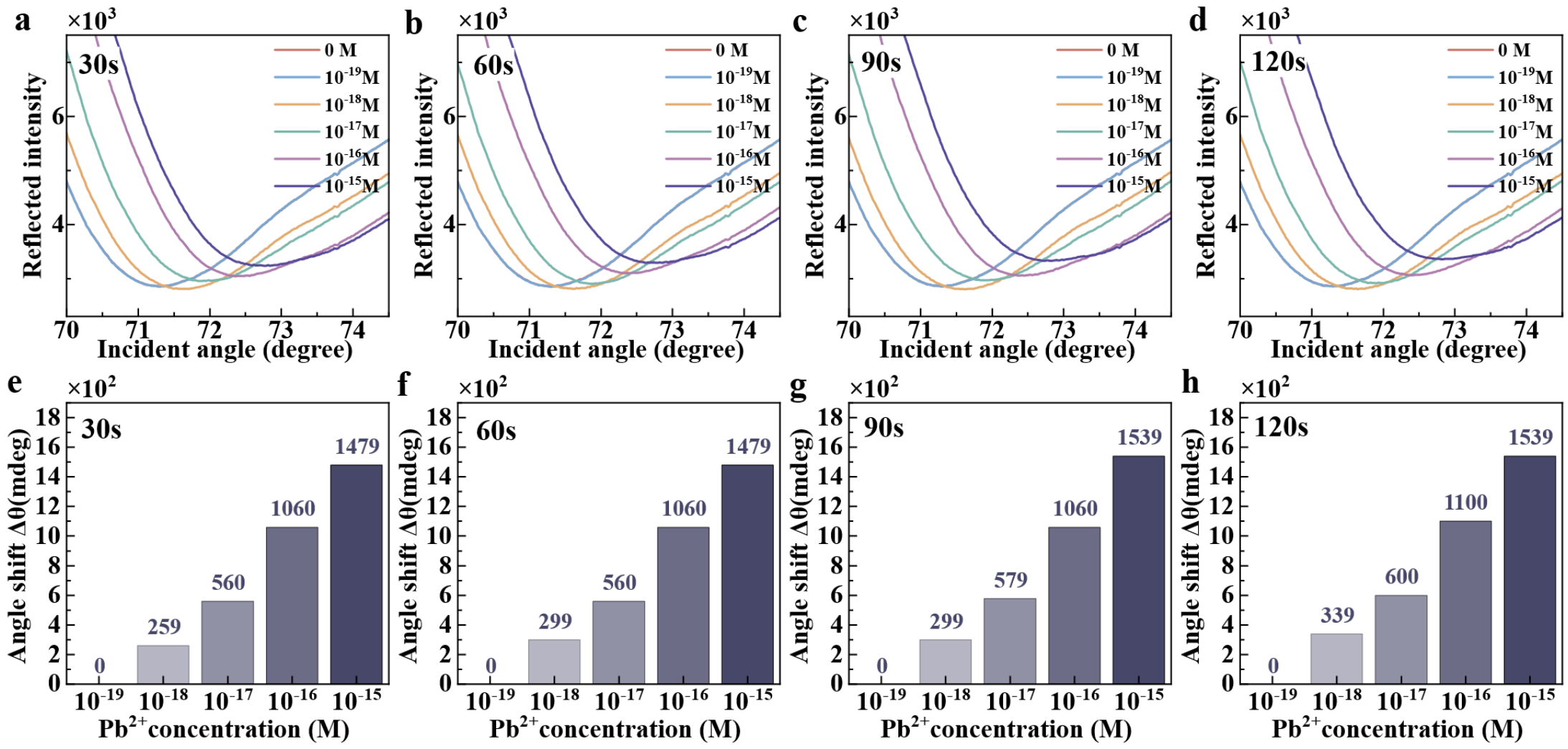

"Fig. S14 Time-dependent Pb^{2+} sensing results obtained from one Au+BP-COOH chip. (a–d) Angle-scanned reflectance curves measured for Pb^{2+} concentrations of 0, 10^{-19}, 10^{-18}, 10^{-17}, 10^{-16} and 10^{-15} M at 30, 60, 90 and 120 s, respectively. **(e–h)** Corresponding resonance-angle shift Δθ values at 30, 60, 90 and 120 s, respectively."

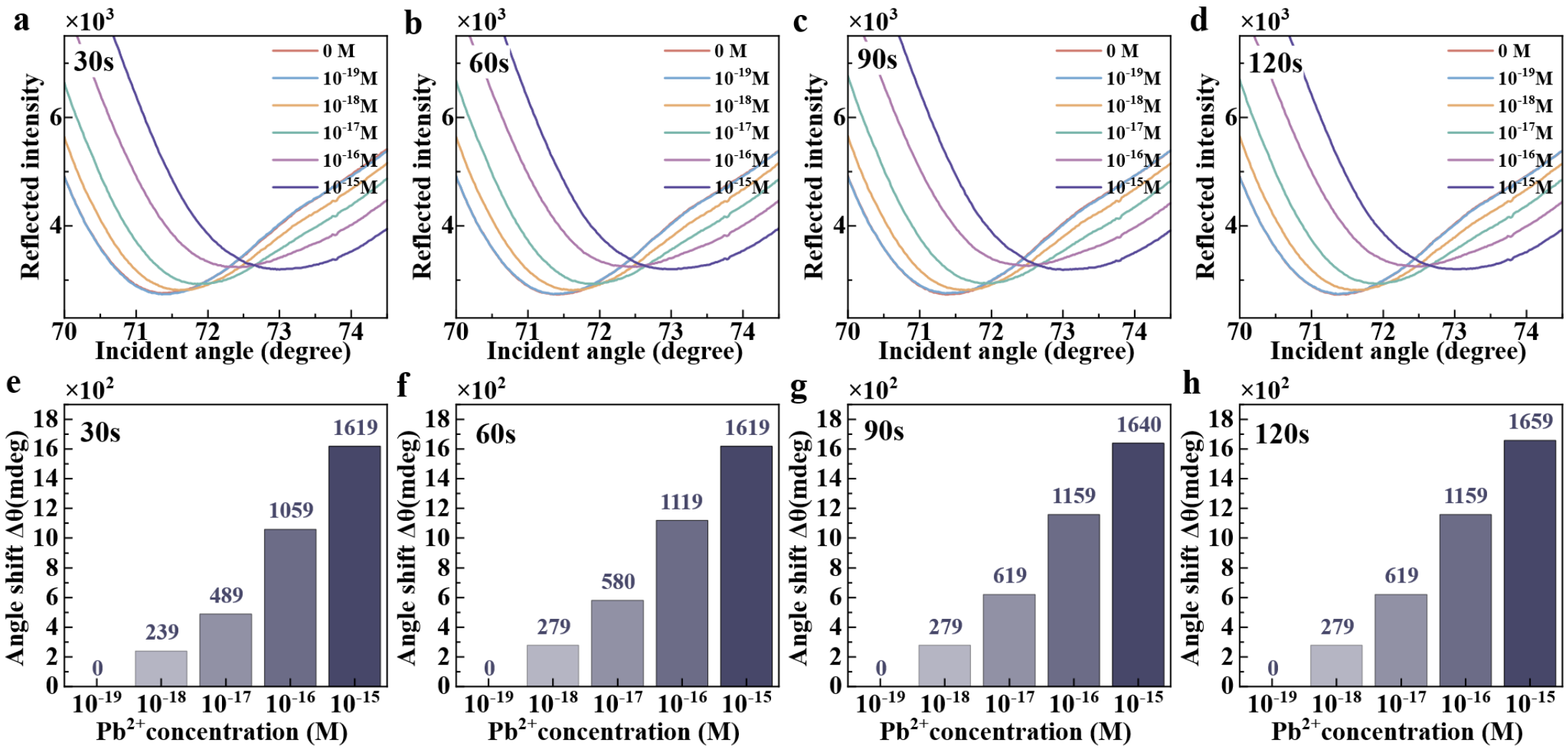

"Fig. S15 Time-dependent Pb^{2+} sensing results obtained from one Au+BP-COOH chip. (a–d) Angle-scanned reflectance curves measured for Pb^{2+} concentrations of 0, 10^{-19}, 10^{-18}, 10^{-17}, 10^{-16} and 10^{-15} M at 30, 60, 90 and 120 s, respectively. **(e–h)** Corresponding resonance-angle shift Δθ values at 30, 60, 90 and 120 s, respectively."

Supporting Information, lines 81–84:

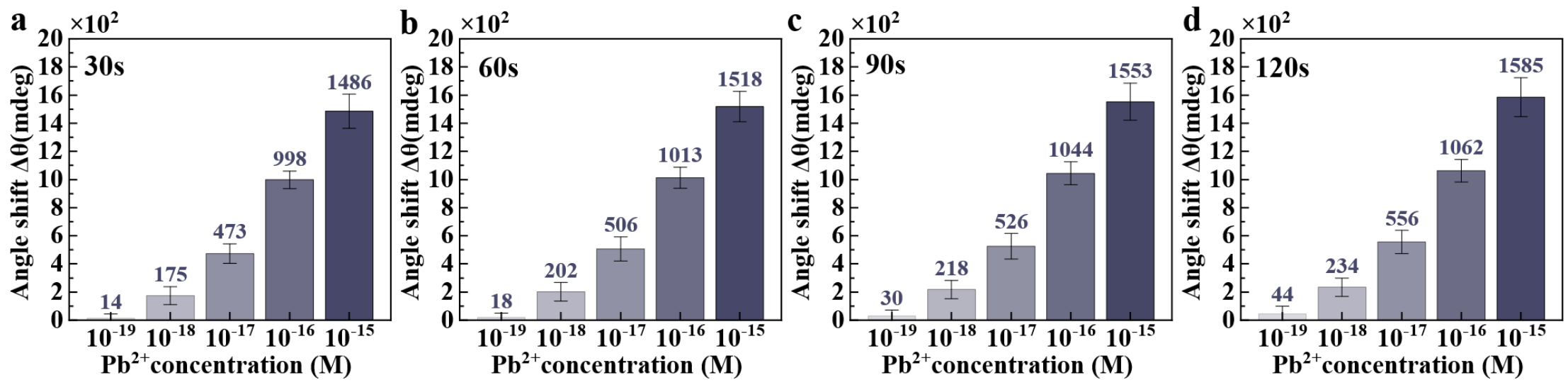

"Fig. S16 Statistical Pb^{2+} sensing results obtained from the ten Au+BP-COOH chips shown in

Figs. S6–S15 at different detection times. (a–d) Resonance-angle shift $\Delta\theta$ as a function of Pb^{2+} concentration at 30, 60, 90 and 120 s, respectively. Error bars are from the ten independently prepared Au+BP-COOH chips shown in Figs. S6–S15.**”**

Manuscript ID: NCOMMS-26-039241B

Title: Built-in electric field-boosted attomolar Pb^{2+} sensing on carboxylated black phosphorene

Reviewer 1

The authors have carefully addressed all of my previous comments and significantly improved the manuscript. The additional experiments and discussion substantially strengthen the manuscript and support the main conclusions. I have no further comments and recommend publication in its present form.

Response to Reviewer 1:

We sincerely thank the Reviewer for the careful, professional, and insightful assessment of our revised manuscript. We greatly appreciate the Reviewer's recognition of the additional experiments and discussion, as well as the positive evaluation that these revisions have substantially strengthened the manuscript and supported its main conclusions. The Reviewer's rigorous comments throughout the review process demonstrated exceptional scholarly depth and played an important role in improving the completeness, clarity, and scientific rigor of our work. We are deeply grateful for the time, expertise, and thoughtful guidance devoted to the evaluation of our manuscript, and we sincerely appreciate the recommendation for publication in its present form.

Reviewer 2

The authors have clearly clarify my concerns.

Response to Reviewer 2:

We are very grateful to the Reviewer for the careful evaluation of our revised manuscript and for acknowledging that the concerns raised in the previous review have been clearly addressed. The Reviewer's comments were highly professional, perceptive, and academically rigorous, providing valuable guidance for improving the scientific presentation, precision, and completeness of the manuscript. We also sincerely appreciate the Reviewer's positive recognition of our revisions. The Reviewer's expertise and thoughtful assessment have made a significant contribution to strengthening the rigor and overall quality of this work.

Reviewer 3

The authors made meaningful and significant revisions to address reviewer comments, which has substantially strengthened the manuscript, particularly the addition of independent-chip statistics, the mixed-ion/drinking-water validation, and the refractive-index calibration comparing bare Au, Au+Graphene, Au+MoS_2, Au+BP and Au+BP-COOH. Most of my original concerns have been adequately addressed. Three points remain that require further clarification or substantiation before the manuscript is suitable for publication.

Response to Reviewer 3:

We greatly appreciate the Reviewer's thorough reassessment of our revised manuscript and the positive recognition of the substantial improvements made during the previous round of revision. We are particularly encouraged that the Reviewer acknowledged the value of the independent-chip statistics, the validation in mixed-ion solutions and drinking water, and the comparative refractive-index calibration of bare Au, Au+Graphene, Au+MoS_2, Au+BP, and Au+BP-COOH. The Reviewer's rigorous evaluation and discerning comments have been highly valuable in further strengthening the scientific foundation, clarity, and completeness of this work.

We also sincerely appreciate the Reviewer for identifying the three remaining issues that required further clarification or substantiation. These comments prompted us to provide additional experimental evidence for the interfacial built-in electric field, expand and clarify the discussion of the ion-selectivity mechanism, and more explicitly quantify and discuss the storage-dependent signal attenuation and possible strategies for improving long-term chip stability.We have carefully addressed each point and revised the manuscript and Supporting Information accordingly. Our detailed point-by-point responses and the corresponding changes are provided below.

Comment 1

The built-in electric field is still not fully supported. The revision relies on an energy-level schematic (Fig. 4c, d) without reporting actual work-function values for Au and BP, and no XPS data are provided to verify interfacial charge transfer between Au and BP. Corroborating spectroscopic and work-function measurements should be included as direct empirical support.

Response:

We thank the Reviewer for this precise and technically important comment. We agree that, in the previous version of the manuscript, the interfacial built-in electric field was inferred primarily from the energy-level schematic in Fig. 4c,d, and that direct experimental evidence of the work-function difference and interfacial charge redistribution was needed to substantiate this interpretation more rigorously. Following the Reviewer's suggestion, we performed ultraviolet photoelectron spectroscopy (UPS) measurements on bare Au, BP, and BP-COOH and acquired high-resolution Au 4f XPS spectra of bare Au, Au+BP, and Au+BP-COOH.

As shown in the newly added Fig. S4a-c, the experimentally determined work functions are 4.99 eV for bare Au, 4.30 eV for BP, and 4.26 eV for BP-COOH. Therefore, the corresponding work-function differences are 0.69 eV for Au/BP and 0.73 eV for Au/BP-COOH. Because the work functions of BP and BP-COOH are lower than that of Au, contact between these components drives electron transfer from BP or BP-COOH toward Au until Fermi-level equilibration is reached. This establishes the experimentally supported direction of interfacial charge transfer illustrated in Fig. 4c,d.

To independently verify this charge-transfer process spectroscopically, we further compared the high-resolution Au 4f XPS spectra of bare Au, Au+BP, and Au+BP-COOH, as presented in the newly added Fig. 4e-g. For bare Au, the Au $4f_{7/2}$ and Au $4f_{5/2}$ peaks are located at 84.26 and 87.98 eV, respectively. After contact with BP, these peaks shift to 83.50 and 87.18 eV, respectively. For Au+BP-COOH, they further shift to 83.34 and 86.98 eV, respectively. The systematic shifts toward lower binding energies are consistent with an increase in electron density on the Au side after interfacial contact. Importantly, the electron-transfer direction inferred from these Au 4f XPS shifts agrees with that determined from the measured work-function differences.

Accordingly, the UPS measurements provide quantitative work-function values and establish the expected direction of electron transfer, whereas the Au 4f XPS results independently confirm the associated increase in electron density on Au. Taken together, these complementary measurements provide direct empirical support for interfacial charge redistribution and the resulting built-in electric field at the Au/BP and Au/BP-COOH interfaces. We have revised Fig. 4, the corresponding figure caption, the main-text discussion, the summary of the physical mechanism, and the Characterization section accordingly. The UPS spectra and their quantitative work-function values have also been added to the Supporting Information as Fig. S4.

Changes made in the manuscript:

Manuscript, Lines 187-194:

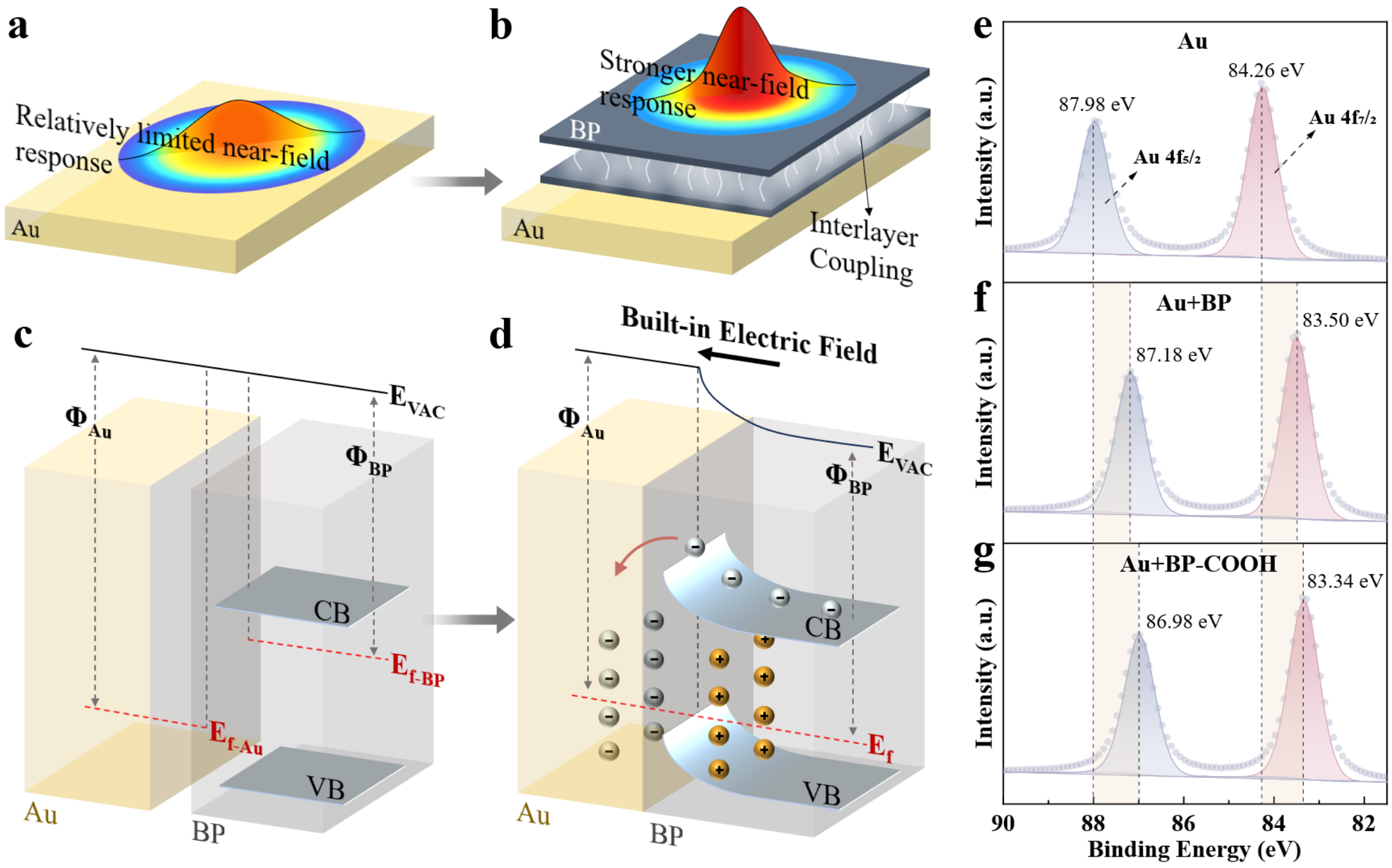

"Fig. 4. Schematic physical interpretation of BP-enhanced near-field response at the SPR interface and XPS characterization of interfacial charge redistribution. **(a)** Schematic near-field distribution at the Au/sensing-medium interface, showing a relatively limited near-field response. **(b)** Schematic near-field distribution after introduction of the BP overlayer, showing enhanced field confinement and a stronger near-field response; interlayer coupling within the multilayer BP structure is also illustrated. **(c)** Schematic energy-level alignment of Au and BP before interfacial charge redistribution, showing the work functions and band positions of the two

components. **(d)** Schematic interfacial charge-transfer process after Au/BP contact, showing the formation of a built-in electric field and the resulting enhancement of local field coupling at the interface. **(e–g)** High-resolution Au 4f XPS spectra of bare Au, Au+BP and Au+BP-COOH, respectively. The Au $4f_{7/2}$ and Au $4f_{5/2}$ peaks are located at 84.26 and 87.98 eV for bare Au, 83.50 and 87.18 eV for Au+BP, and 83.34 and 86.98 eV for Au+BP-COOH, respectively."

Manuscript, Lines 225-248:

"A second contribution is illustrated in Fig. 4c,d. UPS measurements give work functions of 4.99 eV for bare Au, 4.30 eV for BP and 4.26 eV for BP-COOH (Fig. S4). Thus, BP and BP-COOH exhibit comparable work functions, both lower than that of Au. The corresponding work-function differences are 0.69 eV for Au/BP and 0.73 eV for Au/BP-COOH. Upon contact, these work-function differences drive electron transfer from BP or BP-COOH toward Au until the Fermi levels reach equilibrium, leading to directional interfacial charge redistribution. The high-resolution Au 4f XPS spectra in Fig. 4e–g further support this process. The Au $4f_{7/2}$ and Au $4f_{5/2}$ peaks shift from 84.26 and 87.98 eV for bare Au to 83.50 and 87.18 eV for Au+BP, and to 83.34 and 86.98 eV for Au+BP-COOH. These shifts toward lower binding energies are consistent with increased electron density on the Au side after contact, in agreement with the electron-transfer direction indicated by the measured work-function differences. This interfacial charge redistribution leads to the formation of a built-in electric field at the Au/BP and Au/BP-COOH interfaces, which can further strengthen local electromagnetic coupling near the sensing region. In this sense, the role of BP in improving SPR sensitivity is not limited to adding an ultrathin overlayer, but also involves interfacial regulation of the local field environment. BP also exhibits a high carrier mobility, reported up to approximately 10^3 $cm^2/(V·s)$ at room temperature [24], which is favorable for efficient interfacial charge response. Taken together, the high-index confinement effect, multilayer coupling effect, and interfacial charge-transfer effect provide a physically reasonable interpretation for the stronger optical response of Au+BP and Au+BP-COOH observed in Fig. 3.

Taken together, Fig. 3, Fig. 4, Fig. S3 and Fig. S4 show that introducing the BP-based overlayer strengthens the interfacial optical response of the Au-film SPR structure to refractive-index variation. The experimental refractive-index calibration verifies the enhanced angular sensitivity of Au+BP and Au+BP-COOH, while the simulation provides a theoretical reference for the BP-overlayer-induced optical response. The physical interpretation illustrated in Fig. 4 is further supported by the UPS work-function measurements in Fig. S4 and the Au 4f XPS spectra in Fig. 4e–g, which together support electron transfer from BP or BP-COOH toward Au and the resulting interfacial charge redistribution and built-in electric field formation shown in Fig. 4c,d. These results provide the optical and interfacial basis for the subsequent improvement in Pb^{2+} sensing performance observed for Au+BP-COOH."

Manuscript, Lines 481-483:

"UPS measurements were performed using He I radiation with a photon energy of 21.22 eV under a −5 V sample bias, with a pass energy of 2.0 eV and an energy step of 0.020 eV. The Fermi-level reference was calibrated using an Ag standard. The work functions of bare Au, BP and BP-COOH are provided in Fig. S4."

Supporting Information, Lines 36-39:

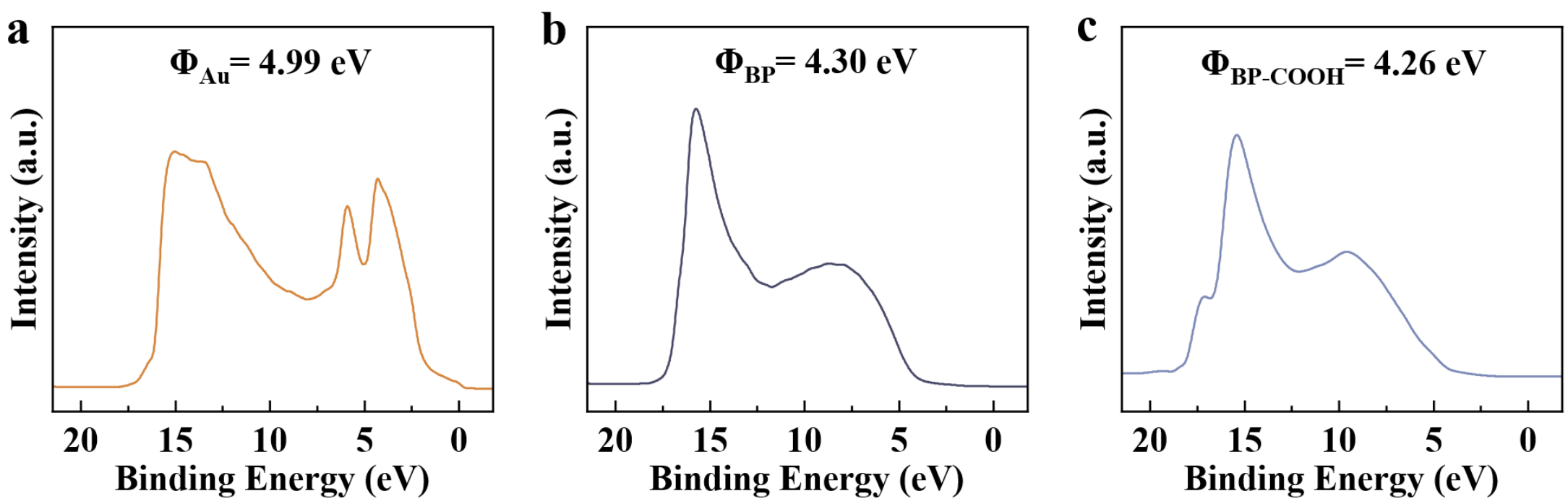

"**Fig. S4 UPS work-function spectra of bare Au, BP and BP-COOH. (a-c)** UPS spectra of bare Au, BP and BP-COOH, respectively, recorded using He I radiation with a photon energy of 21.22 eV under a −5 V sample bias. The calibrated work functions are 4.99 eV for bare Au, 4.30 eV for BP and 4.26 eV for BP-COOH."

Comment 2

The discussion on selectivity mechanism remains insufficient (Line 278-293). The HSAB theory can rationalize the superior binding performance of Pb^{2+} relative to hard hydrated Ca^{2+} and Mg^{2+}, yet it fails to clarify why the material exhibits far stronger preference for Pb^{2+} against Cu^{2+}, Cd^{2+}, Zn^{2+} and Ni^{2+}. A more targeted chemical rationale is required to rationalize the ion selectivity across the full range of tested metal ion species.

Response:

We greatly appreciate the Reviewer's insightful comment concerning the chemical origin of the preferential response toward Pb^{2+}. The Reviewer's observation prompted us to further enrich the mechanistic discussion by considering the distinct hydration and coordination characteristics of all tested metal ions, particularly Cu^{2+}, Cd^{2+}, Zn^{2+}, and Ni^{2+}.

In the revised manuscript, the HSAB-based interpretation has been retained and integrated with additional ion-dependent factors, including hydration-shell stability, dehydration and coordination-shell reorganization, ionic size and polarizability, coordination adaptability, and the accessibility of heterogeneous oxygen-containing sites on the BP-COOH surface. The combined consideration of these factors provides a more comprehensive and targeted chemical explanation for the experimentally observed ion-dependent response.

Pb^{2+} is a highly polarizable divalent ion with a stereochemically active $6s^2$ lone pair, which permits variable coordination numbers and asymmetric coordination geometries. Together with its borderline-soft acid character, these coordination characteristics may favor its interaction with the spatially heterogeneous oxygen-containing groups on BP-COOH, including carboxylate groups and oxidized phosphorus-containing sites.

Ca^{2+} and Mg^{2+} are strongly hydrated hard ions, and the presence of their stable hydration shells limits direct coordination with surface oxygen donors, particularly for Mg^{2+}. Cu^{2+}, Ni^{2+}, and Zn^{2+} can also coordinate with oxygen-containing groups; however, their interactions with the BP-COOH interface are influenced by ion-specific dehydration and the reorganization of their comparatively well-defined hydrated coordination structures. Cu^{2+} and Ni^{2+} possess particularly stable hydrated coordination environments, whereas Zn^{2+} does not exhibit the high polarizability and lone-pair-

associated coordination adaptability characteristic of Pb^{2+}.

Cd^{2+} is less strongly hydrated than Ca^{2+} and Mg^{2+} and is also capable of coordinating with oxygen-containing groups. Nevertheless, the higher polarizability of Pb^{2+} and its $6s^2$-lone-pair-associated coordination flexibility may provide a more favorable match with the spatially heterogeneous oxygen-containing sites on the BP-COOH surface. Accordingly, the experimentally observed response hierarchy may reflect the combined influence of hydration-shell stability, ionic size and polarizability, coordination geometry, HSAB interactions, and surface-site accessibility.

We have revised the corresponding paragraph in the main text to present this more comprehensive chemical interpretation and have incorporated relevant literature concerning the hydration and coordination properties of the tested divalent metal ions. The revised discussion specifically addresses the differences between Pb^{2+} and Cu^{2+}, Cd^{2+}, Zn^{2+}, and Ni^{2+}, while retaining HSAB interactions as one of the contributing factors.

The Discussion section has also been expanded to clarify the scope and significance of the proposed explanation. In future work, we will further investigate the ion-specific interfacial processes related to the preferential response toward Pb^{2+}. We hope that these studies will provide deeper insight into the underlying molecular mechanism and offer valuable guidance for the rational design of BP-based sensing interfaces with enhanced ion selectivity.

We sincerely value the Reviewer's constructive suggestion, which has enabled us to provide a more comprehensive, ion-specific, and chemically informative discussion of the selectivity mechanism.

Changes made in the manuscript:

Manuscript, Lines 296–305:

"The preferential response toward Pb^{2+} may arise from the combined effects of hydration-shell stability, ionic size and polarizability, coordination geometry, HSAB interactions, and the accessibility of oxygen-containing sites in BP-COOH. Pb^{2+} is a highly polarizable divalent ion with a stereochemically active $6s^2$ lone pair, allowing variable coordination numbers and asymmetric geometries; together with its borderline-soft acid character, this coordination flexibility may favor its interaction with surface oxygen-containing groups [31,33]. In comparison, Ca^{2+} and Mg^{2+} are strongly hydrated [34], whereas the interaction of Cu^{2+}, Ni^{2+} and Zn^{2+} with these sites may depend on ion-specific dehydration and reorganization of their more defined hydrated coordination structures [35]. Although Cd^{2+} is less strongly hydrated and can also coordinate with oxygen-containing groups [34,35], the higher polarizability and $6s^2$-lone-pair-associated coordination flexibility of Pb^{2+} may further favor its interfacial interaction [33]. The observed response hierarchy may therefore reflect the combined influence of these ion-dependent factors."

[33] Shimoni-Livny, L., Glusker, J. P. & Bock, C. W. Lone pair functionality in divalent lead compounds. Inorganic Chemistry 37, 1853–1867 (1998).

[34] Marcus, Y. Thermodynamics of solvation of ions. Part 5—Gibbs free energy of hydration at 298.15 K. Journal of the Chemical Society, Faraday Transactions 87, 2995–2999 (1991).

[35] Persson, I. Hydrated metal ions in aqueous solution: How regular are their structures? Pure and Applied Chemistry 82, 1901–1917 (2010).

Manuscript, Lines 372–381:

"The regeneration and storage-stability tests also show that Au+BP-COOH retains distinguishable Pb^{2+} sensing responses under the tested conditions, although the response decreases during storage. Compared with more stable two-dimensional materials, the storage stability of the present BP-based interface remains comparatively limited, but appropriate storage, passivation and packaging strategies offer feasible and promising routes for improvement. The preferential response of this system toward Pb^{2+} over the other tested ions may arise from differences in ion–surface interactions, and further investigation into the underlying mechanism represents an important and promising direction for future work. Overall, this work shows that direct interfacial engineering with a BP-COOH functional layer can substantially improve the performance of conventional label-free Au-film SPR for Pb^{2+} detection. These results provide a simple route for extending traditional Au-film SPR toward ultratrace heavy-metal-ion sensing."

Comment 3

The Day 1 to Day 7 storage data in Fig. S24 show an overall $\Delta\theta$ decay of ~65%, which seems high for a sensor intended for practical ultratrace monitoring. It is recommended to benchmark the observed decay rate against documented stability metrics of analogous 2D-material-based SPR sensors. The fundamental origin of this signal attenuation is suggested to be explicitly elucidated and propose feasible strategies for improving the long-term storage stability of the sensing chips.

Response:

We deeply appreciate the Reviewer's careful evaluation of the storage-stability results and the constructive recommendation to clarify the magnitude, possible origin, and practical implications of the observed signal attenuation. This comment prompted us to provide a more quantitative description of the storage-dependent response and to expand the corresponding discussion in the revised manuscript.

We first quantified the change in sensing response using the mean $\Delta\theta$ values obtained from independently prepared Au+BP-COOH chips. The mean $\Delta\theta$ decreased from 1581 mdeg on Day 1 to 557 mdeg on Day 7, corresponding to a decrease of 64.8% and retention of 35.2% of the initial response. These quantitative values are now explicitly reported in both the main manuscript and the Supporting Information. Although the response to 10^{-15} M Pb^{2+} remained clearly distinguishable at all tested storage time points, we acknowledge and have clarified in the revised manuscript that the magnitude of the response attenuation represents a limitation of the current Au+BP-COOH interface and should be carefully considered when evaluating its practical application.

We would also like to clarify the baseline procedure used in the storage-stability experiment. At each storage time point, pure water was first measured as the corresponding baseline, followed by measurement of 10^{-15} M Pb^{2+}. The reported $\Delta\theta$ was calculated as the net resonance-angle shift after subtraction of the pure-water baseline measured at the same time point. Therefore, the observed 64.8% decrease represents attenuation of the Pb^{2+}-induced sensing response rather than the accumulation of uncorrected baseline drift during the seven-day storage period. This experimental procedure has been clearly stated in the Methods section.

Following the Reviewer's recommendation, we also examined the reported storage-stability evaluations of analogous two-dimensional-material-assisted SPR sensors. However, a rigorous

numerical comparison is difficult because the available studies differ considerably in sensing architecture, two-dimensional material, target analyte, analyte concentration, storage environment, storage duration, chip-preparation procedure, baseline treatment, and definition of the stability metric. Moreover, many published two-dimensional-material-assisted SPR studies do not provide quantitative storage-dependent response retention over comparable periods. Consequently, directly comparing decay percentages obtained under substantially different experimental conditions may not provide a scientifically meaningful benchmark. We have therefore presented the absolute $\Delta\theta$ values, percentage decrease, and response-retention ratio of our sensing chips to enable a transparent and quantitative assessment of their storage performance.

We acknowledge that, compared with more chemically stable two-dimensional materials, such as graphene and several transition-metal dichalcogenides, BP is more susceptible to environmental degradation. The gradual attenuation of the sensing response may therefore be associated with the intrinsic environmental sensitivity of BP. Progressive exposure to oxygen and adsorbed water may alter the surface chemical state and optical properties of BP. In addition, storage-dependent changes in the BP-COOH nanosheet assembly may reduce the accessibility of carboxylate and other oxygen-containing sites involved in Pb^{2+} interaction. These processes may weaken both the interfacial interaction with Pb^{2+} and the corresponding SPR signal transduction.

To improve the long-term storage stability of the sensing chips, we have proposed several feasible strategies, including storage under oxygen- and moisture-limited conditions, optimization of protective polymer passivation while preserving the accessibility of Pb^{2+}-interaction sites, and sealed chip packaging to minimize environmental exposure. These possible origins and improvement strategies have been explicitly discussed in the revised manuscript.

Future work will further explore effective approaches for improving the storage protection and long-term response retention of the Au+BP-COOH sensing interface. Enhancing the environmental stability of the sensing chips while maintaining the accessibility of the Pb^{2+}-interaction sites represents a promising direction for advancing their practical applicability.

We are sincerely grateful for the Reviewer's valuable recommendation, which has helped us present the storage-stability results more quantitatively, clarify the baseline procedure, acknowledge the present limitation, and provide a more complete discussion of the possible causes and feasible improvement strategies.

Changes made in the manuscript:

Manuscript, Lines 315–325:

"Storage stability was evaluated using 10^{-15} M Pb^{2+}, with the sensing results provided in Figs. S19–S24 and the corresponding statistical results summarized in Fig. S25. The mean $\Delta\theta$ decreases from 1581 mdeg on Day 1 to 557 mdeg on Day 7, corresponding to a 64.8% decrease and retention of 35.2% of the initial response, although the Pb^{2+} response remains distinguishable at all tested time points. Within the broader context of two-dimensional-material-assisted SPR interfaces, this response retention indicates that storage stability remains a limitation of the present Au+BP-COOH interface [27,36]. The decrease may arise from gradual changes in the BP-COOH sensing layer during storage. BP is susceptible to oxidation in the presence of oxygen and adsorbed water, which may alter its surface and optical properties [25,26,37–39], while changes in nanosheet assembly and

the accessibility of oxygen-containing sites may also affect Pb^{2+} interaction and SPR transduction. Oxygen- and moisture-limited storage, optimized polymer passivation, and sealed chip packaging may improve the storage stability of the Au+BP-COOH interface [26,38–40].”

[36] Kaushik, S., Tiwari, U. K., Deep, A. & Sinha, R. K. Two-dimensional transition metal dichalcogenides assisted biofunctionalized optical fiber SPR biosensor for efficient and rapid detection of bovine serum albumin. Scientific Reports 9, 6987 (2019).

[37] Favron, A. et al. Photooxidation and quantum confinement effects in exfoliated black phosphorus. Nature Materials 14, 826–832 (2015).

[38] Wood, J. D. et al. Effective passivation of exfoliated black phosphorus transistors against ambient degradation. Nano Letters 14, 6964–6970 (2014).

[39] Abate, Y. et al. Recent progress on stability and passivation of black phosphorus. Advanced Materials 30, 1704749 (2018).

[40] Zhang, Y. et al. Black phosphorus/polymers: status and challenges. Advanced Materials 33, 2100113 (2021).

Manuscript, Lines 372–381:

“The regeneration and storage-stability tests also show that Au+BP-COOH retains distinguishable Pb^{2+} sensing responses under the tested conditions, although the response decreases during storage. Compared with more stable two-dimensional materials, the storage stability of the present BP-based interface remains comparatively limited, but appropriate storage, passivation and packaging strategies offer feasible and promising routes for improvement. The preferential response of this system toward Pb^{2+} over the other tested ions may arise from differences in ion–surface interactions, and further investigation into the underlying mechanism represents an important and promising direction for future work. Overall, this work shows that direct interfacial engineering with a BP-COOH functional layer can substantially improve the performance of conventional label-free Au-film SPR for Pb^{2+} detection. These results provide a simple route for extending traditional Au-film SPR toward ultratrace heavy-metal-ion sensing.”

Manuscript, Lines 460–468:

“The storage stability of Au+BP-COOH chips was evaluated using 10^{-15} M Pb^{2+} as a representative concentration. Day 1 corresponds to freshly prepared Au+BP-COOH chips measured immediately after fabrication. Days 2–7 correspond to Au+BP-COOH chips measured after 1, 2, 3, 4, 5 and 6 days of storage, respectively. Before measurement, the fabricated Au+BP-COOH chips were stored for the specified periods at 22 °C and 50% relative humidity under light-protected conditions. For Day 1, the statistical response was obtained from ten independently prepared Au+BP-COOH chips measured at 120 s. For Days 2–7, three independently prepared Au+BP-COOH chips were measured at each storage time point. At each time point, pure water was first measured as the baseline, followed by 10^{-15} M Pb^{2+}. The storage-stability response was defined as the net resonance-angle shift after subtracting the pure-water baseline.”

Supporting Information, Lines 113–118:

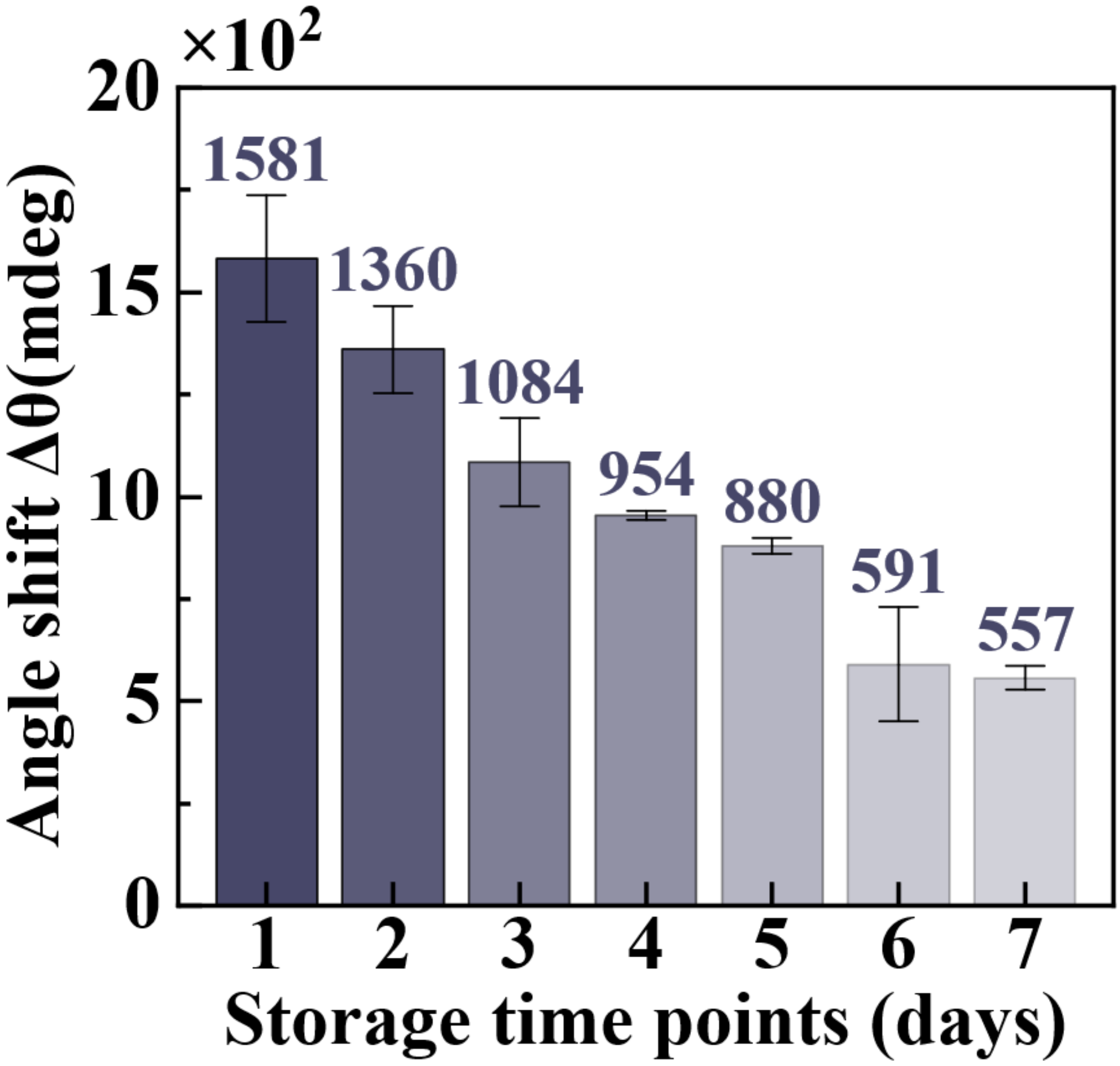

Fig. S25 Storage-stability statistics of Au+BP-COOH chips measured with 10^{-15} M Pb^{2+} at different storage time points. Resonance-angle shift Δθ of Au+BP-COOH chips measured with 10^{-15} M Pb^{2+} from Day 1 to Day 7. Day 1 corresponds to freshly prepared chips measured immediately after fabrication, and Days 2–7 correspond to chips measured after 1, 2, 3, 4, 5 and 6 days of storage, respectively. Error bars are from independently prepared Au+BP-COOH chips. The mean Δθ decreases from 1581 mdeg on Day 1 to 557 mdeg on Day 7, corresponding to a 64.8% decrease and retention of 35.2% of the initial response.
